# Microarray analysis of *Pseudomonas aeruginosa *reveals induction of pyocin genes in response to hydrogen peroxide

**DOI:** 10.1186/1471-2164-6-115

**Published:** 2005-09-08

**Authors:** Wook Chang, David A Small, Freshteh Toghrol, William E Bentley

**Affiliations:** 1 Center for Biosystems Research, University of Maryland Biotechnology Institute, College Park, Maryland 20742, USA; 2 Microarray Research Laboratory, Biological and Economic Analysis Division, Office of Pesticide Programs, U. S. Environmental Protection Agency, Fort Meade, Maryland 20755, USA

## Abstract

**Background:**

*Pseudomonas aeruginosa*, a pathogen infecting those with cystic fibrosis, encounters toxicity from phagocyte-derived reactive oxidants including hydrogen peroxide during active infection. *P. aeruginosa *responds with adaptive and protective strategies against these toxic species to effectively infect humans. Despite advances in our understanding of the responses to oxidative stress in many specific cases, the connectivity between targeted protective genes and the rest of cell metabolism remains obscure.

**Results:**

Herein, we performed a genome-wide transcriptome analysis of the cellular responses to hydrogen peroxide in order to determine a more complete picture of how oxidative stress-induced genes are related and regulated.

Our data reinforce the previous conclusion that DNA repair proteins and catalases may be among the most vital antioxidant defense systems of *P. aeruginosa*. Our results also suggest that sublethal oxidative damage reduces active and/or facilitated transport and that intracellular iron might be a key factor for a relationship between oxidative stress and iron regulation. Perhaps most intriguingly, we revealed that the transcription of all F-, R-, and S-type pyocins was upregulated by oxidative stress and at the same time, a cell immunity protein (pyocin S2 immunity protein) was downregulated, possibly leading to self-killing activity.

**Conclusion:**

This finding proposes that pyocin production might be another novel defensive scheme against oxidative attack by host cells.

## Background

Many microorganisms continuously face a range of reactive oxygen species (ROS) including hydrogen peroxide, superoxide, and the hydroxyl radical derived from many sources. During the process of active infection, pathogenic bacteria are exposed to exogenous oxidative stress that phagocytes utilize as a host defense mechanism [1]. Even normal cellular metabolism produces cytotoxicity arising from its partially-reduced intermediates [2]. For instance, by reacting with intracellular iron, hydrogen peroxide can form the hydroxyl radical through the Fenton reaction, which damages various cellular molecules including lipids, proteins, and DNA [1,3]. Superoxide is also capable of promoting oxidative damage by increasing the concentration of intracellular iron [2,4,5]. Because of the vast array of stimuli and their sources, it is intuitive to anticipate that organisms have developed complex antioxidant strategies that serve to neutralize and repair oxidative damage.

*Pseudomonas aeruginosa *PA01 (*P. aeruginosa*), a Gram-negative pathogen responsible for respiratory infections in individuals with cystic fibrosis and cancer, is also known to possess a multifaceted defense system against reactive oxidants that includes such enzymes as catalase and superoxide dismutase [6-8]. There are many specific defense genes that have been identified and regulatory aspects of their activities have been elucidated in many cases [1,5]. Despite this marked progress, cystic fibrosis remains problematic and our knowledge of *P. aeruginosa *pathogenicity remains incomplete. A more thorough understanding of this bacterium's defense system might serve to enhance the development and efficacy of therapeutic agents for this disease. In particular, an understanding of the linkage between the cell's ROS defense mechanism and the remainder of the cell's metabolism can lead to more innovative methods for combating this pathogen. For example, better elucidation of the molecular events responsible for establishing and maintaining pathogenicity might improve optimal drug and vaccine design [9]. That is, by using microarray analysis that enables us to simultaneously and globally examine the complete transcriptome during cellular responses, we might reinforce known relationships between genes with previously identified functions, and also reveal new target genes that give us more insight into *P. aeruginosa*-host interactions.

To provide a more complete linkage between cell physiology and the well-characterized defense response, we investigated genome-wide changes in *P. aeruginosa *gene transcription upon exposure to hydrogen peroxide using Affymetrix *P. aeruginosa *GeneChip arrays. Notably, we made a significant finding that hydrogen peroxide induced the transcription of each and every pyocin (bacteriocins) reported in *P. aeruginosa*. Moreover, we found that a pyocin immunity gene, which prevents bacterial cell death during pyocin synthesis, was downregulated, possibly leading to self-killing activity. Finally, we've corroborated an anticipated result regarding iron uptake; that oxidative stress in our experimental conditions lead to the repression of iron uptake genes.

## Results and discussion

To investigate the effect of sublethal oxidative stress on *P. aeruginosa*, we performed a transcriptome analysis with microarrays upon 20 min exposure to 1 mM hydrogen peroxide. This concentration successfully induces sublethal oxidative damage in *Escherichia coli *and *P. aeruginosa *[10-12]. Besides providing requisite quantities of mRNA for microarray analyses, sublethal doses of antibiotics are receiving increased interest because of their potential for attenuating pathogenicity but with concern for increased rates of mutation and resistance [13,14]. We confirmed that 1 mM hydrogen peroxide caused strong growth inhibition but not cell death for the first 60 min post-treatment (data not shown).

To determine genome-wide transcriptional changes in response to hydrogen peroxide, we conducted four and five independent microarray experiments in the absence (control) and the presence (experimental) of hydrogen peroxide, respectively. Transcriptome analysis with Affymetrix *P. aeruginosa *GeneChip arrays suggested that mRNA levels of 805 and 827 out of a total of 5,570 genes were increased and decreased (IR <> 1), respectively, after 20 min treatment. We refer to "statistically marked" changes in transcript level for those genes that meet the following criteria: (i) a *p-*value for a Mann-Whitney test should be less than 0.05, (ii) an absolute fold change in transcript level should be equal to or greater than 2, and (iii) a gene should have a presence or marginal call [15] from 50% or more replicates on both the experimental and control replicate sets. Based on these criteria, we pared the set to 116 and 107 genes (a total of 223) that had statistically marked increases and decreases in transcript level, respectively. This result suggests that these genes might be associated with the defensive response of *P. aeruginosa *to hydrogen peroxide-induced oxidative stress. Interestingly, this number is comparable to that found in a study of *E. coli *cells treated with 1 mM hydrogen peroxide (140 genes, = 4-fold) [16], and significantly less than those of a comparable *P. aeruginosa *study (1,854 genes, ≥ 2-fold) [12]. In Table 1, from among the 223 genes, we list 30 most strongly induced and repressed genes as well as their putative functions, many of which are discussed below. Additionally, Supplementary Table 1 shows the average signals, *p*-values, and fold changes of all 5,570 predicted *P. aeruginosa *open reading frames. The data discussed in this publication have been deposited in NCBI's Gene Expression Omnibus [17] and are accessible through GEO Series accession number GSE3090.

**Table 1:** List of 30 *P. aeruginosa *genes most strongly induced and repressed in response to hydrogen peroxide

Gene (Name)	Fold change	*p*-value	Protein (Function)
*Induced Genes*			
PA5530	24.7	0.008	Probable MFS dicarboxylate transporter (Membrane proteins; Transport of small molecules)
PA1466	13.9	0.008	Hypothetical protein (Hypothetical, unclassified, unknown)
PA2288	10.3	0.008	Hypothetical protein (Hypothetical, unclassified, unknown)
PA3414	9.8	0.008	Hypothetical protein (Hypothetical, unclassified, unknown)
PA4763 (*recN*)	8.3	0.008	DNA repair protein (DNA replication, recombination, modification and repair)
PA0182	6.8	0.024	Probable short-chain dehydrogenase (Putative enzymes)
PA3008	5.7	0.008	Hypothetical protein (Hypothetical, unclassified, unknown)
PA0670	5.4	0.008	Hypothetical protein (Hypothetical, unclassified, unknown)
PA0612	5.3	0.008	Hypothetical protein (Hypothetical, unclassified, unknown)
PA0641	5.0	0.008	Probable bacteriophage protein (Related to phage, transposon, or plasmid)
PA0671	5.0	0.008	Hypothetical protein (Hypothetical, unclassified, unknown)
PA4613 (*katB*)	4.5	0.024	Catalase (Adaptation, protection)
PA3413	4.4	0.008	Hypothetical protein (Hypothetical, unclassified, unknown)
PA0620	4.2	0.008	Probable bacteriophage protein (Related to phage, transposon, or plasmid)
PA0615	4.2	0.008	Hypothetical protein (Hypothetical, unclassified, unknown)
PA5471	4.1	0.024	Hypothetical protein (Hypothetical, unclassified, unknown)
PA5470	4.1	0.024	Probable peptide chain release factor (Translation, post-translational modification, degradation)
PA3007 (*lexA*)	4.0	0.008	Repressor protein (Adaptation, protection; Translation, post-translational modification, degradation)
PA0922	3.7	0.008	Hypothetical protein (Hypothetical, unclassified, unknown)
PA0635	3.6	0.008	Hypothetical protein (Related to phage, transposon, or plasmid)
PA0616	3.5	0.008	Hypothetical protein (Hypothetical, unclassified, unknown)
PA0634	3.5	0.008	Hypothetical protein (Related to phage, transposon, or plasmid)
PA0625	3.4	0.008	Hypothetical protein (Related to phage, transposon, or plasmid)
PA0636	3.4	0.008	Hypothetical protein (Related to phage, transposon, or plasmid)
PA0617	3.4	0.008	Probable bacteriophage protein (Hypothetical, unclassified, unknown)
PA0633	3.3	0.008	Hypothetical protein (Related to phage, transposon, or plasmid)
PA0637	3.3	0.008	Conserved hypothetical protein (Related to phage, transposon, or plasmid)
PA3923	3.3	0.008	Hypothetical protein (Hypothetical, unclassified, unknown)
PA4582	3.2	0.008	Conserved hypothetical protein (Hypothetical, unclassified, unknown)
PA0638	3.2	0.008	Probable bacteriophage protein (Hypothetical, unclassified, unknown)

*Repressed Genes*			
PA2398 (*fpvA*)	5.7	0.008	Ferripyoverdine receptor (Transport of small molecules)
PA4156	5.1	0.008	Probable TonB-dependent receptor (Transport of small molecules)
PA4230 (*pchB*)	4.3	0.008	Salicylate biosynthesis protein (Transport of small molecules; Secreted Factors)
PA2405	3.9	0.008	Hypothetical protein (Hypothetical, unclassified, unknown)
PA2409	3.7	0.008	Probable permease of ABC transporter (Membrane proteins; Transport of small molecules)
PA5235 (glpT)	3.4	0.008	Glycerol-3-phosphate transporter (Membrane proteins; Transport of small molecules)
PA4225 (pchF)	3.3	0.008	Pyochelin synthetase (Transport of small molecules; Secreted Factors)
PA2407	3.3	0.008	Probable adhesion protein (Motility & Attachment)
PA2403	3.2	0.008	Hypothetical protein (Hypothetical, unclassified, unknown; Membrane proteins)
PA5479 (*gltP*)	3.2	0.008	Proton-glutamate symporter (Membrane proteins; Transport of small molecules)
PA3009	3.1	0.008	Hypothetical protein (Hypothetical, unclassified, unknown)
PA2426 (*pvdS*)	3.0	0.024	Sigma factor (Transcriptional regulators)
PA2404	3.0	0.008	Hypothetical protein (Hypothetical, unclassified, unknown; Membrane proteins)
PA2667	3.0	0.008	Conserved hypothetical protein (Transcriptional regulators)
PA3610 (*potD*)	3.0	0.008	Polyamine transport protein (Transport of small molecules)
PA4555 (*pilY2*)	3.0	0.008	Type 4 fimbrial biogenesis protein (Motility & Attachment)
PA1414	2.9	0.024	Hypothetical protein (Hypothetical, unclassified, unknown)
PA0280 (*cysA*)	2.9	0.024	Sulfate transport protein (Transport of small molecules)
PA2408	2.9	0.008	Probable ATP-binding component of ABC transporter (Transport of small molecules)
PA4839 (*speA*)	2.9	0.008	Biosynthetic arginine decarboxylase (Amino acid biosynthesis and metabolism)
PA4231 (*pchA*)	2.9	0.008	Salicylate biosynthesis isochorismate synthase (Secreted Factors; Transport of small molecules)
PA0281 (*cysW*)	2.8	0.008	Sulfate transport protein (Membrane proteins; Transport of small molecules)
PA1228	2.8	0.008	Hypothetical protein (Hypothetical, unclassified, unknown)
PA5049 (*rpmE*)	2.8	0.008	50S ribosomal protein (Translation, post-translational modification, degradation)
PA2619 (*infA*)	2.7	0.008	Initiation factor (Translation, post-translational modification, degradation)
PA4221 (*fptA*)	2.7	0.008	Fe(III)-pyochelin receptor precursor (Transport of small molecules)
PA0976	2.7	0.008	Conserved hypothetical protein (Hypothetical, unclassified, unknown)
PA5446	2.7	0.048	Hypothetical protein (Hypothetical, unclassified, unknown)
PA4229 (*pchC*)	2.6	0.008	Pyochelin biosynthetic protein (Transport of small molecules; Secreted Factors)
PA4218	2.6	0.008	Probable transporter (Membrane proteins; Transport of small molecules)

To validate the relative transcript levels obtained by the array analysis, we employed quantitative real-time PCR analysis on PA4613 (*katB*), PA2850 (*ohr*), PA4763 (*recN*), and PA5530. These genes were selected since they displayed a range of mRNA level changes (-1- to 24-fold). Moreover, PA0576 (*rpoD*) was used as a control gene for the relative mRNA level calculation due to the fact that *rpoD *exhibits stable expression level [18]. As shown in Table 2, our microarray results were corroborated with quantitative real-time PCR analyses of the selected genes.

**Table 2:** Transcript level comparison of *P. aeruginosa *genes between real-time PCR analysis and microarray analysis

Gene	mRNA level change		Sense primer sequence (5'-3')	Antisense primer sequence (5'-3')
			
	real-time PCR	microarray		
PA4613	1.69 (± 0.44)	4.54	GAGCAGAACTTCAAGCAGAC	CTCTCGTCGTCGGTGATC
PA2850	-1.64 (± 0.19)	-1.37	GAGGTCGAACTGCACATC	GGGTAGCGTTGGAGTAGG
PA4763	4.24 (± 0.17)	8.27	GGAGCAGGAGCAGAAGAC	GTTGAGGCTGGCATTGAG
PA5530	34.26 (± 7.17)	24.70	AAGAAGGAAGAGCCGAAGG	ATGTAGGTGGTGTAGGTGTAG
PA0576			CGTCCTCAGCGGCTATATCG	TTCTTCTTCCTCGTCGTCCTTC

### Functional classification

**Figure 1:**
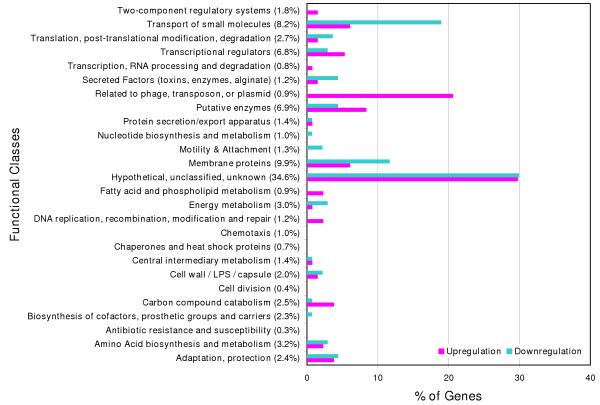
**Functional classification of genes with statistically significant increase and decrease in mRNA level (a total of 223 genes)**. The number in parenthesis represents the percentage of the total number of genes within the genome in each functional class.

To examine how the genes are distributed with regard to their functions, we classified these 223 genes according to the categories described in the *Pseudomonas aeruginosa *Community Annotation Project [19] (see, Figure 1). Both Figure 1 and Table 1 indicate that the most distinctive feature was the completely uniform upregulation of genes involved in DNA modulation (Phage, transposon, or plasmid and DNA replication and repair); this is discussed further below. It also appeared that genes related to various membrane functions including "transport of small molecules", "secreted factors", and "membrane proteins" were noticeably downregulated, suggesting that hydrogen peroxide alters the regulation of membrane proteins. Even among the 30 most downregulated genes presented in Table 1, 17 genes belonged to these functional classes (PA2398 (*fpvA*), PA4156, PA4230 (*pchB*), PA2409, PA5235 (*glpT*), PA4225 (*pchF*), PA2403, PA5479 (*gltP*), PA2404, PA3610 (*potD*), PA0280 (*cysA*), PA2408, PA4231 (*pchA*), PA0281 (*cysW*), PA4221 (*fptA*), PA4229 (*pchC*), and PA4218). This result may reflect attenuation of active and/or facilitated transport through the cell membrane. Notably, many genes associated with these classes were found linked with iron regulation (discussed below). Also, consistent with the sublethal but significant applied stress, as shown in Table 3, several genes involved in primary metabolism exhibited decreased mRNA levels: (i) energy metabolism-related genes, PA1317 (*cyoA*), PA1319 (*cyoC*), and PA3621 (*fdxA*), and PA4133, (ii) polyamine synthesis and uptake genes (related to amino acid synthesis and metabolism), PA0654 (*speD*), PA1687 (*speE*), PA4839 (*speA*), and PA3607-3610 (*potABCD*), and (iii) ribosomal protein genes, PA4432 (*rpsL*), PA4563 (*rpsT*), PA5049 (*rpmE*), and PA5315 (*rpmG*). It is also interesting that putative cell division inhibitors such as PA0671 and PA3008, which are similar to *E. coli sulA *[20], showed increased transcript levels upon exposure to hydrogen peroxide, suggesting that these genes might promote the repression of primary metabolism.

**Table 3:** List of *P. aeruginosa *genes that were discussed in this report, categorized by their related functions.

Gene (Name)	Average signal of experimentals	Average signal of controls	*p*-value	Fold change ^a^
*Primary metabolism-related*				
PA1317 (*cyoA*)	70.5	159.9	0.048	-2.3
PA1319 (*cyoC*)	22.5	46.6	0.008	-2.1
PA3621 (*fdxA*)	321.7	648.6	0.008	-2.0
PA4133	46.8	106.6	0.024	-2.3
PA0654 (*speD*)	416.9	804.4	0.008	-1.9 ^c^
PA1687 (*speE*)	77.8	204.8	0.008	-2.6
PA4839 (*speA*)	111.7	321.2	0.008	-2.9
PA3607 (*potA*)	65.8	143.7	0.008	-2.2
PA3608 (*potB*)	25.8	64.6	0.008	-2.5
PA3609 (*potC*)	40.0	83.00	0.008	-2.1
PA3610 (*potD*)	35.4	105.9	0.008	-3.0
PA4432 (*rpsL*)	493.8	1156.9	0.008	-2.3
PA4563 (*rpsT*)	1600.9	3648.5	0.008	-2.3
PA5049 (*rpmE*)	174.9	482.0	0.008	-2.8
PA5315 (*rpmG*)	340.3	761.5	0.008	-2.2
PA0671	127.5	25.4	0.008	5.0
PA3008	618.6	109.2	0.008	5.7
*Cellular protective mechanism-related*				
PA4236 (*katA*)	543.8	275.1	0.008	2.0
PA4613 (*katB*)	152.6	33.6	0.008	4.5
PA4366 (*sodB*)	1779.4	1923.3	0.484 ^b^	-1.1 ^c^
PA4468 (*sodM*)	27.7	18.8	0.008	1.5 ^c^
PA3007 (*lexA*)	1267.9	320.9	0.008	4.0
PA3008	618.6	109.2	0.008	5.7
PA3616	289.7	105.2	0.008	2.8
PA3617 (*recA*)	1917.5	706.1	0.008	2.7
PA0669	59.4	20.9	0.008	2.8
PA3413	766.0	174.7	0.008	4.4
PA3414	459.5	47.0	0.008	9.8
PA4763 (*recN*)	1270.9	153.6	0.008	8.3
*Iron regulation-related*				
PA2426 (*pvdS*)	21.0	63.0	0.024	-3.0
PA2398 (*fpvA*)	99.5	566.1	0.008	-5.7
PA4221 (*fptA*)	154.6	417.9	0.008	-2.7
PA4225 (*pchF*)	42.1	139.0	0.008	-3.3
PA4226 (*pchE*)	103.3	263.0	0.008	-2.6
PA4228 (*pchD*)	101.1	235.1	0.008	-2.3
PA4229 (*pchC*)	101.2	267.6	0.008	-2.6
PA4230 (*pchB*)	49.6	212.1	0.008	-4.3
PA4231 (*pchA*)	35.6	102.6	0.008	-2.9
PA2403	53.7	173.8	0.008	-3.2
PA2404	45.6	138.1	0.008	-3.0
PA2405	46.9	181.1	0.008	-3.9
PA2406	61.4	113.3	0.008	-1.8 ^c^
PA2407	91.8	300.9	0.008	-3.3
PA2408	35.8	103.7	0.008	-2.9
PA2409	50.9	188.6	0.008	-3.7
PA2410	38.2	94.4	0.008	-2.5
PA4156	3.2	16.3	0.008	-5.1
PA3531 (*bfrB*)	114.0	227.5	0.008	-2.0
*Pyocin system-related*				
PA0985	86.9	45.2	0.024	1.9 ^c^
PA1150 (*pys2*)	359.3	123.9	0.008	2.9
PA3866	129.6	58.9	0.008	2.2
PA0612	941.5	177.8	0.008	5.3
PA0613	220.4	96.1	0.008	2.3
PA0614	635.5	220.0	0.008	2.9
PA0615	708.0	169.1	0.008	4.2
PA0616	1177.1	336.0	0.008	3.5
PA0617	708.9	211.5	0.008	3.4
PA0618	1085.4	350.1	0.008	3.1
PA0619	1189.2	392.5	0.008	3.0
PA0620	1328.8	313.4	0.008	4.2
PA0621	1909.6	742.5	0.008	2.6
PA0622	2536.5	833.8	0.008	3.0
PA0623	2053.5	740.5	0.008	2.8
PA0624	960.5	382.8	0.008	2.5
PA0625	537.4	159.5	0.008	3.4
PA0626	633.5	272.6	0.008	2.3
PA0627	630.3	270.0	0.008	2.3
PA0628	652.2	297.4	0.008	2.2
PA0629	407.9	154.8	0.008	2.6
PA0630	416.1	211.9	0.008	2.0
PA0631	294.6	128.3	0.008	2.3
PA0632	270.8	143.8	0.008	1.9 ^c^
PA0633	1979.9	596.6	0.008	3.3
PA0634	589.4	169.8	0.008	3.5
PA0635	836.9	232.3	0.008	3.6
PA0636	1278.8	380.6	0.008	3.4
PA0637	589.8	178.4	0.008	3.3
PA0638	908.6	283.9	0.008	3.2
PA0639	617.9	209.7	0.008	3.0
PA0640	142.0	55.6	0.008	2.6
PA0641	509.7	102.0	0.008	5.0
PA0642	45.1	16.2	0.008	2.8
PA0643	135.5	65.7	0.008	2.1
PA0644	210.3	119.3	0.008	1.8 ^c^
PA0645	222.9	88.8	0.008	2.5
PA0646	158.0	64.2	0.008	2.5
PA0647	50.84	39.95	0.341 ^b^	1.3 ^c^
PA0648	143.4	94.8	0.024	1.5 ^c^
PA1151 (*imm2*)	34.88	71.58	0.008	-2.1

^a^The fold change is a positive number when the expression level in the experiment increased compared to the control and is a negative number when the expression level in the experiment declined.

^b ^The *p *value is higher than 0.05.

^c ^The fold change is less than 2.

### Genes related to cellular protective mechanisms

Figure 1 and Table 3 show that a number of genes primarily in the classes of "adaptation, protection" and "DNA replication, recombination, modification and repair", which are known to be involved in cellular protective mechanisms, were induced in response to hydrogen peroxide. First, two catalase genes, PA4236 (*katA*), and PA4613 (*katB*) showed significant increases in mRNA levels. It was reported that the KatB enzyme is essential for optimal resistance to hydrogen peroxide [6,21]. The mere 2-fold upregulation of *katA *may support a prior hypothesis that *katA *serves as a primary housekeeping catalase; expressed constitutively throughout the growth cycle [7,21]; as a result, dramatic transcript increases might not occur. Surprisingly, superoxide dismutase genes, PA4366 (*sodB*) and PA4468 (*sodM*), did not show significant increases in mRNA levels. However, *sodB *was among genes that had the highest signal intensities on both control and experimental replicates, whereas *sodM *exhibited low mRNA levels under both conditions. Again, this result may correspond well to a previous report suggesting that SodB expression may be important even during normal aerobic growth while SodM activity is increased only in response to iron deprivation, which is not the case in our study (discussed below) [22].

Second, DNA repair-related genes were highly induced in the presence of hydrogen peroxide. Indeed, it is known that hydrogen peroxide causes oxidative DNA damage by generating hydroxyl or ferryl radicals [1,2,23]. Palma *et al*. demonstrated that genes of *P. aeruginosa *SOS regulon exhibit increases in mRNA level upon exposure to 1 mM hydrogen peroxide [12]. Reportedly, RecA stimulates autodigestion of repressor protein LexA for the activation of the SOS regulon in *E. coli *[24]. Our study also revealed increased expression of PA3007 (*lexA*) and PA3617 (*recA*) (Table 3). Furthermore, PA3008, adjacent to *lexA*, showed high increase in transcript level; notably, this gene is similar to *E. coli sulA*, which inhibits cell division until DNA repair is finished [12,25]. Besides, PA3616 (probable *recX*), a downstream gene of *recA*, is reportedly involved in regulation of RecA expression [26,27]. Other interesting genes possibly relevant to DNA repair-related functions included PA0669 (a DNA polymerase gene), PA3413-PA3414, and PA4763 (*recN*, a DNA repair gene). To be specific, a protein encoded by PA3414 exhibited a high homology to YbeG in *E. coli*, which is inducible by DNA damage [28,29]. Moreover, the fact that DnaE, similar to a product encoded by PA0669, is involved in DNA mismatch correction or DNA repair in *Bacillus subtilis *suggests that the PA0669 protein might also participate in repairing DNA damage caused by hydrogen peroxide in our study [30,31].

**Figure 2:**
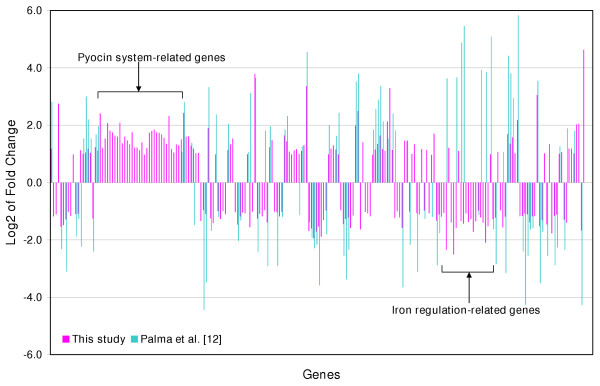
**Comparison of 223 genes with statistically significant mRNA level changes in this study with those of Palma *et al*. [12]**. The fold change is a positive number when the expression level in the experiment increased compared to the control and is a negative number when the expression level in the experiment declined.

As shown in Figure 2, the complete profile of our response is compared directly to that of Palma *et al*. [12]. Our results were generally consistent and, overall, this portion of our analysis corroborates previous studies that have associated oxidative stress response genes with hydrogen peroxide and other ROS insults, and reinforces the conclusion that DNA repair proteins and catalases may be among the most central mechanisms that *P. aeruginosa *employs to counteract lethal effects of reactive oxygen intermediates.

### Genes related to iron regulation

As mentioned above, however, one of the aims of this study was to examine the responses of ROS stress genes to the rest of cellular metabolism, in order to provide new insight on *P. aeruginosa *regulation in response to varied oxygen conditions as is expected in niche environments of the human lung. Among the more interesting findings was an apparent discrepancy between previous reports of iron-regulated genes. These genes are highlighted in Figure 2. Iron metabolism is important for many reasons: there are many iron catalyzed oxygen reactions that can damage cells; there are many sources of iron, particularly localized in the human lung; and iron-related genes are often coordinately regulated with oxidative stress defenses in *E. coli *[32]. Notably, this study also revealed that hydrogen peroxide treatment drastically altered iron regulation in *P. aeruginosa*. In particular, the following genes, all of which are known to be regulated by the ferric uptake regulator (Fur, encoded by PA4764), were repressed in response to hydrogen peroxide (Table 3): (i) iron starvation sigma factor, PA2426 (*pvdS*), (ii) ferri-siderophore (iron-chelating compound) receptor genes, PA2398 (*fpvA*) and PA4221 (*fptA*), (iii) siderophore (pyochelin) biosynthesis genes, PA4225-PA4226 (*pchFE*) and PA4228-PA4231 (*pchDCBA*), and (iv) siderophore (pyoverdin) system-related genes, PAPA2403-PA2410 and PA4156 [33,34].

As is widely accepted, in order to overcome a limited supply of iron, *P. aeruginosa *produces two major siderophores, pyoverdin and pyochelin [34-36] and upon iron deprivation, these siderophores are excreted from the cells, chelate iron and transport it back to the cells through outer membrane receptors, FptA and FpvA, which are specific for the iron-siderophore complexes [37,38]. Moreover, in the absence of iron, PchR, encoded by PA4227, seems to become a repressor of the receptor gene, *fptA*, and of its own gene, *pchR*. On the other hand, excess iron represses the biosynthesis of pyoverdin and pyochelin [35,39]; that is, in the presence of abundant iron, the Fur protein, a repressor, binds to the promoters of genes encoding PvdS and PchR proteins, which positively regulate the biosynthesis of pyoverdin and pyochelin, respectively [38]. These phenomena seem congruent with the transcript levels of iron-related genes in this study.

It has been documented that the intracellular iron level could be affected by oxidative stress [3,5,32]. In particular, superoxide, generated during the reduction process of oxygen, releases free iron from iron-sulfur proteins, thus increasing the levels of intracellular iron [2-4]. Consequently, the result that the iron regulation-related genes such as *pvdS*, *fpvA*, *fptA*, and *pchABCDEF *were repressed in our study could be accounted for by prior reports suggesting that the amount of intracellular iron might be increased as a result of exposure to reactive oxidants [4]. At this point, it should be pointed out that "free iron" was intended to suggest not that the iron is hexa-aqueous, but that the iron is not integrated into enzymes. Iron vigorously binds to most biomolecules so that iron atoms free inside the cell likely exist in a form associated with the surfaces of cellular molecules. As shown in Figure 2, Palma *et al*. previously reported *induction *of several iron starvation-inducible genes such as PA0471, PA0472, PA2426 (*pvdS*), PA2686 (*pfeR*), PA4221 (*fptA*), PA4227 (*pchR*), PA4228 (*pchD*), PA4230 (*pchB*), and PA5531 (*tonB*) upon exposure to hydrogen peroxide, suggesting that cells experience iron starvation and/or a transient loss of Fur repressor function [12]. The reason for this discrepancy is not understood at present; however, it should not be excluded that different growth mediums, exposure times, and/or growth phase (tryptic soy broth, 10 min, optical density 0.5, respectively) as well as a different *P. aeruginosa *isolates were used in the two studies (discussed in more detail later).

Lastly, it should be pointed out that iron regulation system might be an attractive therapeutic target because it appears to be closely related to the antioxidant mechanism of *P. aeruginosa*. For instance, siderophore malfunction by potent drugs could lead to inhibition and/or imbalance of iron regulation and thus, could increase the cell's susceptibility to exogenous oxidative stress that phagocytes utilize during active infection.

### Genes related to pyocin system

**Figure 3:**
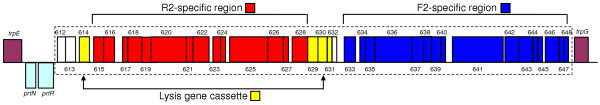
**Genetic organization of the R2/F2 pyocin gene locus according to Michel-Briand and Baysse [40] and Nakayama *et al*. [41]**. Each box represents a gene between PA0609 (*trpE*) and PA0649 (*trpG*). Genes inside the dotted line were upregulated upon exposure to hydrogen peroxide (see, Table 3).

The most striking result of our study, which has not been shown previously in any oxygen-insult studies of any bacterium, was that F-, R- and S- type pyocins, bacteriocins of *P. aeruginosa*, were strongly induced in response to hydrogen peroxide. As shown in Figure 2, these 41 genes were uniformly upregulated. Many strains are known to adapt bacteriocins so as to preserve the initial predominance of bacteriocinogenic bacteria [40]; however, bacteriocins are effective only against the same or closely related species [41-43]. Surprisingly, our study revealed that PA0985 (probable pyocin S5 gene), PA1150 (*pys2*, pyocin S2 gene), and PA3866 (probable pyocin S3 gene) were induced upon exposure to hydrogen peroxide (Table 3). Further, as shown in Figure 3 and Table 3, all the genes of a R2/F2 pyocin gene locus (PA0612-PA0648) exhibited increased mRNA levels (Figure 1, the "phage, transposon, and plasmid" class). Thus, R2 pyocin (PA0615 – PA0628), F2 pyocin (PA0633 – PA0648), and lysis enzymes (PA0614, PA0629 – PA0631) were likely expressed with the hydrogen peroxide treatment (Figure 3). Pyocins cause cell death by DNA breakdown and the inhibition of lipid synthesis; their production is inducible by mutagenic agents such as UV and mitomycin C [40,44]. RecA (a DNA repair protein), PrtR (a repressor protein) and PrtN (an activator protein) co-regulate activity of many pyocin genes. As noted above, DNA-damaging agents increase production of RecA, which in turn, cleaves PrtR and releases PrtN [45]. Hence, since DNA repair-related genes including *recA *were upregulated in our study and regulatory genes such as *recA*, *prtN*, and *prtR *are probably shared by S-, F-, and R-type pyocins [41], we may conclude that DNA damage might also be a reason for the induction of these pyocins, S2, S3, S5, R2, and F2. Moreover, the induction of lysis enzymes (PA0614, PA0629, PA0630, and PA0631), which are likely shared by S-, F-, and R-type pyocins, indicates that these pyocins might have been released from the cells [41].

These results suggest that host cells are exposed to pyocins that are induced by oxidative stress during active infection. As indicated above, pyocins are known to kill species closely related to *P. aeruginosa *(e.g. some of Gram-negative bacteria). Interestingly, pyocins display cytotoxic effects on human cancer cells, triggering lethal events [46,47]. At present, it is unclear whether pyocins are causative toxic agents in patients with defective immune systems; however, it should not be excluded that pyocin production might be another defense mechanism that *P. aeruginosa *employs against oxidative attack by host cells.

Another notable finding was that PA1151 (*imm2*) encoding the pyocin S2 immunity protein exhibited a significant *decrease *in transcript level (Table 3). That is, secreted bacteriocins pose a survival problem to host bacteria, so cells produce immunity enzymes, high affinity inhibitors of bacteriocins, during bacteriocin synthesis [42]. *P. aeruginosa *possesses several pyocin immunity proteins that provide a self-protection function against S-type pyocins [26,40], by binding to the pyocin nuclease domain and neutralizing its activity [40,42]. Thus, our result that the pyocin S2 immunity protein was repressed during pyocin production could render *P. aeruginosa *vulnerable to this bacteriocin, which might even lead to its own death during pyocin synthesis.

Coincident with this, it was earlier observed that strains of *P. aeruginosa *produce pyocins active against themselves [48]. The conditions leading to their production were not investigated. The potential for programmed cell death in this system is an intriguing speculation. It may be that self-killing activity by part of a *P. aeruginosa *population benefits the remainder. For example, microbial population may dissipate damaging effects of extracellular stress such as pH change and oxidative stress by lysing some of the cells, which might be particularly effective for *P. aeruginosa *biofilms where layers of cells exist. Besides, given that DNA damage might elicit pyocin production, cells with damaged DNA may be the most appropriate targets for cell death, enabling preservation of the remainder of the population.

These kinds of activities apparently require cell-to-cell communication; therefore, it is interesting to question whether the quorum sensing system might be involved in pyocin synthesis. Based on quorum sensing-regulated genes that have been previously reported (163 genes from Hentzer *et al*. [49]; 315 genes from Schuster *et al*. [50]; 388 genes from Wagner *et al*. [51]), we found that 62, 69 and 56 genes, respectively, also showed transcription level changes in response to hydrogen peroxide in our study. While coincident up- or down-regulation does not establish a connection between quorum sensing and pyocin systems, this result might indicate that some genes involved in the quorum sensing system were also activated during cellular response to oxidants. In this light, it is noteworthy that degradation of secreted peptide *Staphylococcus aureus *autoinducers has been linked to the antioxidant response [14].

The last and perhaps most interesting result is that S-type pyocins become more lethal under iron-limited conditions because they presumably enter cells through siderophore receptors [52]; thus, the repression of siderophore receptors in our study (discussed above) may increase the possibility that the activation of S-type pyocins had larger impact on producer cells. However, in contrast, it might also be possible that the induction of pyocin production might contribute to the repression of iron uptake-related genes of the other population. Further investigation is required to verify the role of the immunity protein repression and the relationship between pyocins and iron-uptake genes.

### Co-transcription of genes in operons

In our attempt to further evaluate the transcription levels of genes discussed above, we investigated the co-transcription of their operons. That is, since genes within operons are largely co-regulated, we expected a considerable correlation between these genes and their neighboring genes within the same operon. To test this, we first selected 16 putative operons of the genes described in this report using the "Predicting operons in microbial genome" resource of the Institute for Genomic Research . We then calculated the Pearson's correlation coefficient (R) from the transcript levels of 55 pairs of neighboring genes in these 16 operons. For baseline comparison, we also analyzed 1000 pairs of randomly selected neighboring genes across the genome. Note that each pair has nine (four control and five experimental) array datasets. In Table 4, we observed that the neighboring genes sharing the same operon exhibited significantly high co-transcription levels (the mean and median R values) in this study. This result also provides a high degree of confidence in the transcriptome data we presented herein.

**Table 4:** Descriptive statistics for the co-transcription level of neighboring gene pairs

	Number of gene pairs^a^	Mean R ± standard error	Median R
Pairs within operons	55	0.7688 ± 0.0573	0.9449
Random pairs	1000	0.1984 ± 0.0138	0.2154

^a^Each gene pair has nine microarray datasets.

### Comparison of microarray data to other data in the literature

Lastly, in order to provide another assessment on our results, we compared our genes with statistically marked transcript level changes with those published in Palma *et al*. [12]. As indicated above, our study examined the transcriptional response upon 20 min exposure to 1 mM hydrogen peroxide in Luria-bertani broth, whereas Palma *et al*. [12] utilized 10 min exposure time in tryptic soy broth. Since the Mann-Whitney test with the cutoff *p *and fold change values of 0.05 and 2, respectively, was applied to both studies, we performed a comparative analysis between the two transcriptome profiles. Supplementary Table 2 shows that of 223 genes that we previously considered statistically marked in our report, 41 and 46 genes were upregulated and downregulated, respectively, in both studies. Further, 4 genes induced in this report were repressed in Palma *et al*. [12], while 11 genes displayed the opposite expression pattern. In Supplementary Table 2, 70 and 46 genes that were upregulated and downregulated, respectively, in this study were not among genes with statistically significant mRNA level changes in Palma's report. Interestingly, the fold changes were generally lower under our experimental conditions. This result demonstrates that about 40% of our statistically marked genes were regulated in the same direction in Palma *et al*. [12].

Next, we focused on genes that we earlier discussed in this report, which were originally listed in Table 3. Supplementary Table 3 shows that most genes in the categories of "primary metabolism-related" and "cellular protective mechanism-related" were concurrently regulated in the two reports. To be particular, genes encoding enzymes that play integral roles in oxidative response, such as catalases and DNA repair proteins, were consistently upregulated. This result reinforces our prior conclusion that the early transcriptional responses of *P. aeruginosa *to hydrogen peroxide include the repression of primary metabolism and the induction of protective mechanisms. On the other hand, most genes related to iron regulation and pyocin system were discordantly regulated in the two studies (Supplementary Table 3). As previously discussed, while we cannot fully elucidate this disagreement, we should not exclude the possibility that different growth mediums, strain backgrounds, and exposure times account for the divergent microarray data. Indeed, several reports are in line with this speculation: (i) Vasil proposed that dissimilar experimental conditions including strain history and growth mediums bring about discrepancies in the quorum-sensing transcriptional profiles [53], (ii) Spiegeleer *et al*. demonstrated that oxidative stress resistance of *E. coli *is dependent on the growth medium [54], (iii) Kang *et al*. suggested that strain background is one of the factors affecting a microarray-based transcriptome analysis [55], and (iv) our laboratory observed a large difference in the transcriptional profiles of *Staphylococcus aureus *response to oxidative stress between 10 min and 20 min exposures (unpublished data). Therefore, this result suggests that further investigation on iron regulation and pyocin system in *P. aeruginosa *under oxidative stress requires careful evaluation of transcriptional effects by experimental variances.

## Conclusion

In summary, we demonstrated how oxidative stress-induced genes were related and regulated in *P. aeruginosa *by utilizing whole-genome microarrays. Our results suggest that DNA repair proteins and catalases be among the most vital antioxidant defense systems of *P. aeruginosa *for preventing lethal effects of reactive oxygen intermediates. Second, a slowdown of membrane function-related genes was observed, implying that sublethal oxidative damage reduced active and/or facilitated transport through the cell membrane. Third, it was confirmed that oxidative stress could affect iron metabolism in that many of Fur-regulated genes were repressed upon exposure to hydrogen peroxide. In addition, this finding, together with other reports [5,32] suggests that intracellular iron level might be a key factor for the relationship. Most importantly, we provided evidence that reactive oxidant treatment results in the induction of all three types of pyocin genes in *P. aeruginosa*. Furthermore, it was demonstrated that a pyocin immunity protein was repressed, which if coincident events occur might lead to self-killing activity. To our knowledge, this is the first study reporting pyocin upregulation in response to oxidative stress. Hence, we are currently exploring whether the repression of an immunity protein increases the sensitivity of *P. aeruginosa *to pyocins and pyocin production benefits its population against toxic oxidants.

## Methods

### Bacterial strains and growth conditions

In this study, we used *Pseudomonas aeruginosa *PA01 obtained from Dr. E. Peter Greenberg's laboratory at the University of Iowa. To maintain homogeneous culture samples throughout our experiments, we employed the following three steps: (i) we initiated *P. aeruginosa *cultures at 37°C with shaking at 250 rpm using sterilized Luria-Bertani (LB) broth (10 g of tryptone, 5 g of yeast extract and 10 g of sodium chloride per liter), (ii) after 17 hours, we diluted the overnight cultures 1:100 in pre-warmed LB broth and incubated at 37°C with shaking at 250 rpm until an optical density at 600 nm (OD_600_) reached the early logarithmic phase (~ 0.8), and (iii) we re-diluted the cells 1:10 in pre-warmed LB broth and incubated at 37°C with shaking at 250 rpm [10,56]. Then, we added 1 mM hydrogen peroxide (Aldrich Chemical Co., St. Louis, MO) immediately after OD_600 _reached 0.8. Note that culture volumes for all growth conditions were adjusted to be less than 1/10 the total flask volume to maximize aeration.

### Affymetrix *P. aeruginosa *GeneChip arrays

Total RNA was isolated after 20 min incubation using RNeasy Mini kit (Qiagen, Inc., Valencia, CA) according to the manufacturer's protocol. As described previously [56], we added 2 mL of RNAprotect Bacteria Reagent (Qiagen, Inc., Valencia, CA) into 1 mL of the culture before the isolation to stabilize our RNA. RNA quality was determined using both Lambda 25 spectrophotometer (PerkinElmer, Inc., MA) and RNA 6000 Nano LabChip with an Agilent 2100 Bioanalyzer (Agilent Technologies, Palo Alto, CA). Next, we used 12 μg of total RNA with random primers and SuperScript II (both from Invitrogen Corp., Carlsbad, CA) for cDNA synthesis, cDNA fragmentation, labeling, hybridization, staining and washing steps were performed according to the manufacturer's protocol for the Affymetrix *P. aeruginosa *GeneChip arrays (Affymetrix, Inc., Santa Clara, CA). Finally, the arrays were scanned with the Affymetrix GeneChip Scanner 3000.

### Data analysis and real-time PCR

To analyze the array data, we utilized Affymetrix GeneChip Operating Software (GCOS) v. 1.0 and Data Mining Tool (DMT) v. 3.1 (Affymetrix, Inc., Santa Clara, CA) with the following parameters: *alpha *1, 0.04; *alpha *2, 0.06; *tau*, 0.015; target signal, 150. *Alpha *1 and 2 are significance levels that define detection calls (see below), while *tau *determines analysis sensitivity [15]. Further, the average intensity of arrays was scaled to a target signal. We calculated fold change as the ratio between the signal averages of four untreated (control) and five hydrogen peroxide-treated (experimental) cultures. Gene expression fold changes were identified with statistical significance by the Mann-Whitney test (cutoff *p*-value, 0.05). The GCOS detection calls of "Present", "Marginal", and "Absent" are determined based on the Affymetrix detection algorithm [15]. This call indicates whether a transcript is reliably detected (present) or not detected (absent). Genes that received absent calls from 50% or more of the replicates in GCOS were excluded from the final list.

Lastly, to determine the validity of the array data, transcript level changes obtained with the microarray analysis were compared with those from quantitative real-time PCR. Genes and primer sequences employed for the real-time PCR analysis are listed in Table 2. We performed the real-time PCR by utilizing iCycler iQ Real-Time PCR Detection System with iScript cDNA Synthesis Kit and One-Step RT-PCR Kit with SYBR Green (BioRad Laboratories, Inc., Hercules, CA). As stated above, RNA samples were treated with DNase I (Qiagen, Inc., Valencia, CA) to preclude DNA contamination, which was confirmed with Agilent 2100 Bioanalyzer LabChip and gel electrophoresis. In this report, relative quantification based on the relative expression of a target gene versus a reference gene was utilized to determine transcript level changes [57]. For each gene, five biological replicates with three technical replicates each were employed. PCR efficiencies were also derived from standard curve slopes in the iCycler software v. 3.1 (BioRad Laboratories, Inc., Hercules, CA). Finally, melt-curve analysis was performed to evaluate PCR specificity and resulted in single primer-specific melting temperatures.

## Authors' contributions

WC performed microarray experiments, and data analysis, and drafted the manuscript. DAS performed microarray experiments. FT initiated and supervised the study, and reviewed the manuscript. WEB supervised the study and reviewed the manuscript.

## Supplementary Material

Additional File 1 Probe set data (average signals, *p*-values, and fold changes) for experimental and control samples. Click here for file

Additional File 2 **Comparison of the fold changes and their directions of the significantly marked genes in this study with those in Palma *et al*. **[12]**(a total of 223 genes). **The Mann-Whitney test with the cutoff *p *and fold change values of 0.05 and 2, respectively, was applied to both studies, Click here for file

Additional File 3 **Comparison of the expression change directions of genes that we discussed in this study with those in Palma *et al*. **[12]. The genes were classified into the categories of "primary metabolism-related", "cellular protective mechanisms-related", "iron regulation-related", and "pyocin system-related", as presented in Table 3. Click here for file

## References

[B1] Miller RA, Britigan BE (1997). Role of oxidants in microbial pathophysiology. Clin Microbiol Rev.

[B2] Keyer K, Gort AS, Imlay JA (1995). Superoxide and the production of oxidative DNA damage. J Bacteriol.

[B3] McCormick ML, Buettner GR, Britigan BE (1998). Endogenous superoxide dismutase levels regulate iron-dependent hydroxyl radical formation in *Escherichia coli *exposed to hydrogen peroxide. J Bacteriol.

[B4] Keyer K, Imlay JA (1996). Superoxide accelerates DNA damage by elevating free-iron levels. Proc Natl Acad Sci.

[B5] Imlay JA (2003). Pathways of oxidative damage. Annu Rev Microbiol.

[B6] Brown SM, Howell ML, Vasil ML, Anderson AJ, Hassett DJ (1995). Cloning and characterization of the *katB *gene of *Pseudomonas aeruginosa *encoding a hydrogen peroxide-inducible catalase: purification of KatB, cellular localization, and demonstration that it is essential for optimal resistance to hydrogen peroxide. J Bacteriol.

[B7] Ochsner UA, Vasil ML, Alsabbagh E, Parvatiyar K, Hassett DJ (2000). Role of the *Pseudomonas aeruginosa oxyR-recG *operon in oxidative stress defense and DNA repair: OxyR-dependent regulation of *katB-ankB, ahpB*, and *ahpC-ahpF*. J Bacteriol.

[B8] Goodman AL, Lory S (2004). Analysis of regulatory networks in *Pseudomonas aeruginosa *by genomewide transcriptional profiling. Curr Opin Microbiol.

[B9] Talaat A, Lyons R, Howard S, Johnston S (2004). The temporal expression profile of *Mycobacterium tuberculosis *infection in mice. Proc Natl Acad Sci.

[B10] Ma JF, Ochsner UA, Klotz MG, Nanayakkara VK, Howell ML, Johnson Z, Posey JE, Vasil ML, Monaco JJ, Hassett DJ (1999). Bacterioferritin A modulates catalase A (KatA) activity and resistance to hydrogen peroxide in *Pseudomonas aeruginosa*. J Bacteriol.

[B11] Hassett DJ, Alsabbagh E, Parvatiyar K, Howell ML, Wilmott RW, Ochsner UA (2000). A protease-resistant catalase, KatA, released upon cell lysis during stationary phase is essential for aerobic survival of a *Pseudomonas aeruginosa oxyR *mutant at low cell densities. J Bacteriol.

[B12] Palma M, DeLuca D, Worgall S, Quadri LE (2004). Transcriptome analysis of the response of *Pseudomonas aeruginosa *to hydrogen peroxide. J Bacteriol.

[B13] Wu CL, Domenico P, Hassett DJ, Beveridge TJ, Hauser AR, Kazzaz JA (2002). Subinhibitory bismuth-thiols reduce virulence of *Pseudomonas aeruginosa*. Am J Respir Cell Mol Biol.

[B14] Rothfork JM, Timmins GS, Harris MN, Chen X, Lusis AJ, Otto M, Cheung AL, Gresham HD (2004). Inactivation of a bacterial virulence pheromone by phagocyte-derived oxidants: new role for the NADPH oxidase in host defense. Proc Natl Acad Sci.

[B15] Affymetrix GeneChip^® ^expression analysis technical manual. http://www.affymetrix.com/support/technical/manual/expression_manual.affx.

[B16] Zheng M, Wang X, Templeton LJ, Smulski DR, LaRossa RA, Storz G (2001). DNA microarray-mediated transcriptional profiling of the *Escherichia coli *response to hydrogen peroxide. J Bacteriol.

[B17] Gene Expression Omnibus (GEO). http://www.ncbi.nlm.nih.gov/geo.

[B18] Savli H, Karadenizli A, Kolayli F, Gundes S, Ozbek U, Vahaboglu H (2003). Expression stability of six housekeeping genes: a proposal for resistance gene quantification studies of *Pseudomonas aeruginosa *by real-time quantitative RT-PCR. J Med Microbiol.

[B19] The *Pseudomonas aeruginosa *Community Annotation Project. http://www.pseudomonas.com.

[B20] Freudl R, Braun G, Honore N, Cole ST (1987). Evolution of the enterobacterial *sulA *gene: a component of the SOS system encoding an inhibitor of cell division. Gene.

[B21] Elkins JG, Hassett DJ, Stewart PS, Schweizer HP, McDermott TR (1999). Protective role of catalase in *Pseudomonas aeruginosa *biofilm resistance to hydrogen peroxide. Appl Environ Microbiol.

[B22] Hassett DJ, Schweizer HP, Ohman DE (1995). *Pseudomonas aeruginosa sodA *and *sodB *mutants defective in manganese- and iron-cofactored superoxide dismutase activity demonstrate the importance of the iron-cofactored form in aerobic metabolism. J Bacteriol.

[B23] Martinez A, Kolter R (1997). Protection of DNA during oxidative stress by the nonspecific DNA-binding protein Dps. J Bacteriol.

[B24] Little JW (1991). Mechanism of specific LexA cleavage: autodigestion and the role of RecA coprotease. Biochimie.

[B25] Huisman O, D'Ari R, Gottesman S (1984). Cell-division control in *Escherichia coli*: specific induction of the SOS function SfiA protein is sufficient to block septation. Proc Natl Acad Sci.

[B26] Sano Y (1993). Role of the *recA*-related gene adjacent to the *recA *gene in *Pseudomonas aeruginosa*. J Bacteriol.

[B27] Papavinasasundaram KG, Colston MJ, Davis EO (1998). Construction and complementation of a *recA *deletion mutant of *Mycobacterium smegmatis *reveals that the intein in *Mycobacterium tuberculosis recA *does not affect RecA function. Mol Microbiol.

[B28] Lomba MR, Vasconcelos AT, Pacheco AB, de Almeida DF (1997). Identification of *yebG *as a DNA damage-inducible *Escherichia coli *gene. FEMS Microbiol Lett.

[B29] Oh TJ, Kim IG (1999). Identification of genetic factors altering the SOS induction of DNA damage-inducible *yebG *gene in *Escherichia coli*. FEMS Microbiol Lett.

[B30] Dervyn E, Suski C, Daniel R, Bruand C, Chapuis J, Errington J, Janniere L, Ehrlich SD (2001). Two essential DNA polymerases at the bacterial replication fork. Science.

[B31] Le Chatelier E, Becherel OJ, d'Alencon E, Canceill D, Ehrlich SD, Fuchs RP, Janniere L (2004). Involvement of DnaE, the second replicative DNA polymerase from *Bacillus subtilis*, in DNA mutagenesis. J Biol Chem.

[B32] Zheng M, Doan B, Schneider TD, Storz G (1999). OxyR and SoxRS regulation of *fur*. J Bacteriol.

[B33] Visca P, Leoni L, Wilson MJ, Lamont IL (2002). Iron transport and regulation, cell signaling and genomics: lessons from *Escherichia coli *and *Pseudomonas*. Mol Microbiol.

[B34] Palma M, Worgall S, Quadri LE (2003). Transcriptome analysis of the *Pseudomonas aeruginosa *response to iron. Arch Microbiol.

[B35] Reimmann C, Serino L, Beyeler M, Haas D (1998). Dihydroaeruginoic acid synthetase and pyochelin synthetase, products of the *pchEF *genes, are induced by extracellular pyochelin in *Pseudomonas aeruginosa*. Microbiology.

[B36] Vasil ML, Ochsner UA (1999). The response of *Pseudomonas aeruginosa *to iron: genetics, biochemistry and virulence. Mol Microbiol.

[B37] Poole K, Neshat S, Krebes K, Heinrichs DE (1993). Cloning and nucleotide sequence analysis of the ferripyoverdine receptor gene *fpvA *of *Pseudomonas aeruginosa*. J Bacteriol.

[B38] Heinrichs DE, Poole K (1996). PchR, a regulator of ferripyochelin receptor gene (*fptA*) expression in *Pseudomonas aeruginosa*, functions both as an activator and as a repressor. J Bacteriol.

[B39] Serino L, Reimmann C, Visca P, Beyeler M, Chiesa VD, Haas D (1997). Biosynthesis of pyochelin and dihydroaeruginoic acid requires the iron-regulated *pchDCBA *operon in *Pseudomonas aeruginosa*. J Bacteriol.

[B40] Michel-Briand Y, Baysse C (2002). The pyocins of *Pseudomonas aeruginosa*. Biochimie.

[B41] Nakayama K, Takashima K, Ishihara H, Shinomiya T, Kageyama M, Kanaya S, Ohnishi M, Murata T, Mori H, Hayashi T (2000). The R-type pyocin of *Pseudomonas aeruginosa *is related to P2 phage, and the F-type is related to lambda phage. Mol Microbiol.

[B42] Kleanthous C, Walker D (2001). Immunity proteins: enzyme inhibitors that avoid the active site. Trends Biochem Sci.

[B43] de Zamaroczy M, Buckingham RH (2002). Importation of nuclease colicins into *E. coli *cells: endoproteolytic cleavage and its prevention by the immunity protein. Biochimie.

[B44] Sano Y, Matsui H, Kobayashi M, Kageyama M (1993). Molecular structures and functions of pyocins S1 and S2 in *Pseudomonas aeruginosa*. J Bacteriol.

[B45] Matsui H, Sano Y, Ishihara H, Shinomiya T (1993). Regulation of pyocin genes in *Pseudomonas aeruginosa *by positive (*prtN*) and negative (*prtR*) regulatory genes. J Bacteriol.

[B46] Farkas-Himsley H, Hill R, Rosen B, Arab S, Lingwood CA (1995). The bacterial colicin active against tumor cells in vitro and in vivo is verotoxin 1. Proc Natl Acad Sci.

[B47] Abdi-Ali A, Worobec EA, Deezagi A, Malekzadeh F (2004). Cytotoxic effects of pyocin S2 produced by *Pseudomonas aeruginosa *on the growth of three human cell lines. Can J Microbiol.

[B48] Goodwin K, Levin RE, Doggett RG (1972). Autosensitivity of *Pseudomonas aeruginosa *to its own pyocin. Infect Immun.

[B49] Hentzer M, Wu H, Andersen J, Riedel K, Rasmussen T, Bagge N, Kumar N, Schembri M, Song Z, Kristoffersen P, Manefield M, Costerton J, Molin S, Eberl L, Steinberg P, Kjelleberg S, Hoiby N, Givskov M (2003). Attenuation of *Pseudomonas aeruginosa *virulence by quorum sensing inhibitors. EMBO J.

[B50] Schuster M, Lostroh C, Ogi T, Greenberg E (2003). Identification, timing, and signal specificity of *Pseudomonas aeruginosa *quorum-controlled genes: a transcriptome analysis. J Bacteriol.

[B51] Wagner V, Bushnell D, Passador L, Brooks A, Iglewski B (2003). Microarray analysis of *Pseudomonas aeruginosa *quorum-sensing regulons: effects of growth phase and environment. J Bacteriol.

[B52] Baysse C, Meyer JM, Plesiat P, Geoffroy V, Michel-Briand Y, Cornelis P (1999). Uptake of pyocin S3 occurs through the outer membrane ferripyoverdine type II receptor of *Pseudomonas aeruginosa*. J Bacteriol.

[B53] Vasil ML (2003). DNA microarrays in analysis of quorum sensing: strengths and limitations. J Bacteriol.

[B54] de Spiegeleer P, Sermon J, Lietaert A, Aertsen A, Michiels CW (2004). Source of tryptone in growth medium affects oxidative stress resistance in *Escherichia coli*. J Appl Microbiol.

[B55] Kang Y, Weber KD, Qiu Y, Kiley PJ, Blattner FR (2005). Genome-wide expression analysis indicates that FNR of *Escherichia coli *K-12 regulates a large number of genes of unknown function. J Bacteriol.

[B56] Chang W, Small DA, Toghrol F, Bentley WE (2005). Microarray analysis of toxicogenomic effects of peracetic acid on *Pseudomonas aeruginosa*. Environ Sci Technol.

[B57] Pfaffl MW (2001). A new mathematical model for relative quantification in real-time RT-PCR. Nucleic Acids Res.

