# Supplementary material for: Microarray analysis of Pseudomonas aeruginosa reveals induction of pyocin genes in response to hydrogen peroxide
Source: BMC Genomics. 2005 Sep 8;6:115. doi: 10.1186/1471-2164-6-115 (PMC1250226; doi:10.1186/1471-2164-6-115)
Supplement: Additional File 1 — Probe set data (average signals, p-values, and fold changes) for experimental and control samples. [file 1471-2164-6-115-S1.pdf]

Supplementary Table 1. Probe set signal data for experimental and control samples

Affymetrix probe set name	Average signal of experimentals	Average signal of controls	*p*-value	Fold change*
PA0001_dnaA_at	516.62	499.28	0.238	1.03
PA0002_dnaN_at	1337.60	1278.28	0.817	1.05
PA0003_recF_at	364.18	430.35	0.048	-1.18
PA0004_gyrB_at	349.56	461.25	0.024	-1.32
PA0005_at	115.16	146.85	0.024	-1.28
PA0006_at	68.08	92.10	0.008	-1.35
PA0007_at	5.30	23.38	0.008	-4.41
PA0008_glyS_at	346.64	430.80	0.087	-1.24
PA0009_glyQ_at	586.04	675.40	0.238	-1.15
PA0010_tag_at	29.24	25.47	0.817	1.15
PA0011_at	78.84	110.55	0.008	-1.40
PA0012_at	44.20	53.90	0.048	-1.22
PA0013_at	14.52	33.00	0.008	-2.27
PA0014_at	13.04	13.35	0.817	-1.02
PA0015_at	46.68	64.45	0.048	-1.38
PA0016_trkA_at	101.30	180.60	0.008	-1.78
PA0017_at	141.50	214.80	0.087	-1.52
PA0018_fmt_at	256.22	325.40	0.087	-1.27
PA0019_def_at	575.64	745.38	0.008	-1.29
PA0020_at	369.02	335.20	0.151	1.10
PA0021_at	8.14	8.98	0.484	-1.10
PA0022_at	33.12	48.97	0.008	-1.48
PA0023_qor_at	92.56	79.77	0.238	1.16
PA0024_hemF_at	190.28	254.68	0.048	-1.34
PA0025_aroE_at	59.64	58.55	1.000	1.02
PA0026_at	252.96	193.48	0.087	1.31
PA0027_at	65.14	47.40	0.087	1.37
PA0028_at	55.00	40.47	0.238	1.36
PA0029_at	9.90	23.72	0.151	-2.40
PA0030_at	14.78	16.45	0.349	-1.11
PA0031_betC_at	9.46	8.55	0.817	1.11
PA0032_at	38.90	42.50	0.643	-1.09
PA0033_at	29.50	31.98	0.484	-1.08
PA0034_at	46.10	55.35	0.238	-1.20
PA0035_trpA_at	99.16	122.65	0.087	-1.24
PA0036_trpB_at	199.56	192.08	0.643	1.04
PA0037_trpI_at	27.90	36.45	0.151	-1.31
PA0038_at	46.62	60.95	0.151	-1.31
PA0039_at	283.56	435.22	0.048	-1.53
PA0040_s_at	208.78	214.35	0.643	-1.03
PA0041_at	27.98	45.55	0.048	-1.63
PA0042_at	88.92	124.25	0.048	-1.40
PA0043_at	15.70	27.30	0.008	-1.74
PA0044_exoT_at	82.96	75.55	0.238	1.10
PA0045_at	973.22	1156.63	0.024	-1.19
PA0046_at	669.38	917.28	0.024	-1.37

Supplementary Table 1. Probe set signal data for experimental and control samples

PA0047_at	230.96	311.42	0.024	-1.35
PA0048_at	31.00	37.00	0.484	-1.19
PA0049_at	182.86	113.55	0.008	1.61
PA0050_r_at	52.54	67.70	0.238	-1.29
PA0051_at	17.74	21.77	0.643	-1.23
PA0052_at	18.54	11.98	0.484	1.55
PA0053_at	40.14	40.33	0.730	-1.00
PA0054_at	52.16	52.30	0.643	-1.00
PA0055_at	525.16	449.48	0.024	1.17
PA0056_at	22.60	20.92	0.484	1.08
PA0057_at	36.02	33.78	0.643	1.07
PA0058_at	14.40	24.88	0.087	-1.73
PA0059_osmC_at	27.98	21.02	0.087	1.33
PA0060_at	159.12	177.25	0.484	-1.11
PA0061_at	8.80	6.42	0.643	1.37
PA0062_at	19.06	18.65	1.000	1.02
PA0063_at	52.04	43.78	0.238	1.19
PA0064_at	92.68	105.10	0.087	-1.13
PA0065_at	225.44	272.15	0.008	-1.21
PA0066_at	223.02	239.13	0.349	-1.07
PA0067_prlC_at	1204.98	867.25	0.008	1.39
PA0068_at	350.44	318.42	0.349	1.10
PA0069_at	45.14	17.60	0.008	2.56
PA0070_at	2113.70	1860.70	0.238	1.14
PA0071_at	96.40	129.58	0.151	-1.34
PA0072_at	50.42	64.35	0.087	-1.28
PA0073_at	169.40	197.35	0.238	-1.16
PA0074_ppkA_at	186.36	235.07	0.087	-1.26
PA0075_at	322.20	450.83	0.048	-1.40
PA0076_at	156.16	157.95	0.817	-1.01
PA0077_at	321.08	345.10	0.349	-1.07
PA0078_at	358.28	393.55	0.349	-1.10
PA0079_at	419.46	376.70	0.349	1.11
PA0080_at	115.84	182.65	0.024	-1.58
PA0081_at	409.10	395.10	0.643	1.04
PA0082_at	178.76	278.58	0.008	-1.56
PA0083_at	1486.66	1763.73	0.151	-1.19
PA0084_at	962.20	1282.50	0.024	-1.33
PA0085_at	1234.78	1551.17	0.238	-1.26
PA0086_at	611.84	622.13	1.000	-1.02
PA0087_at	285.88	316.40	0.643	-1.11
PA0088_at	166.14	216.47	0.087	-1.30
PA0089_at	189.04	239.55	0.151	-1.27
PA0090_at	654.06	668.85	1.000	-1.02
PA0091_at	126.42	174.55	0.151	-1.38
PA0092_at	73.58	117.50	0.008	-1.60
PA0093_at	297.36	227.45	0.048	1.31
PA0094_at	537.82	486.48	0.151	1.11
PA0095_at	148.04	129.13	0.238	1.15
PA0096_at	66.34	59.65	0.484	1.11
PA0097_at	79.30	77.35	1.000	1.03

Supplementary Table 1. Probe set signal data for experimental and control samples

PA0098_at	58.54	43.18	0.024	1.36
PA0099_at	65.18	55.60	0.484	1.17
PA0100_at	172.58	174.03	1.000	-1.01
PA0101_at	127.74	116.70	1.000	1.09
PA0102_at	391.64	415.50	0.643	-1.06
PA0103_at	55.54	65.05	0.238	-1.17
PA0104_at	15.18	16.27	0.643	-1.07
PA0105_coxB_at	17.18	9.40	0.087	1.83
PA0106_coxA_at	15.00	13.75	0.643	1.09
PA0107_at	14.94	11.35	0.484	1.32
PA0108_coIII_at	21.98	14.98	0.238	1.47
PA0109_at	8.40	6.78	0.817	1.24
PA0110_at	29.54	36.08	0.175	-1.22
PA0111_at	9.74	12.52	0.238	-1.29
PA0112_at	30.86	28.82	0.643	1.07
PA0113_at	40.36	36.75	0.817	1.10
PA0114_at	153.04	166.32	0.349	-1.09
PA0115_at	90.34	88.57	0.643	1.02
PA0116_at	48.16	60.58	0.087	-1.26
PA0117_at	74.76	72.95	0.817	1.02
PA0118_at	31.52	31.18	0.817	1.01
PA0119_at	34.04	27.38	0.643	1.24
PA0120_at	38.78	39.15	1.000	-1.01
PA0121_at	40.14	44.13	0.349	-1.10
PA0122_at	84.56	74.00	0.349	1.14
PA0123_at	58.38	52.25	0.087	1.12
PA0124_at	131.68	144.43	0.151	-1.10
PA0125_at	108.76	144.05	0.048	-1.32
PA0126_at	565.20	708.40	0.048	-1.25
PA0127_at	29.18	31.83	0.817	-1.09
PA0128_at	43.12	97.80	0.008	-2.27
PA0129_gabP_at	108.38	82.25	0.151	1.32
PA0130_at	223.60	158.93	0.008	1.41
PA0131_at	91.00	133.80	0.151	-1.47
PA0132_at	262.30	302.65	0.238	-1.15
PA0133_at	61.68	47.75	0.048	1.29
PA0134_at	31.02	30.95	0.817	1.00
PA0135_at	10.08	11.23	0.817	-1.11
PA0136_at	3.56	11.43	0.024	-3.21
PA0137_at	10.84	17.83	0.151	-1.64
PA0138_at	8.24	8.30	1.000	-1.01
PA0139_ahpC_at	2937.08	2478.05	0.238	1.19
PA0140_ahpF_at	126.76	162.30	0.087	-1.28
PA0141_at	110.68	98.50	0.238	1.12
PA0142_at	44.14	31.07	0.151	1.42
PA0143_at	111.68	177.93	0.008	-1.59
PA0144_at	12.52	27.00	0.024	-2.16
PA0145_i_at	7.70	18.35	0.024	-2.38
PA0146_at	17.90	30.28	0.151	-1.69
PA0147_at	57.34	38.42	0.008	1.49
PA0148_at	118.10	142.48	0.048	-1.21

Supplementary Table 1. Probe set signal data for experimental and control samples

PA0149_at	28.40	20.90	0.087	1.36
PA0150_at	46.14	40.90	0.238	1.13
PA0151_at	22.66	31.40	0.024	-1.39
PA0152_pcaQ_at	31.88	38.97	0.087	-1.22
PA0153_pcaH_at	41.50	47.45	0.484	-1.14
PA0154_pcaG_at	60.14	45.05	0.087	1.33
PA0155_pcaR_at	154.14	159.95	0.484	-1.04
PA0156_at	244.32	205.88	0.048	1.19
PA0157_at	106.78	108.68	1.000	-1.02
PA0158_at	47.76	78.95	0.008	-1.65
PA0159_at	73.70	83.55	0.349	-1.13
PA0160_at	47.26	91.85	0.008	-1.94
PA0161_at	21.42	36.90	0.008	-1.72
PA0162_at	128.78	278.10	0.008	-2.16
PA0163_at	28.86	24.17	0.484	1.19
PA0164_at	24.44	21.50	0.817	1.14
PA0165_at	608.40	784.38	0.008	-1.29
PA0166_at	2.68	10.85	0.008	-4.05
PA0167_at	271.22	187.98	0.048	1.44
PA0168_at	112.48	58.00	0.008	1.94
PA0169_at	55.60	94.08	0.024	-1.69
PA0170_at	109.48	169.83	0.024	-1.55
PA0171_at	223.64	396.85	0.008	-1.77
PA0172_at	101.14	113.28	0.349	-1.12
PA0173_at	11.82	7.73	0.349	1.53
PA0174_at	23.60	25.35	0.817	-1.07
PA0175_at	24.96	27.40	0.484	-1.10
PA0176_at	73.78	60.22	0.008	1.23
PA0177_at	10.88	15.63	0.087	-1.44
PA0178_at	36.78	30.33	0.151	1.21
PA0179_at	113.64	93.47	0.008	1.22
PA0180_at	61.40	65.15	0.643	-1.06
PA0181_at	54.50	50.63	0.643	1.08
PA0182_at	181.04	26.83	0.024	6.75
PA0183_atsA_at	46.70	38.80	0.643	1.20
PA0184_at	10.28	8.52	0.643	1.21
PA0185_at	30.64	27.10	0.349	1.13
PA0186_at	3.14	13.82	0.024	-4.40
PA0187_at	10.40	16.67	0.238	-1.60
PA0188_at	22.16	15.88	0.349	1.40
PA0189_at	31.46	18.17	0.008	1.73
PA0190_at	7.36	6.37	0.643	1.16
PA0191_at	13.54	15.50	0.643	-1.14
PA0192_at	17.00	21.23	0.349	-1.25
PA0193_at	30.74	31.80	0.556	-1.03
PA0194_at	30.34	39.15	0.151	-1.29
PA0195_pntA_at	68.94	74.72	0.484	-1.08
PA0196_pntB_at	24.68	29.65	0.238	-1.20
PA0197_at	32.86	23.80	0.048	1.38
PA0198_exbB1_at	6.88	4.13	0.643	1.67
PA0199_exbD1_at	10.80	25.00	0.024	-2.31

Supplementary Table 1. Probe set signal data for experimental and control samples

PA0200_i_at	23.62	33.85	0.349	-1.43
PA0201_at	52.12	71.38	0.413	-1.37
PA0202_at	20.16	18.95	0.817	1.06
PA0203_at	14.50	10.75	0.151	1.35
PA0204_at	13.16	12.95	0.817	1.02
PA0205_at	26.00	28.20	0.413	-1.08
PA0206_at	34.32	27.97	0.349	1.23
PA0207_at	60.10	48.03	0.087	1.25
PA0208_mdcA_at	15.20	13.25	1.000	1.15
PA0209_at	19.88	17.83	0.817	1.11
PA0210_mdcC_at	22.02	14.35	0.024	1.53
PA0211_mdcD_at	10.04	18.67	0.008	-1.86
PA0212_mdcE_at	5.48	15.35	0.048	-2.80
PA0213_at	12.08	8.40	0.151	1.44
PA0214_at	7.70	17.55	0.048	-2.28
PA0215_at	27.00	21.10	0.238	1.28
PA0216_at	6.78	11.07	0.349	-1.63
PA0217_at	18.16	30.10	0.008	-1.66
PA0218_at	29.58	30.63	0.817	-1.04
PA0219_at	19.68	22.10	0.817	-1.12
PA0220_at	21.96	27.25	0.349	-1.24
PA0221_at	19.78	18.40	0.413	1.08
PA0222_at	3.20	5.43	0.238	-1.70
PA0223_at	25.60	30.85	0.048	-1.21
PA0224_at	36.36	40.15	0.349	-1.10
PA0225_at	46.48	75.25	0.008	-1.62
PA0226_at	21.42	26.33	0.349	-1.23
PA0227_at	30.58	36.13	0.151	-1.18
PA0228_pcaF_at	16.60	17.05	0.817	-1.03
PA0229_pcaT_at	13.08	15.72	0.484	-1.20
PA0230_pcaB_at	92.92	67.22	0.048	1.38
PA0231_pcaD_at	97.10	69.27	0.008	1.40
PA0232_pcaC_at	40.80	38.73	0.484	1.05
PA0233_at	57.68	33.60	0.024	1.72
PA0234_at	15.54	16.55	0.349	-1.06
PA0235_pcaK_at	35.20	34.03	0.484	1.03
PA0236_at	85.34	88.30	0.643	-1.03
PA0237_at	18.76	28.80	0.143	-1.54
PA0238_at	21.48	22.75	0.817	-1.06
PA0239_at	36.72	34.45	0.643	1.07
PA0240_at	5.24	13.42	0.048	-2.56
PA0241_at	35.40	33.08	0.484	1.07
PA0242_at	17.42	20.85	0.238	-1.20
PA0243_at	59.66	51.20	0.817	1.17
PA0244_at	20.82	22.17	1.000	-1.06
PA0245_aroQ2_at	24.12	31.08	0.286	-1.29
PA0246_at	41.62	41.95	1.000	-1.01
PA0247_pobA_at	28.38	23.10	0.349	1.23
PA0248_at	38.36	30.33	0.151	1.26
PA0249_at	29.40	31.58	0.817	-1.07
PA0250_at	35.24	45.43	0.238	-1.29

Supplementary Table 1. Probe set signal data for experimental and control samples

PA0251_at	10.60	7.90	0.349	1.34
PA0252_at	24.18	27.40	0.349	-1.13
PA0253_at	43.38	57.35	0.048	-1.32
PA0254_at	31.18	38.20	0.238	-1.23
PA0255_at	54.38	48.73	0.643	1.12
PA0256_at	20.36	9.98	0.024	2.04
PA0257_at	19.18	15.63	0.349	1.23
PA0258_at	25.24	28.10	0.484	-1.11
PA0259_at	102.08	88.18	0.151	1.16
PA0260_at	146.78	136.23	0.349	1.08
PA0261_at	79.16	73.10	0.238	1.08
PA0262_at	22.50	33.63	0.024	-1.49
PA0263_hcpC_s_at	83.90	122.20	0.151	-1.46
PA0264_at	12.68	16.80	0.643	-1.32
PA0265_gabD_at	710.50	616.82	0.087	1.15
PA0266_gabT_at	1059.68	626.83	0.008	1.69
PA0267_at	39.38	57.28	0.008	-1.45
PA0268_at	22.46	20.48	0.921	1.10
PA0269_at	85.98	70.88	0.151	1.21
PA0270_at	84.50	53.13	0.008	1.59
PA0271_at	60.82	43.52	0.024	1.40
PA0272_at	41.64	41.40	0.817	1.01
PA0273_at	13.04	7.80	0.349	1.67
PA0274_at	17.74	16.48	0.817	1.08
PA0275_at	60.34	43.53	0.151	1.39
PA0276_at	10.54	8.67	0.817	1.22
PA0277_at	837.66	992.20	0.643	-1.18
PA0278_at	22.84	17.38	0.484	1.31
PA0279_at	33.82	25.10	0.151	1.35
PA0280_cysA_at	29.82	86.92	0.024	-2.91
PA0281_cysW_at	54.02	151.23	0.008	-2.80
PA0282_cysT_at	60.56	148.45	0.008	-2.45
PA0283_sbp_at	105.10	213.42	0.008	-2.03
PA0284_at	292.34	657.47	0.048	-2.25
PA0285_at	113.10	117.47	0.643	-1.04
PA0286_at	217.80	205.53	0.151	1.06
PA0287_at	4.26	6.43	0.484	-1.51
PA0288_speB1_at	16.64	10.25	0.238	1.62
PA0289_at	46.06	34.95	0.024	1.32
PA0290_at	23.22	20.38	1.000	1.14
PA0291_oprE_at	1385.40	1142.18	0.024	1.21
PA0292_at	383.24	357.45	0.484	1.07
PA0293_at	82.74	142.92	0.008	-1.73
PA0294_at	72.26	66.68	0.349	1.08
PA0295_at	86.82	61.88	0.048	1.40
PA0296_s_at	453.72	326.70	0.048	1.39
PA0297_at	46.14	43.10	0.817	1.07
PA0298_at	216.56	187.27	0.238	1.16
PA0299_at	805.18	536.53	0.008	1.50
PA0300_potF2_at	597.60	564.63	0.349	1.06
PA0301_potF3_at	262.76	271.58	0.643	-1.03

Supplementary Table 1. Probe set signal data for experimental and control samples

PA0302_potG_at	423.12	316.17	0.048	1.34
PA0303_potH_at	403.22	312.65	0.008	1.29
PA0304_potI_at	46.52	68.90	0.024	-1.48
PA0305_at	75.00	56.10	0.087	1.34
PA0306_at	31.74	25.00	0.024	1.27
PA0307_at	36.46	37.40	0.484	-1.03
PA0308_at	113.54	83.18	0.024	1.36
PA0309_at	97.02	113.18	0.349	-1.17
PA0310_at	51.30	60.30	0.151	-1.18
PA0311_at	22.72	37.45	0.087	-1.65
PA0312_at	123.86	81.73	0.008	1.52
PA0313_at	50.02	58.72	0.087	-1.17
PA0314_at	167.20	128.92	0.024	1.30
PA0315_at	374.88	259.17	0.048	1.45
PA0316_serA_at	425.04	594.08	0.024	-1.40
PA0317_at	507.52	461.78	0.048	1.10
PA0318_at	249.22	227.48	0.349	1.10
PA0319_at	189.72	194.38	0.643	-1.02
PA0320_at	34.04	74.35	0.008	-2.18
PA0321_at	20.12	9.25	0.008	2.18
PA0322_at	9.76	9.53	1.000	1.02
PA0323_at	4.18	6.03	0.238	-1.44
PA0324_at	5.40	8.35	0.238	-1.55
PA0325_at	23.32	19.92	0.556	1.17
PA0326_at	32.06	29.35	0.484	1.09
PA0327_at	8.94	9.40	0.484	-1.05
PA0328_at	120.52	99.05	0.087	1.22
PA0329_at	117.58	120.42	0.817	-1.02
PA0330_rpiA_at	302.12	367.80	0.048	-1.22
PA0331_ilvA1_at	223.58	263.60	0.087	-1.18
PA0332_at	101.38	100.18	0.817	1.01
PA0333_at	63.52	64.58	0.643	-1.02
PA0334_at	44.30	44.10	0.643	1.00
PA0335_at	428.52	217.65	0.008	1.97
PA0336_at	819.72	747.27	0.643	1.10
PA0337_ptsP_at	310.54	237.00	0.008	1.31
PA0338_at	19.52	27.47	0.087	-1.41
PA0339_at	41.40	34.05	0.238	1.22
PA0340_at	110.70	113.77	0.643	-1.03
PA0341_lgt_at	82.46	111.55	0.008	-1.35
PA0342_thyA_at	498.66	502.85	0.817	-1.01
PA0343_at	19.16	25.58	0.087	-1.34
PA0344_at	50.42	46.35	0.349	1.09
PA0345_at	112.90	83.57	0.008	1.35
PA0346_at	39.76	29.67	0.087	1.34
PA0347_glpQ_at	31.98	31.70	0.643	1.01
PA0348_at	21.56	22.88	1.000	-1.06
PA0349_at	9.32	10.03	0.643	-1.08
PA0350_folA_at	129.10	105.30	0.008	1.23
PA0351_at	96.32	84.80	0.190	1.14
PA0352_at	69.84	149.63	0.008	-2.14

Supplementary Table 1. Probe set signal data for experimental and control samples

PA0353_ilvD_at	167.44	154.97	0.817	1.08
PA0354_at	177.06	231.02	0.024	-1.30
PA0355_pfpI_at	33.44	27.63	0.151	1.21
PA0356_at	76.50	120.65	0.008	-1.58
PA0357_mutM_at	129.70	106.92	0.151	1.21
PA0358_at	122.58	125.47	0.643	-1.02
PA0359_at	202.38	206.05	0.484	-1.02
PA0360_at	77.90	66.88	0.349	1.16
PA0361_at	79.48	61.40	0.024	1.29
PA0362_fdx1_at	183.82	290.55	0.008	-1.58
PA0363_coaD_at	237.86	339.42	0.008	-1.43
PA0364_at	17.92	12.57	0.286	1.43
PA0365_at	65.82	55.50	0.349	1.19
PA0366_at	50.86	63.20	0.048	-1.24
PA0367_at	40.20	48.00	0.151	-1.19
PA0368_at	57.42	49.97	0.151	1.15
PA0369_at	60.94	85.20	0.024	-1.40
PA0370_at	257.16	260.47	0.817	-1.01
PA0371_at	223.60	228.55	0.484	-1.02
PA0372_at	392.10	435.10	0.151	-1.11
PA0373_ftsY_at	329.74	346.40	0.087	-1.05
PA0374_ftsE_at	351.52	371.35	0.817	-1.06
PA0375_ftsX_at	282.84	312.83	0.349	-1.11
PA0376_rpoH_at	1061.68	955.80	0.238	1.11
PA0377_at	90.76	92.97	0.905	-1.02
PA0378_at	51.56	40.40	0.151	1.28
PA0379_at	102.38	121.20	0.413	-1.18
PA0380_i_at	385.98	499.10	0.008	-1.29
PA0381_thiG_at	340.16	491.43	0.008	-1.44
PA0382_micA_at	134.78	221.97	0.008	-1.65
PA0383_at	17.00	18.18	0.817	-1.07
PA0384_at	37.10	48.97	0.008	-1.32
PA0385_at	44.84	95.57	0.008	-2.13
PA0386_at	149.04	144.73	0.349	1.03
PA0387_at	374.82	367.08	0.817	1.02
PA0388_at	167.86	264.83	0.008	-1.58
PA0389_at	157.12	203.10	0.008	-1.29
PA0390_metX_at	300.74	384.28	0.008	-1.28
PA0391_at	120.40	102.60	0.087	1.17
PA0392_at	388.52	384.08	0.643	1.01
PA0393_proC_at	456.68	350.70	0.024	1.30
PA0394_at	477.90	465.17	0.643	1.03
PA0395_pilT_at	262.18	202.80	0.008	1.29
PA0396_pilU_at	155.24	146.42	0.238	1.06
PA0397_at	21.08	22.33	0.730	-1.06
PA0398_at	58.24	71.45	0.087	-1.23
PA0399_at	349.54	337.33	0.349	1.04
PA0400_at	229.68	260.27	0.349	-1.13
PA0401_at	159.94	174.07	0.151	-1.09
PA0402_pyrB_at	442.96	394.85	0.238	1.12
PA0403_pyrR_at	200.56	221.75	0.238	-1.11

Supplementary Table 1. Probe set signal data for experimental and control samples

PA0404_i_at	386.02	281.42	0.008	1.37
PA0405_at	372.60	272.20	0.008	1.37
PA0406_at	231.58	201.40	0.484	1.15
PA0407_gshB_at	265.62	244.25	0.643	1.09
PA0408_pilG_at	587.52	745.75	0.048	-1.27
PA0409_pilH_at	1800.62	1501.50	0.087	1.20
PA0410_pilI_at	322.24	340.22	0.817	-1.06
PA0411_pilJ_at	512.10	452.40	0.008	1.13
PA0412_pilK_at	78.74	92.13	0.151	-1.17
PA0413_at	432.94	311.53	0.008	1.39
PA0414_at	136.46	105.52	0.008	1.29
PA0415_at	146.84	128.13	0.349	1.15
PA0416_at	26.52	33.78	0.087	-1.27
PA0417_at	26.12	30.47	0.349	-1.17
PA0418_at	58.86	43.70	0.048	1.35
PA0419_at	138.28	150.22	0.087	-1.09
PA0420_bioA_at	267.50	280.42	0.484	-1.05
PA0421_at	210.24	246.80	0.024	-1.17
PA0422_at	951.18	541.92	0.008	1.76
PA0423_at	224.52	187.63	0.349	1.20
PA0424_mexR_at	109.88	138.85	0.238	-1.26
PA0425_mexA_at	1221.46	877.78	0.008	1.39
PA0426_mexB_at	882.80	593.47	0.008	1.49
PA0427_oprM_at	793.90	605.08	0.008	1.31
PA0428_at	457.98	577.10	0.048	-1.26
PA0429_at	294.96	358.70	0.087	-1.22
PA0430_metF_at	473.98	672.47	0.008	-1.42
PA0431_at	764.92	850.28	0.087	-1.11
PA0432_sahH_at	1644.50	1510.13	0.087	1.09
PA0433_at	96.78	80.10	0.087	1.21
PA0434_at	30.06	41.18	0.087	-1.37
PA0435_at	39.50	20.45	0.024	1.93
PA0436_at	69.44	111.40	0.008	-1.60
PA0437_codA_at	236.64	285.80	0.111	-1.21
PA0438_codB_at	122.02	119.50	0.643	1.02
PA0439_at	8.58	6.45	0.643	1.33
PA0440_at	5.66	3.25	0.643	1.74
PA0441_at	6.66	10.40	0.349	-1.56
PA0442_r_at	3.90	0.00	0.008	
PA0443_at	9.88	11.10	1.000	-1.12
PA0444_at	12.26	19.88	0.286	-1.62
PA0445_s_at	90.40	94.07	0.730	-1.04
PA0446_at	116.10	74.10	0.024	1.57
PA0447_gcdH_at	328.46	150.43	0.008	2.18
PA0448_at	48.74	44.10	0.087	1.11
PA0449_at	143.90	200.50	0.024	-1.39
PA0450_at	10.64	5.13	0.151	2.07
PA0451_at	21.16	16.45	0.238	1.29
PA0452_at	4.26	13.57	0.024	-3.19
PA0453_at	5.02	14.15	0.048	-2.82
PA0454_at	78.76	61.47	0.024	1.28

Supplementary Table 1. Probe set signal data for experimental and control samples

PA0455_dbpA_at	89.74	89.50	0.817	1.00
PA0456_at	936.44	1206.55	0.024	-1.29
PA0457_at	83.70	97.63	0.063	-1.17
PA0458_at	69.54	69.70	1.000	-1.00
PA0459_at	39.88	35.57	0.349	1.12
PA0460_at	46.44	52.63	0.817	-1.13
PA0461_at	193.56	146.38	0.087	1.32
PA0462_at	66.94	51.82	0.349	1.29
PA0463_creB_at	142.62	110.52	0.087	1.29
PA0464_creC_at	44.40	40.95	1.000	1.08
PA0465_creD_at	5.72	10.35	0.087	-1.81
PA0466_at	9.80	10.80	0.643	-1.10
PA0467_at	189.36	161.02	0.024	1.18
PA0468_at	88.82	63.72	0.008	1.39
PA0469_at	125.68	97.80	0.048	1.29
PA0470_at	46.50	47.47	1.000	-1.02
PA0471_at	29.22	40.20	0.087	-1.38
PA0472_at	39.90	52.25	0.087	-1.31
PA0473_at	115.58	89.98	0.238	1.28
PA0474_at	12.62	5.85	0.238	2.16
PA0475_at	28.36	15.70	0.008	1.81
PA0476_at	17.60	6.10	0.048	2.89
PA0477_at	23.46	25.50	0.349	-1.09
PA0478_at	37.04	43.30	0.238	-1.17
PA0479_at	25.18	17.23	0.048	1.46
PA0480_at	8.46	4.45	0.087	1.90
PA0481_at	41.76	48.32	0.413	-1.16
PA0482_glcB_at	633.70	581.60	0.151	1.09
PA0483_at	20.42	17.70	0.817	1.15
PA0484_at	20.22	19.48	1.000	1.04
PA0485_at	55.86	54.00	0.817	1.03
PA0486_at	93.14	71.22	0.024	1.31
PA0487_at	84.70	72.40	0.349	1.17
PA0488_at	47.38	42.67	0.817	1.11
PA0489_at	12.78	10.55	0.992	1.21
PA0490_at	37.56	35.28	1.000	1.06
PA0491_at	31.46	42.72	0.238	-1.36
PA0492_at	420.24	210.28	0.024	2.00
PA0493_at	510.12	248.20	0.008	2.06
PA0494_at	497.00	219.90	0.008	2.26
PA0495_at	723.76	352.55	0.008	2.05
PA0496_at	127.28	68.20	0.008	1.87
PA0497_at	12.58	7.22	0.087	1.74
PA0498_at	7.92	3.80	0.286	2.08
PA0499_at	13.02	11.40	0.484	1.14
PA0500_bioB_at	230.88	286.15	0.087	-1.24
PA0501_bioF_at	135.70	150.48	0.484	-1.11
PA0502_at	107.50	117.97	0.238	-1.10
PA0503_at	68.88	81.52	0.349	-1.18
PA0504_bioD_at	38.28	43.05	0.643	-1.12
PA0505_at	84.44	86.27	0.484	-1.02

Supplementary Table 1. Probe set signal data for experimental and control samples

PA0506_at	130.80	178.78	0.048	-1.37
PA0507_at	35.86	24.65	0.008	1.45
PA0508_at	38.76	44.03	0.238	-1.14
PA0509_nirN_at	33.14	50.03	0.008	-1.51
PA0510_at	25.12	18.02	0.349	1.39
PA0511_nirJ_at	34.80	48.10	0.151	-1.38
PA0512_at	23.46	20.10	0.484	1.17
PA0513_at	22.82	22.20	0.349	1.03
PA0514_nirL_at	29.40	19.23	0.048	1.53
PA0515_at	51.78	43.58	0.643	1.19
PA0516_nirF_at	44.34	35.58	0.238	1.25
PA0517_nirC_at	66.06	59.13	0.349	1.12
PA0518_nirM_at	64.74	96.10	0.151	-1.48
PA0519_nirS_at	74.70	89.90	0.817	-1.20
PA0520_nirQ_at	41.10	34.18	0.349	1.20
PA0521_at	12.86	13.53	0.905	-1.05
PA0522_r_at	84.42	104.90	0.238	-1.24
PA0523_norC_at	11.80	8.15	0.087	1.45
PA0524_norB_at	10.40	5.33	0.111	1.95
PA0525_at	5.90	11.02	0.087	-1.87
PA0526_at	16.02	26.83	0.008	-1.67
PA0527_dnr_at	73.30	128.78	0.008	-1.76
PA0528_at	8.70	31.52	0.008	-3.62
PA0529_at	36.50	20.73	0.008	1.76
PA0530_at	24.78	17.98	0.230	1.38
PA0531_at	4.26	6.35	0.151	-1.49
PA0532_at	12.84	20.23	0.151	-1.58
PA0533_at	28.36	24.08	0.484	1.18
PA0534_at	16.18	17.95	0.730	-1.11
PA0535_at	27.26	26.35	0.817	1.03
PA0536_at	130.66	165.35	0.008	-1.27
PA0537_at	481.70	593.03	0.151	-1.23
PA0538_dsbB_at	73.86	78.60	0.484	-1.06
PA0539_at	14.48	7.07	0.190	2.05
PA0540_at	15.98	14.52	0.643	1.10
PA0541_at	170.82	240.30	0.024	-1.41
PA0542_at	215.32	242.88	0.087	-1.13
PA0543_at	10.32	17.80	0.087	-1.72
PA0544_at	56.38	62.75	0.484	-1.11
PA0545_at	25.76	32.25	0.151	-1.25
PA0546_metK_at	1382.82	979.47	0.024	1.41
PA0547_at	419.16	287.50	0.008	1.46
PA0548_tktA_at	609.36	819.50	0.008	-1.34
PA0549_at	89.90	146.40	0.008	-1.63
PA0550_at	41.00	28.35	0.048	1.45
PA0551_epd_at	592.42	538.05	0.349	1.10
PA0552_pgk_at	480.08	490.22	0.349	-1.02
PA0553_at	499.72	457.00	0.484	1.09
PA0554_at	691.80	852.55	0.008	-1.23
PA0555_fda_at	1564.84	1615.27	0.484	-1.03
PA0556_at	38.90	54.33	0.151	-1.40

Supplementary Table 1. Probe set signal data for experimental and control samples

PA0557_at	10.28	11.62	0.817	-1.13
PA0558_at	49.44	30.60	0.024	1.62
PA0559_at	93.12	130.10	0.008	-1.40
PA0560_at	39.22	49.72	0.048	-1.27
PA0561_at	14.32	17.38	0.643	-1.21
PA0562_at	168.70	172.10	0.484	-1.02
PA0563_at	442.30	715.25	0.008	-1.62
PA0564_at	51.38	44.25	0.230	1.16
PA0565_at	23.36	20.40	0.413	1.15
PA0566_at	60.92	59.50	0.817	1.02
PA0567_i_at	22.18	15.00	0.349	1.48
PA0568_at	50.60	53.70	0.817	-1.06
PA0569_at	45.90	86.40	0.008	-1.88
PA0570_at	79.10	80.78	0.817	-1.02
PA0571_at	101.48	121.38	0.151	-1.20
PA0572_at	60.98	47.62	0.008	1.28
PA0573_at	5.98	3.45	0.087	1.73
PA0574_at	66.12	74.00	0.484	-1.12
PA0575_at	6.78	9.00	0.484	-1.33
PA0576_rpoD_at	1462.74	1552.15	0.238	-1.06
PA0577_dnaG_at	370.00	265.08	0.008	1.40
PA0578_at	774.74	648.95	0.349	1.19
PA0579_rpsU_at	857.38	1630.68	0.008	-1.90
PA0580_gcp_at	89.92	130.23	0.008	-1.45
PA0581_i_at	51.16	52.13	0.484	-1.02
PA0582_folB_at	27.72	66.15	0.008	-2.39
PA0583_at	30.58	45.72	0.008	-1.50
PA0584_cca_at	35.02	32.38	0.230	1.08
PA0585_at	5.38	5.97	0.484	-1.11
PA0586_at	62.10	26.38	0.008	2.35
PA0587_at	43.36	16.88	0.008	2.57
PA0588_at	195.20	113.10	0.008	1.73
PA0589_at	146.04	161.57	0.151	-1.11
PA0590_apaH_at	465.70	314.27	0.008	1.48
PA0591_at	343.38	308.13	0.048	1.11
PA0592_ksgA_at	358.84	341.45	0.817	1.05
PA0593_pdxA_at	220.72	201.68	0.643	1.09
PA0594_surA_at	788.90	696.83	0.008	1.13
PA0595_ostA_at	1869.20	1460.27	0.008	1.28
PA0596_at	157.58	195.23	0.048	-1.24
PA0597_at	53.88	64.65	0.063	-1.20
PA0598_at	70.78	84.88	0.048	-1.20
PA0599_at	60.62	60.20	1.000	1.01
PA0600_at	25.56	20.30	0.151	1.26
PA0601_at	49.58	50.72	0.643	-1.02
PA0602_at	42.18	24.58	0.008	1.72
PA0603_at	34.32	45.07	0.008	-1.31
PA0604_at	25.42	20.65	0.151	1.23
PA0605_at	24.32	14.58	0.024	1.67
PA0606_at	12.84	13.33	0.349	-1.04
PA0607_rpe_at	191.16	213.10	0.024	-1.11

Supplementary Table 1. Probe set signal data for experimental and control samples

PA0608_at	408.62	465.38	0.151	-1.14
PA0609_trpE_at	92.86	117.15	0.008	-1.26
PA0610_prtN_at	103.66	72.15	0.151	1.44
PA0611_prtR_at	147.18	109.20	0.008	1.35
PA0612_i_at	941.52	177.77	0.008	5.30
PA0613_at	220.38	96.13	0.008	2.29
PA0614_at	635.46	220.03	0.008	2.89
PA0615_at	707.96	169.10	0.008	4.19
PA0616_at	1177.10	336.00	0.008	3.50
PA0617_at	708.94	211.50	0.008	3.35
PA0618_at	1085.44	350.05	0.008	3.10
PA0619_at	1189.22	392.45	0.008	3.03
PA0620_at	1328.84	313.42	0.008	4.24
PA0621_at	1909.64	742.50	0.008	2.57
PA0622_at	2536.46	833.83	0.008	3.04
PA0623_at	2053.48	740.50	0.008	2.77
PA0624_at	960.46	382.80	0.008	2.51
PA0625_at	537.44	159.45	0.008	3.37
PA0626_at	633.52	272.57	0.008	2.32
PA0627_at	630.28	270.02	0.008	2.33
PA0628_at	652.18	297.42	0.008	2.19
PA0629_at	407.90	154.82	0.008	2.63
PA0630_at	416.12	211.90	0.008	1.96
PA0631_at	294.56	128.25	0.008	2.30
PA0632_at	270.78	143.80	0.008	1.88
PA0633_at	1979.86	596.63	0.008	3.32
PA0634_at	589.38	169.75	0.008	3.47
PA0635_at	836.88	232.25	0.008	3.60
PA0636_at	1278.80	380.55	0.008	3.36
PA0637_at	589.80	178.42	0.008	3.31
PA0638_at	908.58	283.90	0.008	3.20
PA0639_at	617.88	209.70	0.008	2.95
PA0640_at	141.98	55.60	0.008	2.55
PA0641_at	509.66	102.02	0.008	5.00
PA0642_at	45.10	16.23	0.008	2.78
PA0643_at	135.54	65.72	0.008	2.06
PA0644_at	210.30	119.28	0.008	1.76
PA0645_at	222.88	88.80	0.008	2.51
PA0646_at	158.00	64.23	0.008	2.46
PA0647_at	50.84	39.95	0.341	1.27
PA0648_at	143.44	94.75	0.024	1.51
PA0649_trpG_at	414.22	353.92	0.151	1.17
PA0650_trpD_at	270.24	251.15	0.643	1.08
PA0651_trpC_at	310.02	190.35	0.008	1.63
PA0652_vfr_at	940.92	783.53	0.048	1.20
PA0653_at	62.44	113.20	0.008	-1.81
PA0654_speD_at	416.94	804.38	0.008	-1.93
PA0655_at	210.64	182.10	0.008	1.16
PA0656_at	33.50	34.00	0.817	-1.01
PA0657_at	69.14	55.63	0.087	1.24
PA0658_at	114.56	109.98	0.643	1.04

Supplementary Table 1. Probe set signal data for experimental and control samples

PA0659_at	581.30	411.33	0.008	1.41
PA0660_at	212.32	135.38	0.008	1.57
PA0661_at	401.74	390.92	1.000	1.03
PA0662_argC_at	163.60	181.77	0.087	-1.11
PA0663_at	132.40	194.13	0.008	-1.47
PA0664_at	136.98	161.02	0.349	-1.18
PA0665_at	122.40	171.88	0.024	-1.40
PA0666_at	102.18	89.85	0.349	1.14
PA0667_at	345.88	416.77	0.151	-1.20
PA0668_tyrZ_at	262.62	334.98	0.008	-1.28
PA0669_at	59.36	20.92	0.008	2.84
PA0670_at	107.54	11.42	0.008	9.42
PA0671_at	127.50	25.42	0.008	5.02
PA0672_at	30.46	38.20	0.349	-1.25
PA0673_at	23.54	16.97	0.484	1.39
PA0674_at	12.20	9.30	0.643	1.31
PA0675_at	15.36	15.75	0.643	-1.03
PA0676_at	15.02	10.60	0.151	1.42
PA0677_at	2.90	5.52	0.087	-1.90
PA0678_i_at	11.44	4.82	0.151	2.37
PA0679_at	16.40	14.23	0.238	1.15
PA0680_at	8.42	10.90	0.556	-1.29
PA0681_at	14.80	10.60	0.151	1.40
PA0682_at	15.98	13.73	0.817	1.16
PA0683_at	9.78	5.85	0.349	1.67
PA0684_at	20.98	11.80	0.151	1.78
PA0685_at	5.74	5.35	0.817	1.07
PA0686_at	13.10	11.17	0.413	1.17
PA0687_at	15.96	4.80	0.063	3.32
PA0688_at	8.58	4.98	0.238	1.72
PA0689_at	14.94	11.90	0.484	1.26
PA0690_at	4.20	1.50	0.063	2.80
PA0691_at	13.66	9.75	0.238	1.40
PA0692_at	22.86	10.60	0.008	2.16
PA0693_exbB2_at	11.82	11.43	0.817	1.03
PA0694_exbD2_at	24.88	30.88	0.643	-1.24
PA0695_at	6.62	4.15	0.817	1.60
PA0696_at	11.90	2.35	0.087	5.06
PA0697_at	7.42	11.80	0.151	-1.59
PA0698_at	11.96	13.38	1.000	-1.12
PA0699_at	16.34	21.30	0.048	-1.30
PA0700_at	10.44	18.25	0.151	-1.75
PA0701_at	3.24	4.65	0.643	-1.44
PA0702_at	7.74	7.27	0.817	1.06
PA0703_at	39.60	42.00	0.643	-1.06
PA0704_at	32.36	29.90	0.349	1.08
PA0705_at	402.62	342.02	0.151	1.18
PA0706_cat_at	123.90	164.20	0.016	-1.33
PA0707_toxR_at	32.18	24.52	0.024	1.31
PA0708_at	69.66	71.30	1.000	-1.02
PA0709_at	12.72	16.75	0.238	-1.32

Supplementary Table 1. Probe set signal data for experimental and control samples

PA0710_gloA2_at	8.50	9.15	0.643	-1.08
PA0711_at	14.56	13.32	0.730	1.09
PA0712_at	41.68	31.55	0.048	1.32
PA0713_at	24.38	21.27	0.413	1.15
PA0714_at	4.68	3.10	0.730	1.51
PA0715_at	26.84	12.50	0.087	2.15
PA0716_at	6.32	3.03	0.484	2.09
PA0717_at	5788.04	349.48	0.087	16.56
PA0718_at	5242.88	334.15	0.151	15.69
PA0719_at	4110.20	215.20	0.151	19.10
PA0720_at	7289.32	751.30	0.151	9.70
PA0721_r_at	4921.56	252.35	0.151	19.50
PA0722_at	7302.12	954.47	0.151	7.65
PA0723_coaB_at	6924.28	602.40	0.087	11.49
PA0724_at	3359.20	98.75	0.087	34.02
PA0725_at	859.52	34.30	0.151	25.06
PA0726_at	2952.62	104.85	0.087	28.16
PA0727_at	2308.50	106.72	0.151	21.63
PA0728_at	453.36	26.07	0.048	17.39
PA0729_at	256.82	89.28	0.151	2.88
PA0730_at	351.64	347.65	0.643	1.01
PA0731_at	49.42	54.78	0.151	-1.11
PA0732_at	37.92	44.05	0.643	-1.16
PA0733_at	76.06	85.07	0.349	-1.12
PA0734_i_at	149.12	141.65	0.817	1.05
PA0735_at	119.76	122.80	0.817	-1.03
PA0736_at	93.12	78.53	0.024	1.19
PA0737_at	7.86	6.00	0.349	1.31
PA0738_at	20.54	15.42	0.286	1.33
PA0739_at	33.26	35.13	0.484	-1.06
PA0740_at	8.12	10.35	0.556	-1.27
PA0741_at	7.92	20.60	0.008	-2.60
PA0742_at	15.40	25.38	0.048	-1.65
PA0743_at	9.40	9.90	0.817	-1.05
PA0744_at	246.50	137.40	0.008	1.79
PA0745_at	513.24	168.75	0.008	3.04
PA0746_at	121.86	39.78	0.008	3.06
PA0747_at	68.10	28.17	0.008	2.42
PA0748_at	3.74	6.35	0.238	-1.70
PA0749_at	53.52	68.05	0.048	-1.27
PA0750_ung_at	124.18	148.13	0.024	-1.19
PA0751_at	22.72	21.72	0.484	1.05
PA0752_at	36.06	34.80	1.000	1.04
PA0753_at	19.66	14.27	0.151	1.38
PA0754_at	50.46	51.47	0.643	-1.02
PA0755_at	29.64	29.92	0.817	-1.01
PA0756_at	86.28	73.50	0.151	1.17
PA0757_at	82.78	74.70	0.238	1.11
PA0758_at	117.08	196.27	0.048	-1.68
PA0759_at	151.12	148.98	0.810	1.01
PA0760_at	86.58	138.48	0.008	-1.60

Supplementary Table 1. Probe set signal data for experimental and control samples

PA0761_nadB_at	142.28	152.75	0.087	-1.07
PA0762_algU_at	864.06	837.63	0.643	1.03
PA0763_mucA_at	752.78	587.60	0.008	1.28
PA0764_mucB_at	203.10	142.07	0.008	1.43
PA0765_mucC_at	231.00	263.05	0.087	-1.14
PA0766_mucD_at	338.40	512.15	0.008	-1.51
PA0767_lepA_at	279.86	371.05	0.008	-1.33
PA0768_lepB_at	340.76	464.05	0.008	-1.36
PA0769_at	210.62	338.33	0.008	-1.61
PA0770_rnc_at	334.28	454.40	0.048	-1.36
PA0771_era_at	287.58	350.65	0.087	-1.22
PA0772_recO_at	39.32	39.95	0.817	-1.02
PA0773_pdxJ_at	68.24	99.75	0.008	-1.46
PA0774_at	46.42	76.30	0.048	-1.64
PA0775_at	151.66	174.08	0.087	-1.15
PA0776_at	21.86	11.32	0.087	1.93
PA0777_at	114.40	53.47	0.024	2.14
PA0778_at	171.88	158.60	0.087	1.08
PA0779_at	489.82	459.05	0.817	1.07
PA0780_at	78.88	59.05	0.008	1.34
PA0781_at	40.34	38.70	0.643	1.04
PA0782_putA_at	3614.68	2152.15	0.008	1.68
PA0783_putP_at	2126.62	1222.43	0.008	1.74
PA0784_at	72.48	52.70	0.008	1.38
PA0785_at	14.14	19.15	0.048	-1.35
PA0786_at	7.90	7.60	1.000	1.04
PA0787_at	73.14	64.20	0.151	1.14
PA0788_at	29.34	28.23	0.817	1.04
PA0789_at	371.96	499.85	0.151	-1.34
PA0790_at	7.96	6.75	1.000	1.18
PA0791_at	40.16	43.52	0.238	-1.08
PA0792_prpD_at	84.80	73.25	0.484	1.16
PA0793_at	447.26	301.88	0.151	1.48
PA0794_at	343.54	239.43	0.151	1.43
PA0795_prpC_at	1137.02	809.10	0.087	1.41
PA0796_prpB_at	758.96	496.38	0.087	1.53
PA0797_at	372.34	317.23	0.643	1.17
PA0798_at	16.04	13.12	1.000	1.22
PA0799_at	44.80	57.47	0.151	-1.28
PA0800_at	24.26	32.35	0.048	-1.33
PA0801_at	63.54	68.02	0.484	-1.07
PA0802_i_at	46.42	43.73	1.000	1.06
PA0803_at	8.20	17.52	0.008	-2.14
PA0804_at	49.82	59.97	0.024	-1.20
PA0805_at	136.64	54.77	0.151	2.49
PA0806_i_at	12.72	13.13	0.643	-1.03
PA0807_at	82.70	74.38	0.484	1.11
PA0808_at	15.60	10.05	0.151	1.55
PA0809_at	22.00	17.73	0.413	1.24
PA0810_at	45.64	31.65	0.008	1.44
PA0811_at	4.94	3.23	1.000	1.53

Supplementary Table 1. Probe set signal data for experimental and control samples

PA0812_at	21.84	17.73	0.063	1.23
PA0813_at	19.64	27.75	0.048	-1.41
PA0814_i_at	14.82	16.40	0.643	-1.11
PA0815_at	35.66	28.78	0.238	1.24
PA0816_at	26.06	24.02	0.817	1.08
PA0817_at	34.98	29.50	0.484	1.19
PA0818_at	30.06	30.38	1.000	-1.01
PA0819_f_at	20.40	34.28	0.238	-1.68
PA0820_at	51.22	76.82	0.008	-1.50
PA0821_at	34.12	34.40	0.643	-1.01
PA0822_at	15.84	16.50	0.817	-1.04
PA0823_at	5.46	7.58	0.556	-1.39
PA0824_at	16.90	16.70	0.643	1.01
PA0825_at	3.66	5.05	0.413	-1.38
PA0826_at	26.20	23.45	0.484	1.12
PA0827_at	66.48	58.93	0.048	1.13
PA0828_at	19.40	19.17	0.817	1.01
PA0829_at	16.16	13.63	0.817	1.19
PA0830_at	42.48	32.80	0.048	1.30
PA0831_oruR_at	285.58	147.73	0.008	1.93
PA0832_at	188.44	187.98	0.817	1.00
PA0833_at	385.64	395.00	0.817	-1.02
PA0834_at	49.72	68.15	0.048	-1.37
PA0835_pta_at	228.62	178.85	0.087	1.28
PA0836_at	315.28	245.17	0.087	1.29
PA0837_slyD_at	359.32	588.30	0.008	-1.64
PA0838_at	131.34	163.38	0.349	-1.24
PA0839_at	45.24	35.45	0.341	1.28
PA0840_at	40.40	41.85	0.905	-1.04
PA0841_at	80.84	49.15	0.024	1.64
PA0842_at	13.66	17.35	0.238	-1.27
PA0843_plcR_at	14.26	11.73	0.905	1.22
PA0844_plcH_at	8.06	7.48	0.643	1.08
PA0845_at	3.74	6.20	0.413	-1.66
PA0846_at	60.88	46.07	0.048	1.32
PA0847_at	35.90	12.85	0.008	2.79
PA0848_at	75.80	115.07	0.008	-1.52
PA0849_trxB2_at	51.08	77.88	0.024	-1.52
PA0850_at	19.60	13.20	0.048	1.48
PA0851_at	49.36	36.05	0.008	1.37
PA0852_cpbD_at	154.68	174.85	0.484	-1.13
PA0853_at	83.76	73.85	0.484	1.13
PA0854_fumC2_at	108.46	91.20	0.151	1.19
PA0855_at	160.50	153.00	0.817	1.05
PA0856_at	690.50	860.92	0.008	-1.25
PA0857_bolA_at	313.88	378.63	0.151	-1.21
PA0858_at	179.06	277.92	0.008	-1.55
PA0859_at	93.96	82.65	0.238	1.14
PA0860_at	72.98	65.68	0.556	1.11
PA0861_at	49.58	32.63	0.008	1.52
PA0862_at	191.40	133.05	0.024	1.44

Supplementary Table 1. Probe set signal data for experimental and control samples

PA0863_at	2.90	4.18	0.349	-1.44
PA0864_at	25.10	24.18	0.484	1.04
PA0865_hpd_at	2186.32	1528.60	0.008	1.43
PA0866_aroP2_at	780.40	343.60	0.008	2.27
PA0867_at	263.38	247.77	0.817	1.06
PA0868_at	55.20	47.38	0.087	1.17
PA0869_pbpG_at	214.06	184.20	0.008	1.16
PA0870_phhC_at	2953.46	2060.13	0.008	1.43
PA0871_phhB_at	2395.84	1881.25	0.008	1.27
PA0872_phhA_at	2831.98	1935.43	0.008	1.46
PA0873_phhR_at	40.60	17.38	0.008	2.34
PA0874_at	19.44	8.53	0.048	2.28
PA0875_at	11.88	12.50	0.643	-1.05
PA0876_at	99.82	97.62	1.000	1.02
PA0877_at	13.70	32.30	0.024	-2.36
PA0878_at	11.60	12.75	0.643	-1.10
PA0879_at	2.84	3.15	0.484	-1.11
PA0880_at	15.44	10.63	0.349	1.45
PA0881_at	14.46	16.63	0.484	-1.15
PA0882_at	19.98	8.05	0.024	2.48
PA0883_at	5.22	7.30	0.349	-1.40
PA0884_at	10.48	9.73	0.730	1.08
PA0885_at	6.24	1.97	0.349	3.17
PA0886_at	7.46	8.73	0.556	-1.17
PA0887_acsA_at	1639.96	803.35	0.024	2.04
PA0888_aotJ_at	925.60	882.88	0.643	1.05
PA0889_aotQ_at	276.76	237.57	0.151	1.16
PA0890_aotM_at	319.10	342.15	0.151	-1.07
PA0891_at	432.24	376.83	0.048	1.15
PA0892_aotP_at	633.78	703.35	0.238	-1.11
PA0893_argR_at	249.86	210.25	0.087	1.19
PA0894_at	15.22	11.47	0.151	1.33
PA0895_aruC_at	912.64	609.38	0.008	1.50
PA0896_aruF_at	291.12	402.58	0.024	-1.38
PA0897_aruG_at	345.70	324.00	0.349	1.07
PA0898_aruD_at	359.72	317.75	0.087	1.13
PA0899_aruB_at	442.84	443.55	1.000	-1.00
PA0900_at	1497.06	1346.63	0.048	1.11
PA0901_aruE_at	292.96	288.83	0.643	1.01
PA0902_at	129.66	117.88	0.643	1.10
PA0903_alaS_at	334.98	373.28	0.238	-1.11
PA0904_lysC_at	468.76	625.98	0.008	-1.34
PA0905_csrA_at	124.68	315.30	0.008	-2.53
PA0906_at	72.76	44.03	0.087	1.65
PA0907_at	43.68	29.40	0.048	1.49
PA0908_at	23.12	20.45	0.643	1.13
PA0909_i_at	30.96	38.85	0.087	-1.25
PA0910_at	66.52	65.97	0.643	1.01
PA0911_at	95.74	83.90	0.238	1.14
PA0912_at	12.72	19.90	0.349	-1.56
PA0913_mgtE_at	323.94	310.60	0.643	1.04

Supplementary Table 1. Probe set signal data for experimental and control samples

PA0914_at	18.16	41.78	0.008	-2.30
PA0915_at	79.78	155.77	0.008	-1.95
PA0916_at	145.50	313.15	0.008	-2.15
PA0917_kup_at	81.78	116.63	0.008	-1.43
PA0918_at	78.38	120.33	0.008	-1.54
PA0919_at	112.10	102.85	0.151	1.09
PA0920_at	61.90	62.98	1.000	-1.02
PA0921_at	103.28	173.20	0.008	-1.68
PA0922_at	431.28	115.50	0.008	3.73
PA0923_dinP_at	94.28	71.07	0.048	1.33
PA0924_at	24.92	30.47	0.484	-1.22
PA0925_at	152.38	142.35	0.484	1.07
PA0926_at	262.22	233.30	0.349	1.12
PA0927_ldhA_at	155.80	105.23	0.008	1.48
PA0928_at	53.62	51.92	0.817	1.03
PA0929_at	38.22	53.30	0.008	-1.39
PA0930_at	24.28	21.98	0.643	1.10
PA0931_at	21.58	17.53	0.643	1.23
PA0932_cysM_at	409.10	410.33	0.484	-1.00
PA0933_ygcA_at	200.20	231.48	0.048	-1.16
PA0934_relA_at	262.68	346.97	0.008	-1.32
PA0935_at	122.58	125.82	0.643	-1.03
PA0936_at	69.56	104.15	0.008	-1.50
PA0937_at	299.58	558.22	0.008	-1.86
PA0938_at	147.72	266.43	0.008	-1.80
PA0939_at	2.74	4.13	0.349	-1.51
PA0940_at	29.82	35.25	0.238	-1.18
PA0941_at	31.02	28.48	0.817	1.09
PA0942_at	35.48	39.72	0.643	-1.12
PA0943_at	623.12	801.55	0.024	-1.29
PA0944_purN_at	250.88	286.45	0.238	-1.14
PA0945_purM_at	464.96	614.35	0.024	-1.32
PA0946_at	160.60	108.00	0.008	1.49
PA0947_at	126.54	156.15	0.048	-1.23
PA0948_at	114.62	115.35	1.000	-1.01
PA0949_wrbA_at	295.64	300.48	0.817	-1.02
PA0950_at	351.22	435.80	0.008	-1.24
PA0951_at	37.68	25.52	0.024	1.48
PA0952_at	44.18	36.10	0.238	1.22
PA0953_at	65.02	64.35	0.817	1.01
PA0954_at	18.50	20.27	0.905	-1.10
PA0955_at	219.16	254.97	0.238	-1.16
PA0956_proS_at	740.78	719.55	0.643	1.03
PA0957_at	13.82	9.30	0.151	1.49
PA0958_oprD_at	1913.82	1684.07	0.087	1.14
PA0959_at	113.10	126.22	0.151	-1.12
PA0960_at	186.20	186.48	0.643	-1.00
PA0961_at	200.48	256.88	0.024	-1.28
PA0962_at	707.78	662.75	1.000	1.07
PA0963_aspS_at	695.76	817.58	0.008	-1.18
PA0964_at	472.84	815.55	0.008	-1.72

Supplementary Table 1. Probe set signal data for experimental and control samples

PA0965_ruvC_at	334.04	507.45	0.008	-1.52
PA0966_ruvA_at	196.46	319.50	0.008	-1.63
PA0967_ruvB_at	172.60	234.53	0.008	-1.36
PA0968_at	342.24	538.92	0.008	-1.57
PA0969_tolQ_at	604.54	822.65	0.008	-1.36
PA0970_tolR_at	359.44	548.08	0.008	-1.52
PA0971_tolA_at	393.68	477.85	0.024	-1.21
PA0972_tolB_at	890.60	1035.60	0.048	-1.16
PA0973_oprL_at	1211.02	1406.47	0.087	-1.16
PA0974_at	663.62	585.70	0.238	1.13
PA0975_at	125.78	299.52	0.008	-2.38
PA0976_at	51.60	137.65	0.008	-2.67
PA0977_at	2.12	7.05	0.008	-3.33
PA0978_s_at	31.18	28.45	0.817	1.10
PA0979_s_at	9.20	20.70	0.008	-2.25
PA0980_at	18.88	15.15	0.643	1.25
PA0981_at	2.52	6.37	0.008	-2.53
PA0982_at	25.90	21.90	0.349	1.18
PA0983_at	39.92	20.13	0.024	1.98
PA0984_at	3.12	1.30	0.349	2.40
PA0985_at	86.90	45.15	0.024	1.92
PA0986_i_at	4.48	0.57	0.024	7.86
PA0987_at	7.92	8.80	0.817	-1.11
PA0988_at	33.36	27.13	0.484	1.23
PA0989_at	24.52	40.22	0.008	-1.64
PA0990_at	6.00	6.13	0.635	-1.02
PA0991_at	8.26	8.00	0.817	1.03
PA0992_at	21.00	36.45	0.087	-1.74
PA0993_at	5.50	4.60	1.000	1.20
PA0994_at	11.04	14.55	0.349	-1.32
PA0995_ogt_at	24.94	21.88	0.349	1.14
PA0996_at	214.58	430.15	0.008	-2.00
PA0997_at	476.72	820.50	0.008	-1.72
PA0998_at	319.06	607.78	0.008	-1.90
PA0999_fabH1_at	434.04	770.02	0.008	-1.77
PA1000_at	130.58	315.80	0.008	-2.42
PA1001_phnA_at	129.32	257.05	0.008	-1.99
PA1002_phnB_at	57.76	123.38	0.008	-2.14
PA1003_at	71.20	115.53	0.008	-1.62
PA1004_nadA_at	598.70	587.08	0.817	1.02
PA1005_at	173.98	135.93	0.048	1.28
PA1006_at	73.74	60.95	0.643	1.21
PA1007_at	27.14	31.38	0.151	-1.16
PA1008_bcp_at	726.58	715.65	0.643	1.02
PA1009_at	875.66	795.17	0.087	1.10
PA1010_dapA_at	987.14	825.70	0.024	1.20
PA1011_at	631.26	604.53	0.349	1.04
PA1012_at	71.68	118.60	0.008	-1.65
PA1013_purC_at	357.62	653.95	0.008	-1.83
PA1014_at	90.22	87.65	0.817	1.03
PA1015_at	101.30	90.15	0.349	1.12

Supplementary Table 1. Probe set signal data for experimental and control samples

PA1016_at	55.16	45.63	0.238	1.21
PA1017_pauA_at	18.32	22.90	0.413	-1.25
PA1018_at	29.14	26.10	0.643	1.12
PA1019_mucK_at	8.48	5.08	0.349	1.67
PA1020_at	31.78	25.23	0.151	1.26
PA1021_at	25.54	21.18	0.349	1.21
PA1022_at	85.16	65.38	0.087	1.30
PA1023_at	38.36	34.83	0.151	1.10
PA1024_at	28.06	43.47	0.087	-1.55
PA1025_at	9.98	8.68	0.817	1.15
PA1026_at	58.34	61.00	0.643	-1.05
PA1027_at	32.80	34.42	0.817	-1.05
PA1028_at	14.92	21.55	0.048	-1.44
PA1029_at	109.70	96.80	0.484	1.13
PA1030_at	56.94	45.05	0.151	1.26
PA1031_at	106.26	94.78	0.413	1.12
PA1032_at	54.60	76.07	0.008	-1.39
PA1033_at	136.64	130.52	0.643	1.05
PA1034_at	24.34	44.17	0.008	-1.81
PA1035_at	82.30	91.48	0.484	-1.11
PA1036_at	74.60	59.67	0.048	1.25
PA1037_at	38.22	49.15	0.087	-1.29
PA1038_at	23.96	29.57	0.484	-1.23
PA1039_at	125.00	88.97	0.008	1.40
PA1040_at	138.36	82.53	0.008	1.68
PA1041_at	43.86	19.10	0.008	2.30
PA1042_at	53.32	54.75	0.643	-1.03
PA1043_at	135.40	116.07	0.048	1.17
PA1044_at	81.46	42.17	0.008	1.93
PA1045_at	201.88	136.38	0.008	1.48
PA1046_at	58.72	35.42	0.024	1.66
PA1047_at	214.92	176.25	0.008	1.22
PA1048_at	319.44	399.48	0.008	-1.25
PA1049_pdxH_at	178.86	186.95	0.238	-1.05
PA1050_at	41.78	46.03	0.349	-1.10
PA1051_at	14.54	57.75	0.817	-3.97
PA1052_at	10.32	21.02	0.349	-2.04
PA1053_at	2926.70	2130.95	0.024	1.37
PA1054_at	86.60	80.30	0.349	1.08
PA1055_at	104.20	97.88	0.643	1.06
PA1056_at	79.48	70.28	0.238	1.13
PA1057_at	135.52	129.05	0.484	1.05
PA1058_at	103.50	124.52	0.151	-1.20
PA1059_at	74.08	86.02	0.238	-1.16
PA1060_at	41.70	42.22	1.000	-1.01
PA1061_at	98.28	97.45	0.817	1.01
PA1062_at	50.48	57.20	0.151	-1.13
PA1063_at	22.54	22.17	0.817	1.02
PA1064_at	259.02	256.38	1.000	1.01
PA1065_at	18.10	18.00	0.817	1.01
PA1066_at	4.00	1.75	0.151	2.29

Supplementary Table 1. Probe set signal data for experimental and control samples

PA1067_at	27.34	20.52	0.008	1.33
PA1068_at	546.20	395.87	0.008	1.38
PA1069_at	234.26	260.35	0.484	-1.11
PA1070_braG_at	209.40	229.38	0.643	-1.10
PA1071_braF_at	655.00	569.92	0.238	1.15
PA1072_braE_at	351.78	259.85	0.048	1.35
PA1073_braD_at	369.24	381.50	0.817	-1.03
PA1074_braC_at	1529.06	1154.25	0.048	1.32
PA1075_at	98.34	118.32	0.024	-1.20
PA1076_at	68.60	93.10	0.048	-1.36
PA1077_flgB_at	386.80	246.63	0.151	1.57
PA1078_flgC_at	356.34	325.90	0.087	1.09
PA1079_flgD_at	418.94	390.40	0.349	1.07
PA1080_flgE_at	879.50	771.50	0.024	1.14
PA1081_flgF_at	198.14	174.05	0.349	1.14
PA1082_flgG_at	448.26	428.47	0.817	1.05
PA1083_flgH_at	164.26	158.53	0.817	1.04
PA1084_flgI_at	142.30	138.02	0.817	1.03
PA1085_flgJ_at	171.94	149.18	0.087	1.15
PA1086_flgK_at	235.80	231.30	0.817	1.02
PA1087_flgL_at	356.38	328.73	0.151	1.08
PA1088_at	117.04	145.07	0.008	-1.24
PA1089_at	155.80	126.13	0.048	1.24
PA1090_at	106.20	105.83	0.817	1.00
PA1091_at	118.30	125.47	0.238	-1.06
PA1092_fliC_at	3375.92	2972.55	0.008	1.14
PA1093_at	728.88	834.47	0.087	-1.14
PA1094_fliD_at	499.20	655.78	0.008	-1.31
PA1095_at	467.48	675.00	0.024	-1.44
PA1096_at	968.54	928.30	0.643	1.04
PA1097_fleQ_at	325.12	289.72	0.643	1.12
PA1098_fleS_at	234.46	157.57	0.008	1.49
PA1099_fleR_at	193.90	159.95	0.008	1.21
PA1100_fliE_at	263.40	220.45	0.151	1.19
PA1101_fliF_at	280.92	289.60	0.643	-1.03
PA1102_fliG_at	574.26	487.80	0.024	1.18
PA1103_at	288.64	234.80	0.048	1.23
PA1104_fliI_at	109.62	109.93	1.000	-1.00
PA1105_fliJ_at	40.22	38.72	0.817	1.04
PA1106_at	57.00	35.98	0.008	1.58
PA1107_at	10.36	10.50	0.817	-1.01
PA1108_at	8.62	8.63	0.817	-1.00
PA1109_at	0.86	1.00	0.270	-1.16
PA1110_at	22.00	19.95	0.817	1.10
PA1111_at	12.88	17.75	0.087	-1.38
PA1112_at	58.56	23.25	0.008	2.52
PA1113_at	20.56	16.55	0.349	1.24
PA1114_at	44.76	49.75	0.484	-1.11
PA1115_at	67.90	54.30	0.087	1.25
PA1116_at	88.26	135.33	0.008	-1.53
PA1117_at	37.32	54.10	0.008	-1.45

Supplementary Table 1. Probe set signal data for experimental and control samples

PA1118_at	11.94	16.83	0.151	-1.41
PA1119_at	108.72	129.73	0.484	-1.19
PA1120_at	34.80	30.75	0.238	1.13
PA1121_at	53.88	51.12	0.905	1.05
PA1122_at	114.22	104.07	0.484	1.10
PA1123_at	18.92	34.47	0.048	-1.82
PA1124_dgt_at	46.56	30.92	0.024	1.51
PA1125_at	49.86	39.15	0.048	1.27
PA1126_at	106.42	176.10	0.008	-1.65
PA1127_at	122.10	118.78	0.484	1.03
PA1128_at	50.74	60.78	0.024	-1.20
PA1129_at	11.36	14.45	0.341	-1.27
PA1130_at	18.14	22.52	0.413	-1.24
PA1131_at	22.70	20.35	0.484	1.12
PA1132_at	147.42	114.23	0.024	1.29
PA1133_at	2.92	6.00	0.151	-2.05
PA1134_at	21.94	22.32	1.000	-1.02
PA1135_at	35.94	32.05	0.238	1.12
PA1136_at	9.46	13.60	0.238	-1.44
PA1137_at	14.28	14.95	1.000	-1.05
PA1138_at	23.18	22.47	0.817	1.03
PA1139_at	31.94	25.60	0.008	1.25
PA1140_at	61.36	67.13	0.048	-1.09
PA1141_at	50.12	53.50	0.643	-1.07
PA1142_at	56.24	46.72	0.238	1.20
PA1143_at	10.02	15.83	0.238	-1.58
PA1144_at	2.10	5.07	0.048	-2.41
PA1145_at	29.44	26.07	0.230	1.13
PA1146_at	28.44	15.13	0.008	1.88
PA1147_at	24.20	16.35	0.151	1.48
PA1148_toxA_at	21.46	20.47	0.484	1.05
PA1149_at	51.92	61.22	0.151	-1.18
PA1150_pys2_at	359.30	123.93	0.008	2.90
PA1151_imm2_at	34.88	71.58	0.008	-2.05
PA1152_at	17.00	18.50	0.484	-1.09
PA1153_at	35.92	30.03	0.151	1.20
PA1154_at	19.10	11.70	0.048	1.63
PA1155_nrdB_at	2162.86	1895.00	0.087	1.14
PA1156_nrdA_at	1371.28	1106.53	0.048	1.24
PA1157_at	157.14	112.18	0.008	1.40
PA1158_at	37.42	31.22	0.087	1.20
PA1159_at	752.46	1119.18	0.048	-1.49
PA1160_at	117.88	135.23	0.238	-1.15
PA1161_rrmA_at	99.34	105.13	0.349	-1.06
PA1162_dapE_at	276.58	273.48	0.817	1.01
PA1163_at	24.58	33.43	0.048	-1.36
PA1164_at	84.04	79.93	0.643	1.05
PA1165_at	46.44	75.03	0.008	-1.62
PA1166_at	24.68	12.35	0.008	2.00
PA1167_at	39.50	34.47	0.238	1.15
PA1168_at	3.60	14.13	0.008	-3.93

Supplementary Table 1. Probe set signal data for experimental and control samples

PA1169_at	7.40	1.55	0.024	4.77
PA1170_at	24.92	28.72	0.643	-1.15
PA1171_at	74.56	75.55	0.817	-1.01
PA1172_napC_at	11.62	19.92	0.349	-1.71
PA1173_napB_at	30.16	15.45	0.008	1.95
PA1174_napA_at	27.76	26.05	0.817	1.07
PA1175_napD_at	39.20	32.30	0.143	1.21
PA1176_napF_at	19.32	12.70	0.024	1.52
PA1177_napE_at	12.00	5.10	0.024	2.35
PA1178_oprH_at	2710.64	2888.25	0.484	-1.07
PA1179_phoP_at	681.22	602.28	0.048	1.13
PA1180_phoQ_at	319.92	279.93	0.024	1.14
PA1181_at	48.44	59.07	0.048	-1.22
PA1182_at	62.70	60.38	0.817	1.04
PA1183_dctA_at	22.70	15.92	0.048	1.43
PA1184_at	19.40	14.43	0.643	1.34
PA1185_at	11.92	13.75	0.349	-1.15
PA1186_at	7.82	8.43	0.484	-1.08
PA1187_at	16.12	18.98	0.484	-1.18
PA1188_at	22.00	16.52	0.048	1.33
PA1189_at	55.62	61.58	0.484	-1.11
PA1190_at	5.68	10.05	0.008	-1.77
PA1191_at	17.72	24.63	0.087	-1.39
PA1192_at	329.52	360.13	0.151	-1.09
PA1193_at	114.00	174.73	0.008	-1.53
PA1194_at	12.22	12.70	0.817	-1.04
PA1195_at	17.62	20.35	0.349	-1.15
PA1196_at	19.84	25.20	0.349	-1.27
PA1197_at	27.90	29.77	0.817	-1.07
PA1198_at	1111.94	1274.80	0.008	-1.15
PA1199_at	235.98	376.13	0.008	-1.59
PA1200_at	39.86	68.45	0.008	-1.72
PA1201_at	56.20	60.83	0.643	-1.08
PA1202_at	116.10	130.70	0.048	-1.13
PA1203_at	150.96	139.20	0.484	1.08
PA1204_at	49.10	26.70	0.008	1.84
PA1205_at	65.16	41.35	0.024	1.58
PA1206_at	195.40	238.35	0.087	-1.22
PA1207_kefB_at	38.46	58.22	0.024	-1.51
PA1208_at	15.80	18.52	0.413	-1.17
PA1209_at	35.78	26.03	0.024	1.37
PA1210_at	8.86	5.20	0.048	1.70
PA1211_at	8.84	4.10	0.151	2.16
PA1212_at	5.80	10.73	0.048	-1.85
PA1213_at	19.06	19.80	0.817	-1.04
PA1214_at	23.70	17.95	0.151	1.32
PA1215_at	3.90	4.00	0.643	-1.03
PA1216_at	18.84	24.45	0.087	-1.30
PA1217_at	8.50	14.88	0.087	-1.75
PA1218_at	6.94	9.63	0.349	-1.39
PA1219_at	9.64	22.95	0.048	-2.38

Supplementary Table 1. Probe set signal data for experimental and control samples

PA1220_at	18.14	16.68	0.413	1.09
PA1221_at	24.04	33.17	0.151	-1.38
PA1222_at	101.14	103.03	0.905	-1.02
PA1223_at	12.78	13.77	0.817	-1.08
PA1224_at	45.44	43.88	0.484	1.04
PA1225_at	22.20	26.02	0.349	-1.17
PA1226_at	40.02	53.30	0.238	-1.33
PA1227_at	13.26	25.47	0.008	-1.92
PA1228_at	184.56	509.92	0.008	-2.76
PA1229_at	40.56	39.18	1.000	1.04
PA1230_at	15.62	11.18	0.484	1.40
PA1231_at	15.18	10.25	0.238	1.48
PA1232_at	12.86	6.72	0.087	1.91
PA1233_at	17.46	24.03	0.024	-1.38
PA1234_at	45.56	40.97	0.484	1.11
PA1235_at	46.86	25.48	0.008	1.84
PA1236_at	5.54	6.95	0.817	-1.25
PA1237_at	21.36	25.45	0.238	-1.19
PA1238_at	13.64	9.13	0.817	1.49
PA1239_at	12.94	14.73	0.643	-1.14
PA1240_at	15.64	14.85	1.000	1.05
PA1241_at	45.14	43.13	0.484	1.05
PA1242_at	11.80	8.77	0.817	1.35
PA1243_at	73.46	63.15	0.151	1.16
PA1244_at	564.76	524.70	0.190	1.08
PA1245_at	87.82	68.18	0.087	1.29
PA1246_aprD_at	31.32	22.13	0.087	1.42
PA1247_aprE_at	42.28	34.95	0.151	1.21
PA1248_aprF_at	47.74	29.67	0.024	1.61
PA1249_aprA_at	17.42	16.48	0.817	1.06
PA1250_aprI_at	55.20	82.85	0.008	-1.50
PA1251_at	16.44	17.38	0.817	-1.06
PA1252_at	25.16	21.70	0.238	1.16
PA1253_at	16.34	18.17	0.484	-1.11
PA1254_at	12.76	10.90	0.484	1.17
PA1255_at	23.88	23.70	0.817	1.01
PA1256_at	8.70	15.55	0.238	-1.79
PA1257_at	5.54	7.32	0.643	-1.32
PA1258_at	11.84	8.88	0.238	1.33
PA1259_at	7.94	7.02	1.000	1.13
PA1260_at	4.58	15.25	0.008	-3.33
PA1261_at	28.84	20.83	0.008	1.38
PA1262_at	8.06	5.50	0.484	1.47
PA1263_at	55.98	53.95	0.817	1.04
PA1264_at	10.84	13.57	0.643	-1.25
PA1265_at	9.14	17.45	0.008	-1.91
PA1266_at	17.08	12.35	0.349	1.38
PA1267_at	5.74	8.25	0.476	-1.44
PA1268_at	11.48	19.47	0.048	-1.70
PA1269_at	74.30	100.32	0.024	-1.35
PA1270_at	10.74	15.30	0.238	-1.42

Supplementary Table 1. Probe set signal data for experimental and control samples

PA1271_at	212.60	188.55	0.484	1.13
PA1272_cobO_at	329.86	404.47	0.048	-1.23
PA1273_cobB_at	124.04	142.98	0.151	-1.15
PA1274_at	107.40	132.13	0.024	-1.23
PA1275_cobD_at	85.12	79.75	0.484	1.07
PA1276_cobC_at	88.44	104.38	0.024	-1.18
PA1277_cobQ_at	101.46	103.98	0.817	-1.02
PA1278_cobP_at	157.14	165.13	0.484	-1.05
PA1279_cobU_at	53.98	90.05	0.008	-1.67
PA1280_at	66.16	72.85	0.349	-1.10
PA1281_cobV_at	43.68	60.92	0.024	-1.39
PA1282_at	11.64	8.60	0.643	1.35
PA1283_at	32.50	37.47	0.484	-1.15
PA1284_at	15.94	6.60	0.087	2.42
PA1285_at	39.66	42.20	0.817	-1.06
PA1286_at	21.22	13.55	0.048	1.57
PA1287_at	35.50	42.60	0.238	-1.20
PA1288_at	2134.64	1963.05	0.349	1.09
PA1289_at	23.98	20.02	0.349	1.20
PA1290_at	32.72	39.45	0.238	-1.21
PA1291_at	24.14	20.40	0.484	1.18
PA1292_at	157.90	129.75	0.087	1.22
PA1293_at	305.38	251.38	0.008	1.21
PA1294_rnd_at	224.36	239.70	0.484	-1.07
PA1295_at	59.38	96.00	0.008	-1.62
PA1296_at	108.54	73.18	0.008	1.48
PA1297_at	26.76	31.05	0.349	-1.16
PA1298_i_at	20.16	9.15	0.048	2.20
PA1299_at	63.94	95.35	0.008	-1.49
PA1300_at	51.96	42.38	0.238	1.23
PA1301_at	24.12	28.67	0.230	-1.19
PA1302_at	14.12	17.13	0.484	-1.21
PA1303_at	19.32	23.50	0.151	-1.22
PA1304_at	62.50	61.93	0.817	1.01
PA1305_at	256.70	243.08	0.643	1.06
PA1306_at	102.72	79.40	0.048	1.29
PA1307_at	165.26	156.52	0.238	1.06
PA1308_at	108.48	83.03	0.016	1.31
PA1309_at	13.58	19.13	0.111	-1.41
PA1310_phnW_at	8.40	7.63	0.905	1.10
PA1311_phnX_at	12.26	9.98	0.643	1.23
PA1312_at	17.40	15.08	0.643	1.15
PA1313_at	8.18	6.78	0.484	1.21
PA1314_at	16.92	27.20	0.238	-1.61
PA1315_at	42.22	35.70	0.238	1.18
PA1316_at	8.30	11.87	0.238	-1.43
PA1317_cyoA_at	70.46	159.85	0.048	-2.27
PA1318_cyoB_at	34.58	81.80	0.008	-2.37
PA1319_cyoC_at	22.46	46.55	0.008	-2.07
PA1320_cyoD_at	8.98	17.05	0.087	-1.90
PA1321_cyoE_at	18.12	33.05	0.048	-1.82

Supplementary Table 1. Probe set signal data for experimental and control samples

PA1322_at	11.32	7.50	0.190	1.51
PA1323_at	122.78	77.15	0.008	1.59
PA1324_at	97.74	87.47	0.349	1.12
PA1325_at	18.88	16.75	0.484	1.13
PA1326_ilvA2_at	45.64	51.70	0.484	-1.13
PA1327_at	9.94	22.13	0.008	-2.23
PA1328_at	9.12	8.53	0.643	1.07
PA1329_at	29.22	28.42	0.643	1.03
PA1330_at	60.08	48.10	0.048	1.25
PA1331_at	65.38	51.50	0.087	1.27
PA1332_at	27.84	12.77	0.024	2.18
PA1333_r_at	138.26	141.00	0.817	-1.02
PA1334_at	19.24	22.50	0.484	-1.17
PA1335_at	87.20	93.40	0.484	-1.07
PA1336_at	190.04	169.82	0.087	1.12
PA1337_ansB_at	993.76	809.42	0.087	1.23
PA1338_ggt_at	916.20	635.30	0.048	1.44
PA1339_at	1394.56	1132.80	0.048	1.23
PA1340_at	993.86	687.18	0.048	1.45
PA1341_at	920.80	784.73	0.151	1.17
PA1342_at	3897.76	2791.82	0.008	1.40
PA1343_at	119.66	111.10	0.484	1.08
PA1344_at	46.38	48.28	0.349	-1.04
PA1345_at	10.48	8.88	0.817	1.18
PA1346_at	3.86	1.67	0.016	2.31
PA1347_at	9.78	10.40	1.000	-1.06
PA1348_at	17.36	12.40	0.190	1.40
PA1349_at	21.04	14.30	0.349	1.47
PA1350_at	16.98	15.83	0.484	1.07
PA1351_at	12.18	14.10	0.349	-1.16
PA1352_at	19.74	14.52	0.238	1.36
PA1353_at	17.52	16.63	0.643	1.05
PA1354_at	41.82	25.70	0.024	1.63
PA1355_at	1.36	3.32	0.341	-2.44
PA1356_at	30.70	19.35	0.087	1.59
PA1357_at	41.02	55.83	0.087	-1.36
PA1358_at	21.14	17.80	0.349	1.19
PA1359_at	23.20	26.82	0.349	-1.16
PA1360_at	23.22	32.18	0.151	-1.39
PA1361_at	21.02	22.20	0.238	-1.06
PA1362_i_at	25.48	32.90	0.238	-1.29
PA1363_at	48.10	71.32	0.008	-1.48
PA1364_at	23.04	16.10	0.048	1.43
PA1365_at	100.86	63.30	0.016	1.59
PA1366_at	23.84	33.25	0.048	-1.39
PA1367_at	26.00	33.85	0.087	-1.30
PA1368_at	18.30	24.63	0.111	-1.35
PA1369_at	13.66	11.32	1.000	1.21
PA1370_at	31.18	27.15	0.484	1.15
PA1371_at	9.50	20.60	0.024	-2.17
PA1372_at	22.86	48.13	0.008	-2.11

Supplementary Table 1. Probe set signal data for experimental and control samples

PA1373_fabF2_at	56.18	67.40	0.087	-1.20
PA1374_at	26.58	26.38	0.817	1.01
PA1375_pdxB_at	205.60	143.00	0.008	1.44
PA1376_aceK_at	124.70	82.78	0.008	1.51
PA1377_at	84.12	82.63	0.905	1.02
PA1378_at	27.88	15.95	0.008	1.75
PA1379_at	8.98	14.52	0.024	-1.62
PA1380_at	5.48	9.77	0.151	-1.78
PA1381_at	4.72	6.50	0.238	-1.38
PA1382_at	16.40	19.95	0.087	-1.22
PA1383_at	27.30	33.90	0.079	-1.24
PA1384_galE_at	5.26	2.75	0.643	1.91
PA1385_at	1.24	4.47	0.087	-3.60
PA1386_at	11.62	4.22	0.048	2.75
PA1387_at	15.74	15.13	0.484	1.04
PA1388_at	11.52	14.40	0.341	-1.25
PA1389_at	10.30	12.15	0.341	-1.18
PA1390_at	13.34	8.65	0.286	1.54
PA1391_at	20.80	9.93	0.024	2.09
PA1392_at	8.48	9.73	0.484	-1.15
PA1393_cysC_at	1.34	5.75	0.008	-4.29
PA1394_at	63.32	90.65	0.048	-1.43
PA1395_at	62.72	81.78	0.151	-1.30
PA1396_at	27.76	45.95	0.008	-1.66
PA1397_at	65.96	64.57	0.151	1.02
PA1398_at	48.90	58.18	0.048	-1.19
PA1399_at	24.72	21.42	1.000	1.15
PA1400_at	21.00	17.10	0.238	1.23
PA1401_at	30.36	42.60	0.151	-1.40
PA1402_at	26.84	23.43	0.484	1.15
PA1403_at	19.20	18.82	0.643	1.02
PA1404_at	77.06	49.68	0.024	1.55
PA1405_at	29.02	25.97	0.349	1.12
PA1406_at	11.84	11.32	0.817	1.05
PA1407_at	59.56	68.85	0.817	-1.16
PA1408_at	39.58	19.08	0.008	2.07
PA1409_aphA_at	97.70	54.35	0.008	1.80
PA1410_at	68.56	42.43	0.008	1.62
PA1411_at	59.84	51.45	0.087	1.16
PA1412_at	7.94	10.68	0.238	-1.35
PA1413_at	22.52	28.88	0.151	-1.28
PA1414_at	103.24	303.30	0.024	-2.94
PA1415_at	21.66	20.05	0.643	1.08
PA1416_at	20.28	18.07	0.484	1.12
PA1417_at	39.36	45.52	0.484	-1.16
PA1418_at	38.84	41.38	0.817	-1.07
PA1419_at	12.32	15.17	0.730	-1.23
PA1420_at	28.66	31.43	0.817	-1.10
PA1421_speB2_at	41.10	61.72	0.484	-1.50
PA1422_at	95.44	102.70	0.643	-1.08
PA1423_at	29.40	29.65	1.000	-1.01

Supplementary Table 1. Probe set signal data for experimental and control samples

PA1424_at	21.06	15.00	0.349	1.40
PA1425_at	20.48	19.05	0.643	1.08
PA1426_at	12.90	8.93	0.484	1.44
PA1427_at	14.72	18.60	0.484	-1.26
PA1428_at	8.84	5.48	0.190	1.61
PA1429_at	40.06	43.53	0.151	-1.09
PA1430_lasR_at	135.98	123.55	0.817	1.10
PA1431_rsaL_at	327.98	414.42	0.484	-1.26
PA1432_lasI_at	217.34	439.25	0.008	-2.02
PA1433_at	25.02	29.42	0.349	-1.18
PA1434_at	34.82	30.33	0.238	1.15
PA1435_at	28.56	18.35	0.151	1.56
PA1436_at	21.14	17.20	0.643	1.23
PA1437_at	23.86	25.77	0.643	-1.08
PA1438_at	34.60	24.77	0.048	1.40
PA1439_at	60.22	56.13	0.817	1.07
PA1440_at	709.08	656.28	0.484	1.08
PA1441_at	377.86	350.95	0.048	1.08
PA1442_at	278.80	324.83	0.024	-1.17
PA1443_fliM_at	241.38	269.63	0.151	-1.12
PA1444_fliN_at	80.24	88.15	0.349	-1.10
PA1445_fliO_at	59.62	79.63	0.048	-1.34
PA1446_fliP_at	81.90	75.03	0.238	1.09
PA1447_fliQ_at	51.08	66.88	0.024	-1.31
PA1448_fliR_at	24.92	31.65	0.151	-1.27
PA1449_flhB_at	17.54	17.33	0.817	1.01
PA1450_at	28.60	23.10	0.151	1.24
PA1451_at	39.18	43.28	0.151	-1.10
PA1452_flhA_at	90.08	76.57	0.349	1.18
PA1453_flhF_at	332.14	212.05	0.008	1.57
PA1454_at	654.46	471.55	0.008	1.39
PA1455_fliA_at	390.62	306.15	0.008	1.28
PA1456_cheY_at	347.40	420.52	0.024	-1.21
PA1457_cheZ_at	334.14	421.58	0.008	-1.26
PA1458_at	321.66	335.65	0.238	-1.04
PA1459_at	414.98	356.83	0.238	1.16
PA1460_at	117.82	100.28	0.087	1.17
PA1461_at	78.54	58.10	0.024	1.35
PA1462_at	260.56	218.63	0.024	1.19
PA1463_at	202.58	144.20	0.008	1.40
PA1464_at	599.74	662.70	0.087	-1.10
PA1465_at	91.98	99.05	0.143	-1.08
PA1466_at	277.02	17.27	0.008	16.04
PA1467_at	59.32	58.50	0.817	1.01
PA1468_at	19.22	21.83	0.238	-1.14
PA1469_at	34.94	18.17	0.008	1.92
PA1470_at	15.20	17.30	0.484	-1.14
PA1471_at	56.38	44.90	0.151	1.26
PA1472_at	57.02	49.50	0.349	1.15
PA1473_at	44.98	48.95	0.484	-1.09
PA1474_at	132.04	123.97	0.484	1.07

Supplementary Table 1. Probe set signal data for experimental and control samples

PA1475_ccmA_at	143.90	133.43	0.238	1.08
PA1476_ccmB_at	78.68	64.30	0.048	1.22
PA1477_ccmC_at	49.68	69.45	0.008	-1.40
PA1478_at	183.74	205.10	0.484	-1.12
PA1479_ccmE_at	314.64	379.00	0.048	-1.20
PA1480_ccmF_at	131.74	167.00	0.008	-1.27
PA1481_ccmG_at	120.08	120.50	0.643	-1.00
PA1482_ccmH_at	247.24	319.75	0.024	-1.29
PA1483_cycH_at	140.58	150.48	0.238	-1.07
PA1484_at	32.90	24.52	0.008	1.34
PA1485_at	15.10	14.50	0.817	1.04
PA1486_at	10.14	13.95	0.484	-1.38
PA1487_at	46.14	53.15	0.484	-1.15
PA1488_at	48.86	42.28	0.238	1.16
PA1489_at	35.92	32.72	0.484	1.10
PA1490_at	38.84	41.78	0.817	-1.08
PA1491_at	10.36	9.52	0.817	1.09
PA1492_at	9.26	22.25	0.087	-2.40
PA1493_cysP_at	836.38	765.10	0.087	1.09
PA1494_at	300.32	254.65	0.151	1.18
PA1495_at	60.86	53.35	0.484	1.14
PA1496_at	20.76	20.97	0.643	-1.01
PA1497_at	12.80	11.43	0.643	1.12
PA1498_pykF_at	12.10	10.10	0.484	1.20
PA1499_at	23.50	30.72	0.048	-1.31
PA1500_at	20.10	26.35	0.048	-1.31
PA1501_at	11.00	16.55	0.087	-1.50
PA1502_gcl_at	12.22	27.23	0.024	-2.23
PA1503_at	2.32	10.68	0.024	-4.60
PA1504_at	241.10	300.22	0.008	-1.25
PA1505_moaA2_at	137.26	116.13	0.238	1.18
PA1506_at	30.28	29.55	0.817	1.02
PA1507_at	43.48	22.63	0.008	1.92
PA1508_at	23.78	23.00	0.817	1.03
PA1509_at	25.24	28.35	0.349	-1.12
PA1510_at	13.22	13.02	0.817	1.02
PA1511_at	39.26	40.67	0.817	-1.04
PA1513_at	68.54	56.28	0.151	1.22
PA1514_at	30.00	49.75	0.008	-1.66
PA1515_alc_at	44.80	56.50	0.238	-1.26
PA1516_at	38.16	36.27	0.817	1.05
PA1517_at	144.72	130.80	0.643	1.11
PA1518_at	32.78	44.80	0.048	-1.37
PA1519_at	11.96	4.75	0.238	2.52
PA1520_at	183.06	227.98	0.008	-1.25
PA1521_at	53.00	63.55	0.238	-1.20
PA1522_at	61.78	56.95	0.817	1.08
PA1523_xdhB_at	20.26	22.73	0.817	-1.12
PA1524_xdhA_at	39.06	32.53	0.349	1.20
PA1525_at	20.04	14.13	0.143	1.42
PA1526_at	118.86	129.73	0.349	-1.09

Supplementary Table 1. Probe set signal data for experimental and control samples

PA1527_at	246.68	209.70	0.024	1.18
PA1528_zipA_at	633.86	662.55	0.484	-1.05
PA1529_lig_at	104.38	129.23	0.008	-1.24
PA1530_at	54.08	94.65	0.008	-1.75
PA1531_at	3.76	8.35	0.143	-2.22
PA1532_dnaX_at	558.36	408.75	0.008	1.37
PA1533_at	624.88	666.00	0.151	-1.07
PA1534_recR_at	85.54	68.20	0.087	1.25
PA1535_at	47.00	37.25	0.048	1.26
PA1536_at	26.56	19.05	0.048	1.39
PA1537_at	21.74	8.63	0.024	2.52
PA1538_at	25.66	22.78	0.817	1.13
PA1539_at	34.38	42.20	0.151	-1.23
PA1540_at	11.38	8.10	0.238	1.40
PA1541_at	5.70	1.88	0.111	3.03
PA1542_at	10.76	15.65	0.238	-1.45
PA1543_apt_at	270.36	290.63	0.151	-1.07
PA1544_anr_at	778.80	865.85	0.413	-1.11
PA1545_at	100.90	114.25	0.484	-1.13
PA1546_hemN_at	131.58	181.18	0.151	-1.38
PA1547_at	44.12	33.53	0.087	1.32
PA1548_at	40.08	31.17	0.413	1.29
PA1549_at	45.72	53.15	0.484	-1.16
PA1550_at	135.58	194.98	0.048	-1.44
PA1551_at	112.74	127.80	0.349	-1.13
PA1552_at	729.48	1023.95	0.008	-1.40
PA1553_at	1532.64	1918.82	0.087	-1.25
PA1554_at	2379.04	2795.95	0.151	-1.18
PA1555_at	329.42	394.48	0.484	-1.20
PA1556_at	332.44	547.17	0.238	-1.65
PA1557_at	288.40	486.55	0.238	-1.69
PA1558_at	49.36	38.40	0.048	1.29
PA1559_at	225.68	159.43	0.151	1.42
PA1560_at	79.86	70.45	0.349	1.13
PA1561_aer_at	214.64	338.77	0.024	-1.58
PA1562_acnA_at	40.80	33.47	0.063	1.22
PA1563_at	51.38	61.65	0.087	-1.20
PA1564_at	91.22	141.60	0.008	-1.55
PA1565_at	35.06	39.60	0.730	-1.13
PA1566_at	35.98	42.75	0.484	-1.19
PA1567_at	10.80	9.33	0.643	1.16
PA1568_at	5.18	12.32	0.238	-2.38
PA1569_at	9.88	2.85	0.024	3.47
PA1570_at	31.62	30.95	0.643	1.02
PA1571_at	72.80	69.65	0.468	1.05
PA1572_at	75.96	62.30	0.151	1.22
PA1573_at	62.42	48.55	0.024	1.29
PA1574_at	112.52	268.60	0.008	-2.39
PA1575_at	16.78	19.72	0.413	-1.18
PA1576_at	47.02	31.28	0.008	1.50
PA1577_at	32.02	45.42	0.024	-1.42

Supplementary Table 1. Probe set signal data for experimental and control samples

PA1578_at	14.76	13.22	0.643	1.12
PA1579_at	429.48	409.17	0.817	1.05
PA1580_gltA_at	2277.70	2253.77	0.817	1.01
PA1581_sdhC_at	1863.34	2079.05	0.087	-1.12
PA1582_sdhD_at	2547.78	2420.17	0.643	1.05
PA1583_sdhA_at	1993.26	2014.45	0.151	-1.01
PA1584_sdhB_at	1555.30	1819.38	0.151	-1.17
PA1585_sucA_at	1174.80	1086.13	0.643	1.08
PA1586_sucB_at	2924.20	2385.45	0.048	1.23
PA1587_lpdG_at	1084.46	1171.65	0.484	-1.08
PA1588_sucC_at	4972.62	4064.10	0.008	1.22
PA1589_sucD_at	3158.34	3620.13	0.087	-1.15
PA1590_braB_at	84.52	90.23	0.817	-1.07
PA1591_at	33.72	48.63	0.008	-1.44
PA1592_i_at	716.62	1529.30	0.008	-2.13
PA1593_at	98.94	110.78	0.238	-1.12
PA1594_at	47.06	56.17	0.087	-1.19
PA1595_at	33.42	32.60	1.000	1.03
PA1596_htpG_at	880.12	1252.07	0.008	-1.42
PA1597_at	62.38	52.10	0.238	1.20
PA1598_at	4.18	6.13	0.817	-1.47
PA1599_at	23.62	17.08	0.024	1.38
PA1600_at	45.94	43.08	0.817	1.07
PA1601_at	38.06	38.58	1.000	-1.01
PA1602_at	49.34	54.30	0.238	-1.10
PA1603_at	42.44	35.65	0.349	1.19
PA1604_at	55.14	48.25	0.492	1.14
PA1605_at	25.94	20.80	0.349	1.25
PA1606_at	17.88	27.77	0.024	-1.55
PA1607_at	44.36	43.60	0.643	1.02
PA1608_at	82.16	76.27	0.817	1.08
PA1609_fabB_at	485.60	752.25	0.008	-1.55
PA1610_fabA_at	864.70	1337.43	0.024	-1.55
PA1611_at	52.86	63.88	0.008	-1.21
PA1612_at	50.10	56.92	0.151	-1.14
PA1613_at	84.84	64.30	0.087	1.32
PA1614_gpsA_at	310.56	308.63	1.000	1.01
PA1615_at	124.90	142.70	0.151	-1.14
PA1616_at	147.50	174.43	0.349	-1.18
PA1617_at	39.36	21.63	0.008	1.82
PA1618_at	165.26	96.97	0.008	1.70
PA1619_at	64.00	59.55	0.643	1.07
PA1620_at	18.16	23.63	0.556	-1.30
PA1621_at	129.50	92.88	0.008	1.39
PA1622_at	49.26	49.60	1.000	-1.01
PA1623_at	97.22	89.73	0.484	1.08
PA1624_at	89.32	64.40	0.151	1.39
PA1625_at	17.98	14.05	0.238	1.28
PA1626_at	31.86	41.38	0.079	-1.30
PA1627_at	52.70	54.28	0.817	-1.03
PA1628_at	12.96	16.10	1.000	-1.24

Supplementary Table 1. Probe set signal data for experimental and control samples

PA1629_at	29.42	29.25	0.730	1.01
PA1630_at	43.44	45.72	0.643	-1.05
PA1631_at	9.70	13.05	0.484	-1.35
PA1632_kdpF_at	11.28	32.02	0.008	-2.84
PA1633_kdpA_at	19.44	45.63	0.008	-2.35
PA1634_kdpB_at	25.72	58.48	0.008	-2.27
PA1635_kdpC_at	18.28	34.53	0.024	-1.89
PA1636_kdpD_at	21.22	27.58	0.048	-1.30
PA1637_kdpE_at	14.54	9.73	0.151	1.49
PA1638_at	50.00	68.45	0.008	-1.37
PA1639_at	74.44	93.10	0.151	-1.25
PA1640_at	137.70	86.28	0.008	1.60
PA1641_at	60.50	54.83	0.349	1.10
PA1642_selD_at	148.64	146.00	0.817	1.02
PA1643_at	40.52	65.82	0.008	-1.62
PA1644_at	92.80	83.05	0.151	1.12
PA1645_at	58.22	53.53	0.484	1.09
PA1646_at	19.10	18.50	0.643	1.03
PA1647_at	28.22	26.03	0.643	1.08
PA1648_at	11.66	11.27	0.643	1.03
PA1649_at	18.34	18.60	0.730	-1.01
PA1650_at	17.44	9.50	0.151	1.84
PA1651_at	56.96	46.10	0.008	1.24
PA1652_at	42.48	26.72	0.008	1.59
PA1653_at	41.30	36.42	0.643	1.13
PA1654_at	268.14	166.45	0.008	1.61
PA1655_at	69.34	58.27	0.238	1.19
PA1656_at	121.48	103.63	0.238	1.17
PA1657_at	166.72	255.63	0.087	-1.53
PA1658_at	124.30	176.70	0.048	-1.42
PA1659_at	74.16	123.05	0.024	-1.66
PA1660_at	46.70	54.25	0.349	-1.16
PA1661_at	48.82	58.88	0.087	-1.21
PA1662_at	49.62	56.53	0.349	-1.14
PA1663_at	85.52	90.77	0.817	-1.06
PA1664_at	122.10	141.73	0.643	-1.16
PA1665_at	49.80	83.43	0.008	-1.68
PA1666_at	43.34	73.07	0.008	-1.69
PA1667_at	62.22	106.15	0.048	-1.71
PA1668_at	41.20	61.35	0.087	-1.49
PA1669_at	43.72	63.35	0.238	-1.45
PA1670_stp1_at	42.24	42.70	1.000	-1.01
PA1671_stk1_at	21.18	21.95	1.000	-1.04
PA1672_at	18.04	13.82	0.349	1.31
PA1673_at	93.12	181.40	0.048	-1.95
PA1674_folE2_at	145.92	280.55	0.008	-1.92
PA1675_at	140.22	150.70	0.349	-1.07
PA1676_at	74.46	77.43	0.643	-1.04
PA1677_at	420.20	451.35	0.238	-1.07
PA1678_at	117.50	146.98	0.024	-1.25
PA1679_at	93.20	68.00	0.008	1.37

PA1680_at	38.56	25.75	0.008	1.50
PA1681_aroC_at	190.54	236.25	0.024	-1.24
PA1682_at	36.00	33.60	0.484	1.07
PA1683_at	117.82	107.02	0.143	1.10
PA1684_at	199.40	219.60	0.238	-1.10
PA1685_masA_at	94.46	99.23	0.643	-1.05
PA1686_alkA_at	88.50	68.48	0.048	1.29
PA1687_speE_at	77.78	204.80	0.008	-2.63
PA1688_at	165.30	145.35	0.151	1.14
PA1689_at	132.68	155.50	0.087	-1.17
PA1690_pscU_at	17.72	12.17	0.048	1.46
PA1691_pscT_at	31.72	21.73	0.087	1.46
PA1692_at	72.18	59.00	0.151	1.22
PA1693_pscR_at	33.58	32.57	0.556	1.03
PA1694_pscQ_at	102.66	89.55	0.238	1.15
PA1695_pscP_at	44.04	32.72	0.087	1.35
PA1696_pscO_at	54.16	36.15	0.087	1.50
PA1697_at	96.90	63.60	0.008	1.52
PA1698_popN_at	42.16	38.08	0.484	1.11
PA1699_at	34.50	32.80	0.643	1.05
PA1700_at	62.84	62.10	0.556	1.01
PA1701_at	92.76	73.72	0.048	1.26
PA1702_i_at	11.00	17.60	0.238	-1.60
PA1703_pcrD_at	48.56	41.90	0.643	1.16
PA1704_pcrR_at	17.92	18.77	1.000	-1.05
PA1705_pcrG_i_at	42.42	34.78	0.238	1.22
PA1706_pcrV_at	48.54	47.83	0.817	1.01
PA1707_pcrH_at	52.98	64.47	0.087	-1.22
PA1708_popB_at	128.20	123.07	0.151	1.04
PA1709_popD_at	52.88	67.90	0.024	-1.28
PA1710_exsC_at	178.78	164.55	0.643	1.09
PA1711_at	257.48	162.38	0.008	1.59
PA1712_exsB_at	56.04	61.80	0.484	-1.10
PA1713_exsA_at	75.58	73.17	0.484	1.03
PA1714_at	155.08	119.97	0.087	1.29
PA1715_pscB_at	27.06	20.98	0.151	1.29
PA1716_pscC_at	91.58	70.20	0.484	1.30
PA1717_pscD_at	46.26	37.42	0.349	1.24
PA1718_pscE_at	75.72	128.42	0.008	-1.70
PA1719_pscF_at	57.94	91.28	0.048	-1.58
PA1720_pscG_at	44.32	53.10	0.087	-1.20
PA1721_pscH_at	36.18	39.67	0.643	-1.10
PA1722_pscI_at	92.58	91.38	0.643	1.01
PA1723_pscJ_at	42.26	47.07	0.349	-1.11
PA1724_pscK_at	38.60	37.92	1.000	1.02
PA1725_pscL_at	16.46	21.38	0.087	-1.30
PA1726_bglX_at	341.14	257.55	0.024	1.32
PA1727_at	89.06	82.40	0.151	1.08
PA1728_at	31.84	29.13	0.484	1.09
PA1729_at	46.94	39.33	0.151	1.19
PA1730_at	18.88	24.27	0.151	-1.29

Supplementary Table 1. Probe set signal data for experimental and control samples

PA1731_at	26.92	24.13	0.817	1.12
PA1732_at	39.00	31.40	0.349	1.24
PA1733_at	33.22	37.42	0.905	-1.13
PA1734_at	47.84	32.80	0.008	1.46
PA1735_at	17.50	20.40	0.341	-1.17
PA1736_at	20.26	21.05	0.643	-1.04
PA1737_at	44.46	29.05	0.008	1.53
PA1738_at	37.74	26.15	0.151	1.44
PA1739_at	11.64	15.20	0.349	-1.31
PA1740_at	12.44	4.23	0.024	2.94
PA1741_at	48.54	87.13	0.008	-1.80
PA1742_at	169.08	132.98	0.008	1.27
PA1743_at	3.66	9.83	0.024	-2.69
PA1744_at	14.04	10.02	0.238	1.40
PA1745_at	14.24	19.00	0.484	-1.33
PA1746_at	23.72	38.60	0.048	-1.63
PA1747_at	15.28	22.05	0.238	-1.44
PA1748_at	128.70	138.20	0.643	-1.07
PA1749_at	75.74	85.40	0.151	-1.13
PA1750_at	455.28	445.85	0.817	1.02
PA1751_at	50.72	38.23	0.024	1.33
PA1752_at	147.50	109.65	0.008	1.35
PA1753_at	52.62	62.82	0.151	-1.19
PA1754_cysB_at	348.70	407.03	0.008	-1.17
PA1755_at	32.72	39.10	0.087	-1.19
PA1756_cysH_at	83.30	126.63	0.048	-1.52
PA1757_thrH_at	304.10	359.55	0.087	-1.18
PA1758_pabB_at	57.72	61.42	0.484	-1.06
PA1759_at	181.54	77.60	0.008	2.34
PA1760_at	143.22	51.27	0.008	2.79
PA1761_at	173.78	120.92	0.008	1.44
PA1762_at	34.18	14.22	0.008	2.40
PA1763_at	32.80	20.00	0.008	1.64
PA1764_at	7.02	7.53	1.000	-1.07
PA1765_at	13.72	13.70	0.905	1.00
PA1766_at	200.62	267.23	0.024	-1.33
PA1767_at	274.42	401.25	0.008	-1.46
PA1768_at	576.64	667.10	0.087	-1.16
PA1769_at	91.64	151.32	0.008	-1.65
PA1770_ppsA_at	867.68	937.72	0.238	-1.08
PA1771_at	57.68	69.65	0.238	-1.21
PA1772_at	185.58	209.63	0.151	-1.13
PA1773_at	74.10	84.52	0.151	-1.14
PA1774_at	93.90	108.82	0.048	-1.16
PA1775_at	153.42	161.18	0.349	-1.05
PA1776_at	358.12	722.53	0.008	-2.02
PA1777_oprF_at	1752.26	1914.50	0.643	-1.09
PA1778_cobA_at	1.32	5.25	0.008	-3.98
PA1779_at	4.74	4.22	0.484	1.12
PA1780_nirD_at	6.14	8.98	0.349	-1.46
PA1781_nirB_at	2.16	3.13	0.222	-1.45

Supplementary Table 1. Probe set signal data for experimental and control samples

PA1782_at	23.62	16.90	0.048	1.40
PA1783_nasA_at	8.50	4.55	0.151	1.87
PA1784_at	34.26	29.55	0.484	1.16
PA1785_at	4.10	7.28	0.151	-1.78
PA1786_at	23.60	23.90	0.817	-1.01
PA1787_acnB_at	1908.38	1854.45	1.000	1.03
PA1788_at	44.92	59.88	0.087	-1.33
PA1789_at	101.32	102.40	1.000	-1.01
PA1790_at	26.20	75.47	0.008	-2.88
PA1791_at	337.36	361.67	0.151	-1.07
PA1792_at	538.00	363.02	0.008	1.48
PA1793_ppiB_at	822.76	1207.43	0.048	-1.47
PA1794_glnS_at	604.10	632.57	0.484	-1.05
PA1795_cysS_at	468.84	473.77	0.643	-1.01
PA1796_folD_at	373.84	463.25	0.024	-1.24
PA1797_at	27.78	16.15	0.024	1.72
PA1798_at	87.16	90.57	0.817	-1.04
PA1799_at	285.10	300.05	0.643	-1.05
PA1800_tig_at	1822.92	1921.43	0.484	-1.05
PA1801_clpP_at	1149.28	1265.93	0.087	-1.10
PA1802_clpX_at	712.98	961.65	0.008	-1.35
PA1803_lon_at	359.38	515.08	0.024	-1.43
PA1804_hupB_at	1187.16	1279.38	0.643	-1.08
PA1805_ppiD_at	563.94	657.17	0.008	-1.17
PA1806_fabI_at	290.50	152.85	0.008	1.90
PA1807_at	103.44	111.03	0.817	-1.07
PA1808_at	126.84	87.72	0.008	1.45
PA1809_at	88.54	79.65	0.349	1.11
PA1810_at	40.92	37.90	0.817	1.08
PA1811_at	65.68	69.93	0.484	-1.06
PA1812_mltD_at	755.06	731.03	0.643	1.03
PA1813_at	177.08	152.30	0.349	1.16
PA1814_at	155.08	121.68	0.024	1.27
PA1815_rnhA_at	190.22	226.25	0.024	-1.19
PA1816_dnaQ_at	149.64	149.77	0.643	-1.00
PA1817_at	7.60	10.18	0.151	-1.34
PA1818_at	942.34	516.90	0.008	1.82
PA1819_at	69.20	45.47	0.008	1.52
PA1820_nhaB_at	38.76	26.90	0.008	1.44
PA1821_at	278.02	291.67	0.817	-1.05
PA1822_at	137.12	119.38	0.484	1.15
PA1823_at	103.48	117.68	0.151	-1.14
PA1824_at	26.66	30.10	0.484	-1.13
PA1825_at	30.84	27.80	0.817	1.11
PA1826_at	20.42	12.35	0.087	1.65
PA1827_at	18.32	20.38	0.817	-1.11
PA1828_at	77.32	65.55	0.151	1.18
PA1829_at	140.70	101.10	0.008	1.39
PA1830_at	146.84	218.90	0.024	-1.49
PA1831_at	46.94	51.65	0.817	-1.10
PA1832_at	64.44	106.63	0.008	-1.65

Supplementary Table 1. Probe set signal data for experimental and control samples

PA1833_at	93.14	87.93	0.349	1.06
PA1834_at	20.20	22.48	0.349	-1.11
PA1835_at	61.06	64.40	0.817	-1.05
PA1836_at	11.10	23.62	0.008	-2.13
PA1837_at	188.30	386.17	0.008	-2.05
PA1838_cysI_at	328.54	582.15	0.008	-1.77
PA1839_at	33.92	56.65	0.008	-1.67
PA1840_at	98.96	132.03	0.048	-1.33
PA1841_at	39.66	49.63	0.087	-1.25
PA1842_at	41.22	50.85	0.151	-1.23
PA1843_metH_at	80.12	75.55	0.484	1.06
PA1844_at	25.56	27.63	0.484	-1.08
PA1845_at	36.78	83.60	0.008	-2.27
PA1846_cti_at	74.50	76.25	0.635	-1.02
PA1847_at	232.44	290.17	0.087	-1.25
PA1848_at	9.38	8.07	0.643	1.16
PA1849_at	2.24	6.60	0.008	-2.95
PA1850_at	37.98	24.40	0.024	1.56
PA1851_at	12.54	11.80	0.635	1.06
PA1852_at	117.92	239.65	0.048	-2.03
PA1853_at	78.32	75.40	0.817	1.04
PA1854_at	3.40	17.52	0.008	-5.15
PA1855_r_at	10.72	7.20	0.643	1.49
PA1856_at	12.48	8.13	0.484	1.54
PA1857_at	198.82	202.20	0.643	-1.02
PA1858_str_at	42.12	40.20	0.817	1.05
PA1859_at	41.18	33.50	0.151	1.23
PA1860_at	17.14	18.40	0.817	-1.07
PA1861_modC_at	65.08	44.72	0.008	1.46
PA1862_modB_at	78.56	73.45	0.349	1.07
PA1863_modA_at	91.46	83.63	0.238	1.09
PA1864_at	18.94	13.60	0.349	1.39
PA1865_at	120.46	38.72	0.008	3.11
PA1866_at	73.60	47.57	0.048	1.55
PA1867_at	13.40	9.93	0.151	1.35
PA1868_xqhA_at	26.78	33.08	0.349	-1.24
PA1869_at	171.24	203.48	0.349	-1.19
PA1870_at	13.12	15.40	0.643	-1.17
PA1871_lasA_at	36.88	24.90	0.349	1.48
PA1872_at	50.86	33.25	0.008	1.53
PA1873_at	18.50	20.77	1.000	-1.12
PA1874_at	52.56	32.50	0.024	1.62
PA1875_at	7.54	10.60	0.349	-1.41
PA1876_at	8.28	13.38	0.349	-1.62
PA1877_at	16.14	19.13	0.817	-1.19
PA1878_at	35.24	44.83	0.238	-1.27
PA1879_at	27.54	30.58	1.000	-1.11
PA1880_at	36.40	36.42	0.817	-1.00
PA1881_at	56.96	27.90	0.008	2.04
PA1882_at	26.84	14.02	0.024	1.91
PA1883_at	18.40	15.50	0.817	1.19

Supplementary Table 1. Probe set signal data for experimental and control samples

PA1884_at	16.34	19.33	0.151	-1.18
PA1885_at	26.60	20.13	0.238	1.32
PA1886_polB_at	37.12	27.90	0.016	1.33
PA1887_at	8.02	2.82	0.087	2.84
PA1888_at	3.70	12.10	0.008	-3.27
PA1889_at	25.32	21.50	0.151	1.18
PA1890_at	49.54	56.35	0.238	-1.14
PA1891_at	16.64	20.00	0.484	-1.20
PA1892_at	20.94	20.65	1.000	1.01
PA1893_at	53.78	49.38	0.817	1.09
PA1894_at	112.60	119.52	0.643	-1.06
PA1895_at	99.08	105.10	0.349	-1.06
PA1896_at	62.86	78.72	0.643	-1.25
PA1897_at	118.20	129.55	0.484	-1.10
PA1898_at	41.88	29.55	0.024	1.42
PA1901_s_at	111.12	90.10	0.151	1.23
PA1902_s_at	13.78	18.58	0.349	-1.35
PA1903_s_at	66.56	78.45	0.238	-1.18
PA1904_s_at	24.34	28.63	0.484	-1.18
PA1905_s_at	67.38	57.47	0.484	1.17
PA1906_at	31.46	21.90	0.087	1.44
PA1907_at	11.90	6.65	0.151	1.79
PA1908_at	7.88	8.05	0.349	-1.02
PA1909_at	7.94	12.88	0.238	-1.62
PA1910_at	24.46	16.95	0.143	1.44
PA1911_at	24.96	21.60	0.643	1.16
PA1912_at	42.56	36.53	0.349	1.17
PA1913_at	120.12	122.53	0.817	-1.02
PA1914_at	7.30	7.53	0.817	-1.03
PA1915_at	16.74	11.50	0.238	1.46
PA1916_at	4.42	8.45	0.349	-1.91
PA1917_at	5.58	6.15	1.000	-1.10
PA1918_at	12.44	14.22	0.635	-1.14
PA1919_at	6.22	2.92	0.238	2.13
PA1920_at	23.50	24.42	1.000	-1.04
PA1921_at	3.44	6.27	0.151	-1.82
PA1922_at	13.22	7.38	0.484	1.79
PA1923_at	14.66	19.67	0.484	-1.34
PA1924_at	11.36	11.88	0.817	-1.05
PA1925_at	14.80	14.88	0.643	-1.01
PA1926_at	66.62	83.85	0.024	-1.26
PA1927_metE_at	6.14	9.47	0.238	-1.54
PA1928_rimJ_at	30.56	49.25	0.048	-1.61
PA1929_i_at	29.40	24.32	0.484	1.21
PA1930_at	12.98	13.15	0.905	-1.01
PA1931_at	20.78	16.25	0.484	1.28
PA1932_at	18.80	20.25	0.643	-1.08
PA1933_at	19.36	17.20	0.349	1.13
PA1934_at	34.58	38.52	0.151	-1.11
PA1935_at	18.72	12.48	0.008	1.50
PA1936_at	7.88	9.98	0.730	-1.27

Supplementary Table 1. Probe set signal data for experimental and control samples

PA1939_at	70.86	87.17	0.151	-1.23
PA1940_at	100.16	93.03	0.238	1.08
PA1941_at	152.84	149.68	0.817	1.02
PA1942_at	7.18	15.45	0.151	-2.15
PA1943_at	37.42	42.60	0.349	-1.14
PA1944_at	30.48	30.52	1.000	-1.00
PA1945_at	23.24	18.92	0.238	1.23
PA1946_rbsB_at	159.68	58.97	0.008	2.71
PA1947_rbsA_at	60.90	33.47	0.048	1.82
PA1948_rbsC_at	56.58	49.50	0.413	1.14
PA1949_rbsR_at	193.04	111.63	0.008	1.73
PA1950_rbsK_at	125.80	80.22	0.024	1.57
PA1951_at	3.88	5.83	0.151	-1.50
PA1952_at	13.70	9.23	0.643	1.48
PA1953_at	2.16	1.45	0.048	1.49
PA1954_at	16.24	17.00	1.000	-1.05
PA1955_at	10.28	5.28	0.286	1.95
PA1956_at	10.60	11.70	0.817	-1.10
PA1957_at	30.28	29.58	0.643	1.02
PA1958_at	60.70	64.72	0.286	-1.07
PA1959_bacA_at	89.00	119.53	0.151	-1.34
PA1960_at	29.78	29.07	0.817	1.02
PA1961_at	7.38	17.60	0.008	-2.38
PA1962_at	8.08	11.15	0.905	-1.38
PA1963_at	29.08	31.05	0.817	-1.07
PA1964_at	180.36	219.18	0.349	-1.22
PA1965_at	44.64	59.32	0.008	-1.33
PA1966_at	15.70	59.38	0.008	-3.78
PA1967_at	50.02	73.00	0.008	-1.46
PA1968_at	77.82	92.28	0.048	-1.19
PA1969_at	193.24	150.52	0.151	1.28
PA1970_at	10.52	18.20	0.151	-1.73
PA1971_braZ_at	8.90	40.60	0.008	-4.56
PA1972_at	2.30	0.85	0.063	2.71
PA1973_pqqF_at	33.30	25.90	0.008	1.29
PA1974_at	15.16	9.00	0.349	1.68
PA1975_at	24.72	22.58	0.341	1.09
PA1976_at	16.78	5.55	0.048	3.02
PA1977_at	19.28	18.65	0.643	1.03
PA1978_at	65.40	45.13	0.087	1.45
PA1979_at	2.28	2.07	0.492	1.10
PA1980_at	4.96	2.55	0.286	1.95
PA1981_at	20.72	10.80	0.151	1.92
PA1982_exaA_at	13.40	10.43	0.349	1.28
PA1983_exaB_at	6.28	1.92	0.087	3.27
PA1984_s_at	2858.00	1650.05	0.008	1.73
PA1985_pqqA_at	18.38	28.55	0.048	-1.55
PA1986_pqqB_at	70.54	42.97	0.008	1.64
PA1987_pqqC_at	44.24	37.30	0.238	1.19
PA1988_pqqD_at	49.40	43.72	0.484	1.13
PA1989_pqqE_at	41.70	36.33	0.643	1.15

Supplementary Table 1. Probe set signal data for experimental and control samples

PA1990_at	10.60	9.45	0.643	1.12
PA1991_at	54.04	34.70	0.087	1.56
PA1992_at	19.48	18.95	1.000	1.03
PA1993_at	13.92	18.65	0.413	-1.34
PA1994_at	26.94	26.33	0.817	1.02
PA1995_i_at	90.68	106.55	0.087	-1.18
PA1996_ppiC1_at	58.04	69.22	0.151	-1.19
PA1997_at	64.22	62.53	0.817	1.03
PA1998_at	34.34	29.73	0.349	1.16
PA1999_at	1642.54	966.73	0.024	1.70
PA2000_at	918.10	806.10	0.349	1.14
PA2001_atoB_at	751.48	579.20	0.048	1.30
PA2002_at	53.46	40.20	0.048	1.33
PA2003_bdhA_at	138.22	125.00	1.000	1.11
PA2004_at	74.56	46.13	0.008	1.62
PA2005_at	54.02	37.58	0.008	1.44
PA2006_at	348.16	164.65	0.008	2.11
PA2007_maiA_i_at	952.56	483.58	0.008	1.97
PA2008_fahA_at	3275.64	1511.10	0.008	2.17
PA2009_hmgA_at	2244.54	1003.97	0.008	2.24
PA2010_at	30.40	34.28	0.643	-1.13
PA2011_at	261.96	151.43	0.008	1.73
PA2012_at	192.64	97.03	0.024	1.99
PA2013_at	282.32	183.17	0.024	1.54
PA2014_at	519.84	285.88	0.008	1.82
PA2015_at	1473.12	775.63	0.008	1.90
PA2016_at	543.34	392.15	0.087	1.39
PA2017_at	16.66	16.45	1.000	1.01
PA2018_at	38.54	26.60	0.079	1.45
PA2019_at	174.56	95.43	0.024	1.83
PA2020_at	68.54	57.20	0.238	1.20
PA2021_at	9.92	8.70	1.000	1.14
PA2022_at	18.66	14.67	0.643	1.27
PA2023_galU_at	454.38	583.38	0.008	-1.28
PA2024_at	16.54	13.95	0.349	1.19
PA2025_gor_at	139.06	110.05	0.024	1.26
PA2026_at	17.82	26.58	0.024	-1.49
PA2027_at	19.10	21.75	0.151	-1.14
PA2028_at	25.22	45.43	0.008	-1.80
PA2029_i_at	16.86	29.10	0.008	-1.73
PA2030_at	30.44	53.70	0.008	-1.76
PA2031_i_at	165.60	168.93	0.817	-1.02
PA2032_at	20.26	17.67	0.484	1.15
PA2033_at	15.54	17.07	0.817	-1.10
PA2034_at	2.04	12.40	0.008	-6.08
PA2035_at	17.34	13.72	0.238	1.26
PA2036_at	5.66	8.58	0.238	-1.52
PA2037_at	7.08	13.25	0.024	-1.87
PA2038_at	45.32	49.82	1.000	-1.10
PA2039_at	55.06	45.63	0.087	1.21
PA2041_at	43.52	34.55	0.024	1.26

Supplementary Table 1. Probe set signal data for experimental and control samples

PA2042_at	605.10	649.40	0.349	-1.07
PA2043_at	18.10	21.13	0.817	-1.17
PA2044_at	174.36	171.28	0.643	1.02
PA2045_at	134.02	171.73	0.008	-1.28
PA2046_at	1.66	1.48	0.643	1.12
PA2047_at	37.14	28.50	0.151	1.30
PA2048_at	8.88	10.05	0.643	-1.13
PA2049_at	66.28	65.82	0.817	1.01
PA2050_at	17.34	21.45	0.349	-1.24
PA2051_at	8.86	9.23	0.817	-1.04
PA2052_cynS_at	36.60	34.03	1.000	1.08
PA2053_cynT_at	17.12	18.77	0.643	-1.10
PA2054_cynR_at	23.52	22.63	0.905	1.04
PA2055_at	16.60	8.63	0.048	1.92
PA2056_at	20.80	15.05	0.048	1.38
PA2057_at	2.16	1.35	0.048	1.60
PA2058_at	4.54	9.63	0.032	-2.12
PA2059_at	16.92	10.70	0.349	1.58
PA2060_at	10.74	9.07	0.484	1.18
PA2061_at	9.64	3.22	0.008	2.99
PA2062_at	27.26	38.95	0.238	-1.43
PA2063_at	75.32	84.95	0.048	-1.13
PA2064_pcoB_at	13.26	15.52	0.643	-1.17
PA2065_pcoA_at	4.12	10.48	0.087	-2.54
PA2066_at	28.72	30.25	0.817	-1.05
PA2067_at	25.44	23.90	0.817	1.06
PA2068_at	13.22	11.45	0.349	1.15
PA2069_at	23.24	24.97	0.643	-1.07
PA2070_at	2.62	2.63	0.643	-1.00
PA2071_fusA2_at	22.30	21.88	0.349	1.02
PA2072_at	3.48	10.25	0.048	-2.95
PA2073_at	3.56	2.58	0.905	1.38
PA2074_at	16.80	16.45	0.817	1.02
PA2075_at	20.30	22.62	0.817	-1.11
PA2076_at	49.82	57.85	0.151	-1.16
PA2077_at	15.44	12.05	0.238	1.28
PA2078_at	4.54	3.72	0.643	1.22
PA2079_at	24.38	52.28	0.151	-2.14
PA2080_at	353.14	391.72	0.238	-1.11
PA2081_at	224.40	288.70	0.048	-1.29
PA2082_at	19.18	40.15	0.024	-2.09
PA2083_at	18.32	28.50	0.048	-1.56
PA2084_at	9.88	15.80	0.151	-1.60
PA2085_at	18.00	14.15	0.484	1.27
PA2086_at	12.82	14.72	0.484	-1.15
PA2087_at	9.84	5.33	0.151	1.85
PA2088_at	1.32	2.30	0.008	-1.74
PA2089_at	17.52	17.85	0.349	-1.02
PA2090_at	6.24	9.05	0.151	-1.45
PA2091_at	2.26	4.93	0.349	-2.18
PA2092_at	12.34	9.95	0.349	1.24

Supplementary Table 1. Probe set signal data for experimental and control samples

PA2093_at	5.38	16.20	0.008	-3.01
PA2094_at	17.48	22.65	0.238	-1.30
PA2095_at	10.72	7.80	0.349	1.37
PA2096_at	23.02	19.55	0.238	1.18
PA2097_at	17.80	13.05	0.087	1.36
PA2098_at	19.32	25.95	0.151	-1.34
PA2099_at	8.56	8.50	0.484	1.01
PA2100_at	17.78	23.02	0.349	-1.29
PA2101_at	13.50	10.50	0.238	1.29
PA2102_at	17.90	13.98	0.484	1.28
PA2103_at	41.84	31.85	0.079	1.31
PA2104_at	34.86	30.72	0.817	1.13
PA2105_at	35.22	35.83	0.817	-1.02
PA2106_at	25.92	19.95	0.238	1.30
PA2107_at	27.24	28.10	0.643	-1.03
PA2108_at	8.72	18.17	0.048	-2.08
PA2109_at	67.72	59.87	0.484	1.13
PA2110_at	372.20	225.00	0.008	1.65
PA2111_at	408.60	388.40	0.643	1.05
PA2112_at	867.66	585.90	0.024	1.48
PA2113_at	677.46	432.72	0.024	1.57
PA2114_at	304.26	242.43	0.349	1.26
PA2115_at	45.26	49.80	0.484	-1.10
PA2116_at	70.28	66.57	0.643	1.06
PA2117_at	40.70	41.55	0.556	-1.02
PA2118_ada_at	12.20	23.38	0.008	-1.92
PA2119_at	30.06	47.28	0.151	-1.57
PA2120_at	12.32	14.88	0.643	-1.21
PA2121_at	26.30	25.25	0.643	1.04
PA2122_at	2.84	2.95	0.611	-1.04
PA2123_at	29.22	22.18	0.238	1.32
PA2124_at	6.82	7.35	0.905	-1.08
PA2125_at	21.78	25.78	0.643	-1.18
PA2126_at	33.32	41.72	0.087	-1.25
PA2127_at	85.56	69.95	0.238	1.22
PA2128_at	29.76	26.10	0.817	1.14
PA2129_at	4.72	5.88	0.730	-1.25
PA2130_at	14.48	16.92	0.484	-1.17
PA2131_at	20.00	14.25	0.087	1.40
PA2132_at	13.56	10.85	0.817	1.25
PA2133_at	2.94	4.72	0.556	-1.61
PA2134_at	28.54	32.20	0.817	-1.13
PA2135_at	12.82	9.32	0.111	1.38
PA2136_at	10.14	3.73	0.349	2.72
PA2137_at	3.96	6.68	0.190	-1.69
PA2138_at	7.40	5.88	0.349	1.26
PA2139_r_at	4.82	11.40	0.151	-2.37
PA2140_at	6.04	15.50	0.032	-2.57
PA2141_at	4.40	3.30	0.817	1.33
PA2142_at	5.62	10.47	0.151	-1.86
PA2143_at	4.90	2.45	0.230	2.00

Supplementary Table 1. Probe set signal data for experimental and control samples

PA2144_glgP_at	5.14	17.52	0.008	-3.41
PA2145_at	35.80	13.40	0.008	2.67
PA2146_i_at	8.80	16.50	0.151	-1.88
PA2147_katE_at	1.42	1.70	0.635	-1.20
PA2148_at	10.54	16.17	0.238	-1.53
PA2149_at	18.48	21.73	0.643	-1.18
PA2150_at	2.32	4.90	0.556	-2.11
PA2151_at	2.16	1.80	0.794	1.20
PA2152_at	14.34	14.57	0.817	-1.02
PA2153_glgB_at	6.88	10.70	0.087	-1.56
PA2154_at	1.82	6.47	0.024	-3.55
PA2155_at	2.90	3.57	1.000	-1.23
PA2156_at	0.86	0.70	0.817	1.23
PA2157_at	18.22	16.27	0.643	1.12
PA2158_at	5.12	6.43	0.341	-1.26
PA2159_at	11.48	13.45	0.349	-1.17
PA2160_at	28.00	19.58	0.087	1.43
PA2161_at	2.52	2.22	0.905	1.14
PA2162_at	7.90	13.68	0.151	-1.73
PA2163_at	9.08	9.23	0.643	-1.02
PA2164_at	19.04	14.52	0.238	1.31
PA2165_at	12.60	11.25	0.238	1.12
PA2166_at	17.86	15.02	0.484	1.19
PA2167_at	2.00	5.63	0.032	-2.82
PA2168_at	22.06	25.20	0.817	-1.14
PA2169_at	18.46	22.28	0.643	-1.21
PA2170_at	5.80	6.28	0.905	-1.08
PA2171_at	21.16	11.98	0.087	1.77
PA2172_at	10.44	12.20	0.349	-1.17
PA2173_at	11.38	10.02	0.817	1.14
PA2174_at	15.62	10.27	0.349	1.52
PA2175_at	10.96	18.50	0.048	-1.69
PA2176_at	5.16	3.10	0.992	1.66
PA2177_at	25.50	22.88	0.643	1.11
PA2178_at	12.74	12.77	1.000	-1.00
PA2179_at	6.04	1.80	0.349	3.36
PA2180_at	13.18	15.60	0.643	-1.18
PA2181_at	14.32	12.45	1.000	1.15
PA2182_at	5.74	7.80	0.349	-1.36
PA2183_at	5.62	2.60	0.151	2.16
PA2184_at	14.88	16.40	0.643	-1.10
PA2185_at	2.60	8.32	0.048	-3.20
PA2186_r_at	13.56	20.32	0.349	-1.50
PA2187_at	18.58	9.13	0.008	2.04
PA2188_at	4.90	6.25	0.643	-1.28
PA2189_at	9.56	10.02	0.817	-1.05
PA2190_at	3.18	3.50	0.643	-1.10
PA2191_exoY_at	14.14	11.92	1.000	1.19
PA2192_at	1.40	2.25	0.730	-1.61
PA2193_hcnA_at	198.68	344.17	0.008	-1.73
PA2194_hcnB_at	301.64	354.07	0.484	-1.17

Supplementary Table 1. Probe set signal data for experimental and control samples

PA2195_hcnC_at	125.72	121.22	0.643	1.04
PA2196_at	39.58	46.13	0.087	-1.17
PA2197_at	136.82	112.27	0.048	1.22
PA2198_at	58.16	67.43	0.151	-1.16
PA2199_at	59.36	93.50	0.008	-1.58
PA2200_at	23.94	28.20	0.817	-1.18
PA2201_at	45.34	32.00	0.048	1.42
PA2202_at	9.52	18.85	0.087	-1.98
PA2203_at	28.08	34.13	0.643	-1.22
PA2204_at	142.84	312.98	0.151	-2.19
PA2205_at	35.60	32.58	0.643	1.09
PA2206_at	26.44	19.70	0.024	1.34
PA2207_at	8.78	9.60	0.643	-1.09
PA2208_at	14.76	8.23	0.087	1.79
PA2209_at	6.76	9.57	0.190	-1.42
PA2210_at	17.16	17.77	0.817	-1.04
PA2211_at	5.34	12.23	0.024	-2.29
PA2212_at	25.06	36.22	0.087	-1.45
PA2213_at	5.84	5.80	0.817	1.01
PA2214_at	6.90	6.00	0.817	1.15
PA2215_at	4.02	3.88	0.730	1.04
PA2216_at	32.30	27.05	0.349	1.19
PA2217_at	2.86	2.52	0.817	1.13
PA2218_at	24.26	31.88	0.087	-1.31
PA2219_opdE_at	16.00	15.02	0.349	1.07
PA2220_at	32.08	31.35	0.643	1.02
PA2221_at	12.38	5.98	0.151	2.07
PA2222_at	8.54	11.05	0.238	-1.29
PA2223_at	17.02	19.80	0.238	-1.16
PA2224_at	3.00	1.68	0.111	1.79
PA2225_at	10.48	13.30	0.817	-1.27
PA2226_at	4.54	6.20	0.151	-1.37
PA2227_at	11.18	7.25	0.111	1.54
PA2228_at	3.00	0.60	0.008	5.00
PA2229_at	64.02	65.80	0.817	-1.03
PA2230_at	59.30	58.15	0.817	1.02
PA2231_at	122.86	126.80	0.817	-1.03
PA2232_at	153.24	171.30	0.151	-1.12
PA2233_at	138.84	115.70	0.349	1.20
PA2234_at	253.86	330.00	0.079	-1.30
PA2235_at	178.74	204.85	0.151	-1.15
PA2236_at	146.98	196.38	0.024	-1.34
PA2237_at	76.00	90.32	0.238	-1.19
PA2238_at	49.96	57.30	0.349	-1.15
PA2239_at	45.32	55.57	0.048	-1.23
PA2240_at	38.62	42.82	0.349	-1.11
PA2241_at	24.56	29.10	0.151	-1.18
PA2242_at	78.58	86.52	0.643	-1.10
PA2243_at	22.66	14.70	0.349	1.54
PA2244_at	10.98	8.82	0.349	1.24
PA2245_at	2.76	3.30	0.643	-1.20

Supplementary Table 1. Probe set signal data for experimental and control samples

PA2246_bkdR_at	47.88	46.20	0.484	1.04
PA2247_bkdA1_at	2145.82	1002.33	0.008	2.14
PA2248_bkdA2_at	1414.54	575.22	0.008	2.46
PA2249_bkdB_at	676.44	415.92	0.008	1.63
PA2250_lpdV_at	537.80	338.30	0.024	1.59
PA2251_at	10.76	25.27	0.008	-2.35
PA2252_at	30.02	274.73	0.238	-9.15
PA2253_ansA_at	68.54	143.88	0.643	-2.10
PA2254_pvcA_at	8.98	5.50	0.484	1.63
PA2255_pvcB_at	2.36	3.35	0.349	-1.42
PA2256_pvcC_at	9.86	13.07	0.643	-1.33
PA2257_pvcD_at	14.02	10.10	0.643	1.39
PA2258_ptxR_at	2.82	3.38	0.643	-1.20
PA2259_ptxS_at	115.62	83.68	0.238	1.38
PA2260_at	29.56	25.13	0.484	1.18
PA2261_at	8.98	7.17	0.349	1.25
PA2262_at	12.68	9.80	0.190	1.29
PA2263_at	20.50	20.92	0.984	-1.02
PA2264_at	84.14	115.05	0.008	-1.37
PA2265_at	113.18	126.05	0.238	-1.11
PA2266_at	82.08	64.90	0.024	1.26
PA2267_at	43.06	46.67	0.484	-1.08
PA2268_at	18.50	14.13	0.230	1.31
PA2269_at	23.94	26.42	0.643	-1.10
PA2270_at	248.52	208.20	0.024	1.19
PA2271_i_at	79.98	68.45	0.048	1.17
PA2272_pbpC_at	13.90	15.85	0.817	-1.14
PA2273_at	17.44	29.97	0.048	-1.72
PA2274_at	23.30	21.22	0.643	1.10
PA2275_at	14.48	16.63	0.349	-1.15
PA2276_at	10.08	8.93	1.000	1.13
PA2277_arsR_at	32.10	36.63	0.238	-1.14
PA2278_arsB_at	22.72	24.67	0.349	-1.09
PA2279_arsC_at	42.40	59.60	0.008	-1.41
PA2280_at	23.76	32.95	0.151	-1.39
PA2281_at	65.30	72.18	0.484	-1.11
PA2282_at	32.64	29.12	0.635	1.12
PA2283_at	14.30	15.35	0.817	-1.07
PA2284_at	3.78	10.07	0.008	-2.66
PA2285_at	31.30	59.17	0.008	-1.89
PA2286_at	27.42	35.28	0.048	-1.29
PA2287_at	13.64	13.55	0.817	1.01
PA2288_r_at	449.34	43.72	0.008	10.28
PA2289_at	25.22	27.65	0.468	-1.10
PA2290_gcd_at	168.82	195.55	0.484	-1.16
PA2292_at	3.26	13.50	0.008	-4.14
PA2293_at	3.64	4.93	0.151	-1.35
PA2294_at	20.32	19.05	0.817	1.07
PA2295_at	8.64	12.02	0.286	-1.39
PA2296_at	17.50	10.63	0.341	1.65
PA2297_at	35.96	31.73	0.643	1.13

Supplementary Table 1. Probe set signal data for experimental and control samples

PA2298_at	30.14	37.83	0.238	-1.26
PA2299_at	29.68	28.45	1.000	1.04
PA2300_chiC_at	19.88	26.45	0.484	-1.33
PA2301_at	23.82	33.97	0.087	-1.43
PA2302_at	39.18	28.77	0.008	1.36
PA2303_at	23.74	33.75	0.349	-1.42
PA2304_at	75.28	61.68	0.151	1.22
PA2305_at	100.06	100.32	0.643	-1.00
PA2306_at	71.82	61.75	0.151	1.16
PA2307_at	10.04	4.22	0.349	2.38
PA2308_at	1.88	5.03	0.024	-2.68
PA2309_at	8.64	9.02	0.643	-1.04
PA2310_at	3.04	8.73	0.024	-2.87
PA2311_r_at	49.18	59.23	0.349	-1.20
PA2312_at	17.08	21.20	0.484	-1.24
PA2313_at	8.14	7.32	0.817	1.11
PA2314_at	8.14	3.98	0.024	2.05
PA2315_at	9.78	13.83	0.484	-1.41
PA2316_at	23.44	20.17	0.643	1.16
PA2317_at	27.14	34.92	0.151	-1.29
PA2318_at	37.96	55.55	0.024	-1.46
PA2320_gntR_at	86.06	70.80	0.349	1.22
PA2321_at	61.24	93.45	0.111	-1.53
PA2322_at	43.62	53.90	0.484	-1.24
PA2323_at	184.44	128.68	0.008	1.43
PA2324_at	10.78	8.10	0.484	1.33
PA2325_at	12.60	21.00	0.087	-1.67
PA2326_at	8.46	19.90	0.008	-2.35
PA2327_at	19.56	12.55	0.151	1.56
PA2328_at	30.34	26.52	0.643	1.14
PA2329_at	25.32	20.28	0.087	1.25
PA2330_at	26.16	28.30	0.349	-1.08
PA2331_at	46.24	38.15	0.484	1.21
PA2332_at	83.26	72.18	0.349	1.15
PA2333_at	7.96	12.38	0.238	-1.56
PA2334_at	5.18	23.02	0.008	-4.44
PA2335_at	8.92	6.20	0.643	1.44
PA2336_at	28.08	14.57	0.048	1.93
PA2337_mtlR_at	23.78	17.77	0.238	1.34
PA2338_at	28.50	13.63	0.008	2.09
PA2339_at	11.12	6.22	0.238	1.79
PA2340_at	5.70	10.95	0.151	-1.92
PA2341_at	16.96	28.23	0.151	-1.66
PA2342_mtlD_at	6.70	10.35	0.151	-1.54
PA2343_mtlY_at	11.14	13.13	0.810	-1.18
PA2344_mtlZ_at	48.04	54.90	0.238	-1.14
PA2345_at	20.18	36.28	0.008	-1.80
PA2346_at	5.98	8.85	0.484	-1.48
PA2347_at	4.56	8.60	0.087	-1.89
PA2348_at	9.02	6.63	0.643	1.36
PA2349_at	28.86	24.20	0.341	1.19

Supplementary Table 1. Probe set signal data for experimental and control samples

PA2350_at	10.52	21.98	0.063	-2.09
PA2351_at	18.62	15.55	0.817	1.20
PA2352_at	59.02	80.90	0.024	-1.37
PA2353_at	28.64	21.38	0.008	1.34
PA2354_at	14.42	9.30	0.032	1.55
PA2355_at	9.28	8.95	0.643	1.04
PA2356_msuD_at	4.98	5.25	1.000	-1.05
PA2357_msuE_at	7.32	9.60	0.556	-1.31
PA2358_at	36.90	34.65	0.643	1.06
PA2359_at	4.98	12.20	0.008	-2.45
PA2360_at	31.18	31.03	1.000	1.00
PA2361_at	20.26	12.55	0.008	1.61
PA2362_at	19.08	8.73	0.151	2.19
PA2363_at	31.90	19.48	0.008	1.64
PA2364_at	39.86	27.33	0.008	1.46
PA2365_at	40.82	23.85	0.008	1.71
PA2366_at	18.18	30.58	0.008	-1.68
PA2367_at	18.26	16.02	0.349	1.14
PA2368_i_at	23.74	32.13	0.087	-1.35
PA2369_at	19.60	28.73	0.087	-1.47
PA2370_at	24.72	12.72	0.008	1.94
PA2371_at	16.12	14.53	0.817	1.11
PA2372_at	16.40	2.90	0.008	5.66
PA2373_at	13.80	30.45	0.008	-2.21
PA2374_at	9.14	13.00	0.349	-1.42
PA2375_i_at	10.40	8.88	0.643	1.17
PA2376_at	19.68	18.23	0.349	1.08
PA2377_at	12.00	12.15	1.000	-1.01
PA2378_at	259.12	141.05	0.008	1.84
PA2379_at	209.24	188.60	0.817	1.11
PA2380_at	33.36	50.15	0.024	-1.50
PA2381_at	138.68	102.00	0.151	1.36
PA2382_lldA_at	24.36	17.73	0.341	1.37
PA2383_at	51.64	45.42	0.484	1.14
PA2384_at	10.56	41.92	0.008	-3.97
PA2385_at	21.26	31.88	0.151	-1.50
PA2386_pvdA_at	17.92	53.20	0.048	-2.97
PA2387_at	25.40	40.53	0.024	-1.60
PA2388_at	151.92	183.67	0.024	-1.21
PA2389_at	49.78	77.25	0.008	-1.55
PA2390_at	56.48	73.55	0.151	-1.30
PA2391_at	33.32	54.42	0.008	-1.63
PA2392_at	14.66	16.88	0.238	-1.15
PA2393_at	10.22	29.70	0.024	-2.91
PA2394_at	24.36	40.58	0.151	-1.67
PA2395_at	13.30	46.43	0.024	-3.49
PA2396_at	17.86	30.53	0.048	-1.71
PA2397_pvdE_at	17.22	24.75	0.349	-1.44
PA2398_fpvA_at	99.54	566.10	0.008	-5.69
PA2399_pvdD_at	27.88	29.13	0.817	-1.04
PA2400_at	14.78	8.33	0.413	1.77

Supplementary Table 1. Probe set signal data for experimental and control samples

PA2401_at	14.06	12.98	1.000	1.08
PA2402_at	22.78	27.63	0.484	-1.21
PA2403_at	53.66	173.75	0.008	-3.24
PA2404_at	45.56	138.13	0.008	-3.03
PA2405_at	46.90	181.08	0.008	-3.86
PA2406_at	61.44	113.25	0.008	-1.84
PA2407_at	91.84	300.93	0.008	-3.28
PA2408_at	35.82	103.70	0.008	-2.90
PA2409_at	50.86	188.55	0.008	-3.71
PA2410_at	38.20	94.38	0.008	-2.47
PA2411_at	6.42	35.63	0.024	-5.55
PA2412_at	7.06	47.28	0.024	-6.70
PA2413_at	12.26	29.88	0.048	-2.44
PA2414_at	16.32	9.05	0.087	1.80
PA2415_at	41.18	22.88	0.151	1.80
PA2416_treA_at	20.00	28.38	0.238	-1.42
PA2417_at	15.68	11.50	0.079	1.36
PA2418_at	14.04	11.67	0.484	1.20
PA2419_at	7.10	6.53	0.643	1.09
PA2420_at	13.34	8.68	0.484	1.54
PA2421_at	17.06	13.70	0.484	1.25
PA2422_at	13.06	17.52	0.286	-1.34
PA2423_at	39.46	54.72	0.048	-1.39
PA2424_at	9.20	13.95	0.238	-1.52
PA2425_at	8.98	16.27	0.238	-1.81
PA2426_pvdS_at	21.00	62.95	0.024	-3.00
PA2427_at	6.14	13.90	0.008	-2.26
PA2428_at	24.78	16.67	0.151	1.49
PA2429_at	12.08	10.20	1.000	1.18
PA2430_at	16.18	21.05	0.341	-1.30
PA2431_at	21.06	17.90	0.087	1.18
PA2432_at	50.08	36.40	0.008	1.38
PA2433_at	30.88	28.35	0.643	1.09
PA2434_at	36.90	20.07	0.008	1.84
PA2435_at	19.52	14.35	0.151	1.36
PA2436_at	53.44	62.13	0.238	-1.16
PA2437_at	28.62	21.35	0.048	1.34
PA2438_at	4.54	8.15	0.238	-1.80
PA2439_at	2.92	2.47	0.730	1.18
PA2440_at	31.30	26.85	0.349	1.17
PA2441_at	6.10	9.55	0.286	-1.57
PA2442_gcvT2_at	152.58	160.48	1.000	-1.05
PA2443_sdaA_at	154.80	170.65	0.643	-1.10
PA2444_glyA2_at	145.42	221.80	0.048	-1.53
PA2445_gcvP2_at	1095.08	818.52	0.008	1.34
PA2446_gcvH2_at	694.42	564.15	0.238	1.23
PA2447_at	37.88	22.58	0.087	1.68
PA2448_at	23.68	23.03	0.992	1.03
PA2449_at	51.76	43.77	0.238	1.18
PA2450_at	111.44	90.05	0.151	1.24
PA2451_at	31.48	24.15	0.151	1.30

Supplementary Table 1. Probe set signal data for experimental and control samples

PA2452_at	17.64	27.83	0.151	-1.58
PA2453_at	198.12	390.38	0.008	-1.97
PA2454_at	79.88	108.30	0.087	-1.36
PA2455_at	51.04	63.00	0.087	-1.23
PA2456_at	24.86	33.08	0.024	-1.33
PA2457_at	27.68	42.40	0.087	-1.53
PA2458_at	9.44	9.83	1.000	-1.04
PA2459_at	20.04	24.87	0.349	-1.24
PA2460_i_at	58.90	90.07	0.008	-1.53
PA2461_at	9.08	16.50	0.151	-1.82
PA2462_at	151.72	142.23	0.151	1.07
PA2464_at	329.84	267.55	0.024	1.23
PA2465_at	12.32	3.90	0.024	3.16
PA2466_at	15.74	8.50	0.008	1.85
PA2467_at	25.66	30.35	0.349	-1.18
PA2468_at	22.00	17.02	0.238	1.29
PA2469_at	22.94	27.15	0.817	-1.18
PA2470_gtdA_at	17.42	17.15	1.000	1.02
PA2471_at	5.72	6.75	0.817	-1.18
PA2472_at	7.66	5.35	0.413	1.43
PA2473_at	14.54	8.23	0.087	1.77
PA2474_at	16.20	10.33	0.556	1.57
PA2475_at	36.20	16.75	0.008	2.16
PA2476_dsbG_at	112.70	109.80	0.643	1.03
PA2477_at	33.96	31.10	0.484	1.09
PA2478_at	19.50	19.15	1.000	1.02
PA2479_at	46.10	47.68	0.643	-1.03
PA2480_at	10.16	13.43	0.484	-1.32
PA2481_at	265.12	233.88	0.238	1.13
PA2482_at	297.22	288.15	0.817	1.03
PA2483_at	85.26	93.30	0.151	-1.09
PA2484_at	51.74	57.95	0.349	-1.12
PA2485_at	22.16	15.10	0.151	1.47
PA2486_at	7.66	6.10	0.817	1.26
PA2487_i_at	28.30	15.22	0.008	1.86
PA2488_at	29.36	40.83	0.024	-1.39
PA2489_at	26.58	18.02	0.143	1.48
PA2490_at	14.22	17.45	0.063	-1.23
PA2491_at	108.50	92.82	0.087	1.17
PA2492_mexT_at	94.08	87.40	0.484	1.08
PA2493_mexE_at	9.20	3.08	0.040	2.99
PA2494_mexF_at	7.20	10.85	0.151	-1.51
PA2495_oprN_at	20.06	15.85	0.349	1.27
PA2496_at	34.44	29.60	0.238	1.16
PA2497_at	16.16	17.75	0.817	-1.10
PA2498_at	28.78	34.88	0.151	-1.21
PA2499_at	17.32	9.10	0.484	1.90
PA2500_at	8.32	9.93	0.484	-1.19
PA2501_at	81.40	150.73	0.048	-1.85
PA2502_at	35.88	53.85	0.008	-1.50
PA2503_at	157.66	142.55	0.349	1.11

Supplementary Table 1. Probe set signal data for experimental and control samples

PA2504_at	24.12	27.42	0.111	-1.14
PA2505_at	15.64	11.97	0.238	1.31
PA2506_at	23.72	23.98	0.476	-1.01
PA2507_catA_at	14.88	13.90	0.484	1.07
PA2508_catC_at	16.14	13.70	0.817	1.18
PA2509_catB_at	10.48	2.57	0.048	4.08
PA2510_catR_at	55.70	45.42	0.024	1.23
PA2511_at	14.36	13.77	0.817	1.04
PA2512_antA_at	7.46	3.38	0.087	2.21
PA2513_antB_at	14.68	14.80	0.817	-1.01
PA2514_antC_at	8.30	10.90	0.349	-1.31
PA2515_xylL_at	4.36	6.63	0.238	-1.52
PA2516_xylZ_at	5.58	4.38	0.643	1.27
PA2517_xylY_at	22.40	11.10	0.008	2.02
PA2518_xylX_at	1.54	2.75	0.484	-1.79
PA2519_xylS_at	20.38	13.27	0.087	1.54
PA2520_czcA_at	11.46	16.73	0.238	-1.46
PA2521_czcB_at	27.96	21.92	0.643	1.28
PA2522_czcC_at	3.28	2.67	0.817	1.23
PA2523_at	59.30	29.88	0.048	1.98
PA2524_at	45.72	32.53	0.151	1.41
PA2525_at	63.90	50.83	0.238	1.26
PA2526_at	53.32	36.00	0.008	1.48
PA2527_at	46.24	36.72	0.238	1.26
PA2528_at	113.06	74.42	0.008	1.52
PA2529_at	143.96	88.30	0.008	1.63
PA2530_at	143.90	112.98	0.048	1.27
PA2531_at	15.20	23.30	0.151	-1.53
PA2532_tpx_at	264.32	231.20	0.484	1.14
PA2533_at	265.58	186.95	0.024	1.42
PA2534_at	12.92	19.45	0.238	-1.51
PA2535_at	27.94	25.15	0.817	1.11
PA2536_at	71.20	92.28	0.151	-1.30
PA2537_at	156.70	199.52	0.048	-1.27
PA2538_at	168.14	146.15	0.349	1.15
PA2539_at	61.68	49.82	0.484	1.24
PA2540_at	205.26	170.80	0.643	1.20
PA2541_at	120.90	147.65	0.048	-1.22
PA2542_at	35.00	30.77	0.151	1.14
PA2543_at	49.90	43.70	0.643	1.14
PA2544_at	29.94	20.05	0.008	1.49
PA2545_xthA_at	250.54	258.33	0.349	-1.03
PA2546_at	3.28	20.78	0.008	-6.34
PA2547_at	20.82	22.55	0.817	-1.08
PA2548_at	5.68	8.90	0.151	-1.57
PA2549_at	35.08	25.92	0.238	1.35
PA2550_at	21.44	19.00	0.484	1.13
PA2551_at	148.08	86.30	0.008	1.72
PA2552_at	350.40	155.27	0.008	2.26
PA2553_at	442.80	181.02	0.008	2.45
PA2554_at	240.36	139.22	0.008	1.73

Supplementary Table 1. Probe set signal data for experimental and control samples

PA2555_at	69.66	31.35	0.008	2.22
PA2556_at	37.76	43.10	0.087	-1.14
PA2557_at	114.38	58.05	0.048	1.97
PA2558_at	29.14	30.38	0.817	-1.04
PA2559_at	144.88	107.95	0.008	1.34
PA2560_at	30.42	40.05	0.349	-1.32
PA2561_at	50.82	41.65	0.238	1.22
PA2562_at	54.64	57.03	1.000	-1.04
PA2563_at	9.60	12.50	0.349	-1.30
PA2564_at	11.92	7.65	0.349	1.56
PA2565_at	46.70	40.13	0.643	1.16
PA2566_at	5.50	9.40	0.111	-1.71
PA2567_at	56.58	46.10	0.048	1.23
PA2568_at	36.90	31.92	0.349	1.16
PA2569_at	38.76	20.78	0.008	1.87
PA2570_pa1L_at	16.14	8.05	0.151	2.00
PA2571_at	3.70	2.95	0.484	1.25
PA2572_at	18.16	20.72	0.484	-1.14
PA2573_at	12.80	12.47	0.817	1.03
PA2574_at	7.72	9.58	0.349	-1.24
PA2575_at	337.64	232.82	0.008	1.45
PA2576_at	25.48	27.38	0.817	-1.07
PA2577_at	32.88	38.90	0.349	-1.18
PA2578_at	36.86	15.48	0.008	2.38
PA2579_at	197.40	386.00	0.008	-1.96
PA2580_at	18.44	25.77	0.151	-1.40
PA2581_at	139.32	152.95	0.349	-1.10
PA2582_at	195.62	342.42	0.048	-1.75
PA2583_at	54.28	42.40	0.349	1.28
PA2584_pgsA_at	193.34	242.95	0.087	-1.26
PA2585_uvrC_at	63.52	90.90	0.008	-1.43
PA2586_gacA_at	350.04	345.55	0.817	1.01
PA2587_at	95.18	134.02	0.008	-1.41
PA2588_at	32.34	34.22	1.000	-1.06
PA2589_at	17.22	17.95	0.635	-1.04
PA2590_at	9.54	15.70	0.143	-1.65
PA2591_at	34.04	62.02	0.024	-1.82
PA2592_at	69.34	111.50	0.008	-1.61
PA2593_at	19.74	34.05	0.008	-1.72
PA2594_at	20.26	22.30	0.484	-1.10
PA2595_at	5.60	32.95	0.008	-5.88
PA2596_at	5.56	12.43	0.024	-2.24
PA2597_at	51.28	68.20	0.349	-1.33
PA2598_at	26.64	25.08	0.817	1.06
PA2599_at	27.58	22.28	0.087	1.24
PA2600_at	26.34	12.63	0.008	2.09
PA2601_at	52.60	41.52	0.063	1.27
PA2602_at	15.34	15.43	0.817	-1.01
PA2603_at	21.90	29.88	0.143	-1.36
PA2604_at	473.56	341.47	0.048	1.39
PA2605_at	85.10	85.13	0.817	-1.00

Supplementary Table 1. Probe set signal data for experimental and control samples

PA2606_at	157.88	189.98	0.024	-1.20
PA2607_at	68.70	96.50	0.048	-1.40
PA2608_at	88.82	98.47	0.413	-1.11
PA2609_at	78.98	51.75	0.008	1.53
PA2610_at	21.42	14.10	0.151	1.52
PA2611_cysG_at	100.54	106.52	0.349	-1.06
PA2612_serS_at	841.62	779.65	0.087	1.08
PA2613_at	162.28	170.45	0.643	-1.05
PA2614_lolA_at	278.96	354.10	0.008	-1.27
PA2615_ftsK_at	226.58	243.85	0.484	-1.08
PA2616_trxB1_at	696.80	774.72	0.349	-1.11
PA2617_aat_at	115.86	104.40	0.048	1.11
PA2618_at	34.78	35.58	1.000	-1.02
PA2619_infA_at	280.62	767.30	0.008	-2.73
PA2620_clpA_at	381.68	294.23	0.008	1.30
PA2621_at	164.84	250.15	0.008	-1.52
PA2622_cspD_at	137.38	169.47	0.238	-1.23
PA2623_icd_at	1324.26	1188.75	0.349	1.11
PA2624_idh_at	2521.12	2040.95	0.008	1.24
PA2625_at	163.36	188.45	0.238	-1.15
PA2626_trmU_at	565.28	549.43	0.643	1.03
PA2627_at	349.94	367.48	0.643	-1.05
PA2628_at	36.24	48.73	0.024	-1.34
PA2629_purB_at	606.96	922.92	0.008	-1.52
PA2630_at	88.66	213.63	0.008	-2.41
PA2631_at	215.04	248.75	0.484	-1.16
PA2632_at	72.34	72.65	0.730	-1.00
PA2633_at	69.54	62.72	0.349	1.11
PA2634_at	460.14	396.80	0.349	1.16
PA2635_at	26.30	19.00	0.238	1.38
PA2636_at	17.18	21.85	0.349	-1.27
PA2637_nuoA_at	264.42	480.88	0.008	-1.82
PA2638_nuoB_at	716.56	836.40	0.151	-1.17
PA2639_nuoD_at	792.90	932.18	0.087	-1.18
PA2640_nuoE_at	1424.52	1259.20	0.008	1.13
PA2641_nuoF_at	690.92	793.73	0.151	-1.15
PA2642_nuoG_at	1079.96	984.15	0.151	1.10
PA2643_nuoH_at	922.28	846.42	1.000	1.09
PA2644_nuoI_at	715.96	662.90	0.817	1.08
PA2645_nuoJ_at	533.72	653.78	0.087	-1.22
PA2646_nuoK_at	941.70	1019.75	0.238	-1.08
PA2647_nuoL_at	536.20	548.10	1.000	-1.02
PA2648_nuoM_at	531.08	547.92	0.484	-1.03
PA2649_nuoN_at	368.66	348.82	1.000	1.06
PA2650_at	67.22	52.72	0.048	1.28
PA2651_at	63.84	67.60	0.349	-1.06
PA2652_at	118.70	181.43	0.008	-1.53
PA2653_at	55.80	130.32	0.008	-2.34
PA2654_at	167.32	130.50	0.048	1.28
PA2655_i_at	2.74	5.53	0.087	-2.02
PA2656_at	42.26	36.42	0.048	1.16

Supplementary Table 1. Probe set signal data for experimental and control samples

PA2657_at	96.68	94.63	0.476	1.02
PA2658_at	161.48	169.05	0.484	-1.05
PA2659_at	135.90	148.27	0.238	-1.09
PA2660_at	187.26	155.88	0.087	1.20
PA2661_at	97.90	87.77	0.484	1.12
PA2662_at	11.46	15.82	0.349	-1.38
PA2663_at	12.42	7.98	0.048	1.56
PA2664_fhp_at	2.48	2.05	0.905	1.21
PA2665_at	55.18	48.48	0.151	1.14
PA2666_at	117.26	168.52	0.087	-1.44
PA2667_at	160.48	480.75	0.008	-3.00
PA2668_at	19.30	16.05	0.349	1.20
PA2669_at	8.38	6.83	0.484	1.23
PA2670_at	6.00	2.10	0.048	2.86
PA2671_at	11.88	10.75	0.413	1.11
PA2672_at	2.68	15.55	0.008	-5.80
PA2673_at	5.08	5.78	0.643	-1.14
PA2674_at	15.22	14.77	0.730	1.03
PA2675_at	11.16	18.10	0.151	-1.62
PA2676_at	13.18	8.77	0.286	1.50
PA2677_at	6.24	5.28	0.730	1.18
PA2678_at	4.78	11.32	0.024	-2.37
PA2679_at	22.08	21.77	0.817	1.01
PA2680_at	6.58	10.25	0.349	-1.56
PA2681_at	7.90	13.70	0.151	-1.73
PA2682_at	30.10	53.17	0.024	-1.77
PA2683_at	55.92	67.70	0.087	-1.21
PA2684_at	203.34	164.27	0.048	1.24
PA2685_at	179.12	157.07	1.000	1.14
PA2686_pfeR_at	17.82	20.42	0.484	-1.15
PA2687_pfeS_at	16.80	15.77	0.905	1.07
PA2688_pfeA_at	14.16	6.85	0.151	2.07
PA2689_at	2.66	7.50	0.349	-2.82
PA2691_at	19.44	26.68	0.087	-1.37
PA2692_at	80.46	95.20	0.087	-1.18
PA2693_at	24.78	21.58	0.238	1.15
PA2694_at	12.38	19.65	0.087	-1.59
PA2695_at	48.78	52.60	0.484	-1.08
PA2696_at	15.40	13.93	0.556	1.11
PA2697_at	9.54	13.55	0.190	-1.42
PA2698_at	34.96	29.48	0.190	1.19
PA2699_at	18.42	10.48	0.048	1.76
PA2700_at	7.24	14.13	0.151	-1.95
PA2701_at	8.38	9.17	1.000	-1.09
PA2702_at	171.02	131.50	0.048	1.30
PA2703_at	66.68	66.90	1.000	-1.00
PA2704_at	12.02	15.60	0.238	-1.30
PA2705_at	38.88	51.52	0.087	-1.33
PA2706_at	115.30	132.27	0.008	-1.15
PA2707_at	150.92	139.30	0.643	1.08
PA2708_at	26.62	15.27	0.008	1.74

Supplementary Table 1. Probe set signal data for experimental and control samples

PA2709_cysK_at	345.22	235.10	0.008	1.47
PA2710_at	42.78	48.00	0.341	-1.12
PA2711_at	57.14	43.75	0.151	1.31
PA2712_at	28.10	31.35	0.349	-1.12
PA2713_at	30.40	23.17	0.087	1.31
PA2714_at	17.22	23.25	0.087	-1.35
PA2715_at	12.76	5.88	0.008	2.17
PA2716_at	7.88	5.13	0.484	1.54
PA2717_cpo_at	11.68	11.63	0.643	1.00
PA2718_at	54.72	47.47	0.349	1.15
PA2719_at	9.30	3.83	0.008	2.43
PA2720_at	43.96	48.17	0.349	-1.10
PA2721_at	8.76	6.28	0.286	1.39
PA2722_at	13.16	10.98	0.349	1.20
PA2723_at	69.82	60.65	0.151	1.15
PA2724_r_at	78.02	70.82	0.730	1.10
PA2725_at	149.24	111.78	0.087	1.34
PA2726_at	123.78	76.13	0.008	1.63
PA2727_at	147.06	103.13	0.008	1.43
PA2728_at	159.30	120.38	0.151	1.32
PA2729_at	173.34	98.57	0.008	1.76
PA2730_at	69.18	153.40	0.008	-2.22
PA2731_at	100.54	194.85	0.008	-1.94
PA2732_at	118.44	157.57	0.048	-1.33
PA2733_at	301.44	345.13	0.008	-1.14
PA2734_at	163.22	237.13	0.008	-1.45
PA2735_at	271.26	420.78	0.008	-1.55
PA2736_at	247.90	372.33	0.008	-1.50
PA2737_at	541.68	482.83	0.238	1.12
PA2738_himA_at	863.04	829.03	0.484	1.04
PA2739_pheT_at	454.64	561.45	0.048	-1.23
PA2740_pheS_at	604.48	829.45	0.008	-1.37
PA2741_rplT_at	688.82	1130.90	0.008	-1.64
PA2742_rpmI_at	2963.10	3700.15	0.024	-1.25
PA2743_infC_at	2375.80	2891.90	0.024	-1.22
PA2744_thrS_at	766.20	842.47	0.349	-1.10
PA2745_at	32.04	50.68	0.008	-1.58
PA2746_at	5.18	3.17	0.190	1.63
PA2747_at	41.70	26.45	0.008	1.58
PA2748_at	31.32	35.77	0.349	-1.14
PA2749_endA_at	68.14	90.80	0.008	-1.33
PA2750_at	59.52	54.85	0.349	1.09
PA2751_at	13.14	19.92	0.048	-1.52
PA2752_at	20.28	11.67	0.048	1.74
PA2753_at	12.58	20.00	0.238	-1.59
PA2754_at	31.40	43.75	0.151	-1.39
PA2755_eco_at	329.00	403.15	0.151	-1.23
PA2756_at	52.08	54.80	0.643	-1.05
PA2757_at	130.20	120.72	0.238	1.08
PA2758_at	29.94	25.48	0.087	1.18
PA2759_at	4.52	7.07	0.151	-1.56

Supplementary Table 1. Probe set signal data for experimental and control samples

PA2760_at	1662.74	2565.07	0.024	-1.54
PA2761_at	216.10	227.88	0.643	-1.05
PA2762_at	28.40	22.65	0.238	1.25
PA2763_at	12.44	16.52	0.349	-1.33
PA2764_at	33.54	24.22	0.151	1.38
PA2765_at	327.28	347.33	0.349	-1.06
PA2766_at	24.60	21.80	0.643	1.13
PA2767_at	10.42	14.25	0.238	-1.37
PA2768_at	13.44	14.68	0.643	-1.09
PA2769_at	63.70	72.13	0.238	-1.13
PA2770_at	205.86	281.05	0.008	-1.37
PA2771_at	20.12	14.28	0.087	1.41
PA2772_at	26.70	37.45	0.048	-1.40
PA2773_at	29.02	27.65	0.643	1.05
PA2774_at	97.56	134.68	0.008	-1.38
PA2775_at	82.10	107.73	0.151	-1.31
PA2776_at	130.26	109.85	0.024	1.19
PA2777_at	21.90	17.20	0.238	1.27
PA2778_at	41.82	23.60	0.024	1.77
PA2779_at	70.30	64.28	0.817	1.09
PA2780_at	42.56	45.50	0.484	-1.07
PA2781_at	47.50	39.58	0.151	1.20
PA2782_at	22.48	24.38	0.643	-1.08
PA2783_at	15.36	27.08	0.111	-1.76
PA2784_at	25.00	30.38	0.087	-1.22
PA2785_at	6.24	15.45	0.008	-2.48
PA2786_at	22.20	17.02	0.643	1.30
PA2787_cpg2_at	13.70	3.80	0.008	3.61
PA2788_at	53.28	47.92	0.413	1.11
PA2789_at	23.88	22.77	0.643	1.05
PA2790_at	29.86	23.25	0.063	1.28
PA2791_at	17.04	18.50	0.643	-1.09
PA2792_at	44.26	62.07	0.008	-1.40
PA2793_at	126.54	129.05	1.000	-1.02
PA2794_at	15.80	10.25	0.048	1.54
PA2795_at	55.20	44.45	0.143	1.24
PA2796_tal_at	187.60	138.63	0.151	1.35
PA2797_at	88.56	71.53	0.151	1.24
PA2798_at	267.48	345.58	0.024	-1.29
PA2799_at	14.90	24.25	0.024	-1.63
PA2800_at	526.66	680.45	0.024	-1.29
PA2801_at	80.72	103.15	0.048	-1.28
PA2802_at	95.82	119.45	0.008	-1.25
PA2803_at	7.06	8.55	0.484	-1.21
PA2804_at	16.38	4.18	0.008	3.92
PA2805_at	88.36	154.45	0.024	-1.75
PA2806_at	28.58	33.65	0.238	-1.18
PA2807_at	2.48	2.00	0.905	1.24
PA2808_i_at	20.80	14.95	0.349	1.39
PA2809_at	23.16	27.38	0.349	-1.18
PA2810_at	16.72	25.82	0.024	-1.54

Supplementary Table 1. Probe set signal data for experimental and control samples

PA2811_at	49.58	74.32	0.008	-1.50
PA2812_at	206.92	257.10	0.087	-1.24
PA2813_at	118.70	106.00	0.151	1.12
PA2814_at	10.38	10.02	0.643	1.04
PA2815_at	24.38	27.70	0.349	-1.14
PA2816_i_at	25.38	25.40	0.817	-1.00
PA2817_at	125.10	186.65	0.024	-1.49
PA2818_at	57.88	35.05	0.087	1.65
PA2819_at	16.56	6.75	0.024	2.45
PA2820_at	66.64	90.55	0.048	-1.36
PA2821_at	51.94	36.88	0.048	1.41
PA2822_at	43.60	49.57	0.151	-1.14
PA2823_at	205.64	214.38	0.484	-1.04
PA2824_at	39.12	27.75	0.087	1.41
PA2825_at	31.22	24.92	0.349	1.25
PA2826_at	87.44	77.40	0.190	1.13
PA2827_at	107.78	90.35	0.008	1.19
PA2828_at	232.22	263.88	0.048	-1.14
PA2829_at	62.46	73.77	0.238	-1.18
PA2830_htpX_at	603.96	393.88	0.024	1.53
PA2831_at	115.72	118.40	0.817	-1.02
PA2832_tpm_at	26.10	29.67	0.151	-1.14
PA2833_i_at	18.66	13.65	0.238	1.37
PA2834_at	19.56	30.05	0.087	-1.54
PA2835_at	8.88	11.20	0.238	-1.26
PA2836_at	9.78	9.48	0.817	1.03
PA2837_at	22.86	17.58	0.349	1.30
PA2838_at	7.64	11.52	0.143	-1.51
PA2839_at	29.24	20.35	0.024	1.44
PA2840_at	331.64	309.30	0.643	1.07
PA2841_at	44.30	23.25	0.008	1.91
PA2842_at	34.94	32.70	0.817	1.07
PA2843_at	219.84	160.57	0.048	1.37
PA2844_at	12.00	9.27	0.484	1.29
PA2845_at	16.78	9.77	0.024	1.72
PA2846_at	22.92	23.60	0.817	-1.03
PA2847_at	8.10	3.55	0.024	2.28
PA2848_at	26.66	38.95	0.008	-1.46
PA2849_at	49.74	92.65	0.008	-1.86
PA2850_ohr_at	81.80	111.70	0.151	-1.37
PA2851_efp_at	773.10	1357.25	0.008	-1.76
PA2852_at	34.82	38.53	0.643	-1.11
PA2853_oprI_at	1934.72	3002.38	0.024	-1.55
PA2854_at	207.86	323.48	0.008	-1.56
PA2855_at	128.88	155.57	0.087	-1.21
PA2856_tesA_at	64.68	58.38	0.484	1.11
PA2857_at	78.22	71.20	0.151	1.10
PA2858_at	111.98	78.95	0.008	1.42
PA2859_greB_at	53.18	64.20	0.048	-1.21
PA2860_at	49.32	49.70	1.000	-1.01
PA2861_ligT_at	12.18	5.78	0.286	2.11

Supplementary Table 1. Probe set signal data for experimental and control samples

PA2862_lipA_at	65.74	37.70	0.048	1.74
PA2863_lipH_at	2.92	10.95	0.008	-3.75
PA2864_at	14.94	40.55	0.008	-2.71
PA2865_at	47.96	49.17	0.817	-1.03
PA2866_mttC_at	167.66	171.48	1.000	-1.02
PA2867_at	407.88	291.90	0.024	1.40
PA2868_i_at	49.98	40.80	0.024	1.23
PA2869_at	7.26	13.57	0.087	-1.87
PA2870_at	21.14	18.58	0.484	1.14
PA2871_at	58.42	52.22	0.349	1.12
PA2872_at	41.30	34.50	0.151	1.20
PA2873_at	20.44	9.25	0.008	2.21
PA2874_at	46.80	27.03	0.008	1.73
PA2875_at	68.40	52.63	0.008	1.30
PA2876_pyrF_at	164.80	204.60	0.151	-1.24
PA2877_at	61.94	49.25	0.048	1.26
PA2878_at	13.44	15.15	1.000	-1.13
PA2879_at	48.54	53.45	0.349	-1.10
PA2880_at	11.56	18.58	0.151	-1.61
PA2881_at	10.24	16.88	0.087	-1.65
PA2882_at	17.36	22.25	0.087	-1.28
PA2883_at	41.92	33.20	0.151	1.26
PA2884_at	64.10	54.40	0.238	1.18
PA2885_at	49.78	59.82	0.008	-1.20
PA2886_at	34.20	34.42	0.643	-1.01
PA2887_at	34.40	33.90	1.000	1.01
PA2888_at	40.28	36.05	0.151	1.12
PA2889_at	43.04	35.40	0.008	1.22
PA2890_at	36.00	37.52	0.817	-1.04
PA2891_at	18.08	12.95	0.484	1.40
PA2892_at	33.88	26.10	0.238	1.30
PA2893_at	23.08	34.53	0.087	-1.50
PA2894_at	102.02	97.33	0.817	1.05
PA2895_at	61.54	53.45	0.087	1.15
PA2896_at	98.26	97.38	1.000	1.01
PA2897_at	133.80	145.58	0.643	-1.09
PA2898_at	11.66	14.72	0.349	-1.26
PA2899_at	67.66	52.83	0.151	1.28
PA2900_at	80.36	90.78	0.151	-1.13
PA2901_at	212.84	255.95	0.087	-1.20
PA2902_at	126.62	89.35	0.008	1.42
PA2903_cobJ_at	41.10	42.38	1.000	-1.03
PA2904_cobI_at	83.96	121.43	0.008	-1.45
PA2905_cobH_at	90.84	94.30	0.349	-1.04
PA2906_at	327.76	276.02	0.087	1.19
PA2907_cobL_at	158.10	112.72	0.008	1.40
PA2908_cbiD_at	109.58	92.18	0.151	1.19
PA2909_i_at	40.56	26.80	0.151	1.51
PA2910_at	19.12	13.18	0.238	1.45
PA2911_at	35.66	84.30	0.008	-2.36
PA2912_at	59.36	65.38	0.643	-1.10

Supplementary Table 1. Probe set signal data for experimental and control samples

PA2913_at	31.62	32.88	0.484	-1.04
PA2914_at	24.84	25.22	0.992	-1.02
PA2915_at	86.00	55.50	0.008	1.55
PA2916_at	24.92	14.85	0.151	1.68
PA2917_at	42.48	22.13	0.008	1.92
PA2918_at	63.16	59.05	0.643	1.07
PA2919_at	6.34	2.15	0.087	2.95
PA2920_at	33.88	20.25	0.087	1.67
PA2921_at	52.24	57.33	0.484	-1.10
PA2922_at	14.16	16.92	0.817	-1.19
PA2923_hisJ_at	20.76	16.60	0.484	1.25
PA2924_hisQ_at	6.96	6.75	1.000	1.03
PA2925_hisM_at	24.82	12.10	0.008	2.05
PA2926_hisP_at	3.36	7.07	0.087	-2.10
PA2927_at	19.78	8.50	0.008	2.33
PA2928_at	36.18	34.10	1.000	1.06
PA2929_at	20.44	16.28	0.151	1.26
PA2930_at	32.68	41.92	0.151	-1.28
PA2931_at	38.04	37.35	0.643	1.02
PA2932_morB_at	36.98	42.17	0.238	-1.14
PA2933_at	15.50	19.75	0.349	-1.27
PA2934_at	21.30	19.92	0.349	1.07
PA2935_at	6.34	2.45	0.151	2.59
PA2936_at	12.98	13.60	0.817	-1.05
PA2937_at	7.24	7.77	0.643	-1.07
PA2938_at	11.30	12.72	0.643	-1.13
PA2939_at	14.88	8.63	0.008	1.72
PA2940_at	8.44	16.15	0.151	-1.91
PA2941_at	36.52	25.70	0.151	1.42
PA2942_at	148.26	147.23	0.643	1.01
PA2943_at	85.10	74.45	0.484	1.14
PA2944_cobN_at	123.24	96.77	0.008	1.27
PA2945_at	533.62	453.25	0.087	1.18
PA2946_at	627.22	472.70	0.008	1.33
PA2947_i_at	65.94	74.35	0.484	-1.13
PA2948_cobM_at	75.36	65.05	0.484	1.16
PA2949_at	34.00	40.25	0.643	-1.18
PA2950_at	431.22	655.35	0.008	-1.52
PA2951_etfA_at	1624.76	1232.10	0.008	1.32
PA2952_etfB_at	2733.26	2256.30	0.008	1.21
PA2953_at	441.08	611.63	0.008	-1.39
PA2954_at	34.80	39.03	0.349	-1.12
PA2955_at	33.94	44.90	0.048	-1.32
PA2956_at	48.72	53.20	0.484	-1.09
PA2957_at	291.86	561.58	0.008	-1.92
PA2958_at	28.54	25.95	0.643	1.10
PA2959_at	244.22	241.90	1.000	1.01
PA2960_pilZ_at	113.90	211.60	0.008	-1.86
PA2961_holB_at	145.88	142.35	0.643	1.02
PA2962_tmk_at	213.82	220.07	0.643	-1.03
PA2963_at	120.10	126.15	0.349	-1.05

Supplementary Table 1. Probe set signal data for experimental and control samples

PA2964_pabC_at	245.54	299.90	0.151	-1.22
PA2965_fabF1_at	603.06	623.78	0.817	-1.03
PA2966_acpP_at	1035.40	1713.75	0.008	-1.66
PA2967_fabG_at	675.58	917.63	0.008	-1.36
PA2968_fabD_at	424.44	590.32	0.024	-1.39
PA2969_plsX_at	49.94	64.00	0.040	-1.28
PA2970_rpmF_at	632.26	1120.53	0.008	-1.77
PA2971_at	1539.56	1789.22	0.048	-1.16
PA2972_at	163.42	114.13	0.008	1.43
PA2973_at	583.52	442.20	0.008	1.32
PA2974_at	128.14	129.05	0.817	-1.01
PA2975_rluC_at	89.94	107.52	0.349	-1.20
PA2976_rne_at	2995.94	1906.15	0.008	1.57
PA2977_murB_at	110.74	86.98	0.111	1.27
PA2978_ptpA_at	128.64	162.68	0.008	-1.26
PA2979_kdsB_at	363.88	330.70	0.238	1.10
PA2980_at	394.06	408.65	0.643	-1.04
PA2981_lpxK_at	64.70	50.65	0.048	1.28
PA2982_at	85.58	102.13	0.087	-1.19
PA2983_at	279.48	243.05	0.048	1.15
PA2984_at	19.06	18.70	0.817	1.02
PA2985_at	101.98	77.10	0.008	1.32
PA2986_at	53.36	87.90	0.008	-1.65
PA2987_at	181.20	187.17	0.817	-1.03
PA2988_at	77.82	77.55	0.643	1.00
PA2989_at	55.44	44.50	0.151	1.25
PA2990_at	136.16	110.45	0.008	1.23
PA2991_sth_at	307.54	317.98	0.643	-1.03
PA2992_at	187.64	286.48	0.008	-1.53
PA2993_at	231.60	254.80	0.238	-1.10
PA2994_nqrF_at	329.48	389.08	0.024	-1.18
PA2995_nqrE_at	374.04	461.75	0.048	-1.23
PA2996_nqrD_at	501.54	544.38	0.238	-1.09
PA2997_nqrC_at	145.62	157.52	0.484	-1.08
PA2998_nqrB_at	330.62	399.22	0.024	-1.21
PA2999_nqrA_at	369.94	447.73	0.008	-1.21
PA3000_aroP1_at	39.84	67.82	0.024	-1.70
PA3001_at	2118.48	2107.97	1.000	1.00
PA3002_mfd_at	244.44	250.90	0.484	-1.03
PA3003_at	129.38	144.35	0.151	-1.12
PA3004_at	66.82	77.32	0.151	-1.16
PA3005_at	190.42	172.85	0.484	1.10
PA3006_at	84.42	80.02	1.000	1.05
PA3007_lexA_at	1267.90	320.93	0.008	3.95
PA3008_at	618.58	109.23	0.008	5.66
PA3009_at	75.32	233.80	0.008	-3.10
PA3010_at	128.06	153.73	0.024	-1.20
PA3011_topA_at	403.16	381.72	0.349	1.06
PA3012_at	57.96	73.35	0.048	-1.27
PA3013_foaB_at	128.20	182.50	0.048	-1.42
PA3014_faoA_at	324.86	397.90	0.024	-1.22

Supplementary Table 1. Probe set signal data for experimental and control samples

PA3015_at	61.74	50.20	0.151	1.23
PA3016_at	16.02	23.80	0.238	-1.49
PA3017_at	148.72	109.20	0.024	1.36
PA3018_at	32.58	50.17	0.048	-1.54
PA3019_at	87.24	109.23	0.238	-1.25
PA3020_at	86.62	73.90	0.048	1.17
PA3021_at	614.40	541.40	0.238	1.13
PA3022_at	153.08	110.97	0.008	1.38
PA3023_at	12.76	11.67	0.643	1.09
PA3024_at	12.14	13.43	0.484	-1.11
PA3025_at	30.80	27.90	0.349	1.10
PA3026_at	57.32	38.63	0.008	1.48
PA3027_at	160.02	125.65	0.087	1.27
PA3028_moeA2_at	82.88	64.78	0.032	1.28
PA3029_moaB2_at	397.34	356.88	0.484	1.11
PA3030_at	72.02	46.73	0.024	1.54
PA3031_at	254.80	432.75	0.048	-1.70
PA3032_at	22.86	18.70	0.238	1.22
PA3033_at	143.24	120.10	0.151	1.19
PA3034_at	68.70	72.88	0.643	-1.06
PA3035_at	79.22	102.97	0.111	-1.30
PA3036_at	20.66	26.65	0.048	-1.29
PA3037_at	21.88	31.15	0.048	-1.42
PA3038_at	758.38	448.13	0.048	1.69
PA3039_at	16.64	20.17	0.151	-1.21
PA3040_at	226.54	230.75	0.817	-1.02
PA3041_at	92.20	91.18	1.000	1.01
PA3042_at	53.90	39.15	0.024	1.38
PA3043_at	49.00	49.30	1.000	-1.01
PA3044_at	19.28	17.33	0.484	1.11
PA3045_at	12.24	7.60	0.151	1.61
PA3046_at	148.98	261.05	0.008	-1.75
PA3047_at	145.66	113.65	0.024	1.28
PA3048_at	46.70	64.03	0.048	-1.37
PA3049_rmf_at	19.60	16.90	0.643	1.16
PA3050_pyrD_at	254.00	351.50	0.008	-1.38
PA3051_at	14.30	30.20	0.048	-2.11
PA3052_at	164.36	86.60	0.008	1.90
PA3053_at	71.88	64.30	0.238	1.12
PA3054_at	61.76	69.57	0.349	-1.13
PA3055_at	172.46	165.58	0.817	1.04
PA3056_at	76.46	70.80	0.817	1.08
PA3057_at	17.36	12.77	0.024	1.36
PA3058_at	12.96	20.13	0.413	-1.55
PA3059_at	20.66	26.88	0.238	-1.30
PA3060_at	13.34	23.75	0.087	-1.78
PA3061_at	16.24	28.63	0.048	-1.76
PA3062_at	13.58	17.38	0.730	-1.28
PA3063_at	12.58	15.70	0.484	-1.25
PA3064_at	6.60	1.85	0.008	3.57
PA3065_at	11.28	8.45	0.413	1.33

Supplementary Table 1. Probe set signal data for experimental and control samples

PA3066_at	12.48	22.00	0.048	-1.76
PA3067_at	38.94	31.52	0.087	1.24
PA3068_at	2324.00	1466.85	0.008	1.58
PA3069_at	44.84	30.20	0.024	1.48
PA3070_at	187.22	173.93	0.238	1.08
PA3071_at	173.36	143.73	0.087	1.21
PA3072_at	68.78	91.20	0.024	-1.33
PA3073_at	37.70	35.85	0.484	1.05
PA3074_at	47.18	43.85	0.817	1.08
PA3075_at	29.34	32.55	0.349	-1.11
PA3076_at	66.76	52.72	0.024	1.27
PA3077_at	58.90	72.28	0.349	-1.23
PA3078_at	37.60	53.43	0.008	-1.42
PA3079_at	49.84	47.58	0.484	1.05
PA3080_at	205.76	77.63	0.008	2.65
PA3081_at	299.42	243.40	0.048	1.23
PA3082_at	243.98	351.60	0.024	-1.44
PA3083_pepN_at	94.92	140.60	0.008	-1.48
PA3084_at	167.58	171.20	0.817	-1.02
PA3085_at	138.48	167.45	0.087	-1.21
PA3086_at	29.34	43.03	0.008	-1.47
PA3087_at	47.40	51.23	0.817	-1.08
PA3088_at	156.80	180.73	0.087	-1.15
PA3089_at	34.76	22.88	0.048	1.52
PA3090_at	17.12	7.30	0.008	2.35
PA3091_at	54.92	37.55	0.087	1.46
PA3092_fadH1_at	38.90	49.33	0.151	-1.27
PA3093_at	143.60	117.45	0.151	1.22
PA3094_at	61.72	64.85	0.556	-1.05
PA3095_xcpZ_at	44.84	37.65	1.000	1.19
PA3096_xcpY_at	91.60	91.17	1.000	1.00
PA3097_xcpX_at	47.60	54.58	0.238	-1.15
PA3098_xcpW_at	92.16	67.18	0.024	1.37
PA3099_xcpV_at	110.58	80.92	0.024	1.37
PA3100_xcpU_at	96.76	88.65	0.087	1.09
PA3101_xcpT_at	218.24	217.82	0.817	1.00
PA3102_xcpS_at	68.56	63.70	0.484	1.08
PA3103_xcpR_at	138.16	109.85	0.024	1.26
PA3104_xcpP_at	163.60	146.42	0.238	1.12
PA3105_xcpQ_at	205.58	174.28	0.024	1.18
PA3106_at	105.60	145.45	0.008	-1.38
PA3107_metZ_at	520.96	541.20	0.349	-1.04
PA3108_purF_at	543.52	523.00	0.817	1.04
PA3109_at	85.34	95.30	0.643	-1.12
PA3110_at	246.28	331.05	0.008	-1.34
PA3111_folC_at	323.28	335.88	0.817	-1.04
PA3112_accD_at	321.24	342.42	0.484	-1.07
PA3113_trpF_at	372.24	302.72	0.008	1.23
PA3114_truA_at	93.44	108.00	0.151	-1.16
PA3115_at	645.06	584.85	0.048	1.10
PA3116_at	170.54	256.50	0.008	-1.50

Supplementary Table 1. Probe set signal data for experimental and control samples

PA3117_asd_at	241.18	267.73	0.349	-1.11
PA3118_leuB_at	122.36	133.50	0.817	-1.09
PA3119_at	54.52	49.20	0.238	1.11
PA3120_leuD_at	54.76	51.28	1.000	1.07
PA3121_leuC_at	56.52	50.83	0.349	1.11
PA3122_at	25.68	29.60	0.151	-1.15
PA3123_at	112.64	97.43	0.349	1.16
PA3124_at	61.16	60.28	0.817	1.01
PA3125_at	21.18	17.67	0.484	1.20
PA3126_ibpA_at	118.60	122.93	0.817	-1.04
PA3127_at	38.60	32.13	0.349	1.20
PA3128_at	51.38	28.10	0.079	1.83
PA3129_at	72.90	96.17	0.024	-1.32
PA3130_at	83.86	77.50	0.484	1.08
PA3131_at	149.20	111.28	0.048	1.34
PA3132_at	22.96	35.52	0.032	-1.55
PA3133_at	26.38	27.50	1.000	-1.04
PA3134_gltX_at	308.54	553.72	0.008	-1.79
PA3135_at	46.34	46.67	0.817	-1.01
PA3136_at	19.64	21.92	0.643	-1.12
PA3137_at	11.66	6.35	0.484	1.84
PA3138_uvrB_at	107.16	111.63	0.484	-1.04
PA3139_at	1159.72	1158.90	0.484	1.00
PA3140_at	15.36	6.47	0.008	2.37
PA3141_wbpM_g_at	119.46	138.90	0.238	-1.16
PA3142_i_at	41.06	51.52	0.238	-1.25
PA3143_at	44.26	38.20	0.413	1.16
PA3144_f_at	29.60	48.90	0.008	-1.65
PA3145_wbpL_at	55.14	99.45	0.008	-1.80
PA3146_wbpK_at	226.16	279.23	0.008	-1.23
PA3147_wbpJ_at	177.20	361.52	0.008	-2.04
PA3148_wbpI_at	103.88	154.50	0.024	-1.49
PA3149_wbpH_at	62.94	132.70	0.008	-2.11
PA3150_wbpG_at	51.10	115.27	0.008	-2.26
PA3151_hisF2_at	157.34	255.40	0.008	-1.62
PA3152_hisH2_at	70.62	135.65	0.008	-1.92
PA3153_wzx_at	12.04	18.22	0.151	-1.51
PA3154_wzy_at	20.58	21.10	0.643	-1.03
PA3155_wbpE_at	148.96	275.40	0.008	-1.85
PA3156_wbpD_at	124.18	197.10	0.008	-1.59
PA3157_at	38.46	50.90	0.024	-1.32
PA3158_wbpB_at	211.34	333.45	0.008	-1.58
PA3159_wbpA_at	440.94	523.00	0.048	-1.19
PA3160_wzz_at	80.54	98.97	0.151	-1.23
PA3161_himD_at	147.14	200.35	0.024	-1.36
PA3162_rpsA_at	2505.94	2475.75	0.817	1.01
PA3163_cmk_at	256.18	297.45	0.151	-1.16
PA3164_at	348.04	343.60	0.643	1.01
PA3165_hisC2_at	154.10	209.80	0.008	-1.36
PA3166_pheA_at	300.26	358.20	0.024	-1.19
PA3167_serC_at	353.44	486.45	0.008	-1.38

Supplementary Table 1. Probe set signal data for experimental and control samples

PA3168_gyrA_at	613.50	745.95	0.024	-1.22
PA3169_at	346.54	380.72	0.151	-1.10
PA3170_at	228.06	218.33	0.484	1.04
PA3171_ubiG_at	496.84	424.70	0.008	1.17
PA3172_at	464.60	397.42	0.087	1.17
PA3173_at	134.46	184.55	0.008	-1.37
PA3174_at	19.88	22.15	0.151	-1.11
PA3175_at	26.82	30.05	0.484	-1.12
PA3176_gltS_at	21.44	10.93	0.008	1.96
PA3177_at	118.80	104.80	0.643	1.13
PA3178_at	49.08	47.13	0.643	1.04
PA3179_at	269.30	478.95	0.008	-1.78
PA3180_at	6.88	15.55	0.008	-2.26
PA3181_at	66.28	90.30	0.349	-1.36
PA3182_at	259.04	280.05	0.643	-1.08
PA3183_zwf_at	244.94	285.55	0.484	-1.17
PA3184_at	66.12	78.30	0.151	-1.18
PA3185_at	133.00	105.53	0.087	1.26
PA3186_oprB_s_at	288.52	400.67	0.008	-1.39
PA3187_at	293.90	243.02	0.484	1.21
PA3188_at	118.76	121.25	1.000	-1.02
PA3189_at	98.54	79.57	0.484	1.24
PA3190_at	611.36	398.47	0.048	1.53
PA3191_at	31.08	23.13	0.048	1.34
PA3192_gltR_at	80.94	79.90	0.643	1.01
PA3193_glk_at	118.90	118.45	0.817	1.00
PA3194_edd_at	166.88	181.27	0.238	-1.09
PA3195_gapA_at	278.96	314.45	0.087	-1.13
PA3196_at	62.18	69.78	0.238	-1.12
PA3197_at	95.74	101.85	0.643	-1.06
PA3198_at	50.62	64.05	0.087	-1.27
PA3199_at	122.22	142.90	0.151	-1.17
PA3200_at	93.14	73.47	0.048	1.27
PA3201_at	339.88	300.33	0.151	1.13
PA3202_at	530.32	441.15	0.008	1.20
PA3203_at	360.84	301.58	0.008	1.20
PA3204_at	233.84	161.45	0.024	1.45
PA3205_at	340.96	145.52	0.151	2.34
PA3206_at	68.12	40.65	0.008	1.68
PA3207_at	32.02	43.85	0.063	-1.37
PA3208_at	115.70	92.97	0.008	1.24
PA3209_at	19.46	20.67	0.643	-1.06
PA3210_trkH_at	17.10	55.38	0.008	-3.24
PA3211_at	142.90	105.13	0.024	1.36
PA3212_at	185.86	129.85	0.024	1.43
PA3213_at	121.40	114.03	0.643	1.06
PA3214_at	100.82	86.28	0.048	1.17
PA3215_at	68.28	61.42	0.048	1.11
PA3216_at	21.62	11.82	0.008	1.83
PA3217_at	62.60	64.05	0.643	-1.02
PA3218_at	1.60	2.55	0.730	-1.59

Supplementary Table 1. Probe set signal data for experimental and control samples

PA3219_at	18.26	13.60	0.151	1.34
PA3220_at	84.98	84.38	0.817	1.01
PA3221_csaA_at	39.26	52.93	0.349	-1.35
PA3222_at	46.60	40.32	0.238	1.16
PA3223_acpD_at	17.64	14.10	0.484	1.25
PA3224_at	53.26	75.88	0.008	-1.42
PA3225_at	155.12	148.20	0.484	1.05
PA3226_at	81.40	79.75	0.817	1.02
PA3227_ppiA_at	540.86	507.18	0.238	1.07
PA3228_at	104.26	85.53	0.048	1.22
PA3229_at	21.96	21.70	1.000	1.01
PA3230_at	31.86	30.32	0.349	1.05
PA3231_at	14.12	3.30	0.008	4.28
PA3232_at	44.28	34.07	0.048	1.30
PA3233_at	47.04	25.70	0.048	1.83
PA3234_at	178.76	101.67	0.048	1.76
PA3235_at	184.88	94.60	0.048	1.95
PA3236_at	30.88	31.45	0.730	-1.02
PA3237_at	5.54	5.00	1.000	1.11
PA3238_at	137.38	102.75	0.048	1.34
PA3239_at	169.16	119.15	0.008	1.42
PA3240_at	28.34	36.08	0.151	-1.27
PA3241_at	38.70	36.63	0.484	1.06
PA3242_at	65.30	63.78	0.643	1.02
PA3243_minC_at	286.92	237.67	0.048	1.21
PA3244_minD_at	797.58	831.00	0.643	-1.04
PA3245_minE_at	314.58	431.75	0.008	-1.37
PA3246_rluA_at	211.70	267.30	0.048	-1.26
PA3247_at	120.34	128.25	0.349	-1.07
PA3248_at	37.10	32.13	0.349	1.15
PA3249_at	5.96	1.70	0.151	3.51
PA3250_at	59.42	52.40	0.151	1.13
PA3251_at	22.56	22.00	0.817	1.03
PA3252_at	16.08	9.52	0.111	1.69
PA3253_at	12.42	12.05	0.635	1.03
PA3254_at	23.58	28.00	0.349	-1.19
PA3255_at	113.00	103.83	0.238	1.09
PA3256_at	29.16	40.35	0.008	-1.38
PA3257_prc_at	387.74	332.10	0.349	1.17
PA3258_at	29.12	33.58	0.349	-1.15
PA3259_at	89.92	87.70	0.730	1.03
PA3260_at	126.30	146.85	0.151	-1.16
PA3261_at	25.70	25.05	1.000	1.03
PA3262_at	925.62	945.77	0.817	-1.02
PA3263_at	452.74	416.00	0.349	1.09
PA3264_at	41.66	46.08	0.492	-1.11
PA3265_at	20.72	26.88	0.048	-1.30
PA3266_capB_at	54.86	90.85	0.008	-1.66
PA3267_at	68.40	100.28	0.008	-1.47
PA3268_at	54.08	93.57	0.048	-1.73
PA3269_at	38.82	33.17	0.151	1.17

Supplementary Table 1. Probe set signal data for experimental and control samples

PA3270_at	256.66	228.95	0.484	1.12
PA3271_at	65.20	52.52	0.349	1.24
PA3272_at	60.82	40.00	0.024	1.52
PA3273_at	2.86	3.72	0.730	-1.30
PA3274_at	7.74	5.03	0.643	1.54
PA3275_at	52.90	31.85	0.024	1.66
PA3276_at	44.24	56.90	0.238	-1.29
PA3277_at	27.34	24.35	0.730	1.12
PA3278_at	48.48	49.07	0.817	-1.01
PA3279_oprP_at	4.46	10.65	0.024	-2.39
PA3280_oprO_at	21.92	27.68	0.151	-1.26
PA3281_at	58.74	26.45	0.008	2.22
PA3282_at	107.26	42.30	0.008	2.54
PA3283_at	258.28	82.68	0.008	3.12
PA3284_at	274.66	122.07	0.008	2.25
PA3285_at	192.30	200.00	0.817	-1.04
PA3286_at	434.02	428.18	1.000	1.01
PA3287_at	50.28	43.10	0.238	1.17
PA3288_at	32.48	34.00	0.817	-1.05
PA3289_at	22.34	16.83	0.087	1.33
PA3290_at	50.10	55.05	0.238	-1.10
PA3291_at	15.28	18.00	0.238	-1.18
PA3292_at	13.84	13.47	0.817	1.03
PA3293_at	46.88	33.17	0.008	1.41
PA3294_s_at	106.22	118.15	0.087	-1.11
PA3295_at	140.34	180.45	0.008	-1.29
PA3296_phoA_at	23.32	26.07	0.643	-1.12
PA3297_at	137.10	141.90	0.484	-1.04
PA3298_at	21.66	29.58	0.190	-1.37
PA3299_fadD1_at	482.06	478.80	0.817	1.01
PA3300_fadD2_at	62.62	42.42	0.008	1.48
PA3301_at	275.32	271.55	0.817	1.01
PA3302_at	125.00	178.35	0.008	-1.43
PA3303_at	17.96	12.40	0.349	1.45
PA3304_at	37.28	35.88	1.000	1.04
PA3305_at	39.02	27.75	0.087	1.41
PA3306_at	251.78	150.17	0.024	1.68
PA3307_r_at	78.84	61.05	0.238	1.29
PA3308_hepA_at	295.86	368.33	0.048	-1.24
PA3309_at	194.98	311.00	0.349	-1.60
PA3310_at	85.58	106.13	0.048	-1.24
PA3311_at	1.80	2.05	0.643	-1.14
PA3312_at	41.90	42.90	1.000	-1.02
PA3313_at	743.78	751.65	1.000	-1.01
PA3314_at	398.06	289.33	0.008	1.38
PA3315_at	32.80	39.35	0.151	-1.20
PA3316_at	34.90	42.20	0.008	-1.21
PA3317_at	77.32	77.80	0.643	-1.01
PA3318_at	20.72	23.43	0.349	-1.13
PA3319_plcN_at	5.74	8.30	0.151	-1.45
PA3320_at	7.10	5.80	0.817	1.22

Supplementary Table 1. Probe set signal data for experimental and control samples

PA3321_at	41.90	48.92	0.349	-1.17
PA3322_at	126.46	82.03	0.008	1.54
PA3323_at	34.18	33.15	0.817	1.03
PA3324_at	18.46	11.57	0.048	1.60
PA3325_at	13.46	13.67	0.730	-1.02
PA3326_at	558.56	464.58	0.087	1.20
PA3327_at	303.06	162.43	0.008	1.87
PA3328_at	143.86	77.85	0.008	1.85
PA3329_at	141.46	81.82	0.008	1.73
PA3330_at	163.72	84.73	0.008	1.93
PA3331_at	408.66	276.73	0.008	1.48
PA3332_at	322.84	214.45	0.008	1.51
PA3333_fabH2_at	196.74	120.72	0.008	1.63
PA3334_at	82.92	100.28	0.349	-1.21
PA3335_at	75.92	68.95	0.643	1.10
PA3336_at	23.14	25.90	0.643	-1.12
PA3337_rfaD_at	29.92	32.92	0.484	-1.10
PA3338_at	37.64	36.83	1.000	1.02
PA3339_at	76.32	66.53	0.238	1.15
PA3340_at	106.42	93.00	0.238	1.14
PA3341_at	93.74	124.25	0.151	-1.33
PA3342_at	22.58	20.40	0.643	1.11
PA3343_at	60.70	40.82	0.008	1.49
PA3344_recQ_at	139.06	134.07	0.817	1.04
PA3345_at	110.66	140.08	0.008	-1.27
PA3346_at	19.06	18.05	0.484	1.06
PA3347_at	46.26	39.42	0.151	1.17
PA3348_at	208.28	178.05	0.024	1.17
PA3349_at	295.96	268.10	0.238	1.10
PA3350_at	101.30	100.97	1.000	1.00
PA3351_at	1757.92	1394.52	0.048	1.26
PA3352_at	649.22	561.90	0.151	1.16
PA3353_at	242.12	187.45	0.151	1.29
PA3354_at	34.22	31.40	0.484	1.09
PA3355_at	65.22	33.80	0.008	1.93
PA3356_at	286.18	217.43	0.087	1.32
PA3357_dsdA_at	157.38	135.92	0.048	1.16
PA3358_at	23.44	29.75	0.349	-1.27
PA3359_at	7.24	4.80	0.048	1.51
PA3360_at	11.48	7.18	0.286	1.60
PA3361_at	23.54	28.08	0.484	-1.19
PA3362_at	22.46	16.45	0.238	1.37
PA3363_amiR_at	49.98	31.10	0.008	1.61
PA3364_amiC_at	54.92	32.72	0.008	1.68
PA3365_at	98.74	60.28	0.008	1.64
PA3366_amiE_at	64.36	54.18	0.349	1.19
PA3367_at	4.16	10.40	0.008	-2.50
PA3368_at	18.60	18.38	0.992	1.01
PA3369_at	54.44	43.85	0.024	1.24
PA3370_at	39.80	18.15	0.008	2.19
PA3371_at	42.86	33.25	0.238	1.29

Supplementary Table 1. Probe set signal data for experimental and control samples

PA3372_at	15.10	20.98	0.048	-1.39
PA3373_at	57.96	59.85	0.643	-1.03
PA3374_at	17.82	15.85	0.643	1.12
PA3375_at	12.66	16.42	0.349	-1.30
PA3376_at	2.08	1.78	0.341	1.17
PA3377_at	17.22	18.10	0.643	-1.05
PA3378_at	12.74	14.07	0.643	-1.10
PA3379_at	2.66	3.50	0.905	-1.32
PA3380_at	9.50	7.20	0.817	1.32
PA3381_at	22.96	25.47	0.643	-1.11
PA3382_phnE_at	2.80	7.00	0.190	-2.50
PA3383_at	10.88	11.42	0.643	-1.05
PA3384_phnC_at	5.22	16.85	0.008	-3.23
PA3385_at	2670.96	2186.13	0.643	1.22
PA3386_at	33.48	31.27	0.643	1.07
PA3387_rhlG_at	35.68	26.15	0.048	1.36
PA3388_at	149.96	112.75	0.024	1.33
PA3389_at	16.08	25.02	0.151	-1.56
PA3390_at	18.74	16.38	0.413	1.14
PA3391_nosR_at	6.84	7.98	0.556	-1.17
PA3392_nosZ_at	27.86	20.05	0.190	1.39
PA3393_nosD_at	11.34	28.48	0.008	-2.51
PA3394_nosF_at	11.78	12.72	0.643	-1.08
PA3395_nosY_at	10.68	11.70	0.817	-1.10
PA3396_nosL_at	5.92	8.45	0.238	-1.43
PA3397_fpr_at	221.66	290.97	0.087	-1.31
PA3398_at	55.16	70.47	0.048	-1.28
PA3399_at	40.92	29.03	0.048	1.41
PA3400_at	22.84	34.38	0.024	-1.51
PA3401_at	61.36	54.75	0.349	1.12
PA3402_at	58.78	63.00	0.484	-1.07
PA3403_at	49.52	81.78	0.008	-1.65
PA3404_at	47.40	22.17	0.008	2.14
PA3405_hasE_at	14.34	17.42	0.238	-1.21
PA3406_hasD_at	29.04	28.60	0.484	1.02
PA3407_hasAp_at	1.02	2.30	0.008	-2.25
PA3408_hasR_at	23.54	24.50	0.643	-1.04
PA3409_at	24.22	17.13	0.087	1.41
PA3410_at	43.30	61.43	0.008	-1.42
PA3411_r_at	44.90	59.13	0.151	-1.32
PA3412_at	12.46	11.02	0.413	1.13
PA3413_at	766.00	174.70	0.008	4.38
PA3414_at	459.46	47.00	0.008	9.78
PA3415_at	20.36	21.00	0.643	-1.03
PA3416_at	3.80	6.32	0.190	-1.66
PA3417_at	44.04	17.83	0.008	2.47
PA3418_ldh_at	23.96	20.15	0.349	1.19
PA3419_at	71.86	39.30	0.008	1.83
PA3420_at	7.94	11.75	0.048	-1.48
PA3421_at	12.06	11.07	0.817	1.09
PA3422_at	5.98	2.13	0.151	2.81

Supplementary Table 1. Probe set signal data for experimental and control samples

PA3423_at	44.68	41.35	0.643	1.08
PA3424_at	31.42	22.27	0.008	1.41
PA3425_at	26.76	31.35	0.238	-1.17
PA3426_at	32.38	26.75	0.635	1.21
PA3427_at	11.36	10.05	0.643	1.13
PA3428_at	11.38	10.70	1.000	1.06
PA3429_at	10.44	10.05	0.643	1.04
PA3430_at	19.74	10.90	0.008	1.81
PA3431_at	13.54	27.38	0.008	-2.02
PA3432_i_at	18.26	28.33	0.151	-1.55
PA3433_at	21.44	22.35	0.484	-1.04
PA3435_at	423.86	527.78	0.008	-1.25
PA3436_at	42.76	40.77	1.000	1.05
PA3437_at	158.20	137.40	0.151	1.15
PA3438_folE1_at	81.42	101.15	0.238	-1.24
PA3439_folX_at	227.64	204.42	0.484	1.11
PA3440_at	1720.28	1399.13	0.008	1.23
PA3441_at	7.22	17.33	0.048	-2.40
PA3442_at	10.02	17.50	0.238	-1.75
PA3443_at	20.60	13.68	0.349	1.51
PA3444_at	9.92	4.70	0.087	2.11
PA3445_at	7.84	7.70	0.817	1.02
PA3446_at	31.70	35.15	0.817	-1.11
PA3447_at	3.80	4.50	0.643	-1.18
PA3448_at	14.14	10.45	0.817	1.35
PA3449_at	5.22	4.50	0.643	1.16
PA3450_at	75.92	101.00	0.643	-1.33
PA3451_at	1.46	5.05	0.151	-3.46
PA3452_mqoA_at	264.20	352.20	0.048	-1.33
PA3453_at	208.40	250.25	0.238	-1.20
PA3454_at	20.82	23.10	0.484	-1.11
PA3455_at	85.40	70.10	0.238	1.22
PA3456_at	132.06	97.28	0.024	1.36
PA3457_at	28.90	31.42	0.643	-1.09
PA3458_at	29.28	47.60	0.048	-1.63
PA3459_at	52.98	28.20	0.008	1.88
PA3460_at	44.16	29.67	0.048	1.49
PA3461_at	19.90	19.05	0.484	1.04
PA3462_at	3.72	12.22	0.008	-3.28
PA3463_at	148.64	128.75	0.349	1.15
PA3464_at	23.12	19.50	0.349	1.19
PA3465_at	22.54	18.13	0.484	1.24
PA3466_at	104.46	144.10	0.048	-1.38
PA3467_at	8.82	8.13	0.817	1.08
PA3468_at	228.02	221.70	1.000	1.03
PA3469_at	158.54	130.48	0.008	1.22
PA3470_i_at	75.16	69.50	0.817	1.08
PA3471_at	269.70	181.27	0.048	1.49
PA3472_at	162.48	132.83	0.048	1.22
PA3473_at	34.62	34.05	0.817	1.02
PA3474_at	31.10	39.38	0.008	-1.27

Supplementary Table 1. Probe set signal data for experimental and control samples

PA3475_pheC_at	50.14	62.45	0.151	-1.25
PA3476_rhlL_at	244.06	224.18	0.151	1.09
PA3477_rhlR_at	152.30	165.75	0.349	-1.09
PA3478_rhlB_at	34.20	39.02	0.286	-1.14
PA3479_rhlA_at	82.78	70.83	0.087	1.17
PA3480_at	806.66	627.13	0.008	1.29
PA3481_at	391.34	339.63	0.151	1.15
PA3482_metG_at	980.46	915.28	0.151	1.07
PA3483_at	229.24	237.00	0.643	-1.03
PA3484_at	217.04	241.35	0.349	-1.11
PA3485_r_at	108.34	123.65	0.190	-1.14
PA3486_at	45.30	65.78	0.024	-1.45
PA3487_at	95.98	99.90	0.817	-1.04
PA3488_at	177.26	164.43	0.643	1.08
PA3489_at	99.02	122.10	0.008	-1.23
PA3490_at	85.16	102.82	0.484	-1.21
PA3491_at	235.92	279.83	0.087	-1.19
PA3492_at	49.26	53.97	0.349	-1.10
PA3493_at	42.48	49.00	0.151	-1.15
PA3494_at	60.50	67.45	0.484	-1.11
PA3495_nth_at	79.12	107.72	0.008	-1.36
PA3496_at	73.36	173.32	0.008	-2.36
PA3497_at	12.86	17.02	0.349	-1.32
PA3498_at	12.74	16.75	0.151	-1.31
PA3499_at	33.16	38.75	0.087	-1.17
PA3500_at	36.54	37.03	0.817	-1.01
PA3501_at	24.80	36.00	0.048	-1.45
PA3502_at	31.36	28.53	0.484	1.10
PA3503_at	29.54	26.07	0.484	1.13
PA3504_at	27.44	18.15	0.048	1.51
PA3505_at	24.38	26.52	0.484	-1.09
PA3506_at	25.94	12.10	0.008	2.14
PA3507_at	28.70	15.15	0.008	1.89
PA3508_at	30.38	18.83	0.048	1.61
PA3509_at	34.72	27.05	0.151	1.28
PA3510_at	46.52	25.72	0.008	1.81
PA3511_at	55.96	32.38	0.024	1.73
PA3512_at	25.90	16.33	0.024	1.59
PA3513_at	15.84	6.88	0.087	2.30
PA3514_at	32.10	23.20	0.048	1.38
PA3515_at	5.82	7.60	0.349	-1.31
PA3516_at	16.68	10.52	0.151	1.59
PA3517_at	17.02	21.70	0.238	-1.27
PA3518_at	10.98	8.95	0.643	1.23
PA3519_at	12.92	12.88	0.468	1.00
PA3520_at	10.28	9.23	0.635	1.11
PA3521_at	2.42	3.63	0.063	-1.50
PA3522_at	19.22	11.30	0.151	1.70
PA3523_at	11.98	3.85	0.151	3.11
PA3524_gloA1_at	393.84	637.38	0.024	-1.62
PA3525_argG_at	599.38	962.75	0.008	-1.61

Supplementary Table 1. Probe set signal data for experimental and control samples

PA3526_at	144.92	110.22	0.151	1.31
PA3527_pyrC_at	390.64	476.80	0.008	-1.22
PA3528_rnt_at	515.96	412.33	0.087	1.25
PA3529_at	1051.56	1289.03	0.151	-1.23
PA3530_at	27.84	50.83	0.024	-1.83
PA3531_bfrB_at	113.96	227.50	0.008	-2.00
PA3532_at	27.78	28.45	0.349	-1.02
PA3533_at	490.32	684.65	0.048	-1.40
PA3534_at	28.74	17.17	0.151	1.67
PA3535_at	170.62	106.72	0.008	1.60
PA3536_at	64.10	61.38	0.484	1.04
PA3537_argF_at	393.34	362.03	0.643	1.09
PA3538_at	166.34	152.88	0.238	1.09
PA3539_at	89.00	119.60	0.008	-1.34
PA3540_algD_at	8.40	9.68	0.643	-1.15
PA3541_at	6.88	5.95	0.730	1.16
PA3542_at	22.18	27.10	0.349	-1.22
PA3543_algK_at	4.98	2.80	0.484	1.78
PA3544_algE_at	22.32	16.17	0.238	1.38
PA3545_algG_at	9.54	10.52	0.817	-1.10
PA3546_algX_at	18.56	22.35	0.349	-1.20
PA3547_algL_at	2.14	12.33	0.008	-5.76
PA3548_algI_at	12.58	8.05	0.063	1.56
PA3549_algJ_at	24.98	22.30	0.817	1.12
PA3550_algF_at	13.12	17.88	0.349	-1.36
PA3551_algA_at	34.14	36.20	1.000	-1.06
PA3552_at	716.64	448.57	0.008	1.60
PA3553_at	407.32	288.63	0.008	1.41
PA3554_at	671.30	443.10	0.008	1.52
PA3555_at	108.76	75.97	0.048	1.43
PA3556_at	216.02	218.70	0.349	-1.01
PA3557_at	56.06	66.43	0.048	-1.18
PA3558_at	163.98	177.77	0.238	-1.08
PA3559_at	251.60	155.25	0.024	1.62
PA3560_fruA_at	43.30	36.63	0.048	1.18
PA3561_fruK_at	90.28	88.50	0.643	1.02
PA3562_at	71.06	73.90	0.817	-1.04
PA3563_fruR_at	152.02	165.15	0.817	-1.09
PA3564_at	11.04	12.52	0.817	-1.13
PA3565_at	28.90	27.27	1.000	1.06
PA3566_at	170.00	154.70	0.087	1.10
PA3567_at	133.06	133.48	0.643	-1.00
PA3568_at	10.08	13.02	0.349	-1.29
PA3569_mmsB_at	14.12	13.57	1.000	1.04
PA3570_mmsA_at	40.50	25.70	0.008	1.58
PA3571_mmsR_at	28.94	23.60	0.238	1.23
PA3572_at	47.28	74.22	0.238	-1.57
PA3573_at	30.26	23.48	0.238	1.29
PA3574_at	38.10	88.58	0.008	-2.32
PA3575_at	249.42	148.70	0.024	1.68
PA3576_at	64.26	60.20	0.817	1.07

Supplementary Table 1. Probe set signal data for experimental and control samples

PA3577_i_at	11.34	30.25	0.008	-2.67
PA3578_at	31.12	40.05	0.238	-1.29
PA3579_at	40.50	32.92	0.349	1.23
PA3580_at	76.10	87.82	0.151	-1.15
PA3581_glpF_at	13.98	30.45	0.024	-2.18
PA3582_glpK_at	36.62	75.57	0.048	-2.06
PA3583_glpR_at	154.28	139.95	0.238	1.10
PA3584_glpD_at	21.84	34.10	0.008	-1.56
PA3585_glpM_at	55.50	70.75	0.087	-1.27
PA3586_at	18.62	14.15	0.817	1.32
PA3587_metR_at	96.48	89.95	0.643	1.07
PA3588_at	9.90	8.43	0.817	1.17
PA3589_at	3.04	2.48	0.817	1.23
PA3590_at	13.04	18.90	0.484	-1.45
PA3591_at	12.32	5.75	0.048	2.14
PA3592_at	10.70	17.63	0.087	-1.65
PA3593_at	24.32	17.23	0.024	1.41
PA3594_at	38.84	35.75	0.349	1.09
PA3595_at	6.62	9.32	0.349	-1.41
PA3596_at	14.12	10.65	0.643	1.33
PA3597_at	10.90	10.55	1.000	1.03
PA3598_at	9.54	14.95	0.286	-1.57
PA3599_at	30.12	30.10	0.484	1.00
PA3600_at	14.60	12.75	0.817	1.15
PA3601_at	26.36	28.83	0.817	-1.09
PA3602_at	310.44	181.82	0.008	1.71
PA3603_dgkA_at	82.62	91.65	0.484	-1.11
PA3604_at	376.88	367.35	0.643	1.03
PA3605_at	43.98	39.28	0.238	1.12
PA3606_at	59.28	59.93	0.817	-1.01
PA3607_potA_at	65.80	143.67	0.008	-2.18
PA3608_potB_at	25.84	64.60	0.008	-2.50
PA3609_potC_at	40.00	82.98	0.008	-2.07
PA3610_potD_at	35.38	105.85	0.008	-2.99
PA3611_at	454.28	538.40	0.151	-1.19
PA3612_at	73.36	134.35	0.008	-1.83
PA3613_at	63.82	78.77	1.000	-1.23
PA3614_at	103.56	101.72	0.817	1.02
PA3615_at	120.42	84.68	0.151	1.42
PA3616_at	289.72	105.23	0.008	2.75
PA3617_recA_at	1917.54	706.10	0.008	2.72
PA3618_at	224.80	167.47	0.008	1.34
PA3619_at	19.60	17.20	0.484	1.14
PA3620_mutS_at	332.94	387.13	0.238	-1.16
PA3621_fdxA_at	321.70	648.60	0.008	-2.02
PA3622_rpoS_at	1175.56	742.13	0.008	1.58
PA3623_at	110.72	100.18	0.151	1.11
PA3624_pcm_at	379.26	369.15	0.817	1.03
PA3625_surE_at	844.86	758.58	0.048	1.11
PA3626_at	190.62	230.90	0.008	-1.21
PA3627_at	344.04	330.27	0.643	1.04

Supplementary Table 1. Probe set signal data for experimental and control samples

PA3628_at	194.32	233.88	0.349	-1.20
PA3629_adhC_at	198.40	229.47	0.238	-1.16
PA3630_at	16.32	10.05	0.238	1.62
PA3631_at	159.24	229.88	0.008	-1.44
PA3632_at	207.62	280.90	0.087	-1.35
PA3633_at	172.68	206.27	0.087	-1.19
PA3634_at	210.88	337.08	0.008	-1.60
PA3635_eno_at	866.24	1031.65	0.087	-1.19
PA3636_kdsA_at	292.52	478.13	0.008	-1.63
PA3637_pyrG_at	1663.94	1427.97	0.048	1.17
PA3638_at	67.02	76.30	0.484	-1.14
PA3639_accA_at	324.78	416.05	0.048	-1.28
PA3640_dnaE_at	383.58	371.95	0.349	1.03
PA3641_at	343.90	659.55	0.008	-1.92
PA3642_rnhB_at	86.86	123.75	0.048	-1.42
PA3643_lpxB_at	302.98	337.45	0.484	-1.11
PA3644_lpxA_at	520.78	661.45	0.024	-1.27
PA3645_fabZ_at	606.84	932.13	0.008	-1.54
PA3646_lpxD_at	820.48	928.95	0.349	-1.13
PA3647_at	1128.00	1284.47	0.484	-1.14
PA3648_at	350.64	483.28	0.048	-1.38
PA3649_at	89.42	108.28	0.024	-1.21
PA3650_dxr_at	180.92	211.25	0.151	-1.17
PA3651_cdsA_at	101.06	163.95	0.008	-1.62
PA3652_uppS_at	360.78	475.93	0.024	-1.32
PA3653_frr_at	1054.52	1551.15	0.008	-1.47
PA3654_pyrH_at	1029.92	1334.82	0.024	-1.30
PA3655_tsf_at	1050.40	1602.80	0.008	-1.53
PA3656_rpsB_at	2912.16	3356.13	0.087	-1.15
PA3657_map_at	815.82	923.73	0.151	-1.13
PA3658_glnD_at	116.84	99.80	0.238	1.17
PA3659_at	298.06	334.30	0.087	-1.12
PA3660_at	48.74	48.70	0.802	1.00
PA3661_at	130.78	115.40	0.349	1.13
PA3662_at	292.16	210.35	0.349	1.39
PA3663_at	21.50	38.27	0.008	-1.78
PA3664_at	500.66	435.73	0.008	1.15
PA3665_at	616.62	517.20	0.024	1.19
PA3666_dapD_at	801.14	612.68	0.008	1.31
PA3667_at	80.38	96.10	0.238	-1.20
PA3668_at	47.02	60.38	0.048	-1.28
PA3669_at	24.46	10.72	0.008	2.28
PA3670_at	77.02	54.80	0.048	1.41
PA3671_at	47.48	28.58	0.087	1.66
PA3672_at	161.22	81.05	0.008	1.99
PA3673_plsB_at	208.36	192.35	0.484	1.08
PA3674_at	284.30	259.95	0.238	1.09
PA3675_at	193.38	202.30	0.817	-1.05
PA3676_at	56.84	51.00	0.349	1.11
PA3677_at	28.00	22.40	0.024	1.25
PA3678_at	41.48	37.70	0.484	1.10

Supplementary Table 1. Probe set signal data for experimental and control samples

PA3679_at	49.52	59.28	0.151	-1.20
PA3680_at	68.00	81.70	0.238	-1.20
PA3681_at	23.96	18.30	0.024	1.31
PA3682_at	16.16	19.92	0.349	-1.23
PA3683_at	58.30	51.63	0.238	1.13
PA3684_i_at	84.34	118.00	0.024	-1.40
PA3685_at	79.48	93.25	0.087	-1.17
PA3686_adk_at	621.48	743.78	0.151	-1.20
PA3687_ppc_at	145.34	77.10	0.008	1.89
PA3688_at	9.70	19.40	0.151	-2.00
PA3689_at	113.84	83.30	0.048	1.37
PA3690_at	73.10	64.77	0.349	1.13
PA3691_at	484.44	309.00	0.008	1.57
PA3692_at	190.80	120.65	0.024	1.58
PA3693_at	72.26	66.25	0.643	1.09
PA3694_at	136.56	149.80	0.238	-1.10
PA3695_at	115.42	125.75	0.349	-1.09
PA3696_at	60.18	59.50	0.643	1.01
PA3697_at	209.58	172.95	0.008	1.21
PA3698_at	74.16	70.40	1.000	1.05
PA3699_at	140.48	134.48	0.643	1.04
PA3700_lysS_at	857.24	1089.20	0.048	-1.27
PA3701_prfB_at	623.06	738.55	0.087	-1.19
PA3702_at	94.26	91.55	0.484	1.03
PA3703_at	102.88	110.15	0.484	-1.07
PA3704_at	76.30	76.68	1.000	-1.00
PA3705_at	173.30	151.73	0.349	1.14
PA3706_at	35.06	36.55	0.817	-1.04
PA3707_at	48.22	42.15	0.151	1.14
PA3708_at	97.80	93.43	0.349	1.05
PA3709_at	13.26	4.63	0.238	2.86
PA3710_at	28.16	23.30	0.349	1.21
PA3711_at	34.22	24.52	0.151	1.40
PA3712_at	425.68	169.70	0.008	2.51
PA3713_at	42.76	89.93	0.008	-2.10
PA3714_at	30.70	37.70	0.048	-1.23
PA3715_at	28.72	36.42	0.238	-1.27
PA3716_at	416.68	423.22	0.817	-1.02
PA3717_at	118.96	136.70	0.151	-1.15
PA3718_at	14.22	12.95	0.484	1.10
PA3719_at	31.02	33.60	0.484	-1.08
PA3720_at	36.74	32.33	0.643	1.14
PA3721_at	20.70	22.83	0.484	-1.10
PA3722_at	234.62	246.90	1.000	-1.05
PA3723_at	35.26	37.55	0.817	-1.06
PA3724_lasB_at	134.06	119.88	0.484	1.12
PA3725_recJ_at	130.92	135.52	0.643	-1.04
PA3726_at	147.46	190.63	0.238	-1.29
PA3727_at	73.58	104.40	0.024	-1.42
PA3728_at	136.42	141.95	0.643	-1.04
PA3729_at	510.68	535.35	0.643	-1.05

Supplementary Table 1. Probe set signal data for experimental and control samples

PA3730_at	127.86	138.32	1.000	-1.08
PA3731_at	596.68	577.63	1.000	1.03
PA3732_at	403.08	445.45	0.151	-1.11
PA3733_at	46.26	32.38	0.024	1.43
PA3734_at	25.64	19.20	0.151	1.34
PA3735_thrC_at	303.80	352.15	0.238	-1.16
PA3736_hom_at	1053.10	832.85	0.008	1.26
PA3737_dsbC_at	301.48	336.75	0.238	-1.12
PA3738_xerD_at	119.56	89.68	0.008	1.33
PA3739_at	44.16	38.70	0.349	1.14
PA3740_at	41.48	35.63	0.143	1.16
PA3741_at	60.48	74.77	0.349	-1.24
PA3742_rplS_at	1066.34	1191.25	0.048	-1.12
PA3743_trmD_at	1705.14	2371.48	0.008	-1.39
PA3744_rimM_at	1906.40	2043.15	0.238	-1.07
PA3745_rpsP_at	2813.42	2683.05	0.643	1.05
PA3746_ffh_at	318.50	447.63	0.024	-1.41
PA3747_at	303.10	264.47	0.024	1.15
PA3748_at	178.78	126.73	0.008	1.41
PA3749_at	8.40	10.33	0.151	-1.23
PA3750_at	51.26	50.88	0.817	1.01
PA3751_purT_at	87.66	108.03	0.048	-1.23
PA3752_at	220.40	202.60	0.286	1.09
PA3753_at	146.84	171.70	0.048	-1.17
PA3754_at	137.34	124.23	0.151	1.11
PA3755_at	93.74	87.15	0.643	1.08
PA3756_at	403.02	424.38	0.484	-1.05
PA3757_at	65.86	82.72	0.349	-1.26
PA3758_at	67.64	89.88	0.087	-1.33
PA3759_at	55.48	63.28	0.349	-1.14
PA3760_at	21.24	27.20	0.286	-1.28
PA3761_at	26.30	18.90	0.484	1.39
PA3762_at	48.42	37.47	0.008	1.29
PA3763_purL_at	389.10	529.30	0.024	-1.36
PA3764_at	94.02	122.17	0.024	-1.30
PA3765_at	22.98	27.48	0.349	-1.20
PA3766_at	50.96	50.80	0.817	1.00
PA3767_at	38.50	83.45	0.008	-2.17
PA3768_at	339.84	397.80	0.238	-1.17
PA3769_guaA_at	670.24	850.53	0.087	-1.27
PA3770_guaB_at	685.82	797.18	0.151	-1.16
PA3771_at	6.54	4.55	1.000	1.44
PA3772_at	10.88	10.85	0.817	1.00
PA3773_at	22.28	11.95	0.151	1.86
PA3774_at	17.46	14.05	0.635	1.24
PA3775_at	8.98	9.70	0.643	-1.08
PA3776_at	22.24	31.58	0.087	-1.42
PA3777_xseA_at	141.22	94.18	0.008	1.50
PA3778_at	44.04	50.28	0.238	-1.14
PA3779_at	212.10	114.18	0.008	1.86
PA3780_at	74.52	39.95	0.024	1.87

Supplementary Table 1. Probe set signal data for experimental and control samples

PA3781_at	79.50	46.97	0.008	1.69
PA3782_at	37.58	30.43	0.817	1.23
PA3783_at	39.46	32.47	0.349	1.22
PA3784_at	76.92	73.05	1.000	1.05
PA3785_at	108.86	109.23	0.643	-1.00
PA3786_at	151.68	93.75	0.008	1.62
PA3787_at	232.26	103.57	0.008	2.24
PA3788_at	59.84	51.22	0.643	1.17
PA3789_at	25.20	20.90	0.484	1.21
PA3790_oprC_at	163.16	108.43	0.048	1.50
PA3791_at	80.18	77.55	0.817	1.03
PA3792_leuA_at	445.54	311.85	0.008	1.43
PA3793_at	136.26	176.00	0.024	-1.29
PA3794_at	142.26	172.07	0.087	-1.21
PA3795_at	101.52	61.88	0.087	1.64
PA3796_at	74.20	75.55	1.000	-1.02
PA3797_at	180.64	159.68	0.349	1.13
PA3798_at	179.46	172.83	0.643	1.04
PA3799_at	173.94	295.80	0.008	-1.70
PA3800_at	342.28	545.40	0.008	-1.59
PA3801_at	1021.46	975.13	0.484	1.05
PA3802_hisS_at	468.08	591.13	0.048	-1.26
PA3803_at	612.50	656.92	0.238	-1.07
PA3804_at	331.88	344.73	0.643	-1.04
PA3805_pilF_at	116.00	143.63	0.087	-1.24
PA3806_at	976.20	1019.28	0.484	-1.04
PA3807_ndk_at	1949.36	1995.23	0.817	-1.02
PA3808_at	123.58	243.98	0.008	-1.97
PA3809_fdx2_at	202.62	330.95	0.048	-1.63
PA3810_hscA_at	122.52	168.00	0.024	-1.37
PA3811_hscB_at	288.06	413.35	0.008	-1.43
PA3812_iscA_at	878.40	1042.08	0.024	-1.19
PA3813_iscU_at	496.36	664.80	0.008	-1.34
PA3814_iscS_at	1392.24	1480.57	0.024	-1.06
PA3815_at	1031.14	822.22	0.087	1.25
PA3816_cysE_at	203.14	202.05	0.817	1.01
PA3817_at	90.32	114.75	0.087	-1.27
PA3818_at	283.10	484.92	0.008	-1.71
PA3819_at	294.60	241.10	0.151	1.22
PA3820_secF_at	301.76	392.70	0.008	-1.30
PA3821_secD_at	886.66	1009.88	0.151	-1.14
PA3822_at	1226.26	1367.68	0.024	-1.12
PA3823_tgt_at	171.88	254.83	0.008	-1.48
PA3824_queA_at	202.84	250.10	0.024	-1.23
PA3825_at	8.16	14.68	0.087	-1.80
PA3826_at	32.54	41.65	0.040	-1.28
PA3827_at	167.48	204.07	0.048	-1.22
PA3828_at	281.06	318.17	0.087	-1.13
PA3829_at	16.50	9.32	0.151	1.77
PA3830_at	26.32	21.47	0.190	1.23
PA3831_pepA_at	610.70	512.05	0.238	1.19

Supplementary Table 1. Probe set signal data for experimental and control samples

PA3832_holC_at	225.80	183.73	0.048	1.23
PA3833_at	306.00	330.75	0.349	-1.08
PA3834_valS_at	775.32	905.40	0.008	-1.17
PA3835_at	28.86	20.20	0.048	1.43
PA3836_at	654.62	438.63	0.008	1.49
PA3837_at	408.98	242.85	0.008	1.68
PA3838_at	184.42	152.27	0.238	1.21
PA3839_at	15.44	24.27	0.238	-1.57
PA3840_at	39.10	44.88	0.238	-1.15
PA3841_exoS_at	49.64	53.20	0.643	-1.07
PA3842_at	84.08	112.52	0.087	-1.34
PA3843_at	18.60	11.27	0.024	1.65
PA3844_at	33.56	33.80	0.484	-1.01
PA3845_at	46.64	48.38	0.643	-1.04
PA3846_at	30.56	24.70	0.484	1.24
PA3847_at	57.84	58.10	1.000	-1.00
PA3848_at	142.30	96.50	0.008	1.47
PA3849_at	98.34	157.23	0.008	-1.60
PA3850_at	347.16	302.15	0.151	1.15
PA3851_at	22.36	31.68	0.024	-1.42
PA3852_at	57.20	75.77	0.008	-1.32
PA3853_at	60.88	69.30	0.238	-1.14
PA3854_at	161.66	152.93	0.643	1.06
PA3855_at	63.88	53.83	0.349	1.19
PA3856_at	38.44	35.97	0.643	1.07
PA3857_at	151.22	174.45	0.151	-1.15
PA3858_at	85.90	50.10	0.008	1.71
PA3859_at	154.04	130.32	0.151	1.18
PA3860_at	50.46	44.22	0.238	1.14
PA3861_rhlB_at	371.80	465.05	0.024	-1.25
PA3862_at	722.76	463.88	0.008	1.56
PA3863_at	211.60	171.00	0.087	1.24
PA3864_at	87.04	121.38	0.008	-1.39
PA3865_at	138.44	109.75	0.151	1.26
PA3866_at	129.64	58.85	0.008	2.20
PA3867_at	20.04	17.10	0.643	1.17
PA3868_at	18.28	14.33	0.643	1.28
PA3869_at	15.14	22.40	0.238	-1.48
PA3870_moaA1_at	8.32	10.73	0.643	-1.29
PA3871_at	11.70	12.38	0.643	-1.06
PA3872_narI_at	34.96	37.10	0.556	-1.06
PA3873_narJ_at	22.60	22.17	1.000	1.02
PA3874_narH_at	10.96	14.10	0.484	-1.29
PA3875_narG_at	3.88	4.32	1.000	-1.11
PA3876_narK2_at	9.56	11.18	0.643	-1.17
PA3877_narK1_at	8.96	16.02	0.151	-1.79
PA3878_narX_at	92.02	109.90	0.643	-1.19
PA3879_narL_at	81.18	115.55	0.238	-1.42
PA3880_at	128.68	167.73	0.238	-1.30
PA3881_at	131.72	97.45	0.024	1.35
PA3882_at	60.00	54.25	0.349	1.11

Supplementary Table 1. Probe set signal data for experimental and control samples

PA3883_at	68.82	64.85	0.984	1.06
PA3884_at	22.32	16.05	0.238	1.39
PA3885_at	13.78	15.13	0.151	-1.10
PA3886_at	107.30	72.70	0.008	1.48
PA3887_nhaP_at	111.30	119.95	0.151	-1.08
PA3888_at	30.74	31.30	0.730	-1.02
PA3889_at	31.42	30.65	0.905	1.03
PA3890_at	13.00	14.52	0.484	-1.12
PA3891_at	27.12	32.92	0.484	-1.21
PA3892_at	12.02	33.05	0.008	-2.75
PA3893_at	32.70	42.47	0.238	-1.30
PA3894_at	49.48	63.28	0.048	-1.28
PA3895_at	140.42	140.43	0.484	-1.00
PA3896_at	123.30	105.93	0.349	1.16
PA3897_at	4.66	2.20	0.048	2.12
PA3898_at	66.74	67.22	1.000	-1.01
PA3899_at	30.40	31.80	0.643	-1.05
PA3900_at	8.16	31.13	0.008	-3.81
PA3901_fecA_at	438.82	205.20	0.008	2.14
PA3902_at	87.42	86.57	1.000	1.01
PA3903_prfC_at	449.16	545.13	0.048	-1.21
PA3904_i_at	816.82	1219.27	0.008	-1.49
PA3905_at	136.50	200.60	0.008	-1.47
PA3906_at	471.30	518.33	0.484	-1.10
PA3907_at	367.28	465.00	0.008	-1.27
PA3908_at	130.72	189.82	0.008	-1.45
PA3909_at	16.58	12.10	0.817	1.37
PA3910_at	16.18	11.58	0.151	1.40
PA3911_at	43.04	47.28	0.643	-1.10
PA3912_at	21.58	26.38	0.643	-1.22
PA3913_at	21.14	44.35	0.024	-2.10
PA3914_moeA1_at	10.76	1.55	0.238	6.94
PA3915_moaB1_at	8.86	19.02	0.087	-2.15
PA3916_moaE_at	76.02	98.03	0.048	-1.29
PA3917_moaD_at	153.18	125.83	0.048	1.22
PA3918_moaC_at	283.58	253.05	0.238	1.12
PA3919_at	46.96	34.53	0.087	1.36
PA3920_at	16.98	16.20	0.556	1.05
PA3921_at	63.72	34.85	0.008	1.83
PA3922_at	92.94	47.58	0.008	1.95
PA3923_at	150.04	46.17	0.008	3.25
PA3924_at	63.50	37.50	0.008	1.69
PA3925_at	106.66	146.38	0.008	-1.37
PA3926_at	10.00	10.92	0.238	-1.09
PA3927_at	12.52	10.88	0.151	1.15
PA3928_r_at	18.66	18.85	1.000	-1.01
PA3929_cioB_at	19.62	20.95	0.905	-1.07
PA3930_cioA_at	56.00	47.60	0.238	1.18
PA3931_at	80.52	141.80	0.817	-1.76
PA3932_at	16.76	23.13	0.286	-1.38
PA3933_at	9.98	18.15	0.008	-1.82

PA3934_at	57.60	83.95	0.048	-1.46
PA3935_tauD_at	36.54	29.92	0.087	1.22
PA3936_at	20.84	20.50	0.817	1.02
PA3937_at	22.02	23.03	1.000	-1.05
PA3938_at	25.12	25.52	1.000	-1.02
PA3939_at	20.36	13.02	0.151	1.56
PA3940_at	854.58	1516.23	0.008	-1.77
PA3941_at	55.50	80.18	0.008	-1.44
PA3942_tesB_at	166.84	178.55	0.484	-1.07
PA3943_at	56.94	53.17	0.817	1.07
PA3944_at	82.24	68.55	0.087	1.20
PA3945_at	65.20	40.50	0.048	1.61
PA3946_at	14.08	16.48	0.349	-1.17
PA3947_at	23.38	24.25	0.817	-1.04
PA3948_at	44.36	77.13	0.008	-1.74
PA3949_at	115.92	95.73	0.349	1.21
PA3950_at	158.90	160.27	0.817	-1.01
PA3951_at	87.32	86.80	0.643	1.01
PA3952_at	141.90	95.38	0.151	1.49
PA3953_at	45.64	28.47	0.048	1.60
PA3954_at	22.66	18.55	0.151	1.22
PA3955_at	78.70	79.95	0.643	-1.02
PA3956_at	30.28	26.52	0.484	1.14
PA3957_at	2.96	5.07	0.151	-1.71
PA3958_at	67.00	68.07	1.000	-1.02
PA3959_at	49.84	32.15	0.008	1.55
PA3960_at	54.00	38.25	0.063	1.41
PA3961_at	97.42	97.30	1.000	1.00
PA3962_at	197.16	214.20	0.349	-1.09
PA3963_at	24.08	20.00	0.230	1.20
PA3964_at	19.26	12.90	0.087	1.49
PA3965_at	83.74	123.92	0.024	-1.48
PA3966_at	132.34	204.73	0.008	-1.55
PA3967_at	92.82	233.88	0.008	-2.52
PA3968_at	58.58	56.72	0.817	1.03
PA3969_at	91.92	61.78	0.008	1.49
PA3970_amn_at	181.24	155.20	0.151	1.17
PA3971_at	21.00	17.95	0.151	1.17
PA3972_at	65.84	63.65	0.643	1.03
PA3973_at	63.24	48.28	0.151	1.31
PA3974_at	32.16	32.33	0.238	-1.01
PA3975_thiD_at	676.22	478.17	0.008	1.41
PA3976_thiE_at	652.16	583.53	0.151	1.12
PA3977_hemL_at	1432.28	1094.80	0.008	1.31
PA3978_at	252.70	295.25	0.484	-1.17
PA3979_at	48.36	85.85	0.008	-1.78
PA3980_at	497.88	534.23	0.484	-1.07
PA3981_at	534.08	534.25	1.000	-1.00
PA3982_at	583.18	502.75	0.008	1.16
PA3983_at	33.28	62.65	0.008	-1.88
PA3984_lnt_at	111.54	116.75	0.349	-1.05

Supplementary Table 1. Probe set signal data for experimental and control samples

PA3985_at	25.68	22.98	0.643	1.12
PA3986_at	14.38	16.75	0.238	-1.16
PA3987_leuS_at	831.70	791.03	0.643	1.05
PA3988_at	1170.44	915.42	0.008	1.28
PA3989_holA_at	299.06	332.97	1.000	-1.11
PA3990_at	74.70	73.53	0.817	1.02
PA3991_at	6.20	5.60	0.484	1.11
PA3992_at	125.40	107.43	0.063	1.17
PA3994_at	12.28	6.03	0.008	2.04
PA3995_at	16.94	22.98	0.151	-1.36
PA3996_lipA_at	248.92	300.27	0.048	-1.21
PA3997_lipB_at	411.92	319.95	0.008	1.29
PA3998_at	619.18	587.67	0.484	1.05
PA3999_dacC_at	1196.14	1123.15	0.349	1.06
PA4000_at	181.90	196.95	0.349	-1.08
PA4001_mltB2_at	228.18	220.10	0.643	1.04
PA4002_rodA_at	115.08	106.60	0.238	1.08
PA4003_pbpA_at	149.26	136.90	0.238	1.09
PA4004_at	451.32	441.30	0.484	1.02
PA4005_at	239.52	308.70	0.008	-1.29
PA4006_at	541.64	558.50	0.817	-1.03
PA4007_proA_at	444.84	508.60	0.048	-1.14
PA4008_at	14.26	11.68	0.484	1.22
PA4009_at	15.10	10.32	0.024	1.46
PA4010_at	314.36	319.08	0.643	-1.02
PA4011_at	199.54	228.00	0.024	-1.14
PA4012_at	80.58	87.05	0.349	-1.08
PA4013_at	59.58	63.00	0.643	-1.06
PA4014_at	184.84	164.50	0.286	1.12
PA4015_at	315.00	230.68	0.008	1.37
PA4016_at	31.46	24.98	0.238	1.26
PA4017_at	88.86	80.72	0.817	1.10
PA4018_r_at	40.44	38.45	1.000	1.05
PA4019_at	123.72	138.78	0.048	-1.12
PA4020_mpl_at	338.46	286.28	0.008	1.18
PA4021_at	75.98	47.97	0.008	1.58
PA4023_at	284.94	162.63	0.024	1.75
PA4024_eutB_at	527.40	332.55	0.024	1.59
PA4025_r_at	240.44	157.93	0.008	1.52
PA4026_at	63.38	57.55	0.484	1.10
PA4027_at	47.56	45.80	0.643	1.04
PA4028_i_at	22.68	26.50	0.484	-1.17
PA4029_at	318.42	311.85	0.817	1.02
PA4030_at	343.76	294.10	0.008	1.17
PA4031_ppa_at	1586.76	2055.98	0.008	-1.30
PA4032_at	78.30	90.52	0.087	-1.16
PA4033_at	11.68	11.50	0.817	1.02
PA4034_aqpZ_at	5.70	15.15	0.087	-2.66
PA4035_at	147.84	104.92	0.008	1.41
PA4036_at	26.70	30.27	0.643	-1.13
PA4037_at	28.34	20.98	0.190	1.35

Supplementary Table 1. Probe set signal data for experimental and control samples

PA4038_at	16.58	9.85	0.087	1.68
PA4039_at	15.16	15.57	0.817	-1.03
PA4040_at	36.64	23.08	0.048	1.59
PA4041_at	14.32	3.85	0.024	3.72
PA4042_xseB_at	777.80	469.08	0.008	1.66
PA4043_ispA_at	396.22	287.38	0.008	1.38
PA4044_dxs_at	333.42	346.72	0.643	-1.04
PA4045_at	110.10	90.47	0.151	1.22
PA4046_at	63.24	80.63	0.048	-1.27
PA4047_ribA_at	282.32	291.93	0.817	-1.03
PA4048_at	48.60	44.65	0.484	1.09
PA4049_at	116.92	112.07	0.643	1.04
PA4050_pgpA_at	111.76	171.02	0.008	-1.53
PA4051_thiL_at	286.92	317.83	0.151	-1.11
PA4052_nusB_at	435.14	496.45	0.349	-1.14
PA4053_ribE_at	779.50	1047.72	0.008	-1.34
PA4054_ribB_at	835.60	766.78	0.238	1.09
PA4055_ribC_at	346.70	363.85	0.349	-1.05
PA4056_ribD_at	189.84	189.80	1.000	1.00
PA4057_at	709.72	460.00	0.008	1.54
PA4058_at	243.22	211.25	0.349	1.15
PA4059_at	376.38	362.78	0.643	1.04
PA4060_at	142.20	202.90	0.024	-1.43
PA4061_at	597.10	495.15	0.048	1.21
PA4062_at	7.50	11.75	0.151	-1.57
PA4063_at	85.94	58.05	0.008	1.48
PA4064_at	35.16	27.80	0.151	1.26
PA4065_at	25.78	37.65	0.349	-1.46
PA4066_at	25.38	29.22	0.238	-1.15
PA4067_oprG_at	1062.64	1244.40	0.349	-1.17
PA4068_at	131.62	77.88	0.008	1.69
PA4069_at	373.76	263.98	0.008	1.42
PA4070_at	12.96	9.40	0.643	1.38
PA4071_at	2.22	3.70	0.484	-1.67
PA4072_at	12.26	10.32	0.643	1.19
PA4073_at	40.80	44.52	0.643	-1.09
PA4074_at	4.12	7.68	0.087	-1.86
PA4075_at	34.50	28.33	0.190	1.22
PA4076_at	47.54	58.07	0.111	-1.22
PA4077_at	22.24	25.85	0.484	-1.16
PA4078_at	19.74	14.23	0.286	1.39
PA4079_at	105.36	109.90	0.817	-1.04
PA4080_at	13.26	15.85	0.151	-1.20
PA4081_at	4.44	11.70	0.024	-2.64
PA4082_at	11.60	8.55	0.349	1.36
PA4083_at	8.24	17.08	0.024	-2.07
PA4084_at	4.00	3.15	1.000	1.27
PA4085_at	4.62	5.92	0.238	-1.28
PA4086_at	20.00	33.88	0.008	-1.69
PA4087_at	15.04	18.63	0.817	-1.24
PA4088_at	13.24	11.80	1.000	1.12

Supplementary Table 1. Probe set signal data for experimental and control samples

PA4089_at	2.26	2.83	0.825	-1.25
PA4090_at	163.38	205.55	0.151	-1.26
PA4091_hpaA_at	13.74	22.85	0.087	-1.66
PA4092_hpaC_at	12.88	10.32	0.643	1.25
PA4093_at	18.62	9.30	0.087	2.00
PA4094_at	87.10	55.22	0.008	1.58
PA4095_at	22.36	20.13	0.817	1.11
PA4096_at	1.82	7.65	0.008	-4.20
PA4097_at	18.34	18.45	1.000	-1.01
PA4098_at	23.78	15.25	0.238	1.56
PA4099_at	17.38	7.65	0.048	2.27
PA4100_at	14.88	16.95	0.643	-1.14
PA4101_at	61.64	45.67	0.087	1.35
PA4102_at	51.14	46.45	0.643	1.10
PA4103_at	5.68	8.63	0.230	-1.52
PA4104_at	35.94	27.75	0.151	1.30
PA4105_at	4.66	7.90	0.238	-1.70
PA4106_at	16.18	12.58	0.643	1.29
PA4107_at	23.74	18.98	0.484	1.25
PA4108_at	34.62	25.93	0.238	1.34
PA4109_ampR_at	76.36	70.50	0.817	1.08
PA4110_ampC_at	48.46	42.38	0.556	1.14
PA4111_i_at	84.66	69.77	0.151	1.21
PA4112_at	31.70	27.88	0.635	1.14
PA4113_at	31.48	36.38	0.349	-1.16
PA4114_at	78.30	79.30	0.643	-1.01
PA4115_at	202.62	157.73	0.008	1.28
PA4116_at	234.66	191.03	0.008	1.23
PA4117_at	75.68	86.28	0.238	-1.14
PA4118_at	60.58	62.97	0.484	-1.04
PA4119_aph_at	46.52	65.48	0.008	-1.41
PA4120_at	14.40	20.67	0.151	-1.44
PA4121_at	41.90	62.78	0.048	-1.50
PA4122_at	32.60	33.00	0.817	-1.01
PA4123_hpcC_at	22.10	33.48	0.016	-1.51
PA4124_hpcB_at	13.82	28.20	0.008	-2.04
PA4125_hpcD_at	36.64	35.10	0.817	1.04
PA4126_at	12.78	16.75	0.238	-1.31
PA4127_hpcG_at	12.98	11.05	0.810	1.17
PA4128_at	9.68	21.88	0.024	-2.26
PA4129_at	21.30	31.08	0.349	-1.46
PA4130_at	57.30	80.55	0.238	-1.41
PA4131_at	175.70	358.40	0.087	-2.04
PA4132_at	33.04	71.30	0.048	-2.16
PA4133_at	46.84	106.58	0.024	-2.28
PA4134_i_at	10.24	12.05	0.476	-1.18
PA4135_at	77.08	133.40	0.008	-1.73
PA4136_at	23.78	29.80	0.349	-1.25
PA4137_at	6.96	4.28	0.643	1.63
PA4138_tyrS_at	52.10	55.73	0.817	-1.07
PA4139_at	18.26	25.63	0.087	-1.40

Supplementary Table 1. Probe set signal data for experimental and control samples

PA4140_at	6.02	21.45	0.008	-3.56
PA4141_at	39.98	83.30	0.008	-2.08
PA4142_at	26.00	27.93	0.484	-1.07
PA4143_at	11.62	22.40	0.087	-1.93
PA4144_at	6.30	9.32	0.349	-1.48
PA4145_at	34.56	32.18	0.817	1.07
PA4146_at	22.18	15.33	0.151	1.45
PA4147_acoR_at	10.74	14.20	0.484	-1.32
PA4148_at	6.12	9.45	0.238	-1.54
PA4149_at	14.80	9.75	0.238	1.52
PA4150_at	20.84	15.17	0.484	1.37
PA4151_acoB_at	6.40	10.05	0.413	-1.57
PA4152_at	19.42	19.00	1.000	1.02
PA4153_at	3.78	7.70	0.087	-2.04
PA4154_at	62.04	41.00	0.143	1.51
PA4155_at	22.64	16.20	0.190	1.40
PA4156_at	3.22	16.32	0.008	-5.07
PA4157_at	53.80	54.72	0.817	-1.02
PA4158_fepC_at	6.20	3.83	0.643	1.62
PA4159_fepB_at	22.02	15.13	0.238	1.46
PA4160_fepD_at	8.50	13.15	0.349	-1.55
PA4161_fepG_at	13.38	16.03	0.817	-1.20
PA4162_at	81.48	50.88	0.008	1.60
PA4163_at	486.34	376.88	0.008	1.29
PA4164_at	125.04	133.17	0.643	-1.07
PA4165_at	23.06	18.15	0.151	1.27
PA4166_at	14.24	14.05	1.000	1.01
PA4167_at	5.02	4.05	0.476	1.24
PA4168_at	23.90	16.80	0.048	1.42
PA4169_at	10.66	11.57	0.484	-1.09
PA4170_at	14.78	18.35	0.817	-1.24
PA4171_at	17.88	27.98	0.151	-1.56
PA4172_at	16.86	16.35	1.000	1.03
PA4173_at	11.14	18.55	0.024	-1.67
PA4174_at	36.20	27.73	0.151	1.31
PA4175_at	68.82	75.00	0.484	-1.09
PA4176_ppiC2_at	197.74	242.82	0.048	-1.23
PA4177_at	11.44	19.08	0.087	-1.67
PA4178_at	12.30	23.13	0.048	-1.88
PA4179_at	6.62	40.08	0.008	-6.05
PA4180_at	171.52	125.53	0.008	1.37
PA4181_at	139.12	80.18	0.008	1.74
PA4182_at	134.86	86.70	0.008	1.56
PA4183_at	27.16	30.40	0.413	-1.12
PA4184_at	57.30	46.42	0.151	1.23
PA4185_at	136.44	123.77	0.008	1.10
PA4186_at	21.74	26.02	0.484	-1.20
PA4187_at	6.66	9.85	0.341	-1.48
PA4188_at	20.96	14.20	0.048	1.48
PA4189_at	8.18	5.60	0.349	1.46
PA4190_at	192.04	145.13	0.008	1.32

Supplementary Table 1. Probe set signal data for experimental and control samples

PA4191_at	18.90	24.60	0.151	-1.30
PA4192_at	16.68	28.47	0.087	-1.71
PA4193_at	12.82	25.52	0.151	-1.99
PA4194_at	4.08	20.30	0.024	-4.98
PA4195_at	22.30	47.30	0.024	-2.12
PA4196_at	52.04	48.60	0.643	1.07
PA4197_at	40.44	38.00	0.484	1.06
PA4198_at	123.50	90.57	0.087	1.36
PA4199_at	221.22	95.72	0.008	2.31
PA4200_at	47.96	59.45	0.349	-1.24
PA4201_ddlA_at	45.04	36.63	0.032	1.23
PA4202_at	42.22	48.35	0.238	-1.15
PA4203_at	25.18	26.42	1.000	-1.05
PA4204_at	53.50	53.60	0.817	-1.00
PA4205_at	11.06	22.15	0.024	-2.00
PA4206_at	37.26	28.25	0.349	1.32
PA4207_at	27.46	28.73	0.643	-1.05
PA4208_at	29.82	37.63	0.048	-1.26
PA4209_at	33.38	31.35	0.817	1.06
PA4210_s_at	7.86	8.25	0.817	-1.05
PA4211_g_at	69.78	89.57	0.238	-1.28
PA4217_at	64.52	69.13	0.484	-1.07
PA4218_at	76.72	202.15	0.008	-2.63
PA4219_at	34.88	88.53	0.008	-2.54
PA4220_i_at	93.82	180.85	0.008	-1.93
PA4221_fptA_at	154.60	417.85	0.008	-2.70
PA4222_at	86.22	182.30	0.008	-2.11
PA4223_at	95.32	247.35	0.008	-2.59
PA4224_at	108.04	262.38	0.008	-2.43
PA4225_pchF_at	42.06	138.98	0.008	-3.30
PA4226_pchE_at	103.26	262.97	0.008	-2.55
PA4227_pchR_at	48.00	50.65	0.643	-1.06
PA4228_pchD_at	101.10	235.10	0.008	-2.33
PA4229_pchC_at	101.20	267.57	0.008	-2.64
PA4230_pchB_at	49.60	212.05	0.008	-4.28
PA4231_pchA_at	35.62	102.60	0.008	-2.88
PA4232_ssb_at	1404.06	1334.25	0.238	1.05
PA4233_at	153.48	152.98	0.643	1.00
PA4234_uvrA_at	566.02	472.53	0.024	1.20
PA4235_bfrA_at	337.92	423.70	0.087	-1.25
PA4236_katA_at	543.84	275.05	0.008	1.98
PA4237_rplQ_at	3033.60	2430.35	0.151	1.25
PA4238_rpoA_at	5010.22	4699.45	0.238	1.07
PA4239_rpsD_at	2400.30	2721.05	0.087	-1.13
PA4240_rpsK_at	3079.78	3477.32	0.087	-1.13
PA4241_rpsM_at	3455.08	3729.13	0.151	-1.08
PA4242_rpmJ_at	3890.50	4444.57	0.238	-1.14
PA4243_secY_at	2101.52	3048.18	0.008	-1.45
PA4244_rplO_at	4440.62	3762.63	0.087	1.18
PA4245_rpmD_at	3092.32	3435.60	0.151	-1.11
PA4246_rpsE_at	5106.94	4906.97	0.643	1.04

Supplementary Table 1. Probe set signal data for experimental and control samples

PA4247_rplR_at	4062.82	4190.88	0.643	-1.03
PA4248_rplF_at	4566.36	4002.28	0.151	1.14
PA4249_rpsH_at	5027.46	4745.72	0.643	1.06
PA4250_rpsN_at	3012.66	3142.75	0.349	-1.04
PA4251_rplE_at	6128.40	5275.95	0.238	1.16
PA4252_rplX_at	3276.02	3350.40	0.643	-1.02
PA4253_rplN_at	5149.66	4199.85	0.008	1.23
PA4254_rpsQ_at	6390.00	6072.00	0.484	1.05
PA4255_rpmC_at	1113.52	1394.28	0.048	-1.25
PA4256_rplP_at	4291.40	3968.80	0.484	1.08
PA4257_rpsC_at	3249.28	3317.20	0.643	-1.02
PA4258_rplV_at	6125.44	5046.80	0.008	1.21
PA4259_rpsS_at	3069.18	3465.63	0.048	-1.13
PA4260_rplB_at	6447.30	4698.20	0.024	1.37
PA4261_rplW_at	4185.52	3716.85	0.151	1.13
PA4262_rplD_at	4233.26	3943.32	0.484	1.07
PA4263_rplC_at	5144.92	4261.45	0.048	1.21
PA4264_rpsJ_at	4258.80	4191.70	0.817	1.02
PA4265_tufA_s_at	2034.94	2877.58	0.008	-1.41
PA4266_fusA1_at	2720.94	2721.23	0.817	-1.00
PA4267_rpsG_at	5378.78	4245.30	0.024	1.27
PA4268_rpsL_at	2580.60	3094.78	0.087	-1.20
PA4269_rpoC_at	2950.68	2974.10	0.484	-1.01
PA4270_rpoB_at	956.18	1221.22	0.151	-1.28
PA4271_rplL_at	3448.22	3855.50	0.349	-1.12
PA4272_rplJ_at	4671.44	4460.95	0.643	1.05
PA4273_rplA_at	4555.16	4159.80	0.151	1.10
PA4274_rplK_at	5438.42	4486.55	0.087	1.21
PA4275_nusG_at	570.58	1009.13	0.008	-1.77
PA4276_secE_at	1189.90	1900.88	0.048	-1.60
PA4278_at	36.92	57.85	0.008	-1.57
PA4279_at	123.84	206.37	0.008	-1.67
PA4280_birA_at	237.44	273.58	0.063	-1.15
PA4281_sbcD_at	151.58	93.77	0.008	1.62
PA4282_at	88.18	52.22	0.008	1.69
PA4283_recD_at	62.76	61.38	0.484	1.02
PA4284_recB_at	206.76	164.03	0.024	1.26
PA4285_recC_at	107.72	79.38	0.048	1.36
PA4286_at	218.16	175.38	0.008	1.24
PA4287_at	14.18	10.23	0.008	1.39
PA4288_at	42.52	28.45	0.008	1.49
PA4289_at	31.82	22.83	0.238	1.39
PA4290_at	154.66	99.35	0.151	1.56
PA4291_at	55.52	82.00	0.048	-1.48
PA4292_at	189.24	333.80	0.008	-1.76
PA4293_at	16.24	6.58	0.087	2.47
PA4294_at	12.26	17.90	0.048	-1.46
PA4295_at	6.72	4.85	0.643	1.39
PA4296_at	106.02	76.32	0.087	1.39
PA4297_at	3.14	1.85	0.230	1.70
PA4298_at	14.56	10.57	0.484	1.38

Supplementary Table 1. Probe set signal data for experimental and control samples

PA4299_at	26.84	23.80	0.643	1.13
PA4300_at	7.82	16.08	0.087	-2.06
PA4301_at	15.60	19.27	0.151	-1.24
PA4302_at	2.32	8.95	0.008	-3.86
PA4303_at	19.44	12.95	0.238	1.50
PA4304_at	15.04	6.15	0.024	2.45
PA4305_at	8.46	16.98	0.151	-2.01
PA4306_at	10.16	10.13	0.730	1.00
PA4307_pctC_at	409.44	316.35	0.048	1.29
PA4308_at	67.60	44.50	0.024	1.52
PA4309_pctA_at	125.38	114.60	0.238	1.09
PA4310_pctB_at	507.48	547.20	0.349	-1.08
PA4311_at	27.00	16.08	0.016	1.68
PA4312_at	85.32	88.38	1.000	-1.04
PA4313_at	34.92	33.70	0.817	1.04
PA4314_purU1_at	349.72	392.27	0.151	-1.12
PA4315_mvaT_at	1272.26	2350.00	0.087	-1.85
PA4316_sbcB_at	119.74	113.50	1.000	1.05
PA4317_at	1311.62	1128.07	0.349	1.16
PA4318_at	228.92	195.00	0.238	1.17
PA4319_at	91.16	97.70	0.349	-1.07
PA4320_at	99.68	86.78	0.349	1.15
PA4321_at	274.24	233.10	0.048	1.18
PA4322_at	192.66	244.97	0.151	-1.27
PA4323_at	189.96	171.13	0.349	1.11
PA4324_at	109.82	85.88	0.238	1.28
PA4325_at	111.76	112.83	1.000	-1.01
PA4326_at	59.42	59.35	1.000	1.00
PA4327_at	37.60	31.20	0.643	1.21
PA4328_at	65.60	62.87	0.484	1.04
PA4329_pykA_at	1080.40	725.62	0.008	1.49
PA4330_at	30.00	33.38	0.349	-1.11
PA4331_at	55.78	44.70	0.087	1.25
PA4332_at	38.46	46.13	0.238	-1.20
PA4333_at	774.32	1114.38	0.008	-1.44
PA4334_at	30.32	30.85	1.000	-1.02
PA4335_at	41.66	25.63	0.008	1.63
PA4336_at	227.02	169.55	0.048	1.34
PA4337_at	29.12	24.85	0.349	1.17
PA4338_at	23.00	19.58	0.643	1.17
PA4339_at	49.32	38.80	0.151	1.27
PA4340_at	101.02	90.27	0.048	1.12
PA4341_at	9.04	5.57	0.643	1.62
PA4342_at	21.12	21.08	0.817	1.00
PA4343_at	3.36	2.03	0.024	1.66
PA4344_at	19.78	16.88	0.484	1.17
PA4345_at	16.88	16.42	0.341	1.03
PA4346_at	7.40	6.47	0.817	1.14
PA4347_at	37.68	35.17	0.484	1.07
PA4348_at	130.22	172.98	0.238	-1.33
PA4349_at	62.20	39.65	0.008	1.57

Supplementary Table 1. Probe set signal data for experimental and control samples

PA4350_at	19.68	15.65	0.349	1.26
PA4351_at	7.02	7.38	0.817	-1.05
PA4352_at	76.56	122.35	0.024	-1.60
PA4353_at	36.54	34.53	0.643	1.06
PA4354_at	56.14	73.88	0.151	-1.32
PA4355_at	58.20	58.10	0.817	1.00
PA4356_xenB_at	89.24	116.47	0.087	-1.31
PA4357_r_at	44.44	42.42	0.484	1.05
PA4358_at	38.36	54.88	0.087	-1.43
PA4359_i_at	31.38	39.25	0.151	-1.25
PA4360_at	605.66	540.42	0.087	1.12
PA4361_at	22.56	25.02	0.484	-1.11
PA4362_at	18.24	14.47	0.349	1.26
PA4363_iciA_at	10.04	19.13	0.008	-1.91
PA4364_at	26.76	16.98	0.008	1.58
PA4365_at	13.20	10.57	0.413	1.25
PA4366_sodB_at	1779.44	1923.32	0.484	-1.08
PA4367_at	63.60	55.00	0.048	1.16
PA4368_at	27.62	29.25	0.151	-1.06
PA4369_at	39.26	33.03	0.087	1.19
PA4370_at	1924.68	1805.90	1.000	1.07
PA4371_at	140.62	186.63	0.087	-1.33
PA4372_at	477.94	649.17	0.087	-1.36
PA4373_at	109.34	139.57	0.087	-1.28
PA4374_at	85.56	82.02	0.643	1.04
PA4375_at	24.28	25.60	0.817	-1.05
PA4376_pncB2_at	138.22	135.18	0.817	1.02
PA4377_at	65.88	47.58	0.048	1.38
PA4378_inaA_at	46.78	50.33	0.349	-1.08
PA4379_at	123.56	89.38	0.048	1.38
PA4380_at	44.74	39.35	0.087	1.14
PA4381_at	98.58	89.35	0.238	1.10
PA4382_at	19.36	15.80	0.484	1.23
PA4383_at	34.34	32.03	0.643	1.07
PA4384_at	31.58	43.13	0.048	-1.37
PA4385_groEL_at	2903.34	2964.98	0.643	-1.02
PA4386_groES_at	6642.66	5044.48	0.008	1.32
PA4387_at	146.80	163.80	0.349	-1.12
PA4388_at	97.22	99.58	0.817	-1.02
PA4389_at	117.82	166.70	0.024	-1.41
PA4390_at	48.44	117.05	0.008	-2.42
PA4391_at	37.12	35.88	0.730	1.03
PA4392_at	24.86	30.15	0.238	-1.21
PA4393_at	59.92	45.22	0.024	1.33
PA4394_at	94.90	130.15	0.087	-1.37
PA4395_at	690.36	804.05	0.349	-1.16
PA4396_at	66.54	64.38	0.817	1.03
PA4397_panE_at	54.66	52.18	0.643	1.05
PA4398_at	76.70	59.35	0.087	1.29
PA4399_at	87.10	90.47	1.000	-1.04
PA4400_at	90.08	96.77	0.484	-1.07

Supplementary Table 1. Probe set signal data for experimental and control samples

PA4401_at	88.12	125.45	0.024	-1.42
PA4402_argJ_at	181.38	234.93	0.024	-1.30
PA4403_secA_at	580.56	641.83	0.087	-1.11
PA4404_at	100.82	93.52	0.349	1.08
PA4405_at	31.16	37.15	0.087	-1.19
PA4406_lpxC_at	602.96	788.72	0.024	-1.31
PA4407_ftsZ_at	1133.68	1331.50	0.048	-1.17
PA4408_ftsA_at	302.20	335.93	0.008	-1.11
PA4409_ftsQ_at	351.60	354.67	1.000	-1.01
PA4410_ddlB_at	269.50	255.65	0.484	1.05
PA4411_murC_at	422.94	437.23	0.238	-1.03
PA4412_murG_at	392.84	321.10	0.151	1.22
PA4413_ftsW_at	284.66	236.23	0.048	1.21
PA4414_murD_at	226.02	186.85	0.151	1.21
PA4415_mraY_at	249.54	274.32	0.238	-1.10
PA4416_murF_at	360.22	275.95	0.008	1.31
PA4417_murE_at	218.56	202.45	0.151	1.08
PA4418_ftsI_at	123.04	129.00	0.484	-1.05
PA4419_ftsL_at	105.42	106.82	1.000	-1.01
PA4420_at	250.66	253.00	0.817	-1.01
PA4421_at	299.06	339.30	0.238	-1.13
PA4422_at	96.96	140.32	0.008	-1.45
PA4423_at	727.12	551.53	0.008	1.32
PA4424_at	424.30	501.08	0.008	-1.18
PA4425_at	1008.32	891.40	0.024	1.13
PA4426_at	360.44	406.22	0.087	-1.13
PA4427_sspB_at	107.02	210.47	0.008	-1.97
PA4428_sspA_at	276.04	516.38	0.008	-1.87
PA4429_at	596.10	964.70	0.087	-1.62
PA4430_at	1221.72	1540.03	0.151	-1.26
PA4431_at	3504.86	3078.35	0.151	1.14
PA4432_rpsI_at	493.84	1156.88	0.008	-2.34
PA4433_rplM_at	2525.88	3859.70	0.008	-1.53
PA4434_at	130.58	115.53	0.643	1.13
PA4435_at	16.38	12.25	0.151	1.34
PA4436_at	123.76	96.25	0.484	1.29
PA4437_at	20.26	41.97	0.008	-2.07
PA4438_at	251.06	371.05	0.008	-1.48
PA4439_trpS_at	317.76	343.42	0.484	-1.08
PA4440_at	199.58	181.23	0.238	1.10
PA4441_at	1630.14	1649.43	0.817	-1.01
PA4442_cysN_at	259.76	321.85	0.349	-1.24
PA4443_cysD_at	387.74	616.03	0.008	-1.59
PA4444_mltB1_at	92.74	88.40	0.556	1.05
PA4445_at	89.38	87.78	0.643	1.02
PA4446_algW_at	132.64	129.88	0.635	1.02
PA4447_hisC1_at	103.24	171.63	0.008	-1.66
PA4448_hisD_at	187.98	160.90	0.190	1.17
PA4449_hisG_at	404.86	521.23	0.008	-1.29
PA4450_murA_at	1215.32	1223.22	0.643	-1.01
PA4451_at	825.58	892.75	0.484	-1.08

Supplementary Table 1. Probe set signal data for experimental and control samples

PA4452_at	187.72	221.65	0.151	-1.18
PA4453_at	680.82	884.05	0.008	-1.30
PA4454_at	653.74	679.55	0.484	-1.04
PA4455_at	244.02	370.45	0.008	-1.52
PA4456_at	365.18	461.60	0.024	-1.26
PA4457_at	482.22	525.35	0.349	-1.09
PA4458_at	405.98	357.03	0.151	1.14
PA4459_at	365.28	505.90	0.008	-1.38
PA4460_at	1295.20	1427.88	0.087	-1.10
PA4461_at	465.16	685.32	0.008	-1.47
PA4462_rpoN_at	299.82	379.73	0.008	-1.27
PA4463_at	534.14	671.80	0.087	-1.26
PA4464_ptsN_at	1230.76	895.97	0.008	1.37
PA4465_at	604.68	659.67	1.000	-1.09
PA4466_at	255.56	316.40	0.008	-1.24
PA4467_at	8.26	13.95	0.048	-1.69
PA4468_sodM_at	27.68	18.75	0.008	1.48
PA4469_at	35.74	36.78	0.817	-1.03
PA4470_fumC1_at	37.60	38.53	0.817	-1.02
PA4471_at	10.76	12.77	1.000	-1.19
PA4472_pmbA_at	98.48	83.32	0.151	1.18
PA4473_at	301.96	458.45	0.008	-1.52
PA4474_at	162.30	146.93	0.643	1.10
PA4475_at	132.30	111.63	0.048	1.19
PA4476_at	42.26	49.50	0.238	-1.17
PA4477_cafA_at	88.60	103.88	0.048	-1.17
PA4478_at	72.28	100.20	0.008	-1.39
PA4479_mreD_at	219.36	231.63	0.048	-1.06
PA4480_mreC_at	492.58	437.15	0.024	1.13
PA4481_mreB_at	1143.68	1150.85	0.643	-1.01
PA4482_gatC_at	513.24	665.57	0.024	-1.30
PA4483_gatA_at	850.90	1077.20	0.008	-1.27
PA4484_gatB_at	863.36	643.00	0.024	1.34
PA4485_at	27.06	34.05	0.151	-1.26
PA4486_at	44.74	42.50	0.643	1.05
PA4487_at	133.70	150.75	0.349	-1.13
PA4488_at	62.64	70.52	0.349	-1.13
PA4489_at	268.98	326.77	0.151	-1.21
PA4490_at	408.42	380.92	0.484	1.07
PA4491_at	451.62	288.08	0.024	1.57
PA4492_at	670.60	544.13	0.048	1.23
PA4493_at	278.60	222.50	0.048	1.25
PA4494_at	55.80	53.13	0.643	1.05
PA4495_at	276.30	336.13	0.238	-1.22
PA4496_at	432.12	205.43	0.008	2.10
PA4497_at	109.24	61.68	0.008	1.77
PA4498_at	76.84	143.10	0.008	-1.86
PA4499_at	113.94	124.13	0.238	-1.09
PA4500_at	343.02	447.75	0.238	-1.31
PA4501_at	203.32	207.93	0.817	-1.02
PA4502_at	292.40	307.52	1.000	-1.05

Supplementary Table 1. Probe set signal data for experimental and control samples

PA4503_at	122.76	197.03	0.087	-1.61
PA4504_at	254.16	227.35	0.484	1.12
PA4505_at	116.00	162.95	0.349	-1.40
PA4506_at	222.38	249.30	0.484	-1.12
PA4507_at	49.26	47.62	0.817	1.03
PA4508_at	26.00	28.95	0.238	-1.11
PA4509_at	17.02	21.80	0.484	-1.28
PA4510_at	56.30	44.78	0.151	1.26
PA4511_at	116.02	95.05	0.008	1.22
PA4512_at	80.26	92.18	0.151	-1.15
PA4513_at	32.70	40.50	0.087	-1.24
PA4514_at	393.50	175.88	0.087	2.24
PA4515_at	189.74	232.70	0.087	-1.23
PA4516_at	113.92	95.35	0.484	1.19
PA4517_at	25.06	13.73	0.048	1.83
PA4518_at	28.42	32.58	0.484	-1.15
PA4519_at	108.30	198.88	0.008	-1.84
PA4520_at	52.10	36.32	0.008	1.43
PA4521_at	59.12	46.25	0.024	1.28
PA4522_ampD_at	46.72	34.13	0.024	1.37
PA4523_at	123.28	115.02	0.484	1.07
PA4524_nadC_at	70.84	93.50	0.008	-1.32
PA4525_pilA_at	200.68	291.65	0.008	-1.45
PA4526_pilB_at	167.58	110.97	0.024	1.51
PA4527_pilC_at	146.68	148.70	0.817	-1.01
PA4528_pilD_at	351.76	207.70	0.008	1.69
PA4529_at	370.56	292.92	0.008	1.27
PA4530_at	181.92	134.50	0.024	1.35
PA4531_at	26.16	27.38	0.484	-1.05
PA4532_at	55.98	68.88	0.024	-1.23
PA4533_at	78.76	57.98	0.008	1.36
PA4534_at	62.20	60.18	1.000	1.03
PA4535_at	156.48	93.90	0.008	1.67
PA4536_at	149.52	107.13	0.008	1.40
PA4537_at	253.04	280.25	0.643	-1.11
PA4538_ndh_at	195.84	174.88	0.151	1.12
PA4539_at	32.82	43.88	0.151	-1.34
PA4540_at	19.64	6.15	0.048	3.19
PA4541_at	16.28	20.25	0.817	-1.24
PA4542_clpB_at	116.86	132.50	0.817	-1.13
PA4543_at	56.22	42.75	0.008	1.32
PA4544_rluD_at	268.60	246.00	0.151	1.09
PA4545_comL_at	461.00	688.25	0.008	-1.49
PA4546_pilS_at	81.98	79.15	0.151	1.04
PA4547_pilR_at	141.90	117.28	0.024	1.21
PA4548_at	90.44	109.28	0.063	-1.21
PA4549_fimT_at	6.34	3.15	0.151	2.01
PA4550_fimU_at	58.78	102.88	0.008	-1.75
PA4551_pilV_at	108.78	181.73	0.087	-1.67
PA4552_pilW_at	76.40	129.90	0.024	-1.70
PA4553_pilX_at	37.88	74.40	0.048	-1.96

Supplementary Table 1. Probe set signal data for experimental and control samples

PA4554_pilY1_at	100.22	139.13	0.048	-1.39
PA4555_pilY2_at	28.78	85.03	0.008	-2.95
PA4556_pilE_at	97.10	120.78	0.151	-1.24
PA4557_lytB_at	133.60	161.52	0.111	-1.21
PA4558_at	796.70	875.83	0.151	-1.10
PA4559_lspA_at	300.94	344.15	0.087	-1.14
PA4560_ileS_at	299.94	368.33	0.024	-1.23
PA4561_ribF_at	419.78	358.95	0.151	1.17
PA4562_at	87.96	80.55	0.349	1.09
PA4563_rpsT_at	1600.92	3648.50	0.008	-2.28
PA4564_at	64.76	78.78	0.238	-1.22
PA4565_proB_at	392.24	370.67	0.817	1.06
PA4566_obg_at	450.40	479.75	0.349	-1.07
PA4567_rpmA_at	1402.18	2335.23	0.008	-1.67
PA4568_rplU_at	4032.84	4282.55	0.817	-1.06
PA4569_ispB_at	563.56	549.47	0.643	1.03
PA4570_at	16.12	30.35	0.048	-1.88
PA4571_at	89.92	156.82	0.087	-1.74
PA4572_fklB_at	557.86	660.63	0.238	-1.18
PA4573_at	34.24	33.22	0.349	1.03
PA4574_at	108.00	170.35	0.008	-1.58
PA4575_at	37.36	26.38	0.008	1.42
PA4576_at	217.58	193.00	0.008	1.13
PA4577_at	61.14	51.85	0.635	1.18
PA4578_at	3303.70	2363.07	0.008	1.40
PA4579_at	114.76	94.15	0.008	1.22
PA4580_at	66.94	79.35	0.349	-1.19
PA4581_rtcR_at	29.88	30.53	0.643	-1.02
PA4582_at	212.98	66.05	0.008	3.22
PA4583_at	272.58	106.35	0.008	2.56
PA4584_at	141.50	47.47	0.008	2.98
PA4585_rtcA_at	135.10	66.55	0.008	2.03
PA4586_at	37.82	19.58	0.008	1.93
PA4587_ccpR_at	66.72	64.15	0.643	1.04
PA4588_gdhA_at	40.46	35.42	0.151	1.14
PA4589_at	30.48	28.63	0.484	1.06
PA4590_pra_at	53.50	73.93	0.008	-1.38
PA4591_at	27.96	25.23	0.643	1.11
PA4592_at	46.08	40.17	0.413	1.15
PA4593_at	29.88	25.85	0.484	1.16
PA4594_at	33.48	28.23	0.111	1.19
PA4595_at	858.68	725.83	0.008	1.18
PA4596_at	37.80	44.67	0.151	-1.18
PA4597_oprJ_at	52.82	56.80	0.643	-1.08
PA4598_mexD_at	18.36	31.17	0.032	-1.70
PA4599_mexC_at	22.38	26.00	0.151	-1.16
PA4600_nfxB_at	105.36	127.97	0.087	-1.21
PA4601_at	50.68	47.53	0.817	1.07
PA4602_glyA3_at	1006.08	1374.95	0.024	-1.37
PA4603_at	21.02	22.30	0.484	-1.06
PA4604_at	105.12	100.68	0.643	1.04

Supplementary Table 1. Probe set signal data for experimental and control samples

PA4605_at	130.76	125.03	0.817	1.05
PA4606_at	584.94	441.10	0.151	1.33
PA4607_at	158.40	153.13	0.643	1.03
PA4608_at	74.18	56.92	0.151	1.30
PA4609_radA_at	55.60	68.30	0.048	-1.23
PA4610_at	26.78	29.88	0.484	-1.12
PA4611_at	32.88	60.65	0.151	-1.84
PA4612_at	9.10	14.60	0.048	-1.60
PA4613_katB_at	152.56	33.60	0.008	4.54
PA4614_mscL_at	107.70	185.95	0.008	-1.73
PA4615_at	72.98	81.05	0.238	-1.11
PA4616_at	62.84	96.75	0.008	-1.54
PA4617_at	50.48	51.15	0.817	-1.01
PA4618_at	39.64	35.55	0.817	1.12
PA4619_at	87.20	106.25	0.048	-1.22
PA4620_at	140.36	186.35	0.024	-1.33
PA4621_at	54.46	78.45	0.024	-1.44
PA4622_at	25.00	34.03	0.087	-1.36
PA4623_r_at	14.88	26.85	0.143	-1.80
PA4624_at	18.36	28.55	0.024	-1.56
PA4625_at	53.88	64.00	0.024	-1.19
PA4626_hprA_at	187.76	127.07	0.008	1.48
PA4627_at	324.56	302.27	0.349	1.07
PA4628_lysP_at	70.14	116.78	0.008	-1.66
PA4629_at	82.70	55.03	0.008	1.50
PA4630_at	16.26	13.02	0.349	1.25
PA4631_at	125.32	120.03	0.643	1.04
PA4632_at	1075.08	951.67	0.048	1.13
PA4633_at	196.66	163.55	0.151	1.20
PA4634_at	39.46	39.52	0.643	-1.00
PA4635_at	17.46	9.60	0.151	1.82
PA4636_at	371.28	370.80	0.643	1.00
PA4637_i_at	16.74	21.55	0.349	-1.29
PA4638_at	15.90	33.60	0.008	-2.11
PA4639_at	706.32	765.00	0.349	-1.08
PA4640_mqoB_at	727.98	736.92	0.484	-1.01
PA4641_at	22.52	31.78	0.048	-1.41
PA4642_at	104.62	236.05	0.008	-2.26
PA4643_at	136.00	115.05	0.151	1.18
PA4644_at	37.86	63.82	0.143	-1.69
PA4645_at	305.72	685.57	0.008	-2.24
PA4646_upp_at	550.14	466.27	0.008	1.18
PA4647_uraA_at	183.10	230.70	0.048	-1.26
PA4648_at	42.44	38.05	0.484	1.12
PA4649_at	37.50	16.85	0.008	2.23
PA4650_at	2.58	6.90	0.151	-2.67
PA4651_at	12.48	11.25	0.817	1.11
PA4652_at	5.04	3.92	0.413	1.29
PA4653_at	15.88	11.43	0.238	1.39
PA4654_at	3.42	7.42	0.349	-2.17
PA4655_hemH_at	317.26	321.98	0.817	-1.01

Supplementary Table 1. Probe set signal data for experimental and control samples

PA4656_at	70.80	68.58	0.817	1.03
PA4657_at	94.04	79.15	0.087	1.19
PA4658_at	64.08	69.82	0.484	-1.09
PA4659_at	19.40	28.35	0.048	-1.46
PA4660_phr_at	80.92	69.50	0.151	1.16
PA4661_at	704.34	851.13	0.087	-1.21
PA4662_murI_at	139.30	169.73	0.024	-1.22
PA4663_moeB_at	140.68	160.45	0.643	-1.14
PA4664_hemK_at	303.04	334.92	0.349	-1.11
PA4665_prfA_at	378.42	442.77	0.087	-1.17
PA4666_hemA_at	281.06	320.30	0.151	-1.14
PA4667_at	426.80	336.52	0.008	1.27
PA4668_at	263.36	204.88	0.008	1.29
PA4669_ipk_at	188.96	202.65	0.349	-1.07
PA4670_prs_at	1795.96	1899.40	0.349	-1.06
PA4671_at	2740.04	3300.27	0.349	-1.20
PA4672_at	607.92	780.15	0.048	-1.28
PA4673_at	211.26	448.82	0.008	-2.12
PA4674_at	11.54	15.60	0.151	-1.35
PA4675_at	330.96	584.97	0.048	-1.77
PA4676_at	261.64	237.07	0.008	1.10
PA4677_at	67.54	56.47	0.087	1.20
PA4678_rimI_at	80.66	81.40	0.817	-1.01
PA4679_at	119.00	112.73	0.643	1.06
PA4680_at	22.08	14.80	0.111	1.49
PA4681_at	7.18	22.40	0.024	-3.12
PA4682_at	28.64	23.42	0.190	1.22
PA4683_at	4.86	4.68	0.643	1.04
PA4684_at	173.56	126.82	0.008	1.37
PA4685_at	4.64	1.30	0.063	3.57
PA4686_at	685.40	632.78	0.238	1.08
PA4687_hitA_at	353.46	445.63	0.151	-1.26
PA4688_hitB_at	110.46	166.82	0.008	-1.51
PA4689_at	51.56	56.50	0.151	-1.10
PA4690_at	123.34	111.03	0.008	1.11
PA4691_at	22.10	20.55	1.000	1.08
PA4692_at	41.44	38.22	0.817	1.08
PA4693_pssA_at	197.54	175.98	0.151	1.12
PA4694_ilvC_at	1003.34	925.95	0.087	1.08
PA4695_ilvH_at	692.14	713.75	0.643	-1.03
PA4696_ilvI_at	527.76	439.32	0.008	1.20
PA4697_at	406.22	318.85	0.349	1.27
PA4698_at	346.66	316.47	0.349	1.10
PA4699_at	191.72	158.28	0.024	1.21
PA4700_mrcB_at	188.36	170.13	0.087	1.11
PA4701_at	426.82	432.57	0.817	-1.01
PA4702_at	18.74	19.80	0.817	-1.06
PA4703_at	12.14	18.63	0.286	-1.53
PA4704_at	96.12	69.43	0.024	1.38
PA4705_at	25.84	39.58	0.048	-1.53
PA4706_at	55.12	47.53	0.087	1.16

Supplementary Table 1. Probe set signal data for experimental and control samples

PA4707_at	42.12	28.08	0.008	1.50
PA4708_at	56.82	56.37	0.643	1.01
PA4709_at	43.00	73.40	0.087	-1.71
PA4710_at	17.84	46.00	0.024	-2.58
PA4711_at	93.14	83.92	1.000	1.11
PA4712_at	29.08	41.33	0.087	-1.42
PA4713_at	17.60	12.60	0.151	1.40
PA4714_at	96.38	82.42	0.484	1.17
PA4715_at	67.34	107.47	0.008	-1.60
PA4716_at	49.46	39.92	0.151	1.24
PA4717_at	74.66	68.28	0.151	1.09
PA4718_at	32.90	32.60	0.905	1.01
PA4719_at	103.36	223.68	0.008	-2.16
PA4720_trmA_at	207.66	328.08	0.008	-1.58
PA4721_at	28.42	30.40	0.556	-1.07
PA4722_at	234.14	198.25	0.349	1.18
PA4723_dksA_at	756.76	1289.22	0.008	-1.70
PA4724_at	51.38	73.80	0.008	-1.44
PA4725_at	50.52	42.80	0.008	1.18
PA4726_at	447.48	368.25	0.024	1.22
PA4727_pcnB_at	381.84	328.33	0.024	1.16
PA4728_folK_at	118.46	119.73	0.484	-1.01
PA4729_panB_at	758.96	669.08	0.087	1.13
PA4730_panC_at	642.34	551.28	0.008	1.17
PA4731_panD_at	261.80	453.00	0.008	-1.73
PA4732_pgi_at	417.94	363.33	0.048	1.15
PA4733_acsB_at	159.64	94.68	0.008	1.69
PA4734_at	144.14	115.88	0.008	1.24
PA4735_at	110.54	100.50	0.087	1.10
PA4736_at	65.08	84.60	0.048	-1.30
PA4737_at	61.14	46.75	0.087	1.31
PA4738_at	43.36	43.68	1.000	-1.01
PA4739_at	95.88	97.05	0.817	-1.01
PA4740_pnp_at	685.58	1022.60	0.008	-1.49
PA4741_rpsO_at	1414.28	1798.65	0.008	-1.27
PA4742_truB_at	354.04	472.45	0.024	-1.33
PA4743_rbfA_at	588.02	740.25	0.008	-1.26
PA4744_infB_at	962.88	946.57	0.817	1.02
PA4745_nusA_at	1222.80	1481.63	0.008	-1.21
PA4746_at	472.02	607.85	0.024	-1.29
PA4747_secG_at	149.92	391.15	0.008	-2.61
PA4748_tpiA_at	488.04	675.22	0.008	-1.38
PA4749_glmM_at	293.84	328.85	0.048	-1.12
PA4750_folP_at	139.78	159.85	0.151	-1.14
PA4751_ftsH_at	799.82	578.18	0.008	1.38
PA4752_ftsJ_at	46.64	83.30	0.008	-1.79
PA4753_at	69.04	156.68	0.008	-2.27
PA4754_at	183.28	209.00	0.238	-1.14
PA4755_greA_at	247.36	301.65	0.087	-1.22
PA4756_carB_at	734.52	755.13	1.000	-1.03
PA4757_at	400.16	527.00	0.048	-1.32

Supplementary Table 1. Probe set signal data for experimental and control samples

PA4758_carA_at	443.20	465.00	0.817	-1.05
PA4759_dapB_at	181.44	409.05	0.008	-2.25
PA4760_dnaJ_at	392.84	528.17	0.008	-1.34
PA4761_dnaK_at	1074.44	1359.10	0.024	-1.26
PA4762_grpE_at	2740.28	2730.55	0.643	1.00
PA4763_recN_at	1270.90	153.60	0.008	8.27
PA4764_fur_at	181.52	287.45	0.008	-1.58
PA4765_omlA_at	1041.66	992.20	0.643	1.05
PA4766_at	34.86	45.63	0.087	-1.31
PA4767_at	183.46	156.98	0.151	1.17
PA4768_smpB_at	94.68	140.43	0.024	-1.48
PA4769_at	125.88	129.45	0.643	-1.03
PA4770_lldP_at	17.58	17.63	0.484	-1.00
PA4771_lldD_at	21.02	21.13	1.000	-1.01
PA4772_at	31.12	19.85	0.151	1.57
PA4773_at	623.38	568.58	0.484	1.10
PA4774_at	196.80	171.05	0.238	1.15
PA4775_at	249.76	170.65	0.048	1.46
PA4776_at	326.04	235.57	0.024	1.38
PA4777_at	68.96	54.45	0.048	1.27
PA4778_at	317.74	317.25	0.643	1.00
PA4779_at	16.12	9.20	0.079	1.75
PA4780_at	102.44	112.20	0.349	-1.10
PA4781_at	26.94	13.52	0.048	1.99
PA4782_at	74.66	58.00	0.151	1.29
PA4783_at	20.30	19.88	0.643	1.02
PA4784_at	91.76	88.42	0.817	1.04
PA4785_at	27.48	12.45	0.008	2.21
PA4786_at	34.38	24.60	0.087	1.40
PA4787_at	110.66	73.42	0.024	1.51
PA4788_at	32.66	20.83	0.008	1.57
PA4789_at	110.08	122.38	0.349	-1.11
PA4790_at	68.22	68.43	0.643	-1.00
PA4791_at	95.46	79.55	0.087	1.20
PA4792_at	48.80	46.45	0.643	1.05
PA4793_at	220.06	201.05	0.349	1.09
PA4794_at	106.66	74.15	0.008	1.44
PA4795_at	104.84	116.68	0.484	-1.11
PA4796_at	44.54	55.95	0.048	-1.26
PA4798_at	54.72	35.72	0.008	1.53
PA4799_at	14.94	12.93	0.484	1.16
PA4800_at	27.18	26.20	1.000	1.04
PA4801_at	41.32	40.53	0.817	1.02
PA4802_at	26.00	37.65	0.238	-1.45
PA4803_at	55.40	45.70	0.349	1.21
PA4804_at	12.18	12.67	0.817	-1.04
PA4805_at	10.14	11.93	0.817	-1.18
PA4806_at	23.44	16.60	0.413	1.41
PA4807_selB_at	107.64	71.60	0.024	1.50
PA4808_selA_at	170.40	146.80	0.087	1.16
PA4809_fdhE_at	104.38	98.40	0.484	1.06

Supplementary Table 1. Probe set signal data for experimental and control samples

PA4810_fdnI_at	170.38	132.65	0.349	1.28
PA4811_fdnH_at	163.02	148.20	0.484	1.10
PA4812_fdnG_at	399.78	329.30	0.048	1.21
PA4813_lipC_at	2.24	2.05	0.484	1.09
PA4814_fadH2_at	27.04	20.63	0.643	1.31
PA4815_at	41.94	49.65	0.349	-1.18
PA4816_at	34.18	26.82	0.143	1.27
PA4817_at	54.54	66.40	0.238	-1.22
PA4818_at	6.00	14.73	0.048	-2.45
PA4819_at	18.08	19.90	0.730	-1.10
PA4820_at	5.02	7.27	0.817	-1.45
PA4821_at	25.44	40.70	0.008	-1.60
PA4822_at	9.92	10.22	0.817	-1.03
PA4823_at	10.86	17.90	0.048	-1.65
PA4824_at	22.30	23.00	1.000	-1.03
PA4825_mgtA_at	42.10	27.42	0.151	1.54
PA4826_at	213.84	141.08	0.151	1.52
PA4827_at	25.72	19.57	0.151	1.31
PA4828_at	7.58	13.65	0.087	-1.80
PA4829_lpd3_at	16.50	15.50	0.817	1.06
PA4830_at	23.74	22.60	0.905	1.05
PA4831_at	55.96	54.88	1.000	1.02
PA4832_at	22.38	17.85	0.238	1.25
PA4833_at	34.16	32.33	0.643	1.06
PA4834_at	21.48	12.92	0.151	1.66
PA4835_at	11.86	13.50	0.643	-1.14
PA4836_at	10.98	18.97	0.151	-1.73
PA4837_at	27.68	43.50	0.151	-1.57
PA4838_at	28.44	22.40	0.151	1.27
PA4839_speA_at	111.70	321.22	0.008	-2.88
PA4840_at	80.86	201.27	0.008	-2.49
PA4841_at	95.24	87.73	0.349	1.09
PA4842_at	315.48	329.63	0.238	-1.04
PA4843_at	280.62	138.10	0.008	2.03
PA4844_at	23.80	19.73	0.484	1.21
PA4845_dipZ_at	61.18	70.18	0.238	-1.15
PA4846_aroQ1_at	654.44	982.15	0.008	-1.50
PA4847_accB_at	1810.88	2072.63	0.048	-1.14
PA4848_accC_at	1684.84	1580.83	0.087	1.07
PA4849_at	30.50	43.10	0.008	-1.41
PA4850_prmA_at	568.98	373.33	0.008	1.52
PA4851_at	240.40	198.48	0.063	1.21
PA4852_at	866.12	895.90	1.000	-1.03
PA4853_fis_at	729.12	747.22	0.484	-1.02
PA4854_purH_at	367.04	466.95	0.008	-1.27
PA4855_purD_at	388.66	487.85	0.087	-1.26
PA4856_at	232.62	228.25	0.817	1.02
PA4857_at	22.92	27.00	0.484	-1.18
PA4858_at	13.72	11.55	0.643	1.19
PA4859_at	47.20	35.63	0.151	1.32
PA4860_at	11.54	15.92	0.048	-1.38

PA4861_at	8.02	6.60	1.000	1.22
PA4862_at	5.64	11.95	0.087	-2.12
PA4863_at	166.46	119.60	0.008	1.39
PA4864_ureD_at	47.10	36.50	0.151	1.29
PA4865_ureA_at	141.58	122.15	0.087	1.16
PA4866_at	92.16	60.00	0.008	1.54
PA4867_ureB_at	56.06	52.95	0.484	1.06
PA4868_ureC_at	72.36	59.53	0.151	1.22
PA4869_at	28.14	37.30	0.048	-1.33
PA4870_at	55.86	75.68	0.151	-1.35
PA4871_at	10.70	15.25	0.238	-1.43
PA4872_at	292.34	188.38	0.008	1.55
PA4873_at	40.06	91.47	0.008	-2.28
PA4874_at	116.98	108.63	0.643	1.08
PA4875_at	38.70	27.25	0.008	1.42
PA4876_osmE_at	84.32	80.35	0.643	1.05
PA4877_at	28.74	23.58	0.349	1.22
PA4878_at	55.04	45.82	0.349	1.20
PA4879_at	29.84	21.50	0.349	1.39
PA4880_at	62.60	48.50	0.151	1.29
PA4881_at	21.82	17.67	0.349	1.23
PA4882_at	2.30	7.67	0.238	-3.33
PA4883_at	8.90	4.38	0.151	2.03
PA4884_at	13.28	16.63	0.349	-1.25
PA4885_irlR_at	50.42	47.05	0.817	1.07
PA4886_at	14.64	11.13	0.238	1.32
PA4887_at	39.48	61.67	0.048	-1.56
PA4888_at	50.62	71.68	0.151	-1.42
PA4889_at	58.78	90.75	0.008	-1.54
PA4890_at	112.48	102.72	0.349	1.10
PA4891_ureE_at	16.82	8.35	0.040	2.01
PA4892_ureF_at	38.34	42.67	0.484	-1.11
PA4893_ureG_at	36.82	45.03	0.238	-1.22
PA4894_i_at	21.70	28.58	0.349	-1.32
PA4895_at	16.46	14.17	0.087	1.16
PA4896_at	26.90	27.75	0.905	-1.03
PA4897_at	36.32	32.78	0.556	1.11
PA4898_at	12.52	8.90	0.817	1.41
PA4899_at	8.96	5.72	0.817	1.57
PA4900_at	13.12	16.42	0.349	-1.25
PA4901_mdlC_at	5.54	9.18	0.238	-1.66
PA4902_at	42.96	40.05	0.230	1.07
PA4903_at	24.20	17.67	0.151	1.37
PA4904_vanA_at	16.56	15.65	0.817	1.06
PA4905_vanB_at	1.40	2.98	0.087	-2.13
PA4906_at	52.46	51.17	0.817	1.03
PA4907_at	373.82	364.23	0.643	1.03
PA4908_at	13.08	18.25	0.087	-1.40
PA4909_at	6.44	10.35	0.230	-1.61
PA4910_at	17.58	16.33	0.817	1.08
PA4911_at	13.44	6.50	0.484	2.07

Supplementary Table 1. Probe set signal data for experimental and control samples

PA4912_at	14.36	12.45	0.643	1.15
PA4913_at	28.86	26.52	0.556	1.09
PA4914_at	32.36	27.48	0.151	1.18
PA4915_at	18.24	17.02	0.817	1.07
PA4916_at	63.62	53.15	0.238	1.20
PA4917_at	96.82	77.03	0.087	1.26
PA4918_at	291.84	233.80	0.151	1.25
PA4919_pncB1_at	241.90	208.02	0.151	1.16
PA4920_nadE_at	705.44	527.00	0.008	1.34
PA4921_at	66.18	54.35	0.024	1.22
PA4922_azu_at	3964.62	2592.77	0.008	1.53
PA4923_at	169.66	246.35	0.008	-1.45
PA4924_at	43.68	45.75	1.000	-1.05
PA4925_at	14.26	14.40	1.000	-1.01
PA4926_at	10.02	21.75	0.048	-2.17
PA4927_at	19.84	15.30	0.238	1.30
PA4928_at	154.88	207.00	0.008	-1.34
PA4929_at	14.46	13.22	0.817	1.09
PA4930_alr_at	112.72	121.38	0.817	-1.08
PA4931_dnaB_at	275.60	318.22	0.151	-1.15
PA4932_rplI_at	2612.24	2274.02	0.349	1.15
PA4933_at	1781.12	2134.05	0.024	-1.20
PA4934_rpsR_at	2232.50	2751.55	0.238	-1.23
PA4935_rpsF_at	2483.82	3007.65	0.048	-1.21
PA4936_at	192.06	176.73	0.349	1.09
PA4937_rnr_at	667.64	344.60	0.008	1.94
PA4938_purA_at	642.74	785.25	0.048	-1.22
PA4939_at	148.08	241.55	0.008	-1.63
PA4940_at	391.46	450.92	0.087	-1.15
PA4941_hflC_at	934.08	744.42	0.008	1.25
PA4942_hflK_at	887.60	712.18	0.008	1.25
PA4943_at	1229.78	1018.48	0.151	1.21
PA4944_at	1393.60	1644.67	0.087	-1.18
PA4945_miaA_at	80.26	91.80	0.024	-1.14
PA4946_mutL_at	138.96	134.27	0.643	1.03
PA4947_amiB_at	419.64	310.97	0.008	1.35
PA4948_at	86.10	98.98	0.238	-1.15
PA4949_at	68.16	74.25	0.349	-1.09
PA4950_at	58.42	50.42	0.238	1.16
PA4951_orn_at	288.28	304.08	0.817	-1.05
PA4952_at	153.02	174.35	0.048	-1.14
PA4953_motB_at	150.90	118.57	0.024	1.27
PA4954_motA_at	67.18	79.85	0.349	-1.19
PA4955_at	86.28	61.00	0.024	1.41
PA4956_rhdA_at	194.64	215.12	0.008	-1.11
PA4957_psd_at	296.22	286.52	0.817	1.03
PA4958_at	143.64	120.65	0.048	1.19
PA4959_at	154.90	147.15	0.413	1.05
PA4960_at	157.26	188.73	0.008	-1.20
PA4961_at	205.18	194.60	0.349	1.05
PA4962_at	19.20	17.73	0.643	1.08

Supplementary Table 1. Probe set signal data for experimental and control samples

PA4963_at	96.56	116.45	0.151	-1.21
PA4964_parC_at	269.04	176.75	0.008	1.52
PA4965_at	225.50	171.82	0.349	1.31
PA4966_at	240.80	172.60	0.008	1.40
PA4967_parE_at	240.96	174.93	0.008	1.38
PA4968_at	257.36	150.07	0.008	1.71
PA4969_at	60.52	54.30	0.238	1.11
PA4970_at	187.28	168.98	0.643	1.11
PA4971_at	119.02	123.75	0.817	-1.04
PA4972_at	82.82	110.35	0.008	-1.33
PA4973_thiC_at	41.62	51.65	0.087	-1.24
PA4974_at	540.68	510.48	0.349	1.06
PA4975_at	9.28	18.63	0.238	-2.01
PA4976_aspC_at	29.46	29.10	1.000	1.01
PA4977_at	43.12	29.65	0.151	1.45
PA4978_at	17.48	19.43	0.484	-1.11
PA4979_at	15.14	12.75	0.238	1.19
PA4980_at	22.18	30.48	0.238	-1.37
PA4981_at	4.50	15.05	0.008	-3.34
PA4982_at	21.48	23.20	0.643	-1.08
PA4983_at	62.18	32.80	0.008	1.90
PA4984_at	51.20	34.55	0.048	1.48
PA4985_at	24.00	20.63	0.349	1.16
PA4986_at	27.50	20.10	0.484	1.37
PA4987_at	68.64	60.15	0.151	1.14
PA4988_waaA_at	78.96	59.28	0.048	1.33
PA4989_at	29.54	20.60	0.238	1.43
PA4990_at	12.42	17.20	0.349	-1.38
PA4991_at	167.66	167.52	0.643	1.00
PA4992_at	51.04	63.38	0.087	-1.24
PA4993_at	49.16	45.47	0.349	1.08
PA4994_at	22.76	27.60	0.635	-1.21
PA4995_at	17.62	6.70	0.008	2.63
PA4996_rfaE_at	270.76	266.65	0.643	1.02
PA4997_msbA_at	236.14	292.13	0.087	-1.24
PA4998_at	425.56	344.28	0.008	1.24
PA4999_at	46.58	44.67	0.643	1.04
PA5000_at	102.98	171.23	0.008	-1.66
PA5001_at	138.40	188.43	0.008	-1.36
PA5002_at	125.82	113.60	0.349	1.11
PA5003_at	163.80	157.68	0.643	1.04
PA5004_at	200.40	191.75	0.484	1.05
PA5005_at	494.24	653.92	0.008	-1.32
PA5006_at	119.22	82.08	0.048	1.45
PA5007_at	104.20	146.43	0.008	-1.41
PA5008_at	238.90	233.60	1.000	1.02
PA5009_waaP_at	140.36	165.82	0.087	-1.18
PA5010_waaG_at	188.62	217.30	0.151	-1.15
PA5011_waaC_at	317.36	332.00	0.484	-1.05
PA5012_waaF_at	422.44	521.13	0.151	-1.23
PA5013_ilvE_at	552.90	800.05	0.008	-1.45

Supplementary Table 1. Probe set signal data for experimental and control samples

PA5014_glnE_at	116.40	93.40	0.008	1.25
PA5015_aceE_at	886.66	1283.07	0.008	-1.45
PA5016_aceF_at	553.82	845.20	0.024	-1.53
PA5017_at	120.96	106.85	0.238	1.13
PA5018_msrA_at	343.50	240.15	0.008	1.43
PA5019_at	99.28	96.40	1.000	1.03
PA5020_at	20.48	16.67	0.484	1.23
PA5021_at	81.04	71.05	0.151	1.14
PA5022_at	115.82	103.88	0.238	1.11
PA5023_at	28.50	28.17	0.817	1.01
PA5024_at	21.60	71.60	0.008	-3.31
PA5025_metY_at	135.56	115.20	0.024	1.18
PA5026_at	27.98	36.97	0.151	-1.32
PA5027_at	22.28	32.55	0.238	-1.46
PA5028_at	289.10	248.92	0.151	1.16
PA5029_at	37.84	42.15	0.349	-1.11
PA5030_at	40.12	54.02	0.024	-1.35
PA5031_at	20.58	24.40	0.643	-1.19
PA5032_at	15.10	8.63	0.230	1.75
PA5033_at	114.08	92.13	0.048	1.24
PA5034_hemE_at	53.90	73.68	0.048	-1.37
PA5035_gltD_at	93.36	84.68	0.238	1.10
PA5036_gltB_at	149.72	119.32	0.008	1.25
PA5037_at	426.30	460.70	0.087	-1.08
PA5038_aroB_at	107.18	171.73	0.008	-1.60
PA5039_aroK_at	327.80	380.85	0.048	-1.16
PA5040_pilQ_at	605.84	513.33	0.048	1.18
PA5041_pilP_at	513.30	495.17	0.643	1.04
PA5042_pilO_at	334.46	344.78	1.000	-1.03
PA5043_pilN_at	442.90	437.32	0.817	1.01
PA5044_pilM_at	818.20	694.75	0.048	1.18
PA5045_ponA_at	141.64	127.35	0.238	1.11
PA5046_at	2325.92	1705.93	0.008	1.36
PA5047_at	87.76	136.10	0.008	-1.55
PA5048_at	193.66	225.73	0.008	-1.17
PA5049_rpmE_at	174.94	481.95	0.008	-2.75
PA5050_priA_at	123.64	113.35	0.817	1.09
PA5051_argS_at	265.86	369.17	0.008	-1.39
PA5052_at	359.76	410.23	0.087	-1.14
PA5053_hslV_at	631.56	798.82	0.238	-1.26
PA5054_hslU_at	1739.70	1661.72	0.817	1.05
PA5055_at	206.78	225.32	0.643	-1.09
PA5056_phaC1_at	98.62	69.55	0.087	1.42
PA5057_phaD_at	34.66	22.70	0.008	1.53
PA5058_phaC2_at	24.30	20.80	0.151	1.17
PA5059_at	5.70	2.80	0.111	2.04
PA5060_phaF_at	300.56	266.27	0.349	1.13
PA5061_at	46.18	51.93	0.238	-1.12
PA5062_at	114.96	111.57	0.817	1.03
PA5063_ubiE_at	609.12	583.43	0.349	1.04
PA5064_at	437.08	482.27	0.349	-1.10

Supplementary Table 1. Probe set signal data for experimental and control samples

PA5065_at	220.14	240.48	0.087	-1.09
PA5066_hisI_at	455.62	601.47	0.008	-1.32
PA5067_hisE_at	406.38	554.22	0.008	-1.36
PA5068_tatA_at	276.38	405.13	0.008	-1.47
PA5069_tatB_at	463.38	452.38	0.484	1.02
PA5070_tatC_at	115.50	130.18	0.484	-1.13
PA5071_at	66.32	70.00	0.643	-1.06
PA5072_at	148.48	159.98	0.643	-1.08
PA5073_at	87.24	94.50	0.643	-1.08
PA5074_at	106.84	126.07	0.087	-1.18
PA5075_at	373.68	335.95	0.151	1.11
PA5076_at	129.66	249.40	0.008	-1.92
PA5077_mdoH_at	237.74	205.18	0.238	1.16
PA5078_at	887.92	742.63	0.008	1.20
PA5079_at	142.64	172.63	0.048	-1.21
PA5080_at	343.44	313.95	0.484	1.09
PA5081_at	44.76	33.38	0.087	1.34
PA5082_at	37.72	49.35	0.008	-1.31
PA5083_at	25.16	28.67	0.349	-1.14
PA5084_at	7.80	7.43	0.817	1.05
PA5085_at	70.50	50.80	0.087	1.39
PA5086_at	21.58	12.08	0.087	1.79
PA5087_at	14.20	11.85	0.643	1.20
PA5088_at	13.14	9.95	0.151	1.32
PA5089_at	26.06	31.38	0.032	-1.20
PA5090_at	46.76	41.85	0.413	1.12
PA5091_hutG_at	59.48	62.90	0.349	-1.06
PA5092_hutI_at	23.60	22.88	1.000	1.03
PA5093_at	14.00	10.30	0.151	1.36
PA5094_at	41.82	41.53	0.643	1.01
PA5095_at	45.44	41.85	0.817	1.09
PA5096_at	14.50	15.70	0.817	-1.08
PA5097_at	11.16	13.08	0.817	-1.17
PA5098_hutH_at	14.12	10.07	0.817	1.40
PA5099_at	10.42	9.55	0.817	1.09
PA5100_hutU_at	54.92	39.23	0.024	1.40
PA5101_at	40.16	27.95	0.190	1.44
PA5102_at	5.48	14.50	0.024	-2.65
PA5103_at	24.22	27.63	1.000	-1.14
PA5104_at	39.20	47.40	0.111	-1.21
PA5105_hutC_at	43.32	55.32	0.024	-1.28
PA5106_at	33.94	35.52	0.643	-1.05
PA5107_blc_at	86.28	81.45	0.643	1.06
PA5108_at	80.36	87.08	0.484	-1.08
PA5109_at	125.28	148.77	0.048	-1.19
PA5110_fbp_at	472.32	586.05	0.008	-1.24
PA5111_gloA3_at	246.54	235.68	0.238	1.05
PA5112_estA_at	945.30	613.63	0.008	1.54
PA5113_at	203.66	249.97	0.238	-1.23
PA5114_at	98.34	110.38	0.484	-1.12
PA5115_at	11.48	17.60	0.151	-1.53

Supplementary Table 1. Probe set signal data for experimental and control samples

PA5116_at	4.20	19.25	0.008	-4.58
PA5117_typA_at	425.90	671.80	0.008	-1.58
PA5118_thiI_at	278.08	349.50	0.151	-1.26
PA5119_glnA_at	1409.76	1449.25	0.817	-1.03
PA5120_at	63.48	88.20	0.024	-1.39
PA5121_at	49.48	52.90	0.286	-1.07
PA5122_at	141.72	110.52	0.048	1.28
PA5123_at	73.32	65.82	0.151	1.11
PA5124_ntrB_at	48.18	53.50	0.484	-1.11
PA5125_ntrC_at	60.84	68.82	0.087	-1.13
PA5126_at	49.64	43.85	0.238	1.13
PA5127_at	33.52	42.98	0.238	-1.28
PA5128_secB_at	1027.14	1513.45	0.008	-1.47
PA5129_grx_at	1770.96	1477.47	0.048	1.20
PA5130_at	863.34	965.68	0.238	-1.12
PA5131_pgm_at	597.58	586.72	0.643	1.02
PA5132_at	59.82	62.07	0.643	-1.04
PA5133_at	263.76	268.65	0.817	-1.02
PA5134_at	553.78	380.47	0.008	1.46
PA5135_at	173.36	133.40	0.008	1.30
PA5136_at	160.50	175.45	0.484	-1.09
PA5137_at	27.02	24.05	0.238	1.12
PA5138_at	102.46	108.97	0.817	-1.06
PA5139_at	167.84	316.55	0.008	-1.89
PA5140_hisF1_at	317.92	311.75	0.643	1.02
PA5141_hisA_at	303.80	366.98	0.008	-1.21
PA5142_hisH1_at	510.20	482.70	0.349	1.06
PA5143_hisB_at	368.76	507.27	0.008	-1.38
PA5144_i_at	3.84	4.30	0.286	-1.12
PA5145_at	51.90	43.43	0.087	1.20
PA5146_at	299.22	251.30	0.008	1.19
PA5147_mutY_at	283.72	360.25	0.008	-1.27
PA5148_at	1187.84	1047.60	0.643	1.13
PA5149_at	116.64	86.78	0.008	1.34
PA5150_at	23.22	13.47	0.151	1.72
PA5151_at	81.48	51.27	0.048	1.59
PA5152_at	935.68	533.60	0.008	1.75
PA5153_at	995.36	654.47	0.032	1.52
PA5154_at	201.10	140.88	0.048	1.43
PA5155_at	66.46	55.38	0.349	1.20
PA5156_at	47.80	60.60	0.048	-1.27
PA5157_at	28.42	39.77	0.008	-1.40
PA5158_at	41.96	46.83	0.817	-1.12
PA5159_at	13.06	30.40	0.079	-2.33
PA5160_at	27.04	34.45	0.238	-1.27
PA5161_rmlB_at	502.26	540.05	0.349	-1.08
PA5162_rmlD_at	427.08	510.15	0.048	-1.19
PA5163_rmlA_at	1041.66	1104.35	0.643	-1.06
PA5164_rmlC_at	326.44	419.00	0.024	-1.28
PA5165_at	73.08	58.58	0.048	1.25
PA5166_at	183.94	163.88	0.238	1.12

Supplementary Table 1. Probe set signal data for experimental and control samples

PA5167_at	611.26	240.67	0.008	2.54
PA5168_at	194.76	100.18	0.008	1.94
PA5169_at	111.70	64.68	0.008	1.73
PA5170_arcD_at	403.10	482.80	0.643	-1.20
PA5171_arcA_at	1136.12	1050.07	1.000	1.08
PA5172_arcB_at	1033.46	965.25	0.643	1.07
PA5173_arcC_at	377.38	261.40	0.151	1.44
PA5174_at	335.18	395.65	0.048	-1.18
PA5175_cysQ_at	54.88	45.05	0.238	1.22
PA5176_at	127.10	134.73	0.349	-1.06
PA5177_at	89.54	97.55	0.349	-1.09
PA5178_at	290.98	360.80	0.349	-1.24
PA5179_at	36.62	38.47	0.817	-1.05
PA5180_at	37.68	43.90	0.286	-1.17
PA5181_at	29.68	39.58	0.151	-1.33
PA5182_at	395.08	362.70	0.238	1.09
PA5183_at	87.00	76.95	0.238	1.13
PA5184_at	126.90	102.42	0.024	1.24
PA5185_at	25.64	21.25	0.484	1.21
PA5186_at	30.32	35.17	0.730	-1.16
PA5187_at	32.94	40.88	0.151	-1.24
PA5188_at	34.52	22.60	0.008	1.53
PA5189_at	13.62	11.80	0.484	1.15
PA5190_at	96.80	106.92	0.151	-1.10
PA5191_at	75.74	68.80	0.349	1.10
PA5192_pckA_at	998.98	1374.23	0.008	-1.38
PA5193_yrfI_at	317.00	328.70	0.905	-1.04
PA5194_at	131.90	211.18	0.024	-1.60
PA5195_at	83.02	76.18	0.151	1.09
PA5196_at	118.40	103.80	0.238	1.14
PA5197_rimK_at	34.40	33.73	0.643	1.02
PA5198_at	72.62	56.35	0.048	1.29
PA5199_envZ_at	203.22	140.42	0.111	1.45
PA5200_ompR_at	591.18	359.72	0.008	1.64
PA5201_at	519.68	785.42	0.008	-1.51
PA5202_at	119.02	140.00	0.484	-1.18
PA5203_gshA_at	144.32	181.08	0.008	-1.25
PA5204_argA_at	106.06	86.47	0.238	1.23
PA5205_at	41.68	43.57	1.000	-1.05
PA5206_argE_at	141.32	91.58	0.008	1.54
PA5207_at	15.64	7.22	0.008	2.17
PA5208_at	43.76	85.00	0.048	-1.94
PA5209_at	112.00	98.48	0.151	1.14
PA5210_at	151.10	140.77	0.817	1.07
PA5211_at	44.36	32.05	0.008	1.38
PA5212_i_at	284.18	189.63	0.048	1.50
PA5213_gcvP1_at	21.08	35.63	0.048	-1.69
PA5214_gcvH1_at	499.62	499.63	1.000	-1.00
PA5215_gcvT1_at	295.90	417.90	0.008	-1.41
PA5216_at	68.68	74.93	0.238	-1.09
PA5217_at	454.02	445.82	0.643	1.02

Supplementary Table 1. Probe set signal data for experimental and control samples

PA5218_at	17.76	32.00	0.024	-1.80
PA5219_at	49.28	35.30	0.087	1.40
PA5220_at	128.34	112.90	0.151	1.14
PA5221_at	90.42	92.23	0.984	-1.02
PA5222_at	222.54	220.80	1.000	1.01
PA5223_ubiH_at	180.42	166.95	0.087	1.08
PA5224_pepP_at	265.76	271.92	0.817	-1.02
PA5225_at	376.94	432.90	0.238	-1.15
PA5226_at	151.70	216.60	0.087	-1.43
PA5227_at	203.94	200.40	0.643	1.02
PA5228_at	38.88	44.38	0.048	-1.14
PA5229_at	71.58	63.78	0.349	1.12
PA5230_at	44.86	60.10	0.111	-1.34
PA5231_at	113.88	130.13	0.151	-1.14
PA5232_at	148.46	209.98	0.008	-1.41
PA5233_at	119.60	115.65	1.000	1.03
PA5234_at	62.60	64.28	0.643	-1.03
PA5235_glpT_at	17.92	68.13	0.008	-3.80
PA5236_at	143.62	137.48	0.556	1.04
PA5237_at	275.70	237.15	0.151	1.16
PA5238_at	22.80	17.30	0.484	1.32
PA5239_rho_at	339.72	543.15	0.008	-1.60
PA5240_trxA_at	463.98	628.95	0.349	-1.36
PA5241_ppx_at	255.34	221.80	0.087	1.15
PA5242_ppk_at	376.86	353.67	0.643	1.07
PA5243_hemB_at	393.56	382.15	0.817	1.03
PA5244_at	64.60	63.85	0.643	1.01
PA5245_at	295.18	248.75	0.008	1.19
PA5246_at	16.96	24.30	0.008	-1.43
PA5247_at	93.32	97.72	0.643	-1.05
PA5248_at	62.02	68.77	0.413	-1.11
PA5249_at	16.28	9.38	0.286	1.74
PA5250_at	126.58	154.15	0.048	-1.22
PA5251_at	101.92	120.58	0.151	-1.18
PA5252_at	272.72	298.45	0.238	-1.09
PA5253_algP_at	1771.72	1554.43	0.008	1.14
PA5254_at	58.98	35.10	0.048	1.68
PA5255_algQ_at	576.78	492.92	0.238	1.17
PA5256_dsbH_at	68.92	71.95	0.817	-1.04
PA5257_at	91.28	82.65	0.349	1.10
PA5258_at	183.06	171.57	0.151	1.07
PA5259_hemD_at	81.26	95.55	0.087	-1.18
PA5260_hemC_at	213.16	201.02	0.484	1.06
PA5261_algR_at	105.00	102.63	0.817	1.02
PA5262_algZ_at	73.68	65.60	0.151	1.12
PA5263_argH_at	674.68	629.25	0.048	1.07
PA5264_at	18.22	13.42	0.349	1.36
PA5265_at	2.58	9.07	0.048	-3.52
PA5266_at	31.52	19.27	0.048	1.64
PA5268_corA_at	133.48	112.65	0.048	1.18
PA5269_at	59.20	67.53	0.349	-1.14

Supplementary Table 1. Probe set signal data for experimental and control samples

PA5270_at	190.18	128.52	0.008	1.48
PA5271_at	80.32	124.85	0.008	-1.55
PA5272_cyaA_at	51.90	46.25	0.349	1.12
PA5273_at	90.24	68.10	0.024	1.33
PA5274_rnk_at	594.72	564.03	0.643	1.05
PA5275_at	41.50	36.15	0.484	1.15
PA5276_lppL_i_at	901.80	1342.63	0.008	-1.49
PA5277_lysA_at	552.08	581.75	0.484	-1.05
PA5278_dapF_at	308.40	305.90	0.643	1.01
PA5279_at	587.22	489.90	0.087	1.20
PA5280_sss_at	133.70	115.22	0.087	1.16
PA5281_at	60.36	66.15	0.484	-1.10
PA5282_at	27.70	18.73	0.238	1.48
PA5283_at	28.72	27.40	1.000	1.05
PA5284_at	13.24	11.43	1.000	1.16
PA5285_at	831.24	1504.47	0.008	-1.81
PA5286_at	457.96	571.95	0.048	-1.25
PA5287_amtB_at	44.10	43.22	0.643	1.02
PA5288_glnK_at	395.70	523.23	0.048	-1.32
PA5289_at	433.88	364.15	0.238	1.19
PA5290_at	54.78	58.30	0.349	-1.06
PA5291_at	71.18	85.88	0.151	-1.21
PA5292_at	29.42	27.25	0.484	1.08
PA5293_at	34.12	37.50	0.190	-1.10
PA5294_at	11.14	7.70	0.349	1.45
PA5295_at	141.58	129.45	0.151	1.09
PA5296_rep_at	93.68	130.50	0.008	-1.39
PA5297_poxB_at	13.12	29.53	0.151	-2.25
PA5298_at	333.64	570.25	0.008	-1.71
PA5299_at	43.32	40.65	0.238	1.07
PA5300_cycB_at	416.96	792.70	0.008	-1.90
PA5301_at	535.96	503.00	0.484	1.07
PA5302_dadX_at	363.22	573.13	0.008	-1.58
PA5303_at	791.48	1107.03	0.024	-1.40
PA5304_dadA_at	1501.42	1624.20	0.349	-1.08
PA5305_at	90.40	109.33	0.087	-1.21
PA5306_at	213.74	367.70	0.008	-1.72
PA5307_at	42.24	42.00	1.000	1.01
PA5308_lrp_at	101.54	150.73	0.024	-1.48
PA5309_at	73.40	82.03	0.484	-1.12
PA5310_at	36.64	43.63	0.484	-1.19
PA5311_at	7.94	6.85	1.000	1.16
PA5312_at	828.26	444.92	0.008	1.86
PA5313_at	122.02	78.60	0.048	1.55
PA5314_at	15.10	22.90	0.151	-1.52
PA5315_rpmG_at	340.32	761.45	0.008	-2.24
PA5316_rpmB_at	2833.02	3578.78	0.048	-1.26
PA5317_at	123.34	95.40	0.087	1.29
PA5318_at	57.50	36.97	0.024	1.56
PA5319_radC_at	54.00	53.55	0.817	1.01
PA5320_dfp_at	791.64	757.40	0.484	1.05

Supplementary Table 1. Probe set signal data for experimental and control samples

PA5321_dut_at	160.92	226.82	0.087	-1.41
PA5322_algC_at	446.14	466.40	0.484	-1.05
PA5323_argB_at	129.56	221.77	0.008	-1.71
PA5324_at	50.04	54.40	0.341	-1.09
PA5325_at	8.82	12.88	0.349	-1.46
PA5326_at	9.40	10.45	0.817	-1.11
PA5327_at	22.16	27.55	0.151	-1.24
PA5328_at	16.90	9.42	0.151	1.79
PA5329_at	66.54	54.47	0.238	1.22
PA5330_at	96.30	146.05	0.008	-1.52
PA5331_pyrE_at	295.22	364.90	0.008	-1.24
PA5332_crc_at	673.82	913.88	0.008	-1.36
PA5333_at	79.62	117.88	0.008	-1.48
PA5334_rph_at	315.66	422.90	0.008	-1.34
PA5335_at	381.20	330.97	0.151	1.15
PA5336_gmk_at	358.98	387.85	0.238	-1.08
PA5337_rpoZ_at	659.66	1018.60	0.008	-1.54
PA5338_spoT_at	557.70	512.22	0.238	1.09
PA5339_at	802.08	1238.85	0.008	-1.54
PA5340_at	412.70	570.78	0.008	-1.38
PA5341_at	14.10	11.70	0.413	1.21
PA5342_at	31.52	43.00	0.048	-1.36
PA5343_at	110.24	87.32	0.151	1.26
PA5344_at	552.94	387.15	0.008	1.43
PA5345_recG_at	225.12	169.05	0.024	1.33
PA5346_at	279.08	247.38	0.008	1.13
PA5347_at	74.04	115.52	0.008	-1.56
PA5348_at	1866.20	1198.03	0.238	1.56
PA5349_at	99.78	103.35	0.817	-1.04
PA5350_r_at	314.68	337.55	0.048	-1.07
PA5351_at	155.74	337.70	0.008	-2.17
PA5352_at	18.84	20.20	0.817	-1.07
PA5353_glcF_at	11.22	11.35	1.000	-1.01
PA5354_glcE_at	21.22	15.50	0.484	1.37
PA5355_glcD_at	28.76	24.75	0.238	1.16
PA5356_glcC_at	88.20	69.32	0.048	1.27
PA5357_at	49.24	71.95	0.008	-1.46
PA5358_ubiA_at	110.64	103.77	0.484	1.07
PA5359_at	39.20	28.90	0.016	1.36
PA5360_phoB_at	130.92	166.75	0.008	-1.27
PA5361_phoR_at	9.04	21.83	0.048	-2.41
PA5362_at	191.88	182.77	0.817	1.05
PA5363_at	88.88	85.47	0.238	1.04
PA5364_at	185.58	135.88	0.048	1.37
PA5365_phoU_at	104.14	120.13	0.643	-1.15
PA5366_pstB_at	175.98	220.10	0.087	-1.25
PA5367_pstA_at	102.62	138.10	0.048	-1.35
PA5368_pstC_at	94.48	115.75	0.087	-1.23
PA5369_at	229.60	266.60	0.238	-1.16
PA5370_at	134.38	121.67	0.484	1.10
PA5371_at	61.26	89.68	0.048	-1.46

Supplementary Table 1. Probe set signal data for experimental and control samples

PA5372_betA_at	39.12	32.33	0.087	1.21
PA5373_betB_at	439.04	230.60	0.008	1.90
PA5374_betI_at	308.80	183.40	0.008	1.68
PA5375_betT1_at	61.78	33.90	0.024	1.82
PA5376_at	53.22	46.25	0.238	1.15
PA5377_at	191.00	122.93	0.008	1.55
PA5378_at	77.14	75.85	1.000	1.02
PA5379_sdaB_at	46.24	30.45	0.024	1.52
PA5380_at	280.72	140.32	0.008	2.00
PA5381_at	18.50	15.45	0.643	1.20
PA5382_at	22.56	32.10	0.151	-1.42
PA5383_at	14.88	13.68	0.643	1.09
PA5384_at	9.16	4.55	0.286	2.01
PA5385_at	3.16	18.42	0.008	-5.83
PA5386_at	6.32	10.77	0.151	-1.70
PA5387_at	17.06	19.95	0.817	-1.17
PA5388_at	16.22	18.27	0.484	-1.13
PA5389_at	36.48	34.20	0.817	1.07
PA5390_at	10.46	8.65	0.984	1.21
PA5391_at	16.10	13.27	0.643	1.21
PA5392_at	15.66	9.75	0.190	1.61
PA5393_at	27.92	12.72	0.048	2.19
PA5394_cls_at	31.72	35.03	0.484	-1.10
PA5395_at	15.74	13.88	0.413	1.13
PA5396_at	159.98	95.03	0.008	1.68
PA5397_at	120.42	99.47	0.151	1.21
PA5398_at	54.50	36.85	0.024	1.48
PA5399_at	41.90	24.20	0.048	1.73
PA5400_at	29.54	18.10	0.008	1.63
PA5401_at	48.24	23.20	0.008	2.08
PA5402_at	33.90	47.82	0.087	-1.41
PA5403_at	26.36	40.72	0.008	-1.54
PA5404_at	19.64	41.33	0.008	-2.10
PA5405_i_at	16.20	35.00	0.048	-2.16
PA5406_at	113.64	181.95	0.024	-1.60
PA5407_at	64.46	158.52	0.008	-2.46
PA5408_at	40.44	48.80	0.484	-1.21
PA5409_at	31.54	27.42	0.151	1.15
PA5410_at	118.16	101.47	0.484	1.16
PA5411_at	73.56	57.38	0.008	1.28
PA5412_at	51.56	62.55	0.008	-1.21
PA5413_ltaA_at	171.98	227.28	0.048	-1.32
PA5414_at	640.34	615.25	0.817	1.04
PA5415_glyA1_s_at	574.96	483.10	0.151	1.19
PA5416_soxB_at	81.08	53.25	0.024	1.52
PA5417_soxD_at	62.54	59.97	0.643	1.04
PA5418_soxA_at	70.86	56.83	0.413	1.25
PA5419_soxG_at	160.74	87.27	0.024	1.84
PA5420_purU2_at	46.10	44.40	0.817	1.04
PA5421_fdhA_at	53.80	57.00	0.643	-1.06
PA5422_at	108.12	100.40	0.349	1.08

Supplementary Table 1. Probe set signal data for experimental and control samples

PA5423_at	183.64	162.60	0.349	1.13
PA5424_at	78.00	97.90	0.008	-1.26
PA5425_purK_at	168.28	320.48	0.008	-1.90
PA5426_purE_at	613.16	1031.63	0.008	-1.68
PA5427_adhA_at	69.42	63.83	0.643	1.09
PA5428_at	55.54	62.50	0.087	-1.13
PA5429_aspA_at	1014.74	1528.43	0.008	-1.51
PA5430_at	94.14	110.97	0.238	-1.18
PA5431_at	20.24	17.32	0.484	1.17
PA5432_at	93.74	74.00	0.151	1.27
PA5433_at	36.20	34.03	0.643	1.06
PA5434_mtr_at	27.02	39.45	0.151	-1.46
PA5435_at	176.96	276.15	0.048	-1.56
PA5436_at	852.30	839.85	0.817	1.01
PA5437_at	74.38	84.17	0.151	-1.13
PA5438_at	268.78	278.58	0.484	-1.04
PA5439_at	41.68	36.50	0.087	1.14
PA5440_at	70.52	128.13	0.024	-1.82
PA5441_at	262.24	272.77	0.817	-1.04
PA5442_at	61.48	35.45	0.008	1.73
PA5443_uvrD_at	243.00	240.27	1.000	1.01
PA5444_at	21.60	22.65	1.000	-1.05
PA5445_at	311.88	335.20	0.643	-1.07
PA5446_i_at	385.10	1019.50	0.048	-2.65
PA5447_wbpZ_at	55.02	61.33	0.484	-1.11
PA5448_wbpY_at	98.64	55.35	0.008	1.78
PA5449_wbpX_at	99.96	88.82	0.349	1.13
PA5450_wzt_at	192.04	102.90	0.008	1.87
PA5451_wzm_at	79.82	69.10	0.349	1.16
PA5452_wbpW_at	109.72	48.35	0.008	2.27
PA5453_gmd_at	316.76	139.82	0.008	2.27
PA5454_rmd_at	113.32	69.13	0.024	1.64
PA5455_at	259.40	192.22	0.151	1.35
PA5456_at	312.52	198.30	0.024	1.58
PA5457_at	115.88	94.85	0.024	1.22
PA5458_at	150.06	97.75	0.008	1.54
PA5459_at	131.44	140.50	0.484	-1.07
PA5460_at	9.26	11.68	0.349	-1.26
PA5461_at	531.86	785.35	0.024	-1.48
PA5462_at	363.94	314.70	0.048	1.16
PA5463_at	212.32	250.40	0.008	-1.18
PA5464_at	84.32	91.85	0.413	-1.09
PA5465_at	126.00	62.50	0.024	2.02
PA5466_at	44.66	30.60	0.008	1.46
PA5467_at	24.38	19.67	0.151	1.24
PA5468_at	16.10	23.47	0.087	-1.46
PA5469_at	10.38	15.35	0.349	-1.48
PA5470_at	185.60	45.70	0.024	4.06
PA5471_at	538.56	130.10	0.024	4.14
PA5472_at	166.88	191.90	0.238	-1.15
PA5473_at	27.84	24.20	0.349	1.15

Supplementary Table 1. Probe set signal data for experimental and control samples

PA5474_at	83.18	71.97	0.484	1.16
PA5475_at	41.76	48.55	0.349	-1.16
PA5476_citA_at	15.80	17.98	0.238	-1.14
PA5477_at	32.98	46.53	0.008	-1.41
PA5478_at	64.40	81.75	0.048	-1.27
PA5479_gltP_at	124.52	396.22	0.008	-3.18
PA5480_at	4.06	4.97	0.484	-1.22
PA5481_at	25.76	17.45	0.238	1.48
PA5482_at	45.34	25.67	0.087	1.77
PA5483_algB_at	121.92	110.02	0.238	1.11
PA5484_at	61.68	62.57	1.000	-1.01
PA5485_at	89.44	106.72	0.087	-1.19
PA5486_at	43.48	49.78	0.238	-1.14
PA5487_at	150.02	194.25	0.087	-1.29
PA5488_at	553.12	477.75	0.008	1.16
PA5489_dsbA_at	869.64	738.53	0.024	1.18
PA5490_cc4_at	2000.46	1934.30	1.000	1.03
PA5491_at	943.78	985.85	1.000	-1.04
PA5492_at	297.18	373.90	0.008	-1.26
PA5493_polA_at	221.66	239.57	0.151	-1.08
PA5494_at	239.10	324.98	0.024	-1.36
PA5495_thrB_at	402.52	313.07	0.008	1.29
PA5496_at	42.80	51.35	0.349	-1.20
PA5497_at	65.30	72.15	0.643	-1.10
PA5498_at	82.32	96.75	0.151	-1.18
PA5499_np20_at	251.04	235.50	0.349	1.07
PA5500_znuC_at	216.58	173.93	0.151	1.25
PA5501_znuB_at	62.10	65.98	0.643	-1.06
PA5502_at	227.68	190.32	0.087	1.20
PA5503_at	241.82	302.55	0.024	-1.25
PA5504_at	297.32	403.45	0.008	-1.36
PA5505_at	401.00	718.60	0.008	-1.79
PA5506_at	853.04	457.15	0.008	1.87
PA5507_at	1211.20	905.37	0.008	1.34
PA5508_at	821.08	570.30	0.048	1.44
PA5509_at	461.28	322.20	0.008	1.43
PA5510_at	127.18	100.72	0.008	1.26
PA5511_at	33.32	54.60	0.008	-1.64
PA5512_at	72.98	55.82	0.048	1.31
PA5513_at	119.60	110.63	0.484	1.08
PA5514_at	7.38	10.43	0.349	-1.41
PA5515_at	92.84	63.38	0.008	1.46
PA5516_pdxY_at	117.06	111.60	0.484	1.05
PA5517_at	37.46	37.08	0.643	1.01
PA5518_at	40.10	46.68	0.151	-1.16
PA5519_at	90.90	135.88	0.008	-1.49
PA5520_at	16.98	23.90	0.349	-1.41
PA5521_at	53.76	54.67	0.817	-1.02
PA5522_at	58.16	42.18	0.048	1.38
PA5523_at	86.36	51.85	0.008	1.67
PA5524_at	43.88	56.25	0.048	-1.28

Supplementary Table 1. Probe set signal data for experimental and control samples

PA5525_at	40.92	45.88	0.484	-1.12
PA5526_at	37.10	51.28	0.151	-1.38
PA5527_at	91.88	142.65	0.008	-1.55
PA5528_at	921.58	556.10	0.024	1.66
PA5529_at	95.04	59.45	0.048	1.60
PA5530_at	610.10	24.70	0.008	24.70
PA5531_tonB_at	355.66	463.95	0.048	-1.30
PA5532_at	16.32	8.93	0.151	1.83
PA5533_at	76.92	90.53	0.238	-1.18
PA5534_at	6.52	8.63	0.484	-1.32
PA5535_at	15.86	16.73	0.643	-1.05
PA5536_at	6.90	15.88	0.048	-2.30
PA5537_at	19.20	16.60	0.817	1.16
PA5538_amiA_at	8.94	7.38	0.643	1.21
PA5539_at	21.94	19.85	0.817	1.11
PA5540_at	21.22	24.88	0.484	-1.17
PA5541_at	22.76	19.73	0.905	1.15
PA5542_at	23.52	17.30	0.349	1.36
PA5543_at	72.90	44.97	0.008	1.62
PA5544_at	35.02	31.50	0.484	1.11
PA5545_at	162.72	106.73	0.048	1.52
PA5546_at	83.36	83.93	0.817	-1.01
PA5547_at	41.46	51.50	0.087	-1.24
PA5548_at	17.08	23.40	0.238	-1.37
PA5549_glmS_at	156.24	239.40	0.008	-1.53
PA5550_at	143.38	194.82	0.008	-1.36
PA5551_at	119.54	174.57	0.008	-1.46
PA5552_glmU_at	533.56	575.22	0.151	-1.08
PA5553_atpC_at	561.72	806.93	0.008	-1.44
PA5554_atpD_at	1037.06	1240.22	0.024	-1.20
PA5555_atpG_at	4236.76	3767.47	0.151	1.12
PA5556_atpA_at	4420.42	3018.92	0.008	1.46
PA5557_atpH_at	4279.04	3760.07	0.087	1.14
PA5558_atpF_at	6139.24	4532.70	0.024	1.35
PA5559_atpE_at	3632.70	3653.45	0.817	-1.01
PA5560_atpB_at	1423.86	2250.20	0.008	-1.58
PA5561_atpI_at	186.00	345.05	0.008	-1.86
PA5562_spoOJ_at	276.22	422.70	0.008	-1.53
PA5563_soj_at	607.10	692.17	0.643	-1.14
PA5564_gidB_at	405.72	590.90	0.008	-1.46
PA5565_gidA_at	464.54	538.63	0.151	-1.16
PA5566_at	6.36	7.80	0.341	-1.23
PA5567_at	81.84	117.47	0.008	-1.44
PA5568_at	1133.60	1302.40	0.008	-1.15
PA5569_rnpA_at	2750.88	2581.73	0.024	1.07
PA5570_rpmH_at	988.28	1398.30	0.008	-1.41

* The fold change is a positive number when the expression level in the experiment increased compared to the control and is a negative number when the expression level in the experiment declined.
