# Supplementary material for: Microarray analysis of Pseudomonas aeruginosa reveals induction of pyocin genes in response to hydrogen peroxide
Source: BMC Genomics. 2005 Sep 8;6:115. doi: 10.1186/1471-2164-6-115 (PMC1250226; doi:10.1186/1471-2164-6-115)
Supplement: Additional File 2 — Comparison of the fold changes and their directions of the significantly marked genes in this study with those in Palma et al. [12](a total of 223 genes). The Mann-Whitney test with the cutoff p and fold change values of 0.05 and 2, respectively, was applied to both studies, [file 1471-2164-6-115-S2.pdf]

Supplementary Table 2. Comparison of the fold changes and their directions of the significantly marked genes in this study with those in Palma *et al*. [12]

Gene (Name)	Fold change *		Direction	
	This study	Palma *et al*. [12]	This study	Palma *et al*. [12]
PA0069	2.3	7.0	Upregulation	Upregulation
PA0128	-2.3	Not significant	Downregulation	
PA0162	-2.2	Not significant	Downregulation	
PA0182	6.8	Not significant	Upregulation	
PA0280 (cysA)	-2.9	-5.0	Downregulation	Downregulation
PA0281 (cysW)	-2.8	Not significant	Downregulation	
PA0282 (cysT)	-2.5	-8.6	Downregulation	Downregulation
PA0283 (sbp)	-2.0	Not significant	Downregulation	
PA0284	-2.3	Not significant	Downregulation	
PA0335	2.0	Not significant	Upregulation	
PA0352	-2.1	-3.7	Downregulation	Downregulation
PA0385	-2.1	-2.4	Downregulation	Downregulation
PA0447 (gcdH)	2.2	-4.7	Upregulation	Downregulation
PA0492	2.0	2.9	Upregulation	Upregulation
PA0493	2.1	8.0	Upregulation	Upregulation
PA0494	2.3	4.6	Upregulation	Upregulation
PA0495	2.1	2.9	Upregulation	Upregulation
PA0582 (folB)	-2.4	-5.3	Downregulation	Downregulation
PA0586	2.4	3.2	Upregulation	Upregulation
PA0587	2.2	3.9	Upregulation	Upregulation
PA0612	5.3	Not significant	Upregulation	
PA0613	2.3	Not significant	Upregulation	
PA0614	2.9	Not significant	Upregulation	
PA0615	4.2	Not significant	Upregulation	
PA0616	3.5	Not significant	Upregulation	
PA0617	3.4	Not significant	Upregulation	
PA0618	3.1	Not significant	Upregulation	
PA0619	3.0	Not significant	Upregulation	
PA0620	4.2	Not significant	Upregulation	
PA0621	2.6	Not significant	Upregulation	
PA0622	3.0	Not significant	Upregulation	
PA0623	2.8	Not significant	Upregulation	
PA0624	2.5	Not significant	Upregulation	
PA0625	3.4	Not significant	Upregulation	
PA0626	2.3	Not significant	Upregulation	
PA0627	2.3	Not significant	Upregulation	
PA0628	2.2	Not significant	Upregulation	
PA0629	2.6	Not significant	Upregulation	
PA0630	2.0	Not significant	Upregulation	
PA0631	2.3	Not significant	Upregulation	
PA0633	3.3	Not significant	Upregulation	
PA0634	3.5	Not significant	Upregulation	
PA0635	3.6	Not significant	Upregulation	
PA0636	3.4	Not significant	Upregulation	
PA0637	3.3	Not significant	Upregulation	
PA0638	3.2	Not significant	Upregulation	
PA0639	3.0	Not significant	Upregulation	
PA0640	2.6	Not significant	Upregulation	
PA0641	5.0	Not significant	Upregulation	
PA0642	2.3	Not significant	Upregulation	

Supplementary Table 2. Comparison of the fold changes and their directions of the significantly marked genes in this study with those in Palma *et al*. [12]

PA0643	2.1	Not significant	Upregulation	
PA0645	2.5	Not significant	Upregulation	
PA0646	2.5	Not significant	Upregulation	
PA0669	2.8	2.1	Upregulation	Upregulation
PA0670	5.4	7.0	Upregulation	Upregulation
PA0671	5.02	22.9	Upregulation	Upregulation
PA0745	3.0	Not significant	Upregulation	
PA0746	3.1	Not significant	Upregulation	
PA0747	2.4	2.6	Upregulation	Upregulation
PA0866 (aroP2)	2.3	-2.8	Upregulation	Downregulation
PA0873 (phhR)	2.0	Not significant	Upregulation	
PA0887 (acsA)	2.0	Not significant	Upregulation	
PA0905 (csrA)	-2.5	Not significant	Downregulation	
PA0915	-2.0	-21.7	Downregulation	Downregulation
PA0916	-2.2	-11.2	Downregulation	Downregulation
PA0922	3.7	10.0	Upregulation	Upregulation
PA0975	-2.4	-3.2	Downregulation	Downregulation
PA0976	-2.7	Not significant	Downregulation	
PA0983	2.0	5.2	Upregulation	Upregulation
PA0996	-2.0	-2.3	Downregulation	Downregulation
PA1000	-2.4	Not significant	Downregulation	
PA1001 (phnA)	-2.0	Not significant	Downregulation	
PA1002 (phnB)	-2.1	Not significant	Downregulation	
PA1041	2.2	4.1	Upregulation	Upregulation
PA1112	2.5	Not significant	Upregulation	
PA1150 (pys2)	2.9	Not significant	Upregulation	
PA1151 (imm2)	-2.1	Not significant	Downregulation	
PA1228	-2.8	-4.1	Downregulation	Downregulation
PA1317 (cyoA)	-2.3	-2.5	Downregulation	Downregulation
PA1319 (cyoC)	-2.1	Not significant	Downregulation	
PA1372	-2.1	Not significant	Downregulation	
PA1408	2.0	2.1	Upregulation	Upregulation
PA1414	-2.9	8.7	Downregulation	Upregulation
PA1432 (lasI)	-2.0	Not significant	Downregulation	
PA1466	13.9	12.6	Upregulation	Upregulation
PA1574	-2.4	-5.4	Downregulation	Downregulation
PA1592	-2.1	Not significant	Downregulation	
PA1634 (kdpB)	-2.3	Not significant	Downregulation	
PA1673	-2.0	3.5	Downregulation	Upregulation
PA1687 (speE)	-2.6	-7.6	Downregulation	Downregulation
PA1759	2.3	3.9	Upregulation	Upregulation
PA1760	2.8	Not significant	Upregulation	
PA1776	-2.0	Not significant	Downregulation	
PA1837	-2.1	-7.5	Downregulation	Downregulation
PA1845	-2.3	Not significant	Downregulation	
PA1852	-2.0	-2.3	Downregulation	Downregulation
PA1865	3.1	3.6	Upregulation	Upregulation
PA1946 (rbsB)	2.7	5.0	Upregulation	Upregulation
PA2006	2.1	Not significant	Upregulation	
PA2007 (maiA)	2.0	Not significant	Upregulation	
PA2008 (fahA)	2.2	Not significant	Upregulation	
PA2009 (hmgA)	2.2	Not significant	Upregulation	

Supplementary Table 2. Comparison of the fold changes and their directions of the significantly marked genes in this study with those in Palma *et al*. [12]

PA2012	2.0	-2.2	Upregulation	Downregulation
PA2247 (bkdA1)	2.1	2.4	Upregulation	Upregulation
PA2248 (bkdA2)	2.5	Not significant	Upregulation	
PA2288	10.3	23.5	Upregulation	Upregulation
PA2398 (fpvA)	-5.7	Not significant	Downregulation	
PA2403	-3.2	-2.6	Downregulation	Downregulation
PA2404	-3.0	-3.8	Downregulation	Downregulation
PA2405	-3.9	-4.9	Downregulation	Downregulation
PA2407	-3.3	-4.5	Downregulation	Downregulation
PA2408	-2.9	-12.0	Downregulation	Downregulation
PA2409	-3.7	Not significant	Downregulation	
PA2410	-2.5	Not significant	Downregulation	
PA2426 (pvdS)	-3.0	12.7	Downregulation	Upregulation
PA2453	-2.0	-3.5	Downregulation	Downregulation
PA2523	2.0	4.0	Upregulation	Upregulation
PA2552	2.3	Not significant	Upregulation	
PA2553	2.5	Not significant	Upregulation	
PA2555	2.2	3.1	Upregulation	Upregulation
PA2557	2.0	5.4	Upregulation	Upregulation
PA2579	-2.0	Not significant	Downregulation	
PA2619 (infA)	-2.7	-5.9	Downregulation	Downregulation
PA2630	-2.4	-10.4	Downregulation	Downregulation
PA2653	-2.3	-5.1	Downregulation	Downregulation
PA2667	-3.0	Not significant	Downregulation	
PA2730	-2.2	Not significant	Downregulation	
PA3007 (lexA)	4.0	11.5	Upregulation	Upregulation
PA3008	5.7	13.9	Upregulation	Upregulation
PA3009	-3.1	Not significant	Downregulation	
PA3080	2.7	Not significant	Upregulation	
PA3147 (wbpJ)	-2.0	Not significant	Downregulation	
PA3149 (wbpH)	-2.1	Not significant	Downregulation	
PA3150 (wbpG)	-2.3	Not significant	Downregulation	
PA3235	2.0	3.6	Upregulation	Upregulation
PA3281	2.2	5.9	Upregulation	Upregulation
PA3282	2.5	7.4	Upregulation	Upregulation
PA3283	3.1	10.3	Upregulation	Upregulation
PA3284	2.3	4.4	Upregulation	Upregulation
PA3404	2.1	Not significant	Upregulation	
PA3413	4.4	2.9	Upregulation	Upregulation
PA3414	9.8	Not significant	Upregulation	
PA3417	2.2	5.3	Upregulation	Upregulation
PA3496	-2.4	3.5	Downregulation	Upregulation
PA3531 (bfrB)	-2.0	Not significant	Downregulation	
PA3574	-2.3	Not significant	Downregulation	
PA3607	-2.18	-5.5	Downregulation	Downregulation
PA3608	-2.5	-21.5	Downregulation	Downregulation
PA3609	-2.07	-6.1	Downregulation	Downregulation
PA3610	-2.99	-12.6	Downregulation	Downregulation
PA3610 (potD)	-3.0	-12.6	Downregulation	Downregulation
PA3616	2.8	Not significant	Upregulation	
PA3617 (recA)	2.7	2.8	Upregulation	Upregulation
PA3621 (fdxA)	-2.0	-4.5	Downregulation	Downregulation

Supplementary Table 2. Comparison of the fold changes and their directions of the significantly marked genes in this study with those in Palma *et al*. [12]

PA3672	2.0	Not significant	Upregulation	
PA3712	2.5	Not significant	Upregulation	
PA3713	-2.1	-8.7	Downregulation	Downregulation
PA3767	-2.2	Not significant	Downregulation	
PA3787	2.2	Not significant	Upregulation	
PA3808	-2.0	-2.4	Downregulation	Downregulation
PA3866	2.2	Not significant	Upregulation	
PA3901 (fecA)	2.1	Not significant	Upregulation	
PA3913	-2.1	Not significant	Downregulation	
PA3922	2.0	-2.3	Upregulation	Downregulation
PA3923	3.3	Not significant	Upregulation	
PA3967	-2.5	-7.4	Downregulation	Downregulation
PA4132	-2.2	-3.4	Downregulation	Downregulation
PA4133	-2.3	Not significant	Downregulation	
PA4141	-2.1	Not significant	Downregulation	
PA4156	-5.1	12.4	Downregulation	Upregulation
PA4199	2.3	Not significant	Upregulation	
PA4218	-2.6	Not significant	Downregulation	
PA4219	-2.5	29.4	Downregulation	Upregulation
PA4221 (fptA)	-2.7	44.1	Downregulation	Upregulation
PA4222	-2.1	Not significant	Downregulation	
PA4223	-2.6	Not significant	Downregulation	
PA4224	-2.4	Not significant	Downregulation	
PA4225 (pchF)	-3.3	Not significant	Downregulation	
PA4226 (pchE)	-2.6	Not significant	Downregulation	
PA4228 (pchD)	-2.3	15.2	Downregulation	Upregulation
PA4229 (pchC)	-2.6	Not significant	Downregulation	
PA4230 (pchB)	-4.3	14.5	Downregulation	Upregulation
PA4231 (pchA)	-2.9	Not significant	Downregulation	
PA4236 (katA)	2.0	34.1	Upregulation	Upregulation
PA4390	-2.4	-3.1	Downregulation	Downregulation
PA4427 (sspB)	-2.0	-2.2	Downregulation	Downregulation
PA4432 (rpsI)	-2.3	-7.2	Downregulation	Downregulation
PA4496	2.1	Not significant	Upregulation	
PA4553 (pilX)	-2.0	Not significant	Downregulation	
PA4555 (pilY2)	-3.0	2.1	Downregulation	Upregulation
PA4563 (rpsT)	-2.3	-8.9	Downregulation	Downregulation
PA4582	3.2	21.4	Upregulation	Upregulation
PA4583	2.6	14.0	Upregulation	Upregulation
PA4584	3.0	7.7	Upregulation	Upregulation
PA4585 (rtcA)	2.0	Not significant	Upregulation	
PA4613 (katB)	4.5	56.9	Upregulation	Upregulation
PA4642	-2.3	Not significant	Downregulation	
PA4645	-2.2	-5.3	Downregulation	Downregulation
PA4673	-2.1	-19.2	Downregulation	Downregulation
PA4719	-2.2	-5.9	Downregulation	Downregulation
PA4747 (secG)	-2.6	-3.1	Downregulation	Downregulation
PA4753	-2.3	-3.0	Downregulation	Downregulation
PA4759 (dapB)	-2.3	Not significant	Downregulation	
PA4763 (recN)	8.3	11.7	Upregulation	Upregulation
PA4839 (speA)	-2.9	-11.4	Downregulation	Downregulation
PA4840	-2.5	-3.3	Downregulation	Downregulation

Supplementary Table 2. Comparison of the fold changes and their directions of the significantly marked genes in this study with those in Palma *et al*. [12]

PA4843	2.0	Not significant	Upregulation	
PA5049 (rpmE)	-2.8	-5.9	Downregulation	Downregulation
PA5167	2.5	Not significant	Upregulation	
PA5235 (glpT)	-3.4	Not significant	Downregulation	
PA5315 (rpmG)	-2.2	-7.4	Downregulation	Downregulation
PA5351	-2.2	-4.8	Downregulation	Downregulation
PA5380	2.0	2.4	Upregulation	Upregulation
PA5401	2.1	Not significant	Upregulation	
PA5407	-2.5	-5.1	Downregulation	Downregulation
PA5446	-2.7	3.7	Downregulation	Upregulation
PA5452 (wbpW)	2.3	Not significant	Upregulation	
PA5453 (gmd)	2.3	Not significant	Upregulation	
PA5465	2.0	3.5	Upregulation	Upregulation
PA5470	4.1	Not significant	Upregulation	
PA5471	4.1	Not significant	Upregulation	
PA5479 (gltP)	-3.2	-19.3	Downregulation	Downregulation
PA5530	24.7	Not significant	Upregulation	

* The fold change is a positive number when the expression level in the experiment increased compared to the control and is a negative number when the expression level in the experiment declined.
