# Supplementary material for: Microarray analysis of Pseudomonas aeruginosa reveals induction of pyocin genes in response to hydrogen peroxide
Source: BMC Genomics. 2005 Sep 8;6:115. doi: 10.1186/1471-2164-6-115 (PMC1250226; doi:10.1186/1471-2164-6-115)
Supplement: Additional File 3 — Comparison of the expression change directions of genes that we discussed in this study with those in Palma et al. [12]. The genes were classified into the categories of "primary metabolism-related", "cellular protective mechanisms-related", "iron regulation-related", and "pyocin system-related", as presented in Table 3. [file 1471-2164-6-115-S3.pdf]

Supplementary Table 3. Comparison of the expression change directions of genes that we discussed in this study with those in Palma *et al.* [12]

Gene (Name)	Direction of expression change		Comparison between this study and Palma *et al*. [12] (in the order named)				
			Agreement		Disagreement		
	This study	Palma *et al*. [12]	Upregulation-Upregulation	Downregulation-Downregulation	Downregulation-Upregulation	Upregulation-No significance	Downregulation-No significance
Primary metabolism-related							
PA1317 (*cyoA*)	Downregulation	Downregulation		X			
PA1319 (*cyoC*)	Downregulation						X
PA3621 (*fdxA*)	Downregulation	Downregulation		X			
PA4133	Downregulation						X
PA0654 (*speD*)							
PA1687 (*speE*)	Downregulation	Downregulation		X			
PA4839 (*speA*)	Downregulation	Downregulation		X			
PA3607 (*potA*)	Downregulation	Downregulation		X			
PA3608 (*potB*)	Downregulation	Downregulation		X			
PA3609 (*potC*)	Downregulation	Downregulation		X			
PA3610 (*potD*)	Downregulation	Downregulation		X			
PA4432 (*rpsL*)	Downregulation	Downregulation		X			
PA4563 (*rpsT*)	Downregulation	Downregulation		X			
PA5049 (*rpmE*)	Downregulation	Downregulation		X			
PA5315 (*rpmG*)	Downregulation	Downregulation		X			
PA0671	Upregulation	Upregulation	X				
PA3008	Upregulation	Upregulation	X				
Cellular protective mechanism-related							
PA4236 (*katA*)	Upregulation	Upregulation	X				
PA4613 (*katB*)	Upregulation	Upregulation	X				
PA4366 (*sodB*)							
PA4468 (*sodM*)							
PA3007 (*lexA*)	Upregulation	Upregulation	X				
PA3008	Upregulation	Upregulation	X				
PA3616	Upregulation					X	
PA3617 (*recA*)	Upregulation	Upregulation	X				
PA0669	Upregulation	Upregulation	X				
PA3413	Upregulation	Upregulation	X				
PA3414	Upregulation	Upregulation	X				
PA4763 (*recN)*	Upregulation	Upregulation	X				
Iron regulation-related							
PA2426 (*pvdS*)	Downregulation	Upregulation			X		
PA2398 (*fpvA*)	Downregulation						X
PA4221 (*fptA*)	Downregulation	Upregulation			X		
PA4225 (*pchF*)	Downregulation						X
PA4226 (*pchE*)	Downregulation						X
PA4228 (*pchD*)	Downregulation	Upregulation			X		

Supplementary Table 3. Comparison of the expression change directions of genes that we discussed in this study with those in Palma *et al.* [12]

PA4229 (*pchC*)	Downregulation					X
PA4230 (*pchB*)	Downregulation	Upregulation		X		
PA4231 (*pchA*)	Downregulation					X
PA2403	Downregulation	Downregulation	X			
PA2404	Downregulation	Downregulation	X			
PA2405	Downregulation	Downregulation	X			
PA2406						
PA2407	Downregulation	Downregulation	X			
PA2408	Downregulation	Downregulation	X			
PA2409	Downregulation					X
PA2410	Downregulation					X
PA4156	Downregulation	Upregulation		X		
PA3531 (*bfrB*)	Downregulation					X
Pyocin system-related						
PA0985						
PA1150 (*pys2*)	Upregulation				X	
PA3866	Upregulation				X	
PA0612	Upregulation				X	
PA0613	Upregulation				X	
PA0614	Upregulation				X	
PA0615	Upregulation				X	
PA0616	Upregulation				X	
PA0617	Upregulation				X	
PA0618	Upregulation				X	
PA0619	Upregulation				X	
PA0620	Upregulation				X	
PA0621	Upregulation				X	
PA0622	Upregulation				X	
PA0623	Upregulation				X	
PA0624	Upregulation				X	
PA0625	Upregulation				X	
PA0626	Upregulation				X	
PA0627	Upregulation				X	
PA0628	Upregulation				X	
PA0629	Upregulation				X	
PA0630	Upregulation				X	
PA0631	Upregulation				X	
PA0632	Upregulation				X	
PA0633	Upregulation				X	
PA0634	Upregulation				X	
PA0635	Upregulation				X	
PA0636	Upregulation				X	

Supplementary Table 3. Comparison of the expression change directions of genes that we discussed in this study with those in Palma *et al.* [12]

PA0637	Upregulation	X	
PA0638	Upregulation	X	
PA0639	Upregulation	X	
PA0640	Upregulation	X	
PA0641	Upregulation	X	
PA0642	Upregulation	X	
PA0643	Upregulation	X	
PA0644			
PA0645	Upregulation	X	
PA0646	Upregulation	X	
PA0647			
PA0648			
PA1151 (*imm2*)	Downregulation		X
